# Development of a propionate metabolism-related gene-based molecular subtypes and scoring system for predicting prognosis in bladder cancer

**DOI:** 10.1186/s40001-024-01982-6

**Published:** 2024-07-29

**Authors:** Fuchun Zheng, Zhipeng Wang, Sheng Li, Situ Xiong, Yuyang Yuan, Jin Zeng, Yifan Tan, Xiaoqiang Liu, Songhui Xu, Bin Fu

**Affiliations:** 1 Department of Urology, The First Affiliated Hospital, Jiangxi Medical College, Nanchang University, Nanchang, 330000 China; 2 Jiangxi Institute of Urology, Nanchang, China

**Keywords:** Bladder cancer, Propionate, Prognosis, Risk signature, Target

## Abstract

**Purpose:**

Bladder cancer (BLCA) is a prevalent malignancy. Dysregulated propionate metabolism, a key cancer factor, suggests a potential target for treating metastatic cancer. However, a complete understanding of the link between propionate metabolism-related genes (PMRGs) and bladder cancer is lacking.

**Methods:**

From the Cancer Genome Atlas (TCGA) and Gene Expression Omnibus (GEO) databases, we gathered BLCA patient data, which was classified into distinct subgroups using non-negative matrix factorization (NMF). Survival and pathway analyses were conducted between these clusters. The PMRGs model, created through univariate Cox and least absolute shrinkage and selection operator (LASSO) analyses, was assessed for prognostic significance using Kaplan–Meier and receiver operating characteristic (ROC) curves. A comprehensive evaluation included clinical, tumor microenvironment (TME), drug sensitivity, and immunotherapy analyses. Finally, the expression of HSD17B1 essential genes was confirmed via quantitative real-time polymerase chain reaction (qRT-PCR), with further validation through Transwell, wound healing, colony-formation, and EDU assays.

**Results:**

We discovered two distinct subcategories (CA and CB) within BLCA using NMF analysis, with CA demonstrating significantly better overall survival compared to CB. Additionally, six PMRGs emerged as critical factors associated with propionate metabolism and prognosis. Kaplan–Meier analysis revealed that high-risk PMRGs were correlated with a poorer prognosis in BLCA patients. Moreover, significant differences were observed between the two groups in terms of infiltrated immune cells, immune checkpoint expression, TME scores, and drug sensitivity. Notably, we found that suppressing HSD17B1 gene expression inhibited the invasion of bladder cancer cells.

**Conclusion:**

Our study proposes molecular subtypes and a PMRG-based score as promising prognostic indicators in BLCA. Additionally, cellular experiments underscore the pivotal role of HSD17B1 in bladder cancer metastasis and invasion, suggesting its potential as a novel therapeutic target.

**Supplementary Information:**

The online version contains supplementary material available at 10.1186/s40001-024-01982-6.

## Introduction

Bladder cancer (BLCA) comprises 3% of newly diagnosed cancer cases globally, standing as the eleventh most prevalent cancer type with an annual incidence of 573,000 new cases [1]. The predisposing factors for BLCA encompass genetic elements, environmental and work-related encounters, tobacco use, and conditions related to excessive body weight [2]. Non-muscle-invasive BLCA exhibits a favorable prognosis, boasting an approximate 90% 5-year overall survival (OS) rate. In contrast, metastatic BLCA is characterized by a markedly lower OS, falling below 6% [3]. Roughly half of individuals with clinically confined muscle-invasive BLCA encounter instances of disease recurrence and metastasis [4]. Hence, it is crucial to investigate biomarkers capable of accurately assessing patient prognosis.

The ability to metabolize propionate is a crucial aspect of a microorganism’s metabolic adaptability, enabling the utilization of carbon sources that generate propionyl-CoA in various environmental and host settings [5]. Recent findings indicate that propionate promotes lipid accumulation in adipocytes and demonstrates a significant correlation between serum propionate levels and human obesity [6]. Moreover, propionate exhibits antiapoptotic marker inhibition and induces proapoptotic proteins at both the gene and protein levels, suggesting its potential as a therapeutic option for cervical cancer [7]. Studies have uncovered that disrupting propionate metabolism contributes to a pro-aggressive profile in breast and lung cancer cells, enhancing their metastatic capabilities [8]. However, a research gap remains concerning propionate metabolism-related genes (PMRGs) in the context of BLCA onset and progression.

This study focuses on unraveling the role of PMRGs in BLCA pathogenesis. Leveraging comprehensive genomic datasets, such as those from TCGA and GEO, we aim to construct a robust PMRG gene model. This model promises to elucidate the intricate molecular landscape of BLCA and holds the potential to serve as a predictive tool and guide personalized therapeutic interventions for improved clinical outcomes.

## Methods

### Data collection

Obtain RNA-sequencing data and clinical annotations for 19 normal tissue specimens and 412 BLCA specimens from the TCGA database (https://portal.gdc.cancer.gov/). Concurrently, retrieve the gene expression dataset GSE13507 [9], consisting of 256 BLCA samples, from the GEO repository (https://www.ncbi.nlm.nih.gov/gds). Additionally, gather a set of 812 PMRGs (relevance score > 5) from the GeneCards database (https://www.genecards.org; Table S1).

### Analysis of prognostic relevance and consensus clustering analysis

Initially, We used the ‘limma’ R package [10] to analyze PMRG differential expression in TCGA-BLCA and to identify significant genes. The merged TCGA and GEO datasets with survival data underwent univariate regression analysis using R packages “survival [11]” and “forest plot [12]” to identify prognostic genes. A circular plot visualized significant gene expression differences in normal and tumor tissues. Subsequently, we classified 662 tumor patients into subgroups based on 20 PMRGs using “ConsensusClusterPlus,” from Bioconductor [13] employing PCA, tSNE, and UAMP for accuracy. Kaplan–Meier curves were generated to compare survival outcomes among subtypes. Furthermore, immune-correlation analysis, GO analysis, and KEGG analysis were conducted. Immune-correlation analysis studies how various immune factors relate to each other, shedding light on their roles in diseases and treatment strategies.

### Development of a prognostic signature utilizing PMRGs

Patients from TCGA and GEO databases were randomly split into control and validation groups (1:1 ratio). Lasso regression and multivariable analysis optimized the predictive risk model. The risk score for each sample was calculated using the following formula: coefficient X1 * expression X1 + coefficient X2 * expression X2 + $\cdots \cdots$ + coefficient Xn * expression Xn. The risk score for each sample was calculated using the coefficients 1.005111879 of the regression model obtained and patients were categorized into low-risk and high-risk groups [14, 15]. Visualization depicted survival status, risk gene expressions, and scores across risk groups, with Kaplan–Meier curves illustrating OS rates.

### Constructing and assessing a predictive nomogram

Through multivariate Cox regression analysis, we crafted a nomogram to depict the predictive factors succinctly. Internal validation ensued through calibration plots to verify the nomogram’s accuracy. Decision curve analysis (DCA) was employed to evaluate clinical net benefits [16]. Boxplots were utilized to visually represent the correlation between the scoring system and clinical baseline characteristics alongside pathological features.

### Immune cell signature analyses

Initially, the CIBERSORT [17] algorithm within the R package CIBERSORT assessed the prevalence of 22 tumor-infiltrating immune cells in BLCA samples. Subsequently, we examined variations in immune cell infiltration between groups with high and low risk. Further analysis delved into visual representations of correlations among the 20 immune cells, the six hub genes, and risk scores. Additionally, we employed the ESTIMATE [18] method to calculate the stromal score, immune score, and overall ESTIMATE score.

### Drug sensitivity analysis

We utilized the R package “pRRophetic [19]” to forecast the IC50 values for frequently prescribed drugs in BLCA, discerning variations between high- and low-score groups. This assessment aimed to gauge the distinct sensitivities to various chemotherapy drugs. Statistical significance was established at a *p* value below 0.05, visually depicted through box plots.

### Tumor immune single cell hub (TISCH) database and validation of the HSD17B1

Access RNA-seq data for single cells from the TISCH database (http://tisch.comp-genomics.org), an online resource dedicated to the tumor microenvironment (TME) [20]. The TISCH database, employed in studying TME heterogeneity across diverse datasets and cell types, was a valuable resource. The KM plotter dataset was utilized to validate the predictive risk model. Perform a comprehensive pan-cancer analysis for HSD17B1 using the TIMER database (http://timer.cistrome.org) and evaluate its expression levels in both tumor and normal tissues (https://www.r-project.org/). Verify the protein expression patterns of prognostic genes by consulting the HPA dataset, accessible at https://www.proteinatlas.org/ [21].

### Validation of HSD17B1 expression in BLCA samples

BCLA samples and adjacent tissues were procured from ten recently diagnosed BCLA patients at the First Affiliated Hospital of Nanchang University in Nanchang, China. Total RNA extraction was performed using Invitrogen TRIzol reagent, and cDNA synthesis was accomplished using the Takara PrimeScript RT reagent Kit. Real-time quantitative PCR was employed for gene expression analysis, employing SYBR Green from Roche, Switzerland, with β-actin as an endogenous reference. Each reaction underwent at least three replications. The primer sequences used were HSD17B1_F: CCTCTGTGCTGGACGTGAAT, HSD17B1_R: GCTGGCGCAATAAACGTCAT.

### Cell culture and small interfering ribonucleic acid (siRNA) transfection

BLCA cell lines T24 and BIU were obtained from Procell Life Science & Technology Co., Ltd (Wuhan, China) and the cell bank of the Type Culture Collection of the Chinese Academy of Sciences (Shanghai, China), respectively. The cells were cultured in RPMI-DMEM or 1640 medium (Gibco, USA) supplemented with 10% fetal bovine serum at 37 °C in a humidified atmosphere with 5% CO_2_. Subsequently, cells were seeded into 6-well plates at a concentration of 40% to 60%. SiRNAs targeting HSD17B1 and corresponding controls were synthesized by GenePharma Co., Ltd. (Shanghai, China) and transiently transfected using Lipofectamine 2000 (Invitrogen) according to the manufacturer’s instructions. The specific sequences of the siRNAs were as follows: HSD17B1 siRNA-1 (5′-GCUUCAAAGUGUAUGCCACTT-3′) and HSD17B1 siRNA-2 (5′-GUGGGUGGCUAAUUAAGAUTT-3′).

### Cell migration was assessed through wound-healing and transwell assays

T24 and BIU cells were seeded into 6-well plates and underwent siRNA transfection. Upon reaching a cell density exceeding 90%, a linear wound was created using a 200-μl pipette tip. Subsequently, the medium was substituted with a serum-free culture medium and placed in a cell culture incubator for incubation. Photographic documentation was conducted at 0 h and 24 h to compare scratch healing rates among various groups. In the Transwell assay, cells were suspended in serum-free DMEM or RPMI-1640 medium and seeded onto the upper surface of Transwell chambers, while the lower chamber was filled with medium supplemented with FBS. After a 24-h incubation period, cells attached to the lower membrane were immobilized using 4% paraformaldehyde and subsequently stained with crystal violet. Following this, optical microscope imaging was conducted to capture photographs of the cells.

### Cell proliferation was evaluated via EDU and colony-formation assays

Approximately 1000 transfected cells were seeded in 6-well plates containing culture medium supplemented with 10% FBS and incubated for 7–10 days. Subsequently, the cells were treated with 4% paraformaldehyde solution and fixed for 20 min, after which they were stained with 1% crystal violet solution for 30 min. Additionally, transfected cells were plated at a density of 5000 cells per well in a 96-well plate. After 24 h of incubation, the culture medium was replaced with EdU for an additional 2 h, and staining was performed according to the instructions in the EDU assay kit (C10310-1, RiboBio). Imaging was conducted using a confocal microscope.

### Statistical analysis

R software (Version 4.3.1, https://www.r-project.org/) and GraphPad Prism 8 were employed for data analysis in this study. Statistical significance was determined by two-sided *p*-values less than 0.05. Notably, all experiments were conducted thrice to ensure the reliability and consistency of the findings.

## Results

### Identification of differentially expressed PMRGs and molecular subtyping

**Fig. 1:**
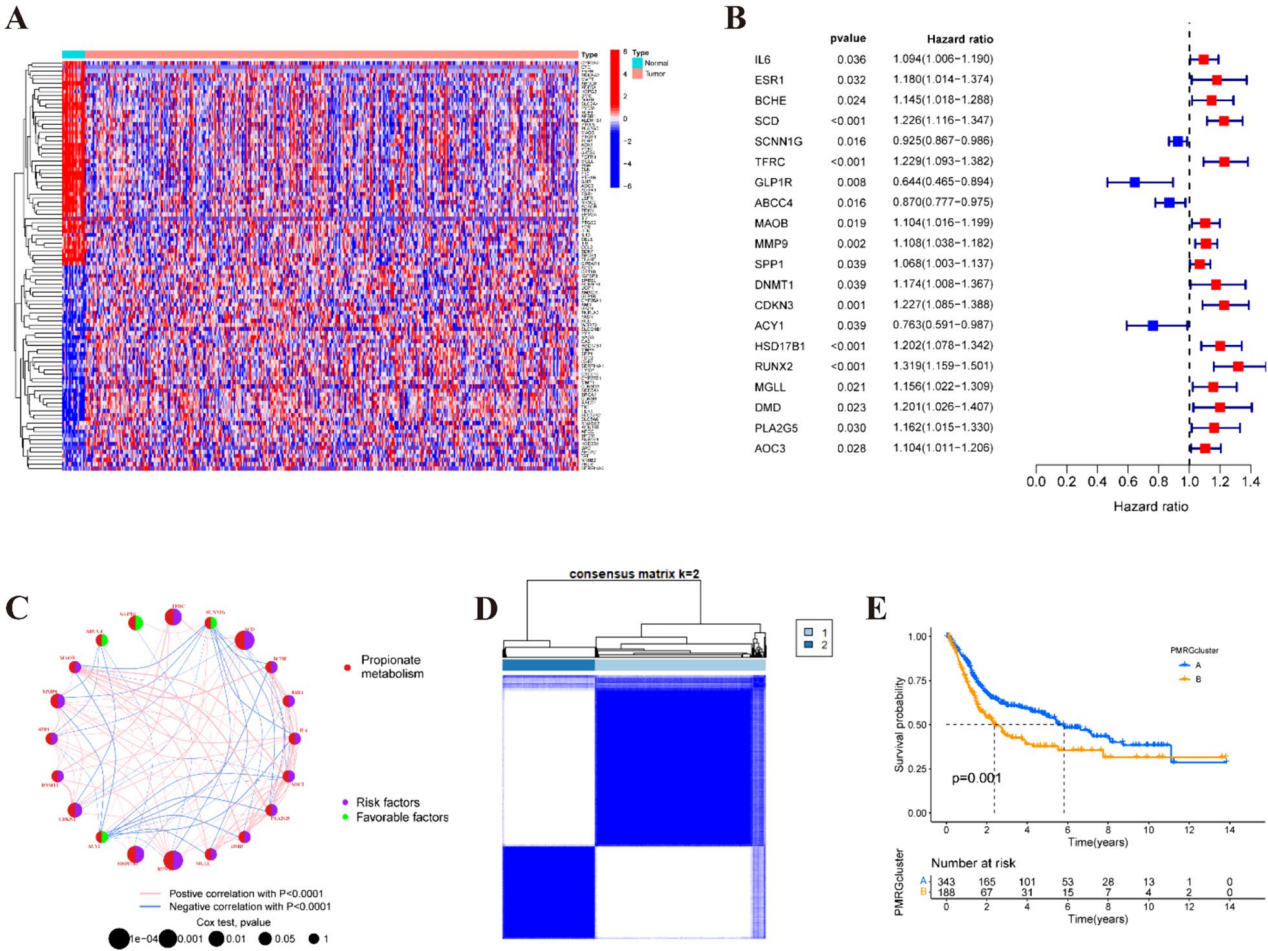
Identification of differentially expressed PMRGs and molecular subtyping. **A** Heatmap of differentially expressed PMRGs. **B** Univariate Cox analysis demonstrated the correlation between propionate metabolism-associated genes and prognosis. **C** A circle plot illustrates the correlations among these PMRGs. **D** The consensus clustering in BLCA samples with *k* = 2. **E** Kaplan–Meier (KM) survival analysis of two subgroups

The entire protocol is delineated in Fig. S1. In the first step, a total of 812 PMRGs were retrieved from the GeneCard database (Table S1). Subsequently, RNA-seq data from the TCGA-BLCA dataset were retrieved, and a differential expression analysis was performed. Following integration with the GSE13507 dataset, 169 differentially expressed PMRGs were identified (Fig. 1A and Fig. S2A). Clinical information from 537 BLCA patients in the TCGA-BLCA database and the GSE13507 dataset was then integrated, and their survival data were utilized for univariate Cox regression analysis, resulting in the discovery of 20 PMRGs (Fig. 1B, C). To gain deeper insights into the functional role of PMRGs in BLCA, we employed the ‘Consensus Cluster Plus R program to assess the 20 PMRGs identified through univariate Cox analysis (Fig. 1D and Fig. S2B–G). Our analysis revealed the segregation of BLCA samples into two distinct subtypes when *k* = 2. Furthermore, survival analysis revealed a notable contrast in prognosis between these two subgroups (Fig. 1E). Notably, increased expression of the upper subset of PMRGs in cluster B was associated with a poorer prognosis in BLCA. Additionally, the efficacy of the non-negative matrix factorization method was validated through PCA, tSNE, and UMAP analyses (Fig. S2H–J).

### Characterizing the biological properties of PMRG subtypes

**Fig. 2:**
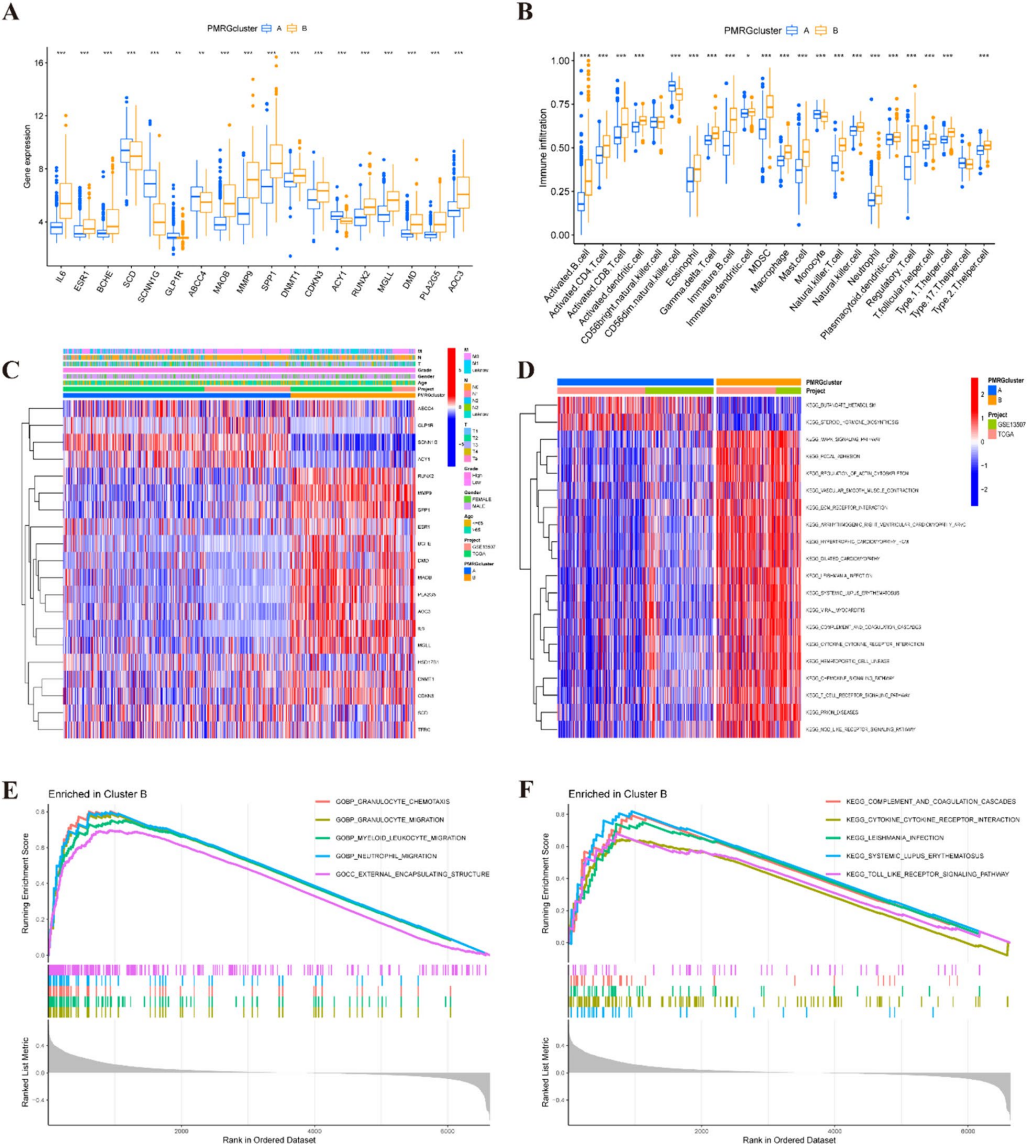
The functional analysis of PMRGs. **A** The 18 differential expressed genes between the PMRGs cluster A and B. **B** Boxplots depicting the 23 immune signature ssGSEA scores of the PMRGs cluster A and B. **C** Difference distribution of clinicopathological features and ARGs expression among the two subtypes. **D** The gene set variation analysis (GSVA) of the differences in KEGG pathways within subgroups A and B. **E**, **F** GSEA between clusters A and B

In our analysis, we identified 18 differentially expressed genes between clusters A and B of PMRGs (Fig. 2A). Additionally, we employed the CIBERSORT algorithm. We employed the CIBERSORT algorithm to estimate the levels of various cell infiltrations in BLCA samples, revealing significant distinctions in the proportions of immune cell and stromal cell infiltration ratios between clusters C1 and C2. Notably, cluster B exhibited higher levels of infiltration of activated B cells, CD4 and CD8 T cells, dendritic cells, eosinophils, macrophages, mast cells, natural killer T cells, neutrophils, regulatory T cells, and T helper cells, while cluster A showed abundance in CD56dim natural killer cells and monocytes (Fig. 2B). Furthermore, the patterns observed in PMRGs cluster B were associated with tumor stages and grades (Fig. 2C). Using gene set variation analysis (GSVA), we investigated the disparate enrichment of KEGG pathways within subgroups A and B. Notably, a robust correlation was observed between the adverse prognosis of cluster B and the heightened expression of associated pathways, including MAPK signaling, chemokine signaling, T cell receptor signaling, and NOD-like receptor signaling pathways (Fig. 2D). Additionally, gene set enrichment analysis (GSEA) revealed that enriched pathways such as granulocyte chemotaxis, external encapsulating structure, cytokine–cytokine receptor interaction, and toll-like receptor signaling pathway may also contribute to the poor prognosis of cluster B for BLCA patients (Fig. 2E, F).

### Construction and validation of the PMRGs signature

**Fig. 3:**
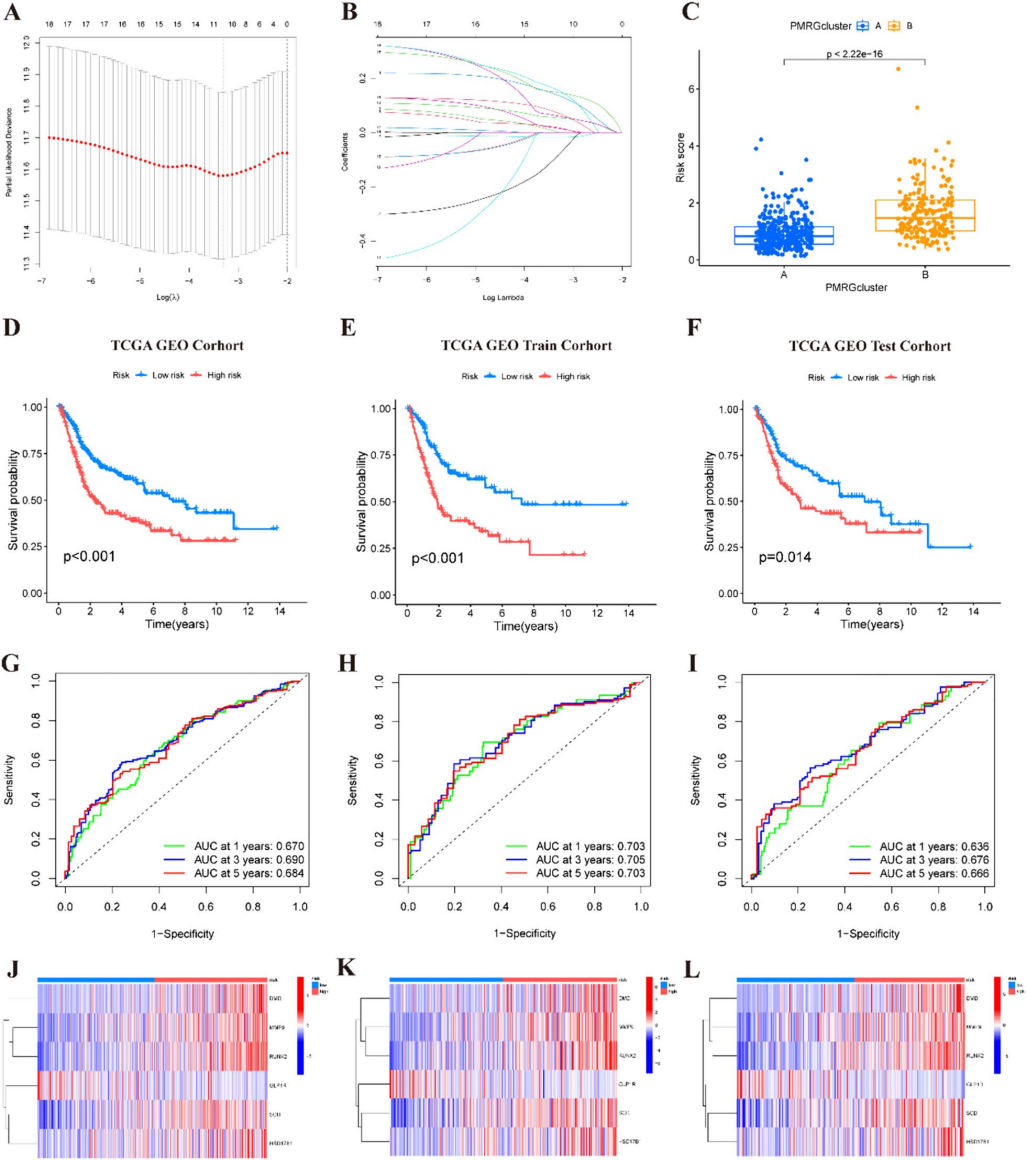
Establishment of a risk score signature based on PMRGs. **A** Plots of the coefficient profiles of six prognostic PMRGs. **B** The least absolute shrinkage and selection operator (LASSO) analysis identified six prognostic genes with tenfold cross-validation. C The risk score of clusters A and B. **D**–**F** The Kaplan–Meier survival curve of the OS rate in different subgroups of patients with BLCA. **G**–**I** 1-, 3-, and 5-year ROC curves of the all training and testing set. **J**–**L** The heat map of 6 PMRGs expression in each subgroup

531 patients diagnosed with BLCA from the TCGA-BLCA dataset and GSE13507 were randomly divided into a training group (*n* = 266) and a validation group (*n* = 265). Through initial univariate Cox analysis followed by LASSO analysis, overfit genes were effectively eliminated, as depicted in Fig. 3A, B. Subsequently, a multivariate Cox analysis was conducted, resulting in the identification of a prognostic signature comprising six PMRGs: SCD, GLP1R, MMP9, HSD17B1, RUNX2, and DMD. Utilizing the derived formula: risk score = (0.226 × SCD expression) + (− 0.334 × GLP1R expression) + (0.091 × MMP9 expression) + (0.152 × HSD17B1 expression) + (0.219 × RUNX2 expression) + (0.322 × DMD expression), the risk scores for each patient were computed. As a result, the patients were grouped by the median risk score 1.005111879 into high- and low-risk categories. Cluster B exhibited higher risk scores than Cluster A (Fig. 3C), substantiating the poorer prognosis associated with Cluster B in BLCA patients. Kaplan–Meier survival analysis revealed a better prognosis for the low-risk group (Fig. 3D). To validate the robustness of these findings, the BLCA patients were further randomly divided into training and test cohorts. KM survival analysis on both cohorts consistently supported the earlier conclusions (Fig. 3E, F). Additionally, ROC curves for 1-, 3-, and 5-year OS yielded areas under the curve (AUC) of 0.670, 0.690, and 0.684, respectively (Fig. 3G). Notably, these results were corroborated in both the train and test cohorts (Fig. 3H, I), underscoring the predictive accuracy of the established prognostic signature across varying time frames. Moreover, the expression levels of PMRGs, including SCD, MMP9, HSD17B1, RUNX2, and DMD, were notably elevated in high-risk patients, indicative of their association with poorer prognosis. Conversely, GLP1R expression exhibited a protective effect as a prognostic predictor (Fig. 3J–L). In pursuit of precise survival risk predictions, we further investigated the efficacy of the risk signature in predicting BLCA progression. Our exploration into the relationship between the risk score and clinicopathological characteristics revealed significant distinctions among different groups concerning age, grade, and TNM stage (all *p* < 0.05). However, no discernible correlation was observed between the risk score and gender (*p* > 0.05; Fig. S3A–F). These findings collectively emphasize the effectiveness of the established prognostic signature in predicting BLCA patient survival across different time points, highlighting its clinical relevance and potential utility in guiding patient management strategies.

### Establishment of a nomogram

**Fig. 4:**
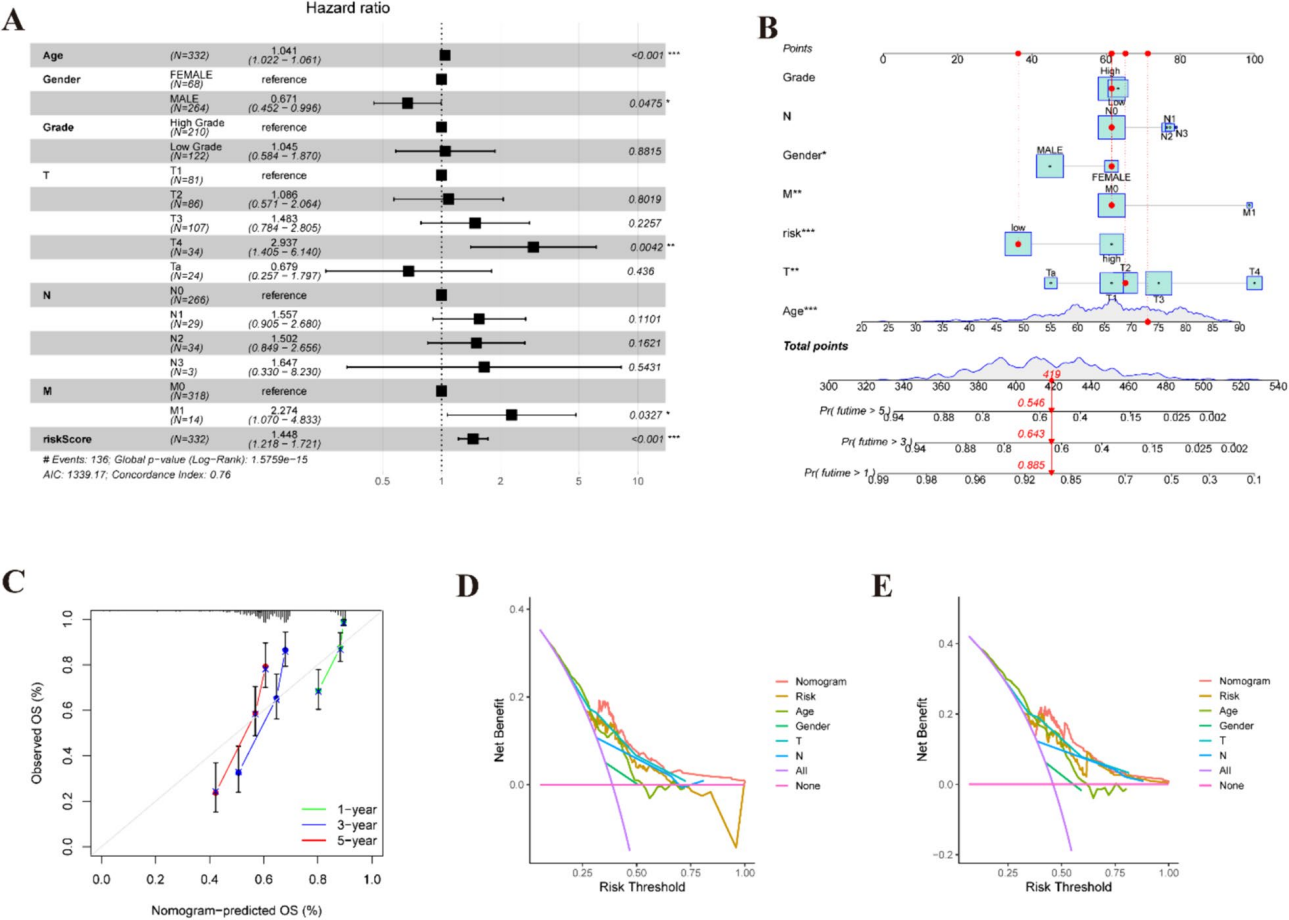
An analysis of the predictive value of risk scores in BLCA patients from the TCGA and GEO datasets. **A** Assessment of clinical features and risk scores using a multivariate Cox analysis. **B** Predictive nomogram for survival over 1, 3, and 5 years based on risk groupings and clinical characteristics. ***P < 0.001. **C** Comparison of actual and predicted outcomes at 1, 3, and 5 years based on calibration curves. The DCA of risk scores and clinical features at 3 D and 5 E years

The outcomes of the multivariate Cox analysis underscored a significant correlation between the risk score derived from the propionate metabolism-related gene signature (PMRS) and prognosis, as illustrated in Fig. 4A, affirming PMRS as an independent prognostic determinant. To refine prognostic evaluation, we devised a novel nomogram amalgamating PMRS with clinical features, showcased in Fig. 4B, which demonstrated commendable predictive accuracy validated by calibration curves (Fig. 4C). Furthermore, DCA underscored the favorable prognostic performance of the nomogram across 3- and 5-year intervals (Fig. 4D, E). In summary, our findings accentuate the effectiveness of PMRS in precisely forecasting outcomes for BLCA patients, thereby furnishing crucial insights for clinical prognosis assessment.

### Immune landscape

**Fig. 5:**
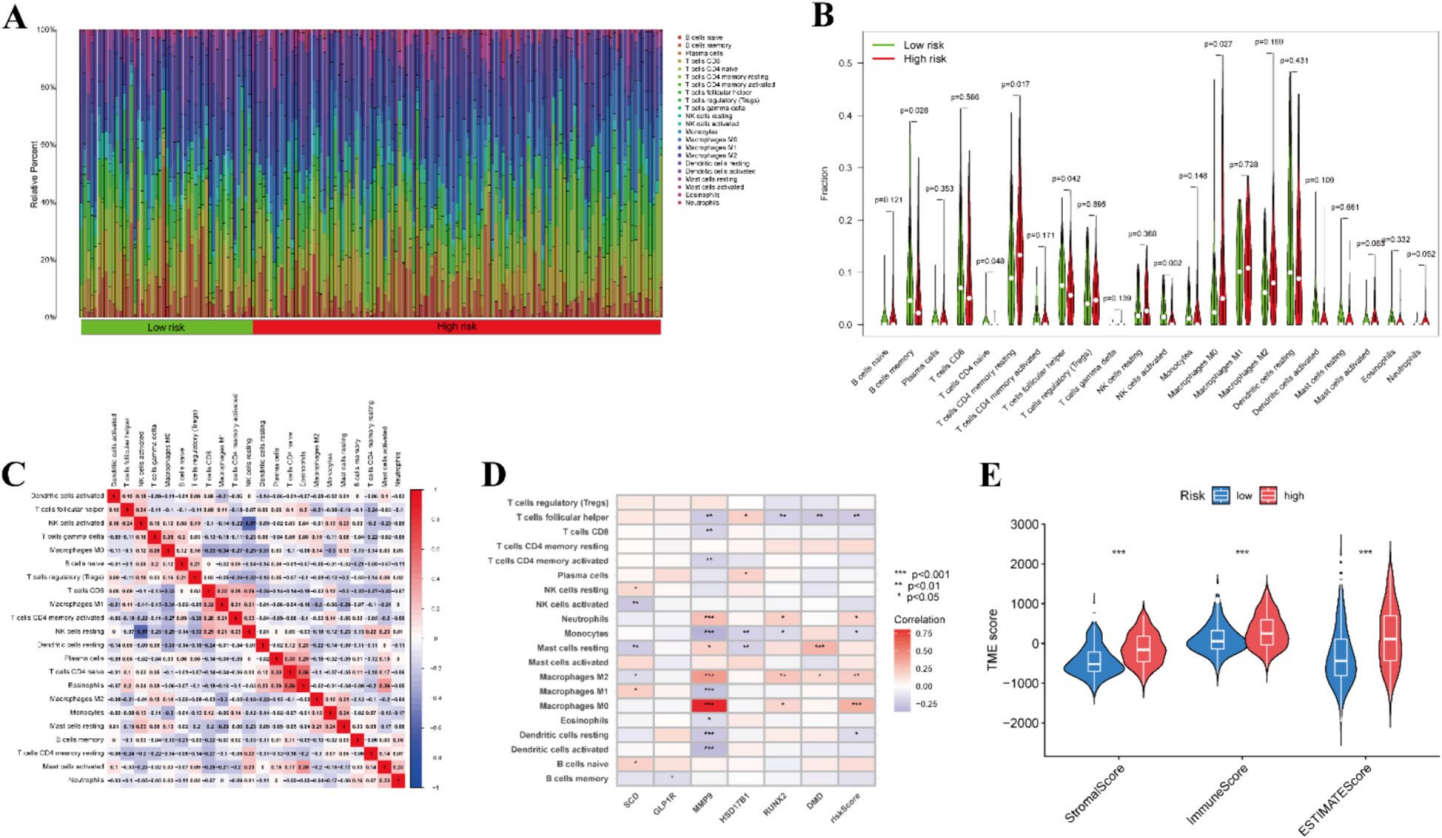
Tumor microenvironment cell infiltration. **A**–**C** An illustration of the fractions of 22 types of infiltrating immune cells for low and high-risk patients. **D** A relation between infiltrating immune cells and six prognostic PMRGs, along with the risk score. **E** TME scores between the low and high-risk patients

We conducted an evaluation and visualization of the proportions of 22 immune cell types in both high- and low-risk categorized patients, as depicted in Fig. 5A. Comparison of immune cell abundance revealed higher levels of T cells CD4 memory resting, T cells follicular helper, and macrophages in high-risk patients. In contrast, memory B cells and NK cells activated were more prevalent in the low-risk group (Fig. 5B). Furthermore, correlation analysis unveiled inverse relationships between most immune cell types (Fig. 5C). Spearman correlation analyses highlighted significant associations between 6 molecular entities and immune cell composition (Fig. 5D and Fig. S3G–L). Particularly noteworthy was the strong positive correlation between neutrophils, M0 macrophages, and M2 macrophages with most genes, while T cells follicular helper and monocytes showed an inverse correlation (*p* < 0.05). In addition, TME scores based on stromal score, immune score, and estimated score of the high-risk group surpassed those of the low-risk group (Fig. 5E). In summary, our risk model accurately predicts immune response activity in BLCA patients and provides valuable insights into the efficacy of immune checkpoint inhibition therapy.

### Drug sensitivity

We explored differences in drug sensitivity between two BLCA patient groups based on PMRS scoring to improve treatment outcomes. As depicted in Fig. S4A–I, the IC50 values of cyclophosphamide, dasatinib, and staurosporine were lower in the high-scoring group compared to the low-scoring group, suggesting heightened sensitivity to these drugs in the former. Conversely, the IC50 values of afatinib, leflunomide, oxaliplatin, palbociclib, selumetinib, and trametinib were lower in the low-scoring group relative to the high-scoring group, indicating greater sensitivity to these drugs in the latter. This nuanced understanding of drug sensitivity profiles offers valuable insights for optimizing treatment strategies tailored to individual patient characteristics.

### Single-cell analysis and validation of the HSD17B1 from external databases

Given the prognostic significance and distinctive distribution of HSD17B1 in BLCA tissues, we delved into its enrichment across specific cell types utilizing scRNA-seq data from the GSE145281_aPDL1 dataset. This analysis revealed 16 cell clusters and 5 cell types within BLCA tissues, as depicted in Fig. S5A–D, with corresponding marker genes delineated in Fig. S5E. Notably, HSD17B1 demonstrated significant enrichment in CD4Tconv, CD8T, macrophage, and NK cell populations (Fig. S5F). To validate the expression patterns of vital prognostic PMRGs, we leveraged the TISCH database to scrutinize HSD17B1 expression in both BLCA and pan-cancer contexts, as illustrated in Fig. S5G and I. Analysis using the KM plotter dataset unveiled an association between high HSD17B1 expression and poorer patient prognosis (Fig. S5H). Additionally, we corroborated these findings using the HPA database, which demonstrated elevated protein expression of HSD17B1 in BLCA compared to normal tissue, as showcased in Fig. S5J.

### Utilizing HSD17B1 as the core gene of the scoring system and conducting in vitro validation

**Fig. 6:**
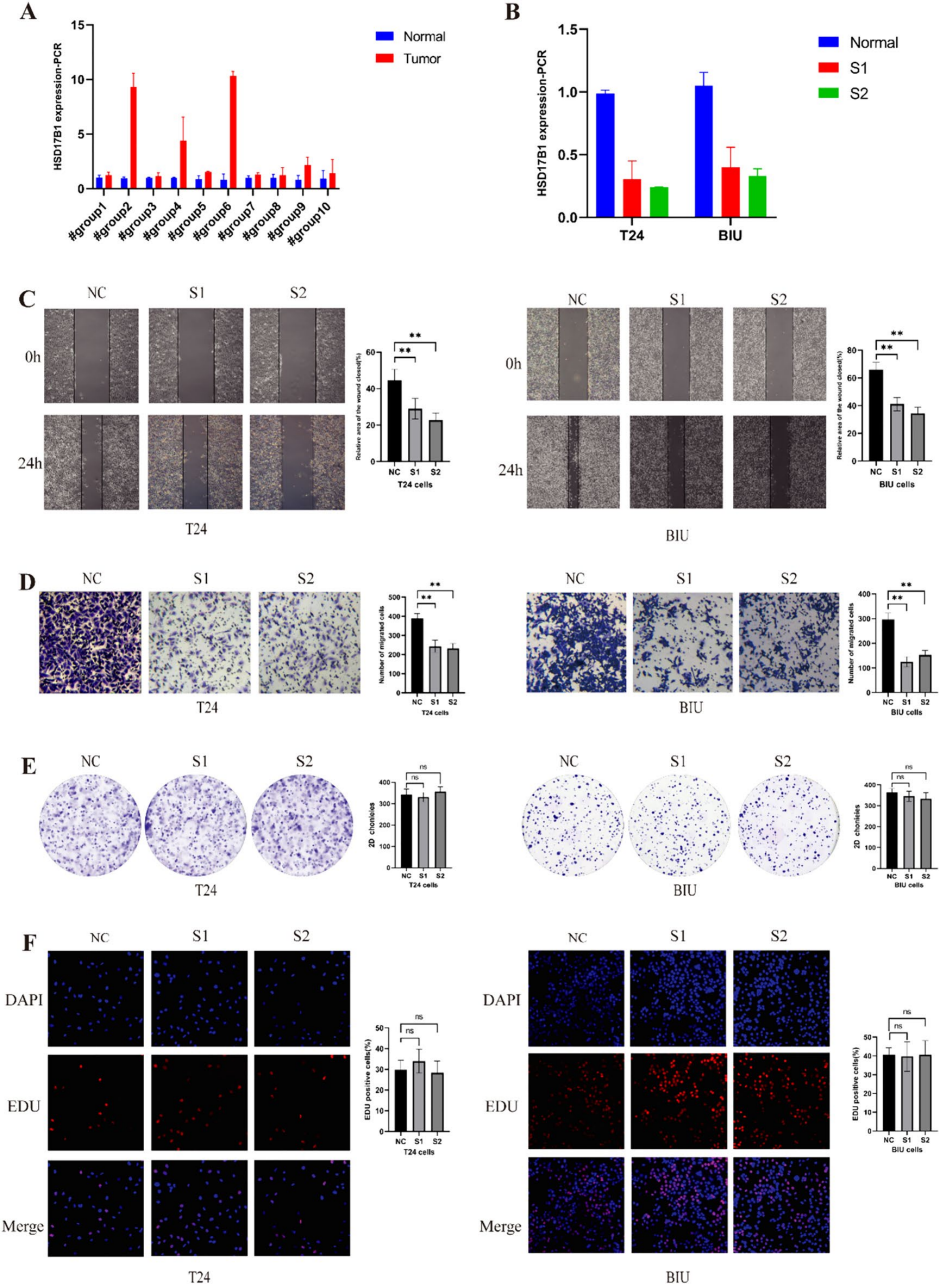
Functional verification of knockdown HSD17B1. HSD17B1 expression in bladder cancer tissues was detected using qPCR, and the interference efficiency of small interfering RNA (siRNA) was also validated (**A**, **B**). Effect of HSD17B1 knockdown on the migration of bladder cancer cells (**C**, **D**). Knockdown of HSD17B1 was followed by colony (**E**) and EdU (**F**) experiments in T24 and BIU cells

We conducted PCR analysis on BLCA patient tissues, confirming its pronounced overexpression in tumor tissues (Fig. 6A). To verify successful transfection of S1 and S2 in T24 and BIU cells, PCR assessments were performed (Fig. 6B). Results from Transwell and wound-healing assays compellingly demonstrate a significant reduction in the migratory capability of BLCA cells, directly attributed to suppressed HSD17B1 expression (Fig. 6C, D). Interestingly, the knockdown of HSD17B1 did not significantly impact the proliferation of T24 and BIU cells, as evidenced by both colony formation and EdU experiments (Fig. 6E, F). In summary, these findings unequivocally highlight the pivotal role of HSD17B1 in modulating the migratory behavior of BLCA cells.

## Discussion

BLCA is a prevalent malignancy characterized by notable progression, significantly impacting patients’ quality of life [22]. The imperative for practical prognostic tools to steer treatment decisions and facilitate outcomes in BLCA patients cannot be overstated. Over time, various gene predictive models associated with BLCA have emerged, spanning those linked to basement inflammation [23], ferroptosis-related mechanisms [24], telomere maintenance [25], and dendritic cell activity [26]. These models have collectively enriched our understanding of BLCA and its therapeutic landscape. Despite these advancements, exploring propionate metabolism-related biomarkers in BLCA remains relatively uncharted territory. Notably, while predictive models related to propionate metabolism have been established for hepatocellular carcinoma [27], their application in BLCA has been sparse. Our study pioneers a novel initiative to address this gap by introducing a predictive model explicitly tailored to propionate metabolism-related risks in BLCA. Through this endeavor, we delineate essential biomarkers intricately linked to propionate metabolism, paving the way for enhanced prognostic assessments and personalized treatment strategies in BLCA patients.

In this study, we utilized an integrated approach combining both differential expression analysis and univariate Cox regression analysis to identify PMRGs that are closely linked to prognosis in BLCA. Subsequently, we stratified BLCA patients into two distinct subgroups, denoted as Cluster A and Cluster B, with Cluster A exhibiting a notably higher survival rate compared to Cluster B. Notably, nearly all identified PMRGs displayed significantly elevated expression levels in Cluster B. Interestingly, our investigation into immune cell infiltration using ssGSEA revealed significantly elevated levels of T cells and macrophages within Cluster B compared to Cluster A. Among these, CD8+ T cells are pivotal in immune surveillance against infections and cancer, while macrophages, the predominant myeloid cell type in cancer, are believed to exert protumor functions predominantly [28].

In this investigation, we conducted an extensive analysis of 812 PMRGs. This meticulous process involved carefully selecting genes exhibiting distinct expression patterns and significant prognostic relevance, culminating in developing a comprehensive risk signature. The finalized set of PMRGs, comprising SCD, GLP1R, MMP9, HSD17B1, RUNX2, and DMD, was the foundation for constructing the risk signature. Notably, SCD emerged as a pivotal enzyme in lipid metabolism, with its expression intricately linked to malignant transformation, tumor proliferation, and OS [29–31]. Furthermore, in a BLCA xenograft model, the inhibition of SCD resulted in substantial suppression of tumor progression, underscoring its potential as a therapeutic target in BLCA management [32]. The GLP1R gene encodes a seven-transmembrane protein, acting as a receptor for GLP1 [33]. According to Toru Shigeoka et al., upregulation of GLP1R may inhibit prostate carcinoma growth by restraining cell cycle progression [34]. Elevated MMP-9 levels are commonly observed in cancer tissues compared to normal adjacent tissues, promoting cancer cell migration and invasion [35, 36]. Studies suggest increased MMP-9 expression in mouse bladder tissues exposed to MC-LR may raise BLCA risk [37]. RUNX2, a member of the RUNX family, regulates crucial developmental processes, including differentiation, apoptosis, proliferation, and cell lineage specification [38]. Initially identified for its role in osteogenesis, RUNX2’s oncogenic functions have been linked to osteosarcoma progression [39]. In BLCA, downregulation of RUNX2 inhibits cell growth, promotes apoptosis, and reduces migration and invasion. These findings suggest that RUNX2 may serve as a prognostic biomarker and therapeutic target in BLCA [40]. The DMD gene, responsible for encoding the DMD protein, is notably abundant in humans [41]. This protein comprises two subunits, *α* and *β*, both of which have been associated with involvement in tumorigenesis [42]. HSD17B1 plays a critical role in various cancers. Research suggests it regulates active estrogen biosynthesis, promoting breast cancer cell proliferation [43]. In non-small cell lung cancer, increased HSD17B1 expression facilitates cancer progression [44]. Clinical studies indicate high HSD17B1 expression is associated with advanced pathologic *N* stage in BLCA, suggesting its potential as a prognostic biomarker [45]. As studies increasingly highlight the significance of HSD17B1 in various tumors, its exploration in BLCA remains limited. Consequently, we aimed to elucidate the specific role of HSD17B1 in BLCA. Our findings reveal upregulated mRNA expression of HSD17B1 in BLCA tissues, with knockdown experiments demonstrating a notable inhibition of BLCA cell migration and invasion.

Furthermore, we developed a nomogram that combines the risk score associated with propionate metabolism and additional clinical parameters, demonstrating excellent performance in prognostic predictions for BLCA patients. This novel risk signature offers an innovative approach to forecasting the survival outcomes of BLCA patients. Calibration curves across the entire dataset are closely aligned with the 45° angle, confirming the robust performance of the nomogram.

The TME plays a pivotal role in driving the proliferation and progression of cancer cells [46]. Our investigation revealed a significant disparity in the immune cell infiltration pattern within the TME, distinguishing between high-risk and low-risk BLCA patients based on the calculated risk score. Specifically, the high-risk group exhibited a notable increase in the abundance of CD4 memory resting T cells, follicular helper T cells, and macrophages compared to the low-risk group. It is evident that the gut microbiota and short-chain fatty acids such as propionate play crucial roles in shaping the generation of both effector and regulatory T cells through epigenetic and metabolic mechanisms [47]. Dysbiosis of the gut microbiota and subsequent propionate production have promoted cancer progression by inducing autophagy in cancer cells and M2 polarization in macrophages [48]. Our investigation identified significant disparities in the TME between high-risk and low-risk BLCA patients, as determined by the risk score. Previous studies have highlighted the predictive role of TME in immunotherapy outcomes, particularly in non-muscle-invasive BLCA [49]. Our findings underscore the importance of TME profiling in BLCA prognosis and treatment decision-making. Future research should focus on elucidating the intricate interplay between TME characteristics, PMRGs, and immunotherapy to optimize patient outcomes in BLCA. To enhance clinical outcomes, we analyzed drug sensitivity to identify different responses between high and low-risk groups. Our results suggest that high-risk individuals may respond well to conventional chemotherapy drugs like cyclophosphamide but may be resistant to leflunomide and oxaliplatin. This highlights the potential for combination chemotherapy to benefit high-risk BLCA patients, enabling more precise and personalized treatment approaches.

However, our study is not without limitations. Firstly, its reliance on public databases means the absence of clinical cohorts, hindering direct clinical validation of the model’s reliability. Secondly, while we observed the impact of HSD17B1 on BLCA cell proliferation and migration, we did not delve deeper into its regulatory mechanisms. Additionally, the retrospective and indirect nature of predicting immune therapy responses underscores the necessity for prospective trials involving larger patient cohorts to enhance the reliability of the scoring system.

## Conclusion

Our study highlights the importance of propionate metabolism in BLCA and introduces a reliable predictive model for predicting patient outcomes and guiding immunotherapy and drug selection. Additionally, we identify HSD17B1 as a promising therapeutic target for BLCA treatment. These findings have meaningful implications and could improve BLCA patients’ treatment outcomes.

### Supplementary Information

Supplementary Material 1: Fig. S1. Overview of study design.

Supplementary Material 2: Fig. S2. Establish risk model and nomogram for predicting survival probability in bladder cancer patients.The volcano plot indicates PMRGs.Consensus matrixes were obtained for *k* = 3, 4, and 5. When *k* = 2, the CDF curve has the lowest slope.PCA, tSNE, and UAMP identified two subtypes based on the expression of PMRGs.Sankey diagram of the interrelationship between two subtypes and high and low risks.The plot of CIR after risk stratification of the nomogram.

Supplementary Material 3: Fig. S3. Clinical pathological characteristics and immune cell infiltration in risk grouping.Correlation analysis between risk scores and clinicopathological features.The infiltrating levels of different immune cells in the high and low-risk groups.

Supplementary Material 4: Fig. S4.Patients’ response to nine common chemotherapeutic drugs in the low- and high-risk groups.

Supplementary Material 5: Fig. S5. Single-cell analysis and validation of the HSD17B1 from external databases.The cell type of GSE145281_aPDL1 dataset.The principal distributions of HSD17B1 on cell types in the GSE145281_aPDL1 dataset.Expression of HSD17B1 in BLCA patients in the TISCH database.Survival analysis for the subgroup classified by HSD17B1 mRNA expression.Expression of HSD17B1 in pan-cancer patients in the TISCH database.The expression of HSD17B1 between normal and tumor was analyzed by immunohistochemistryfrom the Human Protein Atlasdataset.

Supplementary Material 6.

## Data Availability

Data analyzed by the present study can be gained from TCGA (https://portal.gdc.cancer.gov/) databases (under accession: TCGA-BLCA) and GEO (http://www.ncbi.nlm.nih.gov/geo/) (under accession: GSE13507).
