# Supplementary figures and images for: Development of a propionate metabolism-related gene-based molecular subtypes and scoring system for predicting prognosis in bladder cancer
Source: Eur J Med Res. 2024 Jul 29;29:393. doi: 10.1186/s40001-024-01982-6 (PMC11285334; doi:10.1186/s40001-024-01982-6)

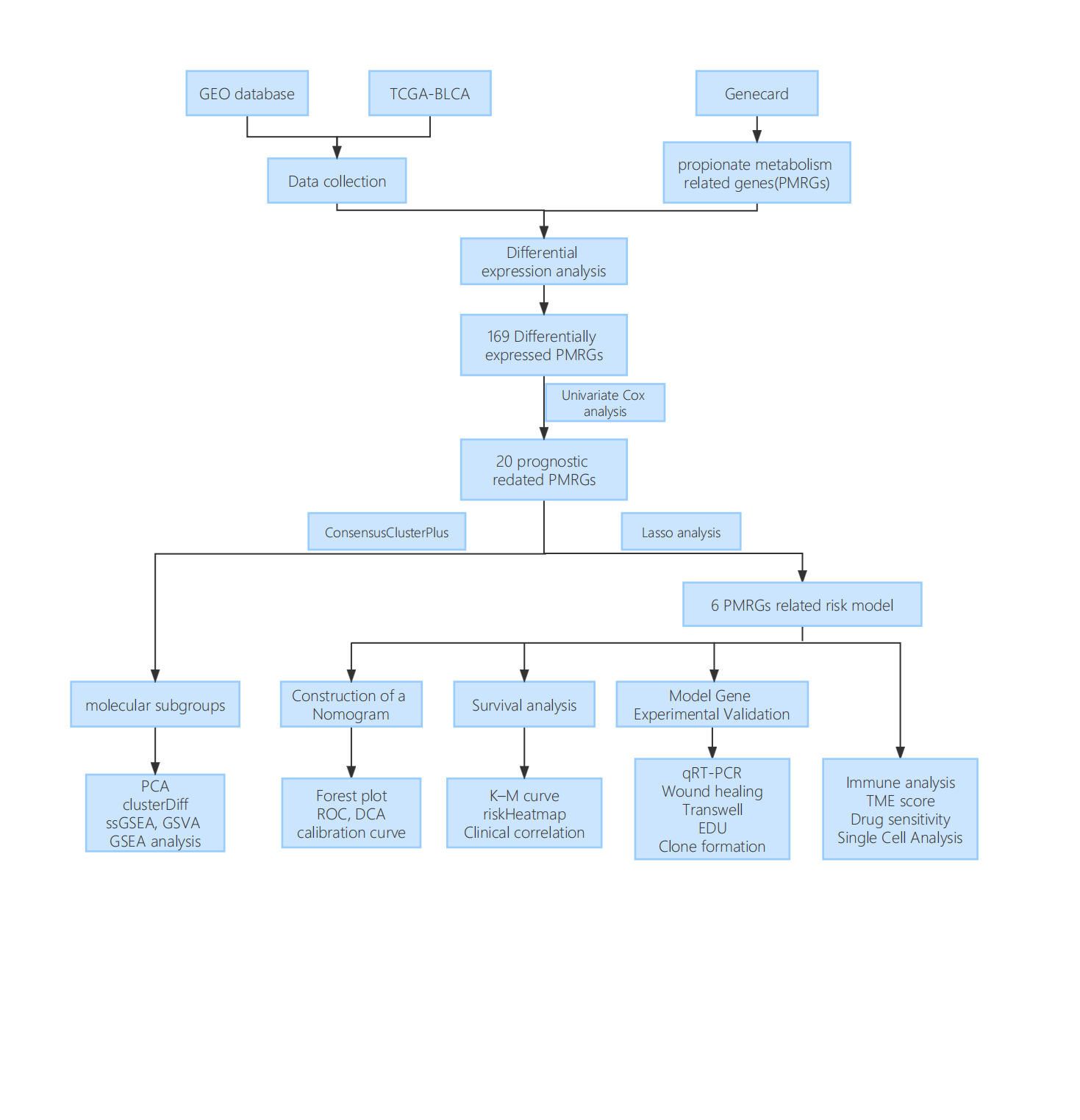

Supplement: Supplementary file 1 — Supplementary Material 1: Fig. S1. Overview of study design. [file 40001_2024_1982_MOESM1_ESM.tif]

**A**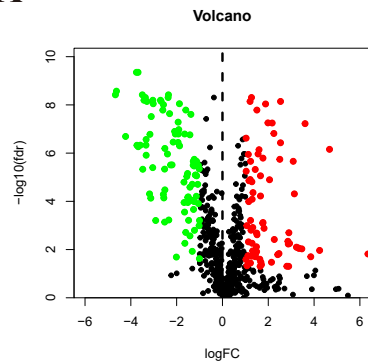**B**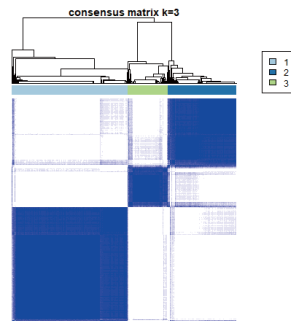**C**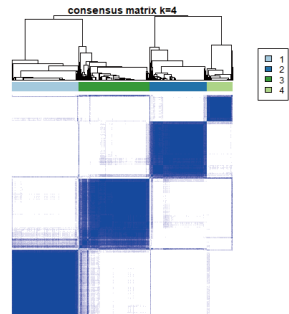**D**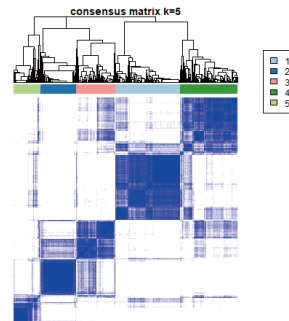**E**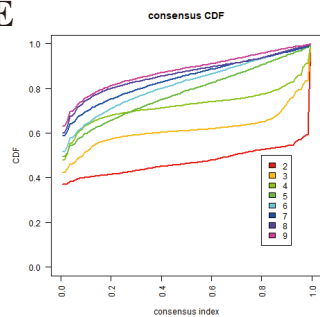**F**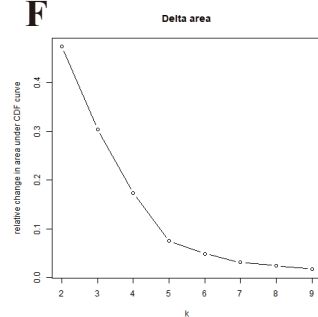**G**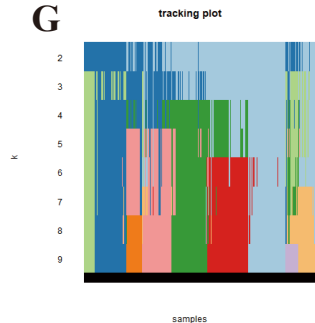**H**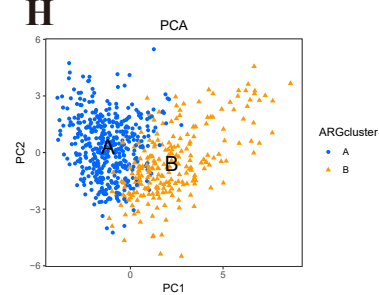**I**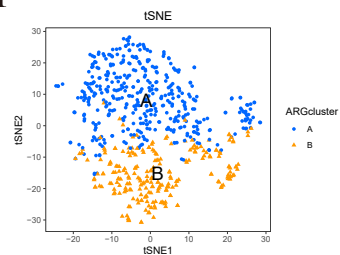**J**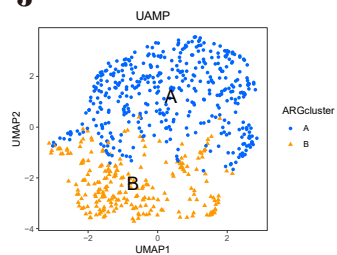**K**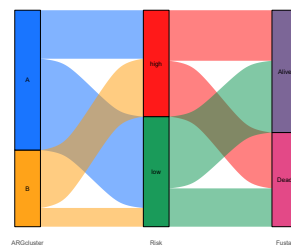**L**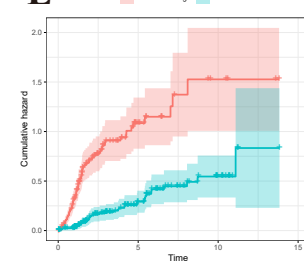

Supplement: Supplementary file 2 — Supplementary Material 2: Fig. S2. Establish risk model and nomogram for predicting survival probability in bladder cancer patients.The volcano plot indicates PMRGs.Consensus matrixes were obtained for k = 3, 4, and 5. When k = 2, the CDF curve has the lowest slope.PCA, tSNE, and UAMP identified two subtypes based on the expression of PMRGs.Sankey diagram of the interrelationship between two subtypes and high and low risks.The plot of CIR after risk stratification of the nomogram. [file 40001_2024_1982_MOESM2_ESM.pdf]

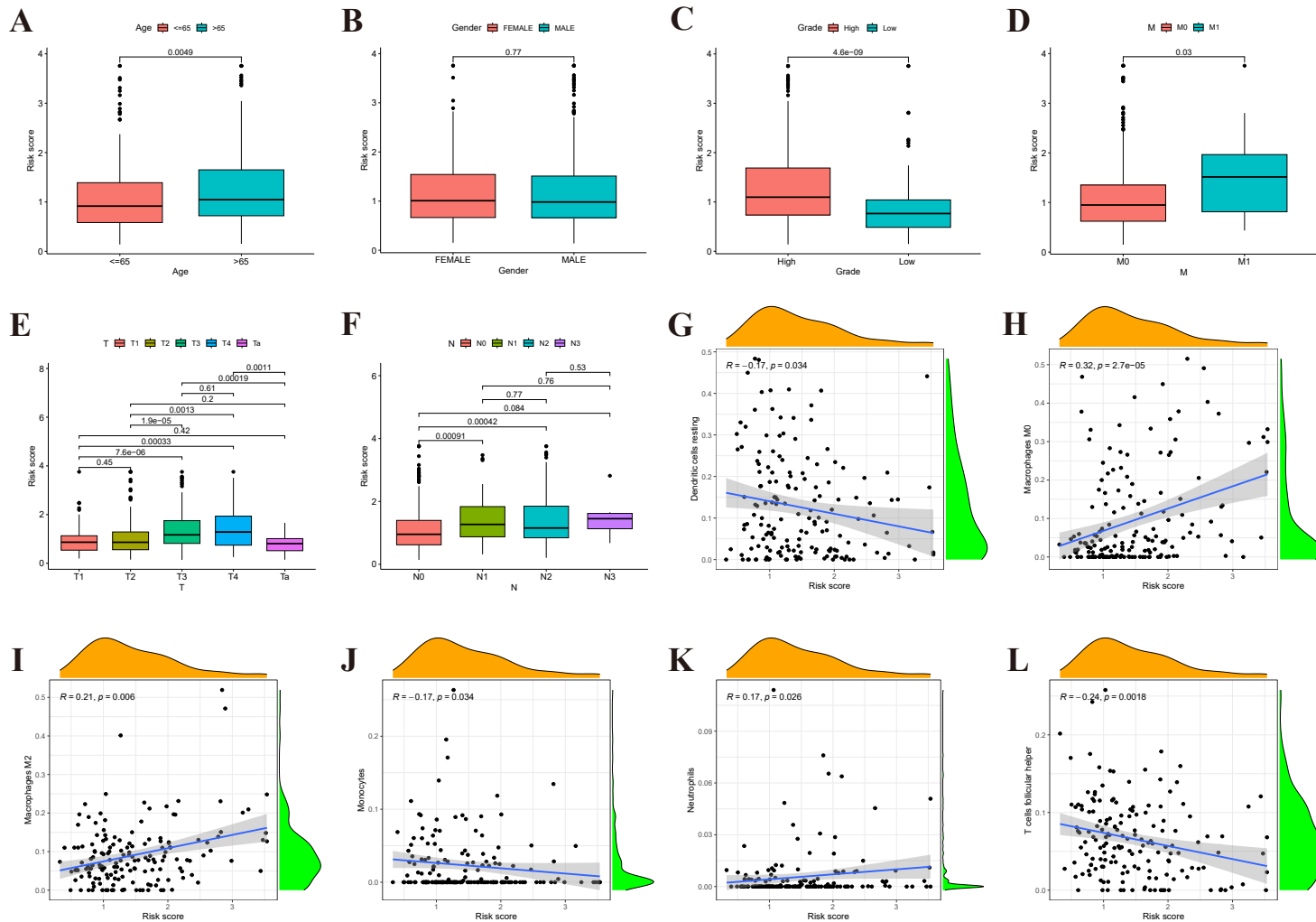

Supplement: Supplementary file 3 — Supplementary Material 3: Fig. S3. Clinical pathological characteristics and immune cell infiltration in risk grouping.Correlation analysis between risk scores and clinicopathological features.The infiltrating levels of different immune cells in the high and low-risk groups. [file 40001_2024_1982_MOESM3_ESM.pdf]

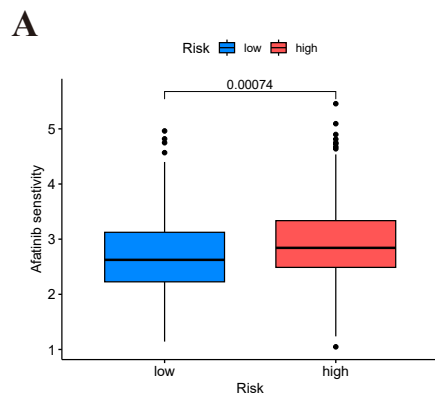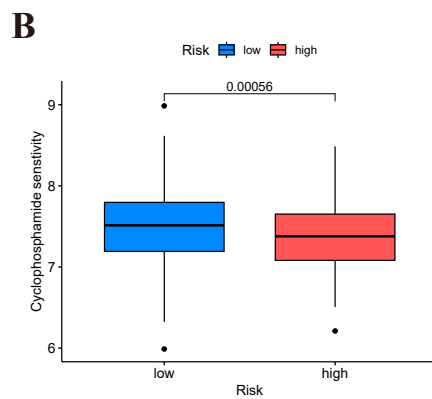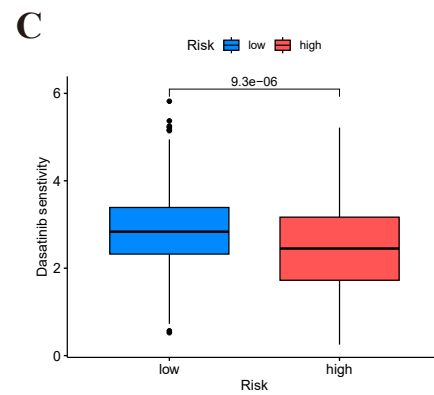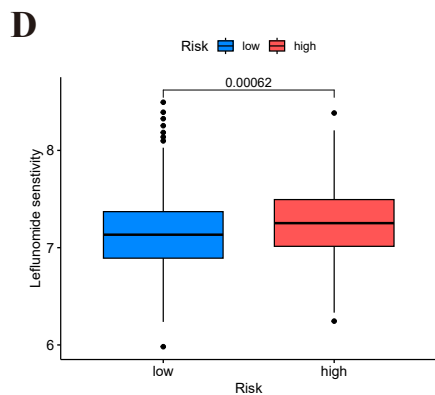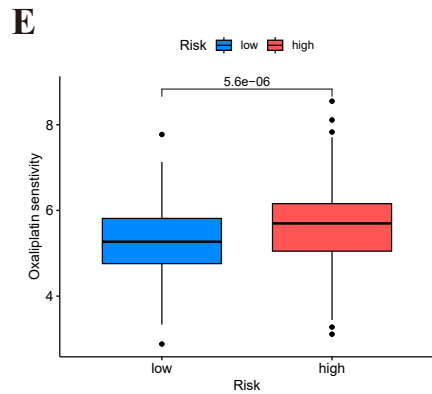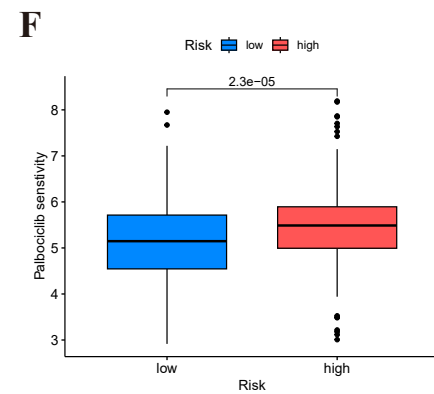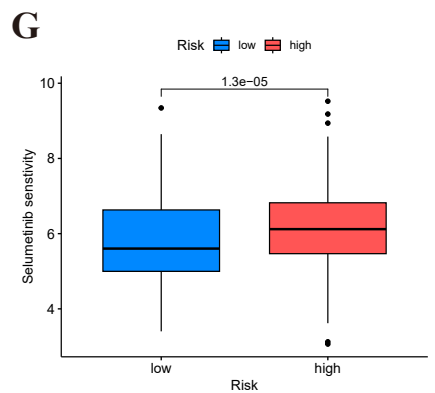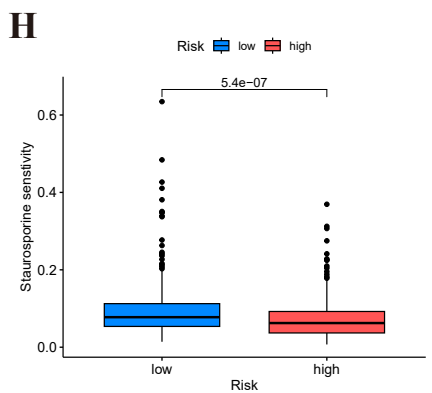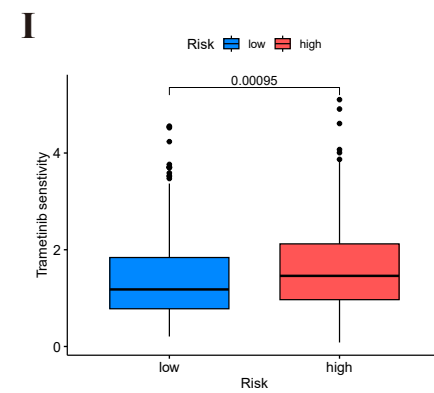

Supplement: Supplementary file 4 — Supplementary Material 4: Fig. S4.Patients’ response to nine common chemotherapeutic drugs in the low- and high-risk groups. [file 40001_2024_1982_MOESM4_ESM.pdf]
