# Supplementary material for: Development of a propionate metabolism-related gene-based molecular subtypes and scoring system for predicting prognosis in bladder cancer
Source: Eur J Med Res. 2024 Jul 29;29:393. doi: 10.1186/s40001-024-01982-6 (PMC11285334; doi:10.1186/s40001-024-01982-6)
Supplement: Supplementary file 6 — Supplementary Material 6. [file 40001_2024_1982_MOESM6_ESM.pdf]

Supplementary Table 1 812 PMRGs obtained from the GeneCards database.

Gene Symbol	Description	Category	Gifts	GC Id	Relevance score	GeneCards Link
PCCB	Propionyl-CoA Carboxylase Subunit Beta	Protein Coding	53	GC03P136250	61.11257935	https://www.genecards.org/cgi-bin/carddisp.pl?gene=PCCB
PCCA	Propionyl-CoA Carboxylase Subunit Alpha	Protein Coding	53	GC13P100089	56.12726974	https://www.genecards.org/cgi-bin/carddisp.pl?gene=PCCA
MMACHC	Metabolism Of Cobalamin Associated C	Protein Coding	50	GC01P045500	47.25754929	https://www.genecards.org/cgi-bin/carddisp.pl?gene=MMACHC
MTR	5-Methyltetrahydrofolate-Homocysteine Methyltransferase	Protein Coding	54	GC01P236795	39.33602524	https://www.genecards.org/cgi-bin/carddisp.pl?gene=MTR
CYP2D6	Cytochrome P450 Family 2 Subfamily D Member 6	Protein Coding	53	GC22M042126	39.22632217	https://www.genecards.org/cgi-bin/carddisp.pl?gene=CYP2D6
MMADHC	Metabolism Of Cobalamin Associated D	Protein Coding	49	GC02M149569	35.76113892	https://www.genecards.org/cgi-bin/carddisp.pl?gene=MMADHC
CYP3A4	Cytochrome P450 Family 3 Subfamily A Member 4	Protein Coding	56	GC07M099759	35.6474762	https://www.genecards.org/cgi-bin/carddisp.pl?gene=CYP3A4
CYP2C19	Cytochrome P450 Family 2 Subfamily C Member 19	Protein Coding	52	GC10P094762	34.64389801	https://www.genecards.org/cgi-bin/carddisp.pl?gene=CYP2C19
INS	Insulin	Protein Coding	55	GC11M002159	33.82258224	https://www.genecards.org/cgi-bin/carddisp.pl?gene=INS
MMUT	Methylmalonyl-CoA Mutase	Protein Coding	52	GC06M049430	31.02311325	https://www.genecards.org/cgi-bin/carddisp.pl?gene=MMUT
ABCA3	ATP Binding Cassette Subfamily A Member 3	Protein Coding	55	GC16M002275	30.44045639	https://www.genecards.org/cgi-bin/carddisp.pl?gene=ABCA3
MMAA	Metabolism Of Cobalamin Associated A	Protein Coding	47	GC04P145610	30.1062355	https://www.genecards.org/cgi-bin/carddisp.pl?gene=MMAA
UQCRFS1	Ubiquinol-Cytochrome C Reductase, Rieske Iron-Sulfur Polypeptide 1	Protein Coding	53	GC19M029205	30.09693146	https://www.genecards.org/cgi-bin/carddisp.pl?gene=UQCRFS1
ACADM	Acyl-CoA Dehydrogenase Medium Chain	Protein Coding	52	GC01P075724	29.86544609	https://www.genecards.org/cgi-bin/carddisp.pl?ge

						ne=ACADM
PPARG	Peroxisome Proliferator Activated Receptor Gamma	Protein Coding	60	GC03P012287	29.00304985	https://www.genecards.org/cgi-bin/carddisp.pl?gene=PPARG
MTRR	5-Methyltetrahydrofolate-Homocysteine Methyltransferase Reductase	Protein Coding	50	GC05P007851	28.36356544	https://www.genecards.org/cgi-bin/carddisp.pl?gene=MTRR
LEP	Leptin	Protein Coding	53	GC07P128241	28.21476173	https://www.genecards.org/cgi-bin/carddisp.pl?gene=LEP
MMAB	Metabolism Of Cobalamin Associated B	Protein Coding	51	GC12M109553	28.03660583	https://www.genecards.org/cgi-bin/carddisp.pl?gene=MMAB
OTC	Ornithine Transcarbamylase	Protein Coding	52	GC0XP038353	26.92333984	https://www.genecards.org/cgi-bin/carddisp.pl?gene=OTC
CYP2C9	Cytochrome P450 Family 2 Subfamily C Member 9	Protein Coding	53	GC10P094938	25.8538208	https://www.genecards.org/cgi-bin/carddisp.pl?gene=CYP2C9
LDLR	Low Density Lipoprotein Receptor	Protein Coding	58	GC19P011091	25.81306458	https://www.genecards.org/cgi-bin/carddisp.pl?gene=LDLR
ALB	Albumin	Protein Coding	56	GC04P073397	25.16794777	https://www.genecards.org/cgi-bin/carddisp.pl?gene=ALB
CYP2C8	Cytochrome P450 Family 2 Subfamily C Member 8	Protein Coding	52	GC10M095038	25.04213715	https://www.genecards.org/cgi-bin/carddisp.pl?gene=CYP2C8
APOE	Apolipoprotein E	Protein Coding	57	GC19P073055	24.89061928	https://www.genecards.org/cgi-bin/carddisp.pl?gene=APOE
ADIPOQ	Adiponectin, C1Q And Collagen Domain Containing	Protein Coding	52	GC03P186842	24.75322914	https://www.genecards.org/cgi-bin/carddisp.pl?gene=ADIPOQ
CYP1A2	Cytochrome P450 Family 1 Subfamily A Member 2	Protein Coding	52	GC15P074748	24.42164421	https://www.genecards.org/cgi-bin/carddisp.pl?gene=CYP1A2
IL6	Interleukin 6	Protein Coding	58	GC07P022725	24.29815102	https://www.genecards.org/cgi-bin/carddisp.pl?gene=IL6
LIPC	Lipase C, Hepatic Type	Protein Coding	52	GC15P058410	24.07993889	https://www.genecards.org/cgi-bin/carddisp.pl?gene=LIPC
LPL	Lipoprotein Lipase	Protein Coding	56	GC08P019901	23.63115692	https://www.genecards.org/cgi-bin/carddisp.pl?ge

						ne=LPL
APOB	Apolipoprotein B	Protein Coding	53	GC02M020956	23.32738113	https://www.genecards.org/cgi-bin/carddisp.pl?gene=APOB
UGT1A1	UDP Glucuronosyltransferase Family 1 Member A1	Protein Coding	57	GC02P233760	23.1049614	https://www.genecards.org/cgi-bin/carddisp.pl?gene=UGT1A1
CYP1A1	Cytochrome P450 Family 1 Subfamily A Member 1	Protein Coding	55	GC15M074719	23.08771324	https://www.genecards.org/cgi-bin/carddisp.pl?gene=CYP1A1
GCDH	Glutaryl-CoA Dehydrogenase	Protein Coding	52	GC19P012891	23.0321064	https://www.genecards.org/cgi-bin/carddisp.pl?gene=GCDH
PPARA	Peroxisome Proliferator Activated Receptor Alpha	Protein Coding	51	GC22P046150	22.84316635	https://www.genecards.org/cgi-bin/carddisp.pl?gene=PPARA
CYP2B6	Cytochrome P450 Family 2 Subfamily B Member 6	Protein Coding	52	GC19P040991	22.70144463	https://www.genecards.org/cgi-bin/carddisp.pl?gene=CYP2B6
IGF1	Insulin Like Growth Factor 1	Protein Coding	55	GC12M102395	22.66413879	https://www.genecards.org/cgi-bin/carddisp.pl?gene=IGF1
CYP2A6	Cytochrome P450 Family 2 Subfamily A Member 6	Protein Coding	53	GC19M040843	22.56680679	https://www.genecards.org/cgi-bin/carddisp.pl?gene=CYP2A6
TPMT	Thiopurine S-Methyltransferase	Protein Coding	53	GC06M018128	22.38929176	https://www.genecards.org/cgi-bin/carddisp.pl?gene=TPMT
CBS	Cystathionine Beta-Synthase	Protein Coding	56	GC21M043053	22.33657455	https://www.genecards.org/cgi-bin/carddisp.pl?gene=CBS
TNF	Tumor Necrosis Factor	Protein Coding	59	GC06P092154	21.97315598	https://www.genecards.org/cgi-bin/carddisp.pl?gene=TNF
HMGCL	3-Hydroxy-3-Methylglutaryl-CoA Lyase	Protein Coding	51	GC01M023801	21.91726685	https://www.genecards.org/cgi-bin/carddisp.pl?gene=HMGCL
CYP2E1	Cytochrome P450 Family 2 Subfamily E Member 1	Protein Coding	52	GC10P133520	21.77449989	https://www.genecards.org/cgi-bin/carddisp.pl?gene=CYP2E1
CSF2RA	Colony Stimulating Factor 2 Receptor Subunit Alpha	Protein Coding	52	GC0XP001845	21.42407227	https://www.genecards.org/cgi-bin/carddisp.pl?gene=CSF2RA
PAH	Phenylalanine Hydroxylase	Protein Coding	55	GC12M102836	21.21108818	https://www.genecards.org/cgi-bin/carddisp.pl?ge

						ne=PAH
CYP3A5	Cytochrome P450 Family 3 Subfamily A Member 5	Protein Coding	51	GC07M099648	21.14499283	https://www.genecards.org/cgi-bin/carddisp.pl?gene=CYP3A5
BTD	Biotinidase	Protein Coding	52	GC03P017386	21.06881714	https://www.genecards.org/cgi-bin/carddisp.pl?gene=BTD
GHRL	Ghrelin And Obestatin Prepropeptide	Protein Coding	49	GC03M010285	20.99878693	https://www.genecards.org/cgi-bin/carddisp.pl?gene=GHRL
SFTPB	Surfactant Protein B	Protein Coding	51	GC02M085657	20.57275009	https://www.genecards.org/cgi-bin/carddisp.pl?gene=SFTPB
ACADVL	Acyl-CoA Dehydrogenase Very Long Chain	Protein Coding	51	GC17P007219	20.4634285	https://www.genecards.org/cgi-bin/carddisp.pl?gene=ACADVL
CPT2	Carnitine Palmitoyltransferase 2	Protein Coding	56	GC01P053196	20.11322403	https://www.genecards.org/cgi-bin/carddisp.pl?gene=CPT2
HADHA	Hydroxyacyl-CoA Dehydrogenase Trifunctional Multienzyme Complex Subunit Alpha	Protein Coding	52	GC02M026190	20.02347183	https://www.genecards.org/cgi-bin/carddisp.pl?gene=HADHA
ETFDH	Electron Transfer Flavoprotein Dehydrogenase	Protein Coding	52	GC04P158672	19.88984299	https://www.genecards.org/cgi-bin/carddisp.pl?gene=ETFDH
ACADS	Acyl-CoA Dehydrogenase Short Chain	Protein Coding	52	GC12P126959	19.86980438	https://www.genecards.org/cgi-bin/carddisp.pl?gene=ACADS
PC	Pyruvate Carboxylase	Protein Coding	53	GC11M066848	19.85100937	https://www.genecards.org/cgi-bin/carddisp.pl?gene=PC
ADSL	Adenylosuccinate Lyase	Protein Coding	54	GC22P040346	19.84980774	https://www.genecards.org/cgi-bin/carddisp.pl?gene=ADSL
CRP	C-Reactive Protein	Protein Coding	53	GC01M159729	19.67492294	https://www.genecards.org/cgi-bin/carddisp.pl?gene=CRP
GGT1	Gamma-Glutamyltransferase 1	Protein Coding	56	GC22P024583	19.48277092	https://www.genecards.org/cgi-bin/carddisp.pl?gene=GGT1
NR3C1	Nuclear Receptor Subfamily 3 Group C Member 1	Protein Coding	56	GC05M143277	19.25010681	https://www.genecards.org/cgi-bin/carddisp.pl?gene=NR3C1
BGLAP	Bone Gamma-Carboxyglutamate Protein	Protein Coding	46	GC01P156242	18.94005585	https://www.genecards.org/cgi-bin/carddisp.pl?ge

						ne=BGLAP
INSR	Insulin Receptor	Protein Coding	60	GC19M007112	18.79404449	https://www.genecards.org/cgi-bin/carddisp.pl?gene=INSR
CPT1A	Carnitine Palmitoyltransferase 1A	Protein Coding	55	GC11M068754	18.33993149	https://www.genecards.org/cgi-bin/carddisp.pl?gene=CPT1A
APOA1	Apolipoprotein A1	Protein Coding	57	GC11M116835	18.12685776	https://www.genecards.org/cgi-bin/carddisp.pl?gene=APOA1
BCKDHB	Branched Chain Keto Acid Dehydrogenase E1 Subunit Beta	Protein Coding	50	GC06P080106	18.03951263	https://www.genecards.org/cgi-bin/carddisp.pl?gene=BCKDHB
AKT1	AKT Serine/Threonine Kinase 1	Protein Coding	61	GC14M104769	17.96221733	https://www.genecards.org/cgi-bin/carddisp.pl?gene=AKT1
TP53	Tumor Protein P53	Protein Coding	60	GC17M007661	17.83651924	https://www.genecards.org/cgi-bin/carddisp.pl?gene=TP53
CES1	Carboxylesterase 1	Protein Coding	55	GC16M055836	17.61368179	https://www.genecards.org/cgi-bin/carddisp.pl?gene=CES1
HNF4A	Hepatocyte Nuclear Factor 4 Alpha	Protein Coding	56	GC20P044355	17.52772713	https://www.genecards.org/cgi-bin/carddisp.pl?gene=HNF4A
ABCD4	ATP Binding Cassette Subfamily D Member 4	Protein Coding	50	GC14M074285	17.33675766	https://www.genecards.org/cgi-bin/carddisp.pl?gene=ABCD4
UGT1A6	UDP Glucuronosyltransferase Family 1 Member A6	Protein Coding	48	GC02P233691	17.17436981	https://www.genecards.org/cgi-bin/carddisp.pl?gene=UGT1A6
ASS1	Argininosuccinate Synthase 1	Protein Coding	56	GC09P130444	17.09732819	https://www.genecards.org/cgi-bin/carddisp.pl?gene=ASS1
MCEE	Methylmalonyl-CoA Epimerase	Protein Coding	47	GC02M071110	17.0945816	https://www.genecards.org/cgi-bin/carddisp.pl?gene=MCEE
HLCS	Holocarboxylase Synthetase	Protein Coding	48	GC21M036750	17.08519173	https://www.genecards.org/cgi-bin/carddisp.pl?gene=HLCS
POMC	Proopiomelanocortin	Protein Coding	55	GC02M025160	16.98484993	https://www.genecards.org/cgi-bin/carddisp.pl?gene=POMC
ACE	Angiotensin I Converting Enzyme	Protein Coding	59	GC17P063477	16.94721031	https://www.genecards.org/cgi-bin/carddisp.pl?ge

						ne=ACE
IVD	Isovaleryl-CoA Dehydrogenase	Protein Coding	51	GC15P040405	16.93725967	https://www.genecards.org/cgi-bin/carddisp.pl?gene=IVD
HIBCH	3-Hydroxyisobutyryl-CoA Hydrolase	Protein Coding	50	GC02M190189	16.7645874	https://www.genecards.org/cgi-bin/carddisp.pl?gene=HIBCH
NAGS	N-Acetylglutamate Synthase	Protein Coding	47	GC17P044004	16.73843575	https://www.genecards.org/cgi-bin/carddisp.pl?gene=NAGS
LMBRD1	LMBR1 Domain Containing 1	Protein Coding	47	GC06M072251	16.69300079	https://www.genecards.org/cgi-bin/carddisp.pl?gene=LMBRD1
SLC2A1	Solute Carrier Family 2 Member 1	Protein Coding	60	GC01M042925	16.59392548	https://www.genecards.org/cgi-bin/carddisp.pl?gene=SLC2A1
CETP	Cholesteryl Ester Transfer Protein	Protein Coding	53	GC16P056961	16.59146118	https://www.genecards.org/cgi-bin/carddisp.pl?gene=CETP
CPS1	Carbamoyl-Phosphate Synthase 1	Protein Coding	55	GC02P210477	16.44989777	https://www.genecards.org/cgi-bin/carddisp.pl?gene=CPS1
CYP1B1	Cytochrome P450 Family 1 Subfamily B Member 1	Protein Coding	54	GC02M038066	16.42423248	https://www.genecards.org/cgi-bin/carddisp.pl?gene=CYP1B1
SLC22A5	Solute Carrier Family 22 Member 5	Protein Coding	53	GC05P132369	16.36189461	https://www.genecards.org/cgi-bin/carddisp.pl?gene=SLC22A5
GPT	Glutamic--Pyruvic Transaminase	Protein Coding	48	GC08P144502	16.35798264	https://www.genecards.org/cgi-bin/carddisp.pl?gene=GPT
GLDC	Glycine Decarboxylase	Protein Coding	53	GC09M006522	16.15870285	https://www.genecards.org/cgi-bin/carddisp.pl?gene=GLDC
IL1B	Interleukin 1 Beta	Protein Coding	54	GC02M112829	16.14551735	https://www.genecards.org/cgi-bin/carddisp.pl?gene=IL1B
VDR	Vitamin D Receptor	Protein Coding	56	GC12M047841	16.03931808	https://www.genecards.org/cgi-bin/carddisp.pl?gene=VDR
FH	Fumarate Hydratase	Protein Coding	53	GC01M241499	15.98605347	https://www.genecards.org/cgi-bin/carddisp.pl?gene=FH
PRODH	Proline Dehydrogenase 1	Protein Coding	52	GC22M018912	15.97478485	https://www.genecards.org/cgi-bin/carddisp.pl?ge

						ne=PRODH
CYP7A1	Cytochrome P450 Family 7 Subfamily A Member 1	Protein Coding	49	GC08M058476	15.92755032	https://www.genecards.org/cgi-bin/carddisp.pl?gene=CYP7A1
HSD11B1	Hydroxysteroid 11-Beta Dehydrogenase 1	Protein Coding	58	GC01P209686	15.90985107	https://www.genecards.org/cgi-bin/carddisp.pl?gene=HSD11B1
ENPP1	Ectonucleotide Pyrophosphatase/Phosphodiesterase 1	Protein Coding	56	GC06P131808	15.89481449	https://www.genecards.org/cgi-bin/carddisp.pl?gene=ENPP1
HSD11B2	Hydroxysteroid 11-Beta Dehydrogenase 2	Protein Coding	52	GC16P067433	15.8716507	https://www.genecards.org/cgi-bin/carddisp.pl?gene=HSD11B2
CYP27B1	Cytochrome P450 Family 27 Subfamily B Member 1	Protein Coding	52	GC12M057790	15.85970306	https://www.genecards.org/cgi-bin/carddisp.pl?gene=CYP27B1
XDH	Xanthine Dehydrogenase	Protein Coding	54	GC02M031334	15.85227299	https://www.genecards.org/cgi-bin/carddisp.pl?gene=XDH
RETN	Resistin	Protein Coding	49	GC19P007669	15.76069736	https://www.genecards.org/cgi-bin/carddisp.pl?gene=RETN
ABCB1	ATP Binding Cassette Subfamily B Member 1	Protein Coding	57	GC07M087504	15.75623131	https://www.genecards.org/cgi-bin/carddisp.pl?gene=ABCB1
CA5A	Carbonic Anhydrase 5A	Protein Coding	50	GC16M087882	15.74926186	https://www.genecards.org/cgi-bin/carddisp.pl?gene=CA5A
MAT1A	Methionine Adenosyltransferase 1A	Protein Coding	52	GC10M080271	15.67016792	https://www.genecards.org/cgi-bin/carddisp.pl?gene=MAT1A
LEPR	Leptin Receptor	Protein Coding	57	GC01P065421	15.63416672	https://www.genecards.org/cgi-bin/carddisp.pl?gene=LEPR
IRS1	Insulin Receptor Substrate 1	Protein Coding	53	GC02M226731	15.51399326	https://www.genecards.org/cgi-bin/carddisp.pl?gene=IRS1
UGT1A8	UDP Glucuronosyltransferase Family 1 Member A8	Protein Coding	43	GC02P233618	15.4636898	https://www.genecards.org/cgi-bin/carddisp.pl?gene=UGT1A8
GAMT	Guanidinoacetate N-Methyltransferase	Protein Coding	52	GC19M001397	15.44042587	https://www.genecards.org/cgi-bin/carddisp.pl?gene=GAMT
ABCA1	ATP Binding Cassette Subfamily A Member 1	Protein Coding	55	GC09M104781	15.39727402	https://www.genecards.org/cgi-bin/carddisp.pl?ge

						ne=ABCA1
UGT1A9	UDP Glucuronosyltransferase Family 1 Member A9	Protein Coding	51	GC02P233671	15.35033798	https://www.genecards.org/cgi-bin/carddisp.pl?gene=UGT1A9
GALT	Galactose-1-Phosphate Uridylyltransferase	Protein Coding	53	GC09P048949	15.34730434	https://www.genecards.org/cgi-bin/carddisp.pl?gene=GALT
FGF23	Fibroblast Growth Factor 23	Protein Coding	52	GC12M004368	15.22850227	https://www.genecards.org/cgi-bin/carddisp.pl?gene=FGF23
CYP17A1	Cytochrome P450 Family 17 Subfamily A Member 1	Protein Coding	55	GC10M102830	15.21972752	https://www.genecards.org/cgi-bin/carddisp.pl?gene=CYP17A1
G6PC1	Glucose-6-Phosphatase Catalytic Subunit 1	Protein Coding	51	GC17P063334	15.16344547	https://www.genecards.org/cgi-bin/carddisp.pl?gene=G6PC1
HFE	Homeostatic Iron Regulator	Protein Coding	52	GC06P026087	15.10912323	https://www.genecards.org/cgi-bin/carddisp.pl?gene=HFE
APOC3	Apolipoprotein C3	Protein Coding	52	GC11P116829	15.01816845	https://www.genecards.org/cgi-bin/carddisp.pl?gene=APOC3
FASN	Fatty Acid Synthase	Protein Coding	55	GC17M082078	15.00522327	https://www.genecards.org/cgi-bin/carddisp.pl?gene=FASN
DPYD	Dihydropyrimidine Dehydrogenase	Protein Coding	59	GC01M097015	14.99942017	https://www.genecards.org/cgi-bin/carddisp.pl?gene=DPYD
BCKDHA	Branched Chain Keto Acid Dehydrogenase E1 Subunit Alpha	Protein Coding	51	GC19P072922	14.99351788	https://www.genecards.org/cgi-bin/carddisp.pl?gene=BCKDHA
ADRB2	Adrenoceptor Beta 2	Protein Coding	56	GC05P148825	14.96682453	https://www.genecards.org/cgi-bin/carddisp.pl?gene=ADRB2
ALDH2	Aldehyde Dehydrogenase 2 Family Member	Protein Coding	57	GC12P111766	14.95112228	https://www.genecards.org/cgi-bin/carddisp.pl?gene=ALDH2
NR3C2	Nuclear Receptor Subfamily 3 Group C Member 2	Protein Coding	52	GC04M148078	14.85720921	https://www.genecards.org/cgi-bin/carddisp.pl?gene=NR3C2
SLCO1B1	Solute Carrier Organic Anion Transporter Family Member 1B1	Protein Coding	55	GC12P023907	14.79038334	https://www.genecards.org/cgi-bin/carddisp.pl?gene=SLCO1B1
HCFC1	Host Cell Factor C1	Protein Coding	53	GC0XM153947	14.77022457	https://www.genecards.org/cgi-bin/carddisp.pl?ge

						ne=HCFC1
IL10	Interleukin 10	Protein Coding	55	GC01M206767	14.64819908	https://www.genecards.org/cgi-bin/carddisp.pl?gene=IL10
CASR	Calcium Sensing Receptor	Protein Coding	57	GC03P122183	14.60171509	https://www.genecards.org/cgi-bin/carddisp.pl?gene=CASR
GCG	Glucagon	Protein Coding	48	GC02M162142	14.56726742	https://www.genecards.org/cgi-bin/carddisp.pl?gene=GCG
PCSK9	Proprotein Convertase Subtilisin/Kexin Type 9	Protein Coding	57	GC01P055039	14.51441669	https://www.genecards.org/cgi-bin/carddisp.pl?gene=PCSK9
HSD17B10	Hydroxysteroid 17-Beta Dehydrogenase 10	Protein Coding	53	GC0XM053431	14.5091486	https://www.genecards.org/cgi-bin/carddisp.pl?gene=HSD17B10
SHBG	Sex Hormone Binding Globulin	Protein Coding	48	GC17P007613	14.43100166	https://www.genecards.org/cgi-bin/carddisp.pl?gene=SHBG
SLC2A4	Solute Carrier Family 2 Member 4	Protein Coding	52	GC17P011713	14.34994125	https://www.genecards.org/cgi-bin/carddisp.pl?gene=SLC2A4
CYP19A1	Cytochrome P450 Family 19 Subfamily A Member 1	Protein Coding	55	GC15M051208	14.28239822	https://www.genecards.org/cgi-bin/carddisp.pl?gene=CYP19A1
HPD	4-Hydroxyphenylpyruvate Dioxygenase	Protein Coding	52	GC12M121839	14.13055611	https://www.genecards.org/cgi-bin/carddisp.pl?gene=HPD
HIF1A	Hypoxia Inducible Factor 1 Subunit Alpha	Protein Coding	54	GC14P061695	14.09283161	https://www.genecards.org/cgi-bin/carddisp.pl?gene=HIF1A
VKORC1	Vitamin K Epoxide Reductase Complex Subunit 1	Protein Coding	54	GC16M038547	14.08553123	https://www.genecards.org/cgi-bin/carddisp.pl?gene=VKORC1
AHCY	Adenosylhomocysteinase	Protein Coding	56	GC20M034942	14.07678223	https://www.genecards.org/cgi-bin/carddisp.pl?gene=AHCY
FBP1	Fructose-Bisphosphatase 1	Protein Coding	56	GC09M094603	14.06463623	https://www.genecards.org/cgi-bin/carddisp.pl?gene=FBP1
NOS3	Nitric Oxide Synthase 3	Protein Coding	56	GC07P150990	14.06137276	https://www.genecards.org/cgi-bin/carddisp.pl?gene=NOS3
ALDH5A1	Aldehyde Dehydrogenase 5 Family Member A1	Protein Coding	54	GC06P024494	14.05005932	https://www.genecards.org/cgi-bin/carddisp.pl?ge

						ne=ALDH5A1
FTCD	Formimidoyltransferase Cyclodeaminase	Protein Coding	49	GC21M051374	14.04514503	https://www.genecards.org/cgi-bin/carddisp.pl?gene=FTCD
LDHA	Lactate Dehydrogenase A	Protein Coding	57	GC11P018394	14.0241642	https://www.genecards.org/cgi-bin/carddisp.pl?gene=LDHA
PTH	Parathyroid Hormone	Protein Coding	52	GC11M013492	14.01707935	https://www.genecards.org/cgi-bin/carddisp.pl?gene=PTH
PPARD	Peroxisome Proliferator Activated Receptor Delta	Protein Coding	52	GC06P092273	13.99849129	https://www.genecards.org/cgi-bin/carddisp.pl?gene=PPARD
UCP2	Uncoupling Protein 2	Protein Coding	52	GC11M073974	13.88011551	https://www.genecards.org/cgi-bin/carddisp.pl?gene=UCP2
GSTP1	Glutathione S-Transferase Pi 1	Protein Coding	57	GC11P067583	13.80813503	https://www.genecards.org/cgi-bin/carddisp.pl?gene=GSTP1
ESR1	Estrogen Receptor 1	Protein Coding	61	GC06P151656	13.80347061	https://www.genecards.org/cgi-bin/carddisp.pl?gene=ESR1
PTGS2	Prostaglandin-Endoperoxide Synthase 2	Protein Coding	56	GC01M186671	13.66300106	https://www.genecards.org/cgi-bin/carddisp.pl?gene=PTGS2
PON1	Paraoxonase 1	Protein Coding	55	GC07M095297	13.57410049	https://www.genecards.org/cgi-bin/carddisp.pl?gene=PON1
ETFA	Electron Transfer Flavoprotein Subunit Alpha	Protein Coding	52	GC15M122358	13.51145744	https://www.genecards.org/cgi-bin/carddisp.pl?gene=ETFA
HMGCR	3-Hydroxy-3-Methylglutaryl-CoA Reductase	Protein Coding	53	GC05P075336	13.458951	https://www.genecards.org/cgi-bin/carddisp.pl?gene=HMGCR
FAH	Fumarylacetoacetate Hydrolase	Protein Coding	53	GC15P080152	13.40405846	https://www.genecards.org/cgi-bin/carddisp.pl?gene=FAH
PSAP	Prosaposin	Protein Coding	54	GC10M071816	13.35322666	https://www.genecards.org/cgi-bin/carddisp.pl?gene=PSAP
CP	Ceruloplasmin	Protein Coding	55	GC03M149162	13.33574104	https://www.genecards.org/cgi-bin/carddisp.pl?gene=CP
COMT	Catechol-O-Methyltransferase	Protein Coding	59	GC22P019941	13.30807877	https://www.genecards.org/cgi-bin/carddisp.pl?ge

						ne=COMT
SREBF1	Sterol Regulatory Element Binding Transcription Factor 1	Protein Coding	53	GC17M017810	13.25699425	https://www.genecards.org/cgi-bin/carddisp.pl?gene=SREBF1
NR1H2	Nuclear Receptor Subfamily 1 Group H Member 2	Protein Coding	53	GC19P050329	13.08687782	https://www.genecards.org/cgi-bin/carddisp.pl?gene=NR1H2
ASL	Argininosuccinate Lyase	Protein Coding	52	GC07P066075	12.97810268	https://www.genecards.org/cgi-bin/carddisp.pl?gene=ASL
HADH	Hydroxyacyl-CoA Dehydrogenase	Protein Coding	54	GC04P107989	12.95173168	https://www.genecards.org/cgi-bin/carddisp.pl?gene=HADH
SMPD1	Sphingomyelin Phosphodiesterase 1	Protein Coding	56	GC11P006390	12.94299412	https://www.genecards.org/cgi-bin/carddisp.pl?gene=SMPD1
COX5A	Cytochrome C Oxidase Subunit 5A	Protein Coding	50	GC15M074919	12.93949127	https://www.genecards.org/cgi-bin/carddisp.pl?gene=COX5A
REN	Renin	Protein Coding	55	GC01M204154	12.88049412	https://www.genecards.org/cgi-bin/carddisp.pl?gene=REN
GSS	Glutathione Synthetase	Protein Coding	53	GC20M034928	12.87272263	https://www.genecards.org/cgi-bin/carddisp.pl?gene=GSS
G6PD	Glucose-6-Phosphate Dehydrogenase	Protein Coding	57	GC0XM154562	12.85865021	https://www.genecards.org/cgi-bin/carddisp.pl?gene=G6PD
SLC16A1	Solute Carrier Family 16 Member 1	Protein Coding	54	GC01M113013	12.82639217	https://www.genecards.org/cgi-bin/carddisp.pl?gene=SLC16A1
CD36	CD36 Molecule	Protein Coding	56	GC07P080369	12.77978039	https://www.genecards.org/cgi-bin/carddisp.pl?gene=CD36
NPC1	NPC Intracellular Cholesterol Transporter 1	Protein Coding	56	GC18M023506	12.75236893	https://www.genecards.org/cgi-bin/carddisp.pl?gene=NPC1
DDC	Dopa Decarboxylase	Protein Coding	57	GC07M050458	12.74859524	https://www.genecards.org/cgi-bin/carddisp.pl?gene=DDC
APOA5	Apolipoprotein A5	Protein Coding	51	GC11M116789	12.7049284	https://www.genecards.org/cgi-bin/carddisp.pl?gene=APOA5
CYP21A2	Cytochrome P450 Family 21 Subfamily A Member 2	Protein Coding	51	GC06P092176	12.66181755	https://www.genecards.org/cgi-bin/carddisp.pl?ge

						ne=CYP21A2
CXCL8	C-X-C Motif Chemokine Ligand 8	Protein Coding	50	GC04P073740	12.65779114	https://www.genecards.org/cgi-bin/carddisp.pl?gene=CXCL8
UGT1A3	UDP Glucuronosyltransferase Family 1 Member A3	Protein Coding	44	GC02P233729	12.65203857	https://www.genecards.org/cgi-bin/carddisp.pl?gene=UGT1A3
EPHX1	Epoxide Hydrolase 1	Protein Coding	52	GC01P225810	12.62948704	https://www.genecards.org/cgi-bin/carddisp.pl?gene=EPHX1
MLYCD	Malonyl-CoA Decarboxylase	Protein Coding	48	GC16P083899	12.62700081	https://www.genecards.org/cgi-bin/carddisp.pl?gene=MLYCD
CYP24A1	Cytochrome P450 Family 24 Subfamily A Member 1	Protein Coding	53	GC20M054153	12.61680889	https://www.genecards.org/cgi-bin/carddisp.pl?gene=CYP24A1
PDHA1	Pyruvate Dehydrogenase E1 Subunit Alpha 1	Protein Coding	54	GC0XP019343	12.60220623	https://www.genecards.org/cgi-bin/carddisp.pl?gene=PDHA1
PRKAA2	Protein Kinase AMP-Activated Catalytic Subunit Alpha 2	Protein Coding	57	GC01P056645	12.5937233	https://www.genecards.org/cgi-bin/carddisp.pl?gene=PRKAA2
SOD1	Superoxide Dismutase 1	Protein Coding	60	GC21P031659	12.58113956	https://www.genecards.org/cgi-bin/carddisp.pl?gene=SOD1
UGT1A10	UDP Glucuronosyltransferase Family 1 Member A10	Protein Coding	48	GC02P233636	12.54686928	https://www.genecards.org/cgi-bin/carddisp.pl?gene=UGT1A10
SLC17A5	Solute Carrier Family 17 Member 5	Protein Coding	51	GC06M073593	12.54128933	https://www.genecards.org/cgi-bin/carddisp.pl?gene=SLC17A5
NAT2	N-Acetyltransferase 2	Protein Coding	47	GC08P018391	12.47551346	https://www.genecards.org/cgi-bin/carddisp.pl?gene=NAT2
LIPE	Lipase E, Hormone Sensitive Type	Protein Coding	54	GC19M042401	12.46535015	https://www.genecards.org/cgi-bin/carddisp.pl?gene=LIPE
PLA2G4A	Phospholipase A2 Group IVA	Protein Coding	55	GC01P186798	12.43510914	https://www.genecards.org/cgi-bin/carddisp.pl?gene=PLA2G4A
ACACA	Acetyl-CoA Carboxylase Alpha	Protein Coding	55	GC17M037084	12.39061165	https://www.genecards.org/cgi-bin/carddisp.pl?gene=ACACA
PRKAA1	Protein Kinase AMP-Activated Catalytic Subunit Alpha 1	Protein Coding	54	GC05M040759	12.38722038	https://www.genecards.org/cgi-bin/carddisp.pl?ge

						ne=PRKAA1
ADA	Adenosine Deaminase	Protein Coding	57	GC20M044620	12.38394356	https://www.genecards.org/cgi-bin/carddisp.pl?gene=ADA
NR1I2	Nuclear Receptor Subfamily 1 Group I Member 2	Protein Coding	51	GC03P119780	12.3428793	https://www.genecards.org/cgi-bin/carddisp.pl?gene=NR1I2
QDPR	Quinoid Dihydropteridine Reductase	Protein Coding	53	GC04M017460	12.31072426	https://www.genecards.org/cgi-bin/carddisp.pl?gene=QDPR
ALDH6A1	Aldehyde Dehydrogenase 6 Family Member A1	Protein Coding	52	GC14M074059	12.25684834	https://www.genecards.org/cgi-bin/carddisp.pl?gene=ALDH6A1
ABCC2	ATP Binding Cassette Subfamily C Member 2	Protein Coding	55	GC10P099782	12.24063396	https://www.genecards.org/cgi-bin/carddisp.pl?gene=ABCC2
F2	Coagulation Factor II, Thrombin	Protein Coding	56	GC11P046720	12.21356201	https://www.genecards.org/cgi-bin/carddisp.pl?gene=F2
DLD	Dihydrolipoamide Dehydrogenase	Protein Coding	56	GC07P107890	12.20763779	https://www.genecards.org/cgi-bin/carddisp.pl?gene=DLD
PYGM	Glycogen Phosphorylase, Muscle Associated	Protein Coding	53	GC11M064746	12.18904305	https://www.genecards.org/cgi-bin/carddisp.pl?gene=PYGM
HBB	Hemoglobin Subunit Beta	Protein Coding	51	GC11M006827	12.17414379	https://www.genecards.org/cgi-bin/carddisp.pl?gene=HBB
CCL2	C-C Motif Chemokine Ligand 2	Protein Coding	56	GC17P034255	12.15099144	https://www.genecards.org/cgi-bin/carddisp.pl?gene=CCL2
MCCC2	Methylcrotonyl-CoA Carboxylase Subunit 2	Protein Coding	51	GC05P073552	12.12744713	https://www.genecards.org/cgi-bin/carddisp.pl?gene=MCCC2
ARSA	Arylsulfatase A	Protein Coding	53	GC22M050622	12.1175108	https://www.genecards.org/cgi-bin/carddisp.pl?gene=ARSA
UGT1A7	UDP Glucuronosyltransferase Family 1 Member A7	Protein Coding	47	GC02P233681	12.10301018	https://www.genecards.org/cgi-bin/carddisp.pl?gene=UGT1A7
KL	Klotho	Protein Coding	54	GC13P033016	12.0826025	https://www.genecards.org/cgi-bin/carddisp.pl?gene=KL
SERPINE1	Serpin Family E Member 1	Protein Coding	57	GC07P101127	12.04139709	https://www.genecards.org/cgi-bin/carddisp.pl?ge

						ne=SERPINE1
DYRK1B	Dual Specificity Tyrosine Phosphorylation Regulated Kinase 1B	Protein Coding	53	GC19M039825	12.03226757	https://www.genecards.org/cgi-bin/carddisp.pl?gene=DYRK1B
SUOX	Sulfite Oxidase	Protein Coding	51	GC12P055997	12.02076054	https://www.genecards.org/cgi-bin/carddisp.pl?gene=SUOX
NR1H4	Nuclear Receptor Subfamily 1 Group H Member 4	Protein Coding	55	GC12P100473	12.00567818	https://www.genecards.org/cgi-bin/carddisp.pl?gene=NR1H4
BCKDK	Branched Chain Keto Acid Dehydrogenase Kinase	Protein Coding	52	GC16P042844	11.98306084	https://www.genecards.org/cgi-bin/carddisp.pl?gene=BCKDK
PTEN	Phosphatase And Tensin Homolog	Protein Coding	59	GC10P094594	11.97610664	https://www.genecards.org/cgi-bin/carddisp.pl?gene=PTEN
CS	Citrate Synthase	Protein Coding	51	GC12M056271	11.92381477	https://www.genecards.org/cgi-bin/carddisp.pl?gene=CS
ALDOB	Aldolase, Fructose-Bisphosphate B	Protein Coding	52	GC09M101420	11.91490173	https://www.genecards.org/cgi-bin/carddisp.pl?gene=ALDOB
APOA2	Apolipoprotein A2	Protein Coding	52	GC01M161222	11.90166092	https://www.genecards.org/cgi-bin/carddisp.pl?gene=APOA2
IFNG	Interferon Gamma	Protein Coding	57	GC12M068154	11.8699913	https://www.genecards.org/cgi-bin/carddisp.pl?gene=IFNG
DPP4	Dipeptidyl Peptidase 4	Protein Coding	57	GC02M161992	11.85106945	https://www.genecards.org/cgi-bin/carddisp.pl?gene=DPP4
PNP	Purine Nucleoside Phosphorylase	Protein Coding	56	GC14P034354	11.83236408	https://www.genecards.org/cgi-bin/carddisp.pl?gene=PNP
ALPL	Alkaline Phosphatase, Biomineralization Associated	Protein Coding	58	GC01P021508	11.82943535	https://www.genecards.org/cgi-bin/carddisp.pl?gene=ALPL
ALOX5	Arachidonate 5-Lipoxygenase	Protein Coding	54	GC10P045374	11.82166386	https://www.genecards.org/cgi-bin/carddisp.pl?gene=ALOX5
GATM	Glycine Amidinotransferase	Protein Coding	52	GC15M045361	11.68083858	https://www.genecards.org/cgi-bin/carddisp.pl?gene=GATM
MCCC1	Methylcrotonyl-CoA Carboxylase Subunit 1	Protein Coding	50	GC03M183015	11.66801453	https://www.genecards.org/cgi-bin/carddisp.pl?ge

						ne=MCCC1
BCHE	Butyrylcholinesterase	Protein Coding	57	GC03M165772	11.63358974	https://www.genecards.org/cgi-bin/carddisp.pl?gene=BCHE
PIK3CA	Phosphatidylinositol-4,5-Bisphosphate 3-Kinase Catalytic Subunit Alpha	Protein Coding	59	GC03P179148	11.63045788	https://www.genecards.org/cgi-bin/carddisp.pl?gene=PIK3CA
MPO	Myeloperoxidase	Protein Coding	59	GC17M058269	11.59176159	https://www.genecards.org/cgi-bin/carddisp.pl?gene=MPO
ARG1	Arginase 1	Protein Coding	56	GC06P131473	11.57893658	https://www.genecards.org/cgi-bin/carddisp.pl?gene=ARG1
SLC4A1	Solute Carrier Family 4 Member 1 (Diego Blood Group)	Protein Coding	54	GC17M051897	11.54837894	https://www.genecards.org/cgi-bin/carddisp.pl?gene=SLC4A1
FABP4	Fatty Acid Binding Protein 4	Protein Coding	50	GC08M081478	11.49729919	https://www.genecards.org/cgi-bin/carddisp.pl?gene=FABP4
CAT	Catalase	Protein Coding	58	GC11P034460	11.48341846	https://www.genecards.org/cgi-bin/carddisp.pl?gene=CAT
STAR	Steroidogenic Acute Regulatory Protein	Protein Coding	51	GC08M038145	11.43203926	https://www.genecards.org/cgi-bin/carddisp.pl?gene=STAR
ABCC8	ATP Binding Cassette Subfamily C Member 8	Protein Coding	53	GC11M017392	11.42554283	https://www.genecards.org/cgi-bin/carddisp.pl?gene=ABCC8
OGDH	Oxoglutarate Dehydrogenase	Protein Coding	53	GC07P044606	11.42041874	https://www.genecards.org/cgi-bin/carddisp.pl?gene=OGDH
NQO1	NAD(P)H Quinone Dehydrogenase 1	Protein Coding	55	GC16M069706	11.40145969	https://www.genecards.org/cgi-bin/carddisp.pl?gene=NQO1
HADHB	Hydroxyacyl-CoA Dehydrogenase Trifunctional Multienzyme Complex Subunit Beta	Protein Coding	54	GC02P026243	11.36002731	https://www.genecards.org/cgi-bin/carddisp.pl?gene=HADHB
SULT1A1	Sulfotransferase Family 1A Member 1	Protein Coding	50	GC16M038249	11.33466053	https://www.genecards.org/cgi-bin/carddisp.pl?gene=SULT1A1
MVK	Mevalonate Kinase	Protein Coding	54	GC12P109573	11.33141518	https://www.genecards.org/cgi-bin/carddisp.pl?gene=MVK
AMPD3	Adenosine Monophosphate Deaminase 3	Protein Coding	51	GC11P010309	11.29901218	https://www.genecards.org/cgi-bin/carddisp.pl?ge

						ne=AMPD3
FABP2	Fatty Acid Binding Protein 2	Protein Coding	48	GC04M119317	11.29714584	https://www.genecards.org/cgi-bin/carddisp.pl?gene=FABP2
UGT1A	UDP Glucuronosyltransferase Family 1 Member A Complex Locus	Uncategorized	13	GC02P233592	11.28607178	https://www.genecards.org/cgi-bin/carddisp.pl?gene=UGT1A
TF	Transferrin	Protein Coding	57	GC03P135056	11.27254391	https://www.genecards.org/cgi-bin/carddisp.pl?gene=TF
RYR1	Ryanodine Receptor 1	Protein Coding	53	GC19P072806	11.22693443	https://www.genecards.org/cgi-bin/carddisp.pl?gene=RYR1
SDHB	Succinate Dehydrogenase Complex Iron Sulfur Subunit B	Protein Coding	54	GC01M018297	11.20557117	https://www.genecards.org/cgi-bin/carddisp.pl?gene=SDHB
CYP3A7	Cytochrome P450 Family 3 Subfamily A Member 7	Protein Coding	47	GC07M099705	11.19146252	https://www.genecards.org/cgi-bin/carddisp.pl?gene=CYP3A7
ACSS2	Acyl-CoA Synthetase Short Chain Family Member 2	Protein Coding	52	GC20P034873	11.14794254	https://www.genecards.org/cgi-bin/carddisp.pl?gene=ACSS2
ACAT1	Acetyl-CoA Acetyltransferase 1	Protein Coding	56	GC11P108121	11.1475544	https://www.genecards.org/cgi-bin/carddisp.pl?gene=ACAT1
CTH	Cystathionine Gamma-Lyase	Protein Coding	56	GC01P070411	11.13909245	https://www.genecards.org/cgi-bin/carddisp.pl?gene=CTH
AR	Androgen Receptor	Protein Coding	59	GC0XP067544	11.10406113	https://www.genecards.org/cgi-bin/carddisp.pl?gene=AR
GNAS	GNAS Complex Locus	Protein Coding	56	GC20P058839	11.09564114	https://www.genecards.org/cgi-bin/carddisp.pl?gene=GNAS
GCSH	Glycine Cleavage System Protein H	Protein Coding	49	GC16M081081	11.08065605	https://www.genecards.org/cgi-bin/carddisp.pl?gene=GCSH
PNPLA3	Patatin Like Phospholipase Domain Containing 3	Protein Coding	48	GC22P043923	11.0245285	https://www.genecards.org/cgi-bin/carddisp.pl?gene=PNPLA3
FGF21	Fibroblast Growth Factor 21	Protein Coding	46	GC19P073194	10.99773979	https://www.genecards.org/cgi-bin/carddisp.pl?gene=FGF21
CPOX	Coproporphyrinogen Oxidase	Protein Coding	51	GC03M098576	10.99168968	https://www.genecards.org/cgi-bin/carddisp.pl?ge

						ne=CPOX
CNR1	Cannabinoid Receptor 1	Protein Coding	53	GC06M088139	10.95663166	https://www.genecards.org/cgi-bin/carddisp.pl?gene=CNR1
TAT	Tyrosine Aminotransferase	Protein Coding	52	GC16M071565	10.91602612	https://www.genecards.org/cgi-bin/carddisp.pl?gene=TAT
GRIA2	Glutamate Ionotropic Receptor AMPA Type Subunit 2	Protein Coding	58	GC04P157204	10.91508484	https://www.genecards.org/cgi-bin/carddisp.pl?gene=GRIA2
EHHADH	Enoyl-CoA Hydratase And 3-Hydroxyacyl CoA Dehydrogenase	Protein Coding	50	GC03M185190	10.90606213	https://www.genecards.org/cgi-bin/carddisp.pl?gene=EHHADH
CFTR	CF Transmembrane Conductance Regulator	Protein Coding	59	GC07P117287	10.90104771	https://www.genecards.org/cgi-bin/carddisp.pl?gene=CFTR
UPB1	Beta-Ureidopropionase 1	Protein Coding	51	GC22P024494	10.89578056	https://www.genecards.org/cgi-bin/carddisp.pl?gene=UPB1
PTGS1	Prostaglandin-Endoperoxide Synthase 1	Protein Coding	53	GC09P122370	10.85939598	https://www.genecards.org/cgi-bin/carddisp.pl?gene=PTGS1
RBP4	Retinol Binding Protein 4	Protein Coding	52	GC10M093591	10.77317238	https://www.genecards.org/cgi-bin/carddisp.pl?gene=RBP4
HMOX1	Heme Oxygenase 1	Protein Coding	59	GC22P035380	10.77141571	https://www.genecards.org/cgi-bin/carddisp.pl?gene=HMOX1
POR	Cytochrome P450 Oxidoreductase	Protein Coding	56	GC07P075899	10.76194668	https://www.genecards.org/cgi-bin/carddisp.pl?gene=POR
MB	Myoglobin	Protein Coding	50	GC22M035606	10.72063637	https://www.genecards.org/cgi-bin/carddisp.pl?gene=MB
SDHA	Succinate Dehydrogenase Complex Flavoprotein Subunit A	Protein Coding	54	GC05P000220	10.72057343	https://www.genecards.org/cgi-bin/carddisp.pl?gene=SDHA
ABCG2	ATP Binding Cassette Subfamily G Member 2 (Junior Blood Group)	Protein Coding	56	GC04M088090	10.62441158	https://www.genecards.org/cgi-bin/carddisp.pl?gene=ABCG2
DGAT1	Diacylglycerol O-Acyltransferase 1	Protein Coding	55	GC08M144325	10.61318207	https://www.genecards.org/cgi-bin/carddisp.pl?gene=DGAT1
CYP11B2	Cytochrome P450 Family 11 Subfamily B Member 2	Protein Coding	53	GC08M142910	10.61117077	https://www.genecards.org/cgi-bin/carddisp.pl?ge

						ne=CYP11B2
UGT2B7	UDP Glucuronosyltransferase Family 2 Member B7	Protein Coding	50	GC04P069051	10.59724236	https://www.genecards.org/cgi-bin/carddisp.pl?gene=UGT2B7
CYP27A1	Cytochrome P450 Family 27 Subfamily A Member 1	Protein Coding	53	GC02P218781	10.5703907	https://www.genecards.org/cgi-bin/carddisp.pl?gene=CYP27A1
SLC25A20	Solute Carrier Family 25 Member 20	Protein Coding	51	GC03M048864	10.55376339	https://www.genecards.org/cgi-bin/carddisp.pl?gene=SLC25A20
PRL	Prolactin	Protein Coding	50	GC06M022287	10.54038048	https://www.genecards.org/cgi-bin/carddisp.pl?gene=PRL
UGT1A4	UDP Glucuronosyltransferase Family 1 Member A4	Protein Coding	49	GC02P233718	10.53925133	https://www.genecards.org/cgi-bin/carddisp.pl?gene=UGT1A4
VCAM1	Vascular Cell Adhesion Molecule 1	Protein Coding	52	GC01P100719	10.49340725	https://www.genecards.org/cgi-bin/carddisp.pl?gene=VCAM1
KYNU	Kynureninase	Protein Coding	55	GC02P142877	10.47294903	https://www.genecards.org/cgi-bin/carddisp.pl?gene=KYNU
SRD5A2	Steroid 5 Alpha-Reductase 2	Protein Coding	49	GC02M031522	10.46125317	https://www.genecards.org/cgi-bin/carddisp.pl?gene=SRD5A2
TYMP	Thymidine Phosphorylase	Protein Coding	54	GC22M050525	10.40325737	https://www.genecards.org/cgi-bin/carddisp.pl?gene=TYMP
MC4R	Melanocortin 4 Receptor	Protein Coding	53	GC18M060371	10.38755989	https://www.genecards.org/cgi-bin/carddisp.pl?gene=MC4R
NDUFS4	NADH:Ubiquinone Oxidoreductase Subunit S4	Protein Coding	50	GC05P053560	10.37858486	https://www.genecards.org/cgi-bin/carddisp.pl?gene=NDUFS4
PCK2	Phosphoenolpyruvate Carboxykinase 2, Mitochondrial	Protein Coding	53	GC14P024094	10.35907841	https://www.genecards.org/cgi-bin/carddisp.pl?gene=PCK2
SCD	Stearoyl-CoA Desaturase	Protein Coding	57	GC10P100347	10.35372066	https://www.genecards.org/cgi-bin/carddisp.pl?gene=SCD
GSR	Glutathione-Disulfide Reductase	Protein Coding	56	GC08M030678	10.34006882	https://www.genecards.org/cgi-bin/carddisp.pl?gene=GSR
GRIA1	Glutamate Ionotropic Receptor AMPA Type Subunit 1	Protein Coding	56	GC05P153467	10.3355341	https://www.genecards.org/cgi-bin/carddisp.pl?ge

						ne=GRIA1
HMGCS2	3-Hydroxy-3-Methylglutaryl-CoA Synthase 2	Protein Coding	53	GC01M119747	10.30286884	https://www.genecards.org/cgi-bin/carddisp.pl?gene=HMGCS2
FFAR2	Free Fatty Acid Receptor 2	Protein Coding	48	GC19P072731	10.30257607	https://www.genecards.org/cgi-bin/carddisp.pl?gene=FFAR2
BLOC1S1	Biogenesis Of Lysosomal Organelles Complex 1 Subunit 1	Protein Coding	42	GC12P055950	10.30231476	https://www.genecards.org/cgi-bin/carddisp.pl?gene=BLOC1S1
GH1	Growth Hormone 1	Protein Coding	50	GC17M063917	10.25849533	https://www.genecards.org/cgi-bin/carddisp.pl?gene=GH1
KCNJ1	Potassium Inwardly Rectifying Channel Subfamily J Member 1	Protein Coding	54	GC11M128741	10.25777531	https://www.genecards.org/cgi-bin/carddisp.pl?gene=KCNJ1
APP	Amyloid Beta Precursor Protein	Protein Coding	58	GC21M025880	10.25391865	https://www.genecards.org/cgi-bin/carddisp.pl?gene=APP
EPHX2	Epoxide Hydrolase 2	Protein Coding	53	GC08P027490	10.23595047	https://www.genecards.org/cgi-bin/carddisp.pl?gene=EPHX2
PTPN1	Protein Tyrosine Phosphatase Non-Receptor Type 1	Protein Coding	57	GC20P050510	10.23029804	https://www.genecards.org/cgi-bin/carddisp.pl?gene=PTPN1
GOT2	Glutamic-Oxaloacetic Transaminase 2	Protein Coding	53	GC16M058707	10.2194376	https://www.genecards.org/cgi-bin/carddisp.pl?gene=GOT2
KCNJ11	Potassium Inwardly Rectifying Channel Subfamily J Member 11	Protein Coding	53	GC11M017663	10.20500565	https://www.genecards.org/cgi-bin/carddisp.pl?gene=KCNJ11
NR1I3	Nuclear Receptor Subfamily 1 Group I Member 3	Protein Coding	51	GC01M161229	10.19310284	https://www.genecards.org/cgi-bin/carddisp.pl?gene=NR1I3
CYP11B1	Cytochrome P450 Family 11 Subfamily B Member 1	Protein Coding	52	GC08M142872	10.17906284	https://www.genecards.org/cgi-bin/carddisp.pl?gene=CYP11B1
HK2	Hexokinase 2	Protein Coding	52	GC02P074833	10.16204357	https://www.genecards.org/cgi-bin/carddisp.pl?gene=HK2
ATP7A	ATPase Copper Transporting Alpha	Protein Coding	52	GC0XP078031	10.16186047	https://www.genecards.org/cgi-bin/carddisp.pl?gene=ATP7A
MTOR	Mechanistic Target Of Rapamycin Kinase	Protein Coding	62	GC01M011106	10.12988853	https://www.genecards.org/cgi-bin/carddisp.pl?ge

						ne=MTOR
IGFBP3	Insulin Like Growth Factor Binding Protein 3	Protein Coding	54	GC07M045912	10.12064362	https://www.genecards.org/cgi-bin/carddisp.pl?gene=IGFBP3
TCN2	Transcobalamin 2	Protein Coding	50	GC22P044849	10.1113596	https://www.genecards.org/cgi-bin/carddisp.pl?gene=TCN2
BDNF	Brain Derived Neurotrophic Factor	Protein Coding	56	GC11M027654	10.10180569	https://www.genecards.org/cgi-bin/carddisp.pl?gene=BDNF
AKR1A1	Aldo-Keto Reductase Family 1 Member A1	Protein Coding	51	GC01P045550	10.08849812	https://www.genecards.org/cgi-bin/carddisp.pl?gene=AKR1A1
UCP1	Uncoupling Protein 1	Protein Coding	50	GC04M140559	10.07172298	https://www.genecards.org/cgi-bin/carddisp.pl?gene=UCP1
SERPINA1	Serpin Family A Member 1	Protein Coding	56	GC14M094376	10.03485966	https://www.genecards.org/cgi-bin/carddisp.pl?gene=SERPINA1
TTR	Transthyretin	Protein Coding	55	GC18P031557	10.01377964	https://www.genecards.org/cgi-bin/carddisp.pl?gene=TTR
CYP4F2	Cytochrome P450 Family 4 Subfamily F Member 2	Protein Coding	50	GC19M015878	9.978006363	https://www.genecards.org/cgi-bin/carddisp.pl?gene=CYP4F2
ALDH9A1	Aldehyde Dehydrogenase 9 Family Member A1	Protein Coding	49	GC01M165681	9.949209213	https://www.genecards.org/cgi-bin/carddisp.pl?gene=ALDH9A1
AKT2	AKT Serine/Threonine Kinase 2	Protein Coding	61	GC19M040230	9.924123764	https://www.genecards.org/cgi-bin/carddisp.pl?gene=AKT2
HNF1A	HNF1 Homeobox A	Protein Coding	53	GC12P120978	9.914362907	https://www.genecards.org/cgi-bin/carddisp.pl?gene=HNF1A
PPIG	Peptidylprolyl Isomerase G	Protein Coding	48	GC02P169584	9.875458717	https://www.genecards.org/cgi-bin/carddisp.pl?gene=PPIG
LIPA	Lipase A, Lysosomal Acid Type	Protein Coding	55	GC10M089213	9.852193832	https://www.genecards.org/cgi-bin/carddisp.pl?gene=LIPA
SCNN1G	Sodium Channel Epithelial 1 Subunit Gamma	Protein Coding	53	GC16P023182	9.823068619	https://www.genecards.org/cgi-bin/carddisp.pl?gene=SCNN1G
UROC1	Urocanate Hydratase 1	Protein Coding	46	GC03M126481	9.821082115	https://www.genecards.org/cgi-bin/carddisp.pl?ge

						ne=UROC1
PCK1	Phosphoenolpyruvate Carboxykinase 1	Protein Coding	54	GC20P057561	9.816595078	https://www.genecards.org/cgi-bin/carddisp.pl?gene=PCK1
NPY	Neuropeptide Y	Protein Coding	51	GC07P024290	9.814024925	https://www.genecards.org/cgi-bin/carddisp.pl?gene=NPY
SLC25A15	Solute Carrier Family 25 Member 15	Protein Coding	50	GC13P040789	9.767385483	https://www.genecards.org/cgi-bin/carddisp.pl?gene=SLC25A15
MT-ND6	Mitochondrially Encoded NADH:Ubiquinone Oxidoreductase Core Subunit 6	Protein Coding	38	GCMTM014151	9.757986069	https://www.genecards.org/cgi-bin/carddisp.pl?gene=MT-ND6
HGD	Homogentisate 1,2-Dioxygenase	Protein Coding	50	GC03M120628	9.720542908	https://www.genecards.org/cgi-bin/carddisp.pl?gene=HGD
SERPINA3	Serpin Family A Member 3	Protein Coding	50	GC14P094612	9.719280243	https://www.genecards.org/cgi-bin/carddisp.pl?gene=SERPINA3
ASAH1	N-Acylsphingosine Amidohydrolase 1	Protein Coding	56	GC08M018055	9.712446213	https://www.genecards.org/cgi-bin/carddisp.pl?gene=ASAH1
PDP1	Pyruvate Dehydrogenase Phosphatase Catalytic Subunit 1	Protein Coding	54	GC08P093857	9.703587532	https://www.genecards.org/cgi-bin/carddisp.pl?gene=PDP1
ABCC1	ATP Binding Cassette Subfamily C Member 1	Protein Coding	56	GC16P015949	9.692297935	https://www.genecards.org/cgi-bin/carddisp.pl?gene=ABCC1
DPYS	Dihydropyrimidinase	Protein Coding	52	GC08M104331	9.645685196	https://www.genecards.org/cgi-bin/carddisp.pl?gene=DPYS
SLC5A6	Solute Carrier Family 5 Member 6	Protein Coding	51	GC02M027201	9.638032913	https://www.genecards.org/cgi-bin/carddisp.pl?gene=SLC5A6
TPI1	Triosephosphate Isomerase 1	Protein Coding	54	GC12P006867	9.596486092	https://www.genecards.org/cgi-bin/carddisp.pl?gene=TPI1
PRKAB1	Protein Kinase AMP-Activated Non-Catalytic Subunit Beta 1	Protein Coding	53	GC12P119632	9.594614029	https://www.genecards.org/cgi-bin/carddisp.pl?gene=PRKAB1
ABCB11	ATP Binding Cassette Subfamily B Member 11	Protein Coding	53	GC02M168922	9.566059113	https://www.genecards.org/cgi-bin/carddisp.pl?gene=ABCB11
TNFSF11	TNF Superfamily Member 11	Protein Coding	56	GC13P042562	9.559355736	https://www.genecards.org/cgi-bin/carddisp.pl?ge

						ne=TNFSF11
PHKA2	Phosphorylase Kinase Regulatory Subunit Alpha 2	Protein Coding	51	GC0XM018892	9.55429554	https://www.genecards.org/cgi-bin/carddisp.pl?gene=PHKA2
EPO	Erythropoietin	Protein Coding	49	GC07P100720	9.553358078	https://www.genecards.org/cgi-bin/carddisp.pl?gene=EPO
EPRS1	Glutamyl-Prolyl-TRNA Synthetase 1	Protein Coding	50	GC01M219969	9.552751541	https://www.genecards.org/cgi-bin/carddisp.pl?gene=EPRS1
MAOA	Monoamine Oxidase A	Protein Coding	55	GC0XP043654	9.547767639	https://www.genecards.org/cgi-bin/carddisp.pl?gene=MAOA
ACAT2	Acetyl-CoA Acetyltransferase 2	Protein Coding	51	GC06P160802	9.544605255	https://www.genecards.org/cgi-bin/carddisp.pl?gene=ACAT2
SCARB1	Scavenger Receptor Class B Member 1	Protein Coding	55	GC12M124776	9.489313126	https://www.genecards.org/cgi-bin/carddisp.pl?gene=SCARB1
MT-CYB	Mitochondrially Encoded Cytochrome B	Protein Coding	38	GCMTP014749	9.488700867	https://www.genecards.org/cgi-bin/carddisp.pl?gene=MT-CYB
BCL2	BCL2 Apoptosis Regulator	Protein Coding	57	GC18M063123	9.478082657	https://www.genecards.org/cgi-bin/carddisp.pl?gene=BCL2
BMP6	Bone Morphogenetic Protein 6	Protein Coding	50	GC06P007726	9.47426033	https://www.genecards.org/cgi-bin/carddisp.pl?gene=BMP6
ANGPTL4	Angiopoietin Like 4	Protein Coding	52	GC19P008363	9.469734192	https://www.genecards.org/cgi-bin/carddisp.pl?gene=ANGPTL4
MAPK1	Mitogen-Activated Protein Kinase 1	Protein Coding	59	GC22M021759	9.460432053	https://www.genecards.org/cgi-bin/carddisp.pl?gene=MAPK1
SREBF2	Sterol Regulatory Element Binding Transcription Factor 2	Protein Coding	50	GC22P041833	9.449868202	https://www.genecards.org/cgi-bin/carddisp.pl?gene=SREBF2
SLC6A8	Solute Carrier Family 6 Member 8	Protein Coding	53	GC0XP153688	9.426587105	https://www.genecards.org/cgi-bin/carddisp.pl?gene=SLC6A8
PGR	Progesterone Receptor	Protein Coding	56	GC11M102130	9.400068283	https://www.genecards.org/cgi-bin/carddisp.pl?gene=PGR
PKM	Pyruvate Kinase M1/2	Protein Coding	56	GC15M072199	9.394825935	https://www.genecards.org/cgi-bin/carddisp.pl?ge

						ne=PKM
PRPS1	Phosphoribosyl Pyrophosphate Synthetase 1	Protein Coding	54	GC0XP107628	9.386370659	https://www.genecards.org/cgi-bin/carddisp.pl?gene=PRPS1
AHR	Aryl Hydrocarbon Receptor	Protein Coding	54	GC07P016916	9.34418869	https://www.genecards.org/cgi-bin/carddisp.pl?gene=AHR
ACSS1	Acyl-CoA Synthetase Short Chain Family Member 1	Protein Coding	47	GC20M024986	9.330976486	https://www.genecards.org/cgi-bin/carddisp.pl?gene=ACSS1
RRM2B	Ribonucleotide Reductase Regulatory TP53 Inducible Subunit M2B	Protein Coding	56	GC08M102204	9.317185402	https://www.genecards.org/cgi-bin/carddisp.pl?gene=RRM2B
B3GAT1	Beta-1,3-Glucuronyltransferase 1	Protein Coding	50	GC11M134378	9.305902481	https://www.genecards.org/cgi-bin/carddisp.pl?gene=B3GAT1
MMP1	Matrix Metallopeptidase 1	Protein Coding	57	GC11M102810	9.27759552	https://www.genecards.org/cgi-bin/carddisp.pl?gene=MMP1
AOX1	Aldehyde Oxidase 1	Protein Coding	51	GC02P200585	9.275713921	https://www.genecards.org/cgi-bin/carddisp.pl?gene=AOX1
TFRC	Transferrin Receptor	Protein Coding	58	GC03M196056	9.241100311	https://www.genecards.org/cgi-bin/carddisp.pl?gene=TFRC
GAPDH	Glyceraldehyde-3-Phosphate Dehydrogenase	Protein Coding	57	GC12P023539	9.224225998	https://www.genecards.org/cgi-bin/carddisp.pl?gene=GAPDH
ALDH3A2	Aldehyde Dehydrogenase 3 Family Member A2	Protein Coding	52	GC17P019648	9.220035553	https://www.genecards.org/cgi-bin/carddisp.pl?gene=ALDH3A2
CYP26A1	Cytochrome P450 Family 26 Subfamily A Member 1	Protein Coding	50	GC10P093073	9.213516235	https://www.genecards.org/cgi-bin/carddisp.pl?gene=CYP26A1
SELE	Selectin E	Protein Coding	50	GC01M169722	9.211622238	https://www.genecards.org/cgi-bin/carddisp.pl?gene=SELE
ODC1	Ornithine Decarboxylase 1	Protein Coding	55	GC02M010432	9.199753761	https://www.genecards.org/cgi-bin/carddisp.pl?gene=ODC1
SI	Sucrase-Isomaltase	Protein Coding	51	GC03M164978	9.185455322	https://www.genecards.org/cgi-bin/carddisp.pl?gene=SI
IL4	Interleukin 4	Protein Coding	53	GC05P132673	9.176439285	https://www.genecards.org/cgi-bin/carddisp.pl?ge

						ne=IL4
TGFB1	Transforming Growth Factor Beta 1	Protein Coding	60	GC19M041301	9.174732208	https://www.genecards.org/cgi-bin/carddisp.pl?gene=TGFB1
CALCA	Calcitonin Related Polypeptide Alpha	Protein Coding	50	GC11M014945	9.166168213	https://www.genecards.org/cgi-bin/carddisp.pl?gene=CALCA
SARDH	Sarcosine Dehydrogenase	Protein Coding	48	GC09M133663	9.150572777	https://www.genecards.org/cgi-bin/carddisp.pl?gene=SARDH
IRS2	Insulin Receptor Substrate 2	Protein Coding	51	GC13M109752	9.141462326	https://www.genecards.org/cgi-bin/carddisp.pl?gene=IRS2
TSHR	Thyroid Stimulating Hormone Receptor	Protein Coding	54	GC14P080954	9.134458542	https://www.genecards.org/cgi-bin/carddisp.pl?gene=TSHR
ABAT	4-Aminobutyrate Aminotransferase	Protein Coding	52	GC16P008674	9.114062309	https://www.genecards.org/cgi-bin/carddisp.pl?gene=ABAT
PLA2G7	Phospholipase A2 Group VII	Protein Coding	56	GC06M046704	9.094242096	https://www.genecards.org/cgi-bin/carddisp.pl?gene=PLA2G7
PPOX	Protoporphyrinogen Oxidase	Protein Coding	50	GC01P161793	9.073926926	https://www.genecards.org/cgi-bin/carddisp.pl?gene=PPOX
BSND	Barttin CLCNK Type Accessory Subunit Beta	Protein Coding	46	GC01P054998	9.067605019	https://www.genecards.org/cgi-bin/carddisp.pl?gene=BSND
NFE2L2	NFE2 Like BZIP Transcription Factor 2	Protein Coding	57	GC02M177227	9.067396164	https://www.genecards.org/cgi-bin/carddisp.pl?gene=NFE2L2
TH	Tyrosine Hydroxylase	Protein Coding	57	GC11M002163	9.036359787	https://www.genecards.org/cgi-bin/carddisp.pl?gene=TH
GLP1R	Glucagon Like Peptide 1 Receptor	Protein Coding	53	GC06P039048	9.011398315	https://www.genecards.org/cgi-bin/carddisp.pl?gene=GLP1R
B2M	Beta-2-Microglobulin	Protein Coding	56	GC15P044711	9.009315491	https://www.genecards.org/cgi-bin/carddisp.pl?gene=B2M
ABCC4	ATP Binding Cassette Subfamily C Member 4	Protein Coding	52	GC13M095019	9.002803802	https://www.genecards.org/cgi-bin/carddisp.pl?gene=ABCC4
FECH	Ferrochelatase	Protein Coding	53	GC18M057544	8.987259865	https://www.genecards.org/cgi-bin/carddisp.pl?ge

						ne=FECH
SCNN1B	Sodium Channel Epithelial 1 Subunit Beta	Protein Coding	55	GC16P023278	8.966176987	https://www.genecards.org/cgi-bin/carddisp.pl?gene=SCNN1B
CAV1	Caveolin 1	Protein Coding	55	GC07P116524	8.965511322	https://www.genecards.org/cgi-bin/carddisp.pl?gene=CAV1
PLA2G6	Phospholipase A2 Group VI	Protein Coding	54	GC22M063106	8.922856331	https://www.genecards.org/cgi-bin/carddisp.pl?gene=PLA2G6
SLC9A3	Solute Carrier Family 9 Member A3	Protein Coding	54	GC05M000472	8.890376091	https://www.genecards.org/cgi-bin/carddisp.pl?gene=SLC9A3
KRAS	KRAS Proto-Oncogene, GTPase	Protein Coding	59	GC12M025204	8.88931179	https://www.genecards.org/cgi-bin/carddisp.pl?gene=KRAS
ACSS3	Acyl-CoA Synthetase Short Chain Family Member 3	Protein Coding	44	GC12P080936	8.88329792	https://www.genecards.org/cgi-bin/carddisp.pl?gene=ACSS3
PNLIP	Pancreatic Lipase	Protein Coding	55	GC10P116545	8.861320496	https://www.genecards.org/cgi-bin/carddisp.pl?gene=PNLIP
WNK1	WNK Lysine Deficient Protein Kinase 1	Protein Coding	55	GC12P000733	8.852413177	https://www.genecards.org/cgi-bin/carddisp.pl?gene=WNK1
AMACR	Alpha-Methylacyl-CoA Racemase	Protein Coding	51	GC05M033986	8.844797134	https://www.genecards.org/cgi-bin/carddisp.pl?gene=AMACR
TPO	Thyroid Peroxidase	Protein Coding	56	GC02P001374	8.836055756	https://www.genecards.org/cgi-bin/carddisp.pl?gene=TPO
PLTP	Phospholipid Transfer Protein	Protein Coding	51	GC20M045898	8.819312096	https://www.genecards.org/cgi-bin/carddisp.pl?gene=PLTP
PLA2G2A	Phospholipase A2 Group IIA	Protein Coding	53	GC01M019975	8.813098907	https://www.genecards.org/cgi-bin/carddisp.pl?gene=PLA2G2A
CBLIF	Cobalamin Binding Intrinsic Factor	Protein Coding	50	GC11M059829	8.777448654	https://www.genecards.org/cgi-bin/carddisp.pl?gene=CBLIF
ALOX12	Arachidonate 12-Lipoxygenase, 12S Type	Protein Coding	51	GC17P006995	8.752995491	https://www.genecards.org/cgi-bin/carddisp.pl?gene=ALOX12
PEPD	Peptidase D	Protein Coding	52	GC19M033386	8.72979641	https://www.genecards.org/cgi-bin/carddisp.pl?ge

						ne=PEPD
MAOB	Monoamine Oxidase B	Protein Coding	51	GC0XM043766	8.728715897	https://www.genecards.org/cgi-bin/carddisp.pl?gene=MAOB
SUCLG1	Succinate-CoA Ligase GDP/ADP-Forming Subunit Alpha	Protein Coding	53	GC02M084423	8.726793289	https://www.genecards.org/cgi-bin/carddisp.pl?gene=SUCLG1
NR1H3	Nuclear Receptor Subfamily 1 Group H Member 3	Protein Coding	53	GC11P047248	8.693900108	https://www.genecards.org/cgi-bin/carddisp.pl?gene=NR1H3
ALDOA	Aldolase, Fructose-Bisphosphate A	Protein Coding	55	GC16P030064	8.690598488	https://www.genecards.org/cgi-bin/carddisp.pl?gene=ALDOA
TALDO1	Transaldolase 1	Protein Coding	53	GC11P002101	8.67280674	https://www.genecards.org/cgi-bin/carddisp.pl?gene=TALDO1
PIK3C2A	Phosphatidylinositol-4-Phosphate 3-Kinase Catalytic Subunit Type 2 Alpha	Protein Coding	54	GC11M017656	8.672620773	https://www.genecards.org/cgi-bin/carddisp.pl?gene=PIK3C2A
MIR132	MicroRNA 132	RNA Gene	24	GC17M002049	8.668548584	https://www.genecards.org/cgi-bin/carddisp.pl?gene=MIR132
ALDH7A1	Aldehyde Dehydrogenase 7 Family Member A1	Protein Coding	55	GC05M126541	8.663873672	https://www.genecards.org/cgi-bin/carddisp.pl?gene=ALDH7A1
IGF1R	Insulin Like Growth Factor 1 Receptor	Protein Coding	61	GC15P098648	8.65890789	https://www.genecards.org/cgi-bin/carddisp.pl?gene=IGF1R
SERPINA6	Serpin Family A Member 6	Protein Coding	52	GC14M103107	8.658004761	https://www.genecards.org/cgi-bin/carddisp.pl?gene=SERPINA6
MYC	MYC Proto-Oncogene, BHLH Transcription Factor	Protein Coding	59	GC08P127735	8.656543732	https://www.genecards.org/cgi-bin/carddisp.pl?gene=MYC
FAS	Fas Cell Surface Death Receptor	Protein Coding	57	GC10P094616	8.649011612	https://www.genecards.org/cgi-bin/carddisp.pl?gene=FAS
HAL	Histidine Ammonia-Lyase	Protein Coding	50	GC12M095972	8.647676468	https://www.genecards.org/cgi-bin/carddisp.pl?gene=HAL
AKR1C3	Aldo-Keto Reductase Family 1 Member C3	Protein Coding	52	GC10P005035	8.643188477	https://www.genecards.org/cgi-bin/carddisp.pl?gene=AKR1C3
VEGFA	Vascular Endothelial Growth Factor A	Protein Coding	56	GC06P043770	8.640114784	https://www.genecards.org/cgi-bin/carddisp.pl?ge

						ne=VEGFA
NPPA	Natriuretic Peptide A	Protein Coding	52	GC01M011846	8.623929024	https://www.genecards.org/cgi-bin/carddisp.pl?gene=NPPA
IGF2	Insulin Like Growth Factor 2	Protein Coding	55	GC11M004506	8.617035866	https://www.genecards.org/cgi-bin/carddisp.pl?gene=IGF2
NAT1	N-Acetyltransferase 1	Protein Coding	50	GC08P018183	8.583248138	https://www.genecards.org/cgi-bin/carddisp.pl?gene=NAT1
CYB5A	Cytochrome B5 Type A	Protein Coding	50	GC18M074250	8.580019951	https://www.genecards.org/cgi-bin/carddisp.pl?gene=CYB5A
SOD2	Superoxide Dismutase 2	Protein Coding	53	GC06M159669	8.579874039	https://www.genecards.org/cgi-bin/carddisp.pl?gene=SOD2
TLR4	Toll Like Receptor 4	Protein Coding	58	GC09P117704	8.57736969	https://www.genecards.org/cgi-bin/carddisp.pl?gene=TLR4
AGK	Acylglycerol Kinase	Protein Coding	47	GC07P141551	8.569777489	https://www.genecards.org/cgi-bin/carddisp.pl?gene=AGK
AGPAT2	1-Acylglycerol-3-Phosphate O-Acyltransferase 2	Protein Coding	52	GC09M136673	8.565602303	https://www.genecards.org/cgi-bin/carddisp.pl?gene=AGPAT2
SRD5A1	Steroid 5 Alpha-Reductase 1	Protein Coding	50	GC05P006633	8.563120842	https://www.genecards.org/cgi-bin/carddisp.pl?gene=SRD5A1
FBN1	Fibrillin 1	Protein Coding	53	GC15M048408	8.536642075	https://www.genecards.org/cgi-bin/carddisp.pl?gene=FBN1
IL5	Interleukin 5	Protein Coding	52	GC05M132541	8.535798073	https://www.genecards.org/cgi-bin/carddisp.pl?gene=IL5
GLS	Glutaminase	Protein Coding	56	GC02P190880	8.528938293	https://www.genecards.org/cgi-bin/carddisp.pl?gene=GLS
EDN1	Endothelin 1	Protein Coding	55	GC06P012256	8.526630402	https://www.genecards.org/cgi-bin/carddisp.pl?gene=EDN1
GALC	Galactosylceramidase	Protein Coding	51	GC14M087837	8.512210846	https://www.genecards.org/cgi-bin/carddisp.pl?gene=GALC
MMP9	Matrix Metallopeptidase 9	Protein Coding	61	GC20P046008	8.467338562	https://www.genecards.org/cgi-bin/carddisp.pl?ge

						ne=MMP9
SPP1	Secreted Phosphoprotein 1	Protein Coding	53	GC04P087975	8.406263351	https://www.genecards.org/cgi-bin/carddisp.pl?gene=SPP1
SOAT1	Sterol O-Acyltransferase 1	Protein Coding	51	GC01P179262	8.406112671	https://www.genecards.org/cgi-bin/carddisp.pl?gene=SOAT1
FGFR1	Fibroblast Growth Factor Receptor 1	Protein Coding	63	GC08M038400	8.392232895	https://www.genecards.org/cgi-bin/carddisp.pl?gene=FGFR1
MME	Membrane Metalloendopeptidase	Protein Coding	57	GC03P155024	8.376724243	https://www.genecards.org/cgi-bin/carddisp.pl?gene=MME
FOXO3	Forkhead Box O3	Protein Coding	53	GC06P108559	8.376056671	https://www.genecards.org/cgi-bin/carddisp.pl?gene=FOXO3
GLUL	Glutamate-Ammonia Ligase	Protein Coding	55	GC01M182378	8.333607674	https://www.genecards.org/cgi-bin/carddisp.pl?gene=GLUL
CA2	Carbonic Anhydrase 2	Protein Coding	59	GC08P085463	8.320440292	https://www.genecards.org/cgi-bin/carddisp.pl?gene=CA2
GLA	Galactosidase Alpha	Protein Coding	56	GC0XM101393	8.318837166	https://www.genecards.org/cgi-bin/carddisp.pl?gene=GLA
ALAD	Aminolevulinate Dehydratase	Protein Coding	53	GC09M113386	8.308020592	https://www.genecards.org/cgi-bin/carddisp.pl?gene=ALAD
UGT2B15	UDP Glucuronosyltransferase Family 2 Member B15	Protein Coding	50	GC04M068646	8.296926498	https://www.genecards.org/cgi-bin/carddisp.pl?gene=UGT2B15
ACOX1	Acyl-CoA Oxidase 1	Protein Coding	52	GC17M075941	8.251636505	https://www.genecards.org/cgi-bin/carddisp.pl?gene=ACOX1
AKR1C2	Aldo-Keto Reductase Family 1 Member C2	Protein Coding	52	GC10M004987	8.24772644	https://www.genecards.org/cgi-bin/carddisp.pl?gene=AKR1C2
IGF2R	Insulin Like Growth Factor 2 Receptor	Protein Coding	52	GC06P159969	8.232765198	https://www.genecards.org/cgi-bin/carddisp.pl?gene=IGF2R
LPA	Lipoprotein(A)	Protein Coding	44	GC06M160531	8.226680756	https://www.genecards.org/cgi-bin/carddisp.pl?gene=LPA
AKR1B1	Aldo-Keto Reductase Family 1 Member B	Protein Coding	53	GC07M134442	8.226289749	https://www.genecards.org/cgi-bin/carddisp.pl?ge

						ne=AKR1B1
CLOCK	Clock Circadian Regulator	Protein Coding	51	GC04M055427	8.168092728	https://www.genecards.org/cgi-bin/carddisp.pl?gene=CLOCK
GPX1	Glutathione Peroxidase 1	Protein Coding	52	GC03M051566	8.151756287	https://www.genecards.org/cgi-bin/carddisp.pl?gene=GPX1
MIR22	MicroRNA 22	RNA Gene	23	GC17M001713	8.137874603	https://www.genecards.org/cgi-bin/carddisp.pl?gene=MIR22
ALDH1A1	Aldehyde Dehydrogenase 1 Family Member A1	Protein Coding	54	GC09M072900	8.133259773	https://www.genecards.org/cgi-bin/carddisp.pl?gene=ALDH1A1
COG2	Component Of Oligomeric Golgi Complex 2	Protein Coding	47	GC01P230642	8.130456924	https://www.genecards.org/cgi-bin/carddisp.pl?gene=COG2
UROD	Uroporphyrinogen Decarboxylase	Protein Coding	52	GC01P045458	8.115438461	https://www.genecards.org/cgi-bin/carddisp.pl?gene=UROD
BRCA1	BRCA1 DNA Repair Associated	Protein Coding	56	GC17M043044	8.113157272	https://www.genecards.org/cgi-bin/carddisp.pl?gene=BRCA1
CLCNKA	Chloride Voltage-Gated Channel Ka	Protein Coding	47	GC01P016018	8.101045609	https://www.genecards.org/cgi-bin/carddisp.pl?gene=CLCNKA
EPM2A	EPM2A Glucan Phosphatase, Laforin	Protein Coding	50	GC06M145382	8.040835381	https://www.genecards.org/cgi-bin/carddisp.pl?gene=EPM2A
JUN	Jun Proto-Oncogene, AP-1 Transcription Factor Subunit	Protein Coding	55	GC01M058780	8.033901215	https://www.genecards.org/cgi-bin/carddisp.pl?gene=JUN
SLC52A2	Solute Carrier Family 52 Member 2	Protein Coding	46	GC08P144333	8.015874863	https://www.genecards.org/cgi-bin/carddisp.pl?gene=SLC52A2
AHSG	Alpha 2-HS Glycoprotein	Protein Coding	51	GC03P186636	8.000261307	https://www.genecards.org/cgi-bin/carddisp.pl?gene=AHSG
FFAR3	Free Fatty Acid Receptor 3	Protein Coding	44	GC19P073871	7.985510826	https://www.genecards.org/cgi-bin/carddisp.pl?gene=FFAR3
DSP	Desmoplakin	Protein Coding	57	GC06P007541	7.967627048	https://www.genecards.org/cgi-bin/carddisp.pl?gene=DSP
TNFRSF11	TNF Receptor Superfamily Member 11b	Protein Coding	54	GC08M118923	7.947682381	https://www.genecards.org/cgi-bin/carddisp.pl?ge

B						ne=TNFRSF11B
ACO1	Aconitase 1	Protein Coding	51	GC09P032374	7.935599327	https://www.genecards.org/cgi-bin/carddisp.pl?gene=ACO1
APC	APC Regulator Of WNT Signaling Pathway	Protein Coding	56	GC05P112707	7.932646751	https://www.genecards.org/cgi-bin/carddisp.pl?gene=APC
CASP8	Caspase 8	Protein Coding	59	GC02P201233	7.92381525	https://www.genecards.org/cgi-bin/carddisp.pl?gene=CASP8
GHR	Growth Hormone Receptor	Protein Coding	53	GC05P042429	7.920368671	https://www.genecards.org/cgi-bin/carddisp.pl?gene=GHR
FGFR4	Fibroblast Growth Factor Receptor 4	Protein Coding	59	GC05P177086	7.900533676	https://www.genecards.org/cgi-bin/carddisp.pl?gene=FGFR4
LBR	Lamin B Receptor	Protein Coding	54	GC01M225401	7.891428947	https://www.genecards.org/cgi-bin/carddisp.pl?gene=LBR
TDO2	Tryptophan 2,3-Dioxygenase	Protein Coding	53	GC04P155854	7.886833191	https://www.genecards.org/cgi-bin/carddisp.pl?gene=TDO2
NOS2	Nitric Oxide Synthase 2	Protein Coding	56	GC17M027756	7.879232407	https://www.genecards.org/cgi-bin/carddisp.pl?gene=NOS2
DBH	Dopamine Beta-Hydroxylase	Protein Coding	59	GC09P133636	7.875431061	https://www.genecards.org/cgi-bin/carddisp.pl?gene=DBH
GUSB	Glucuronidase Beta	Protein Coding	54	GC07M065960	7.864967823	https://www.genecards.org/cgi-bin/carddisp.pl?gene=GUSB
GRIA4	Glutamate Ionotropic Receptor AMPA Type Subunit 4	Protein Coding	56	GC11P105609	7.864396095	https://www.genecards.org/cgi-bin/carddisp.pl?gene=GRIA4
PKLR	Pyruvate Kinase L/R	Protein Coding	53	GC01M155289	7.860248566	https://www.genecards.org/cgi-bin/carddisp.pl?gene=PKLR
TKT	Transketolase	Protein Coding	52	GC03M053224	7.831721306	https://www.genecards.org/cgi-bin/carddisp.pl?gene=TKT
TSPO	Translocator Protein	Protein Coding	51	GC22P043151	7.831576824	https://www.genecards.org/cgi-bin/carddisp.pl?gene=TSPO
ELANE	Elastase, Neutrophil Expressed	Protein Coding	58	GC19P003656	7.827675819	https://www.genecards.org/cgi-bin/carddisp.pl?ge

						ne=ELANE
ACSM3	Acyl-CoA Synthetase Medium Chain Family Member 3	Protein Coding	46	GC16P020610	7.801516533	https://www.genecards.org/cgi-bin/carddisp.pl?gene=ACSM3
FTH1	Ferritin Heavy Chain 1	Protein Coding	57	GC11M061959	7.793233395	https://www.genecards.org/cgi-bin/carddisp.pl?gene=FTH1
SNCA	Synuclein Alpha	Protein Coding	59	GC04M089724	7.786792278	https://www.genecards.org/cgi-bin/carddisp.pl?gene=SNCA
ATM	ATM Serine/Threonine Kinase	Protein Coding	60	GC11P108222	7.77983284	https://www.genecards.org/cgi-bin/carddisp.pl?gene=ATM
APOA4	Apolipoprotein A4	Protein Coding	48	GC11M116820	7.776916027	https://www.genecards.org/cgi-bin/carddisp.pl?gene=APOA4
PIK3R1	Phosphoinositide-3-Kinase Regulatory Subunit 1	Protein Coding	57	GC05P068215	7.765260696	https://www.genecards.org/cgi-bin/carddisp.pl?gene=PIK3R1
DNMT1	DNA Methyltransferase 1	Protein Coding	59	GC19M010133	7.755319595	https://www.genecards.org/cgi-bin/carddisp.pl?gene=DNMT1
SUCLA2	Succinate-CoA Ligase ADP-Forming Subunit Beta	Protein Coding	53	GC13M047745	7.749754429	https://www.genecards.org/cgi-bin/carddisp.pl?gene=SUCLA2
ICAM1	Intercellular Adhesion Molecule 1	Protein Coding	57	GC19P010749	7.744787693	https://www.genecards.org/cgi-bin/carddisp.pl?gene=ICAM1
MEN1	Menin 1	Protein Coding	51	GC11M064803	7.738993168	https://www.genecards.org/cgi-bin/carddisp.pl?gene=MEN1
FTL	Ferritin Light Chain	Protein Coding	52	GC19P048965	7.7378335	https://www.genecards.org/cgi-bin/carddisp.pl?gene=FTL
OXA1L	OXA1L Mitochondrial Inner Membrane Protein	Protein Coding	45	GC14P022766	7.737726212	https://www.genecards.org/cgi-bin/carddisp.pl?gene=OXA1L
TPH1	Tryptophan Hydroxylase 1	Protein Coding	52	GC11M018040	7.725982666	https://www.genecards.org/cgi-bin/carddisp.pl?gene=TPH1
COX4I1	Cytochrome C Oxidase Subunit 4I1	Protein Coding	52	GC16P085798	7.713577747	https://www.genecards.org/cgi-bin/carddisp.pl?gene=COX4I1
SLC9A1	Solute Carrier Family 9 Member A1	Protein Coding	57	GC01M027239	7.711806297	https://www.genecards.org/cgi-bin/carddisp.pl?ge

						ne=SLC9A1
GCH1	GTP Cyclohydrolase 1	Protein Coding	53	GC14M054842	7.711480618	https://www.genecards.org/cgi-bin/carddisp.pl?gene=GCH1
AKR1C1	Aldo-Keto Reductase Family 1 Member C1	Protein Coding	50	GC10P004963	7.708277702	https://www.genecards.org/cgi-bin/carddisp.pl?gene=AKR1C1
F12	Coagulation Factor XII	Protein Coding	56	GC05M177402	7.694033146	https://www.genecards.org/cgi-bin/carddisp.pl?gene=F12
ABHD5	Abhydrolase Domain Containing 5, Lysophosphatidic Acid Acyltransferase	Protein Coding	51	GC03P043707	7.663006783	https://www.genecards.org/cgi-bin/carddisp.pl?gene=ABHD5
AMN	Amnion Associated Transmembrane Protein	Protein Coding	47	GC14P102922	7.659276962	https://www.genecards.org/cgi-bin/carddisp.pl?gene=AMN
NR0B2	Nuclear Receptor Subfamily 0 Group B Member 2	Protein Coding	50	GC01M027236	7.647449017	https://www.genecards.org/cgi-bin/carddisp.pl?gene=NR0B2
CRAT	Carnitine O-Acetyltransferase	Protein Coding	51	GC09M129094	7.64017868	https://www.genecards.org/cgi-bin/carddisp.pl?gene=CRAT
ALDH1B1	Aldehyde Dehydrogenase 1 Family Member B1	Protein Coding	50	GC09P038392	7.639844418	https://www.genecards.org/cgi-bin/carddisp.pl?gene=ALDH1B1
AFP	Alpha Fetoprotein	Protein Coding	53	GC04P073431	7.626152992	https://www.genecards.org/cgi-bin/carddisp.pl?gene=AFP
UGT1A5	UDP Glucuronosyltransferase Family 1 Member A5	Protein Coding	40	GC02P233712	7.624723434	https://www.genecards.org/cgi-bin/carddisp.pl?gene=UGT1A5
CTNNB1	Catenin Beta 1	Protein Coding	60	GC03P041194	7.623961449	https://www.genecards.org/cgi-bin/carddisp.pl?gene=CTNNB1
SLC5A8	Solute Carrier Family 5 Member 8	Protein Coding	49	GC12M101155	7.623325348	https://www.genecards.org/cgi-bin/carddisp.pl?gene=SLC5A8
AKR1C4	Aldo-Keto Reductase Family 1 Member C4	Protein Coding	53	GC10P005195	7.621516705	https://www.genecards.org/cgi-bin/carddisp.pl?gene=AKR1C4
ACP5	Acid Phosphatase 5, Tartrate Resistant	Protein Coding	53	GC19M011574	7.61563921	https://www.genecards.org/cgi-bin/carddisp.pl?gene=ACP5
CCND1	Cyclin D1	Protein Coding	60	GC11P069641	7.614154339	https://www.genecards.org/cgi-bin/carddisp.pl?ge

						ne=CCND1
KYAT1	Kynurenine Aminotransferase 1	Protein Coding	47	GC09M128833	7.60359478	https://www.genecards.org/cgi-bin/carddisp.pl?gene=KYAT1
NOS1	Nitric Oxide Synthase 1	Protein Coding	56	GC12M117208	7.60078907	https://www.genecards.org/cgi-bin/carddisp.pl?gene=NOS1
SULT1A3	Sulfotransferase Family 1A Member 3	Protein Coding	43	GC16P030199	7.598351002	https://www.genecards.org/cgi-bin/carddisp.pl?gene=SULT1A3
CYCS	Cytochrome C, Somatic	Protein Coding	55	GC07M025118	7.579985619	https://www.genecards.org/cgi-bin/carddisp.pl?gene=CYCS
ACLY	ATP Citrate Lyase	Protein Coding	53	GC17M041866	7.559963703	https://www.genecards.org/cgi-bin/carddisp.pl?gene=ACLY
CDKN3	Cyclin Dependent Kinase Inhibitor 3	Protein Coding	48	GC14P054398	7.558909893	https://www.genecards.org/cgi-bin/carddisp.pl?gene=CDKN3
JAK2	Janus Kinase 2	Protein Coding	60	GC09P004985	7.554831028	https://www.genecards.org/cgi-bin/carddisp.pl?gene=JAK2
STAT3	Signal Transducer And Activator Of Transcription 3	Protein Coding	61	GC17M042313	7.553854942	https://www.genecards.org/cgi-bin/carddisp.pl?gene=STAT3
HCRT	Hypocretin Neuropeptide Precursor	Protein Coding	46	GC17M051782	7.507509232	https://www.genecards.org/cgi-bin/carddisp.pl?gene=HCRT
SQSTM1	Sequestosome 1	Protein Coding	55	GC05P179806	7.495340824	https://www.genecards.org/cgi-bin/carddisp.pl?gene=SQSTM1
DBT	Dihydrolipoamide Branched Chain Transacylase E2	Protein Coding	49	GC01M100186	7.495174885	https://www.genecards.org/cgi-bin/carddisp.pl?gene=DBT
TNFRSF11A	TNF Receptor Superfamily Member 11a	Protein Coding	54	GC18P062325	7.492922783	https://www.genecards.org/cgi-bin/carddisp.pl?gene=TNFRSF11A
PHYH	Phytanoyl-CoA 2-Hydroxylase	Protein Coding	52	GC10M013277	7.48562479	https://www.genecards.org/cgi-bin/carddisp.pl?gene=PHYH
PYY	Peptide YY	Protein Coding	49	GC17M043952	7.484090805	https://www.genecards.org/cgi-bin/carddisp.pl?gene=PYY
VLDLR	Very Low Density Lipoprotein Receptor	Protein Coding	56	GC09P002611	7.477643013	https://www.genecards.org/cgi-bin/carddisp.pl?ge

						ne=VLDLR
CSF2	Colony Stimulating Factor 2	Protein Coding	51	GC05P132073	7.462800026	https://www.genecards.org/cgi-bin/carddisp.pl?gene=CSF2
FOXO1	Forkhead Box O1	Protein Coding	56	GC13M040555	7.41369772	https://www.genecards.org/cgi-bin/carddisp.pl?gene=FOXO1
TTPA	Alpha Tocopherol Transfer Protein	Protein Coding	47	GC08M063048	7.413542747	https://www.genecards.org/cgi-bin/carddisp.pl?gene=TTPA
LTC4S	Leukotriene C4 Synthase	Protein Coding	49	GC05P179793	7.400671959	https://www.genecards.org/cgi-bin/carddisp.pl?gene=LTC4S
GSTZ1	Glutathione S-Transferase Zeta 1	Protein Coding	48	GC14P077320	7.394896507	https://www.genecards.org/cgi-bin/carddisp.pl?gene=GSTZ1
ACACB	Acetyl-CoA Carboxylase Beta	Protein Coding	52	GC12P109116	7.383708	https://www.genecards.org/cgi-bin/carddisp.pl?gene=ACACB
CRYAA	Crystallin Alpha A	Protein Coding	52	GC21P043169	7.340756893	https://www.genecards.org/cgi-bin/carddisp.pl?gene=CRYAA
SLC10A1	Solute Carrier Family 10 Member 1	Protein Coding	51	GC14M069775	7.333871365	https://www.genecards.org/cgi-bin/carddisp.pl?gene=SLC10A1
OPLAH	5-Oxoprolinase, ATP-Hydrolysing	Protein Coding	50	GC08M144278	7.333218098	https://www.genecards.org/cgi-bin/carddisp.pl?gene=OPLAH
IGF2BP2	Insulin Like Growth Factor 2 MRNA Binding Protein 2	Protein Coding	51	GC03M185643	7.331263065	https://www.genecards.org/cgi-bin/carddisp.pl?gene=IGF2BP2
CD320	CD320 Molecule	Protein Coding	47	GC19M008302	7.313445091	https://www.genecards.org/cgi-bin/carddisp.pl?gene=CD320
PTPN11	Protein Tyrosine Phosphatase Non-Receptor Type 11	Protein Coding	61	GC12P112418	7.310684204	https://www.genecards.org/cgi-bin/carddisp.pl?gene=PTPN11
HLA-B	Major Histocompatibility Complex, Class I, B	Protein Coding	52	GC06M071682	7.308247089	https://www.genecards.org/cgi-bin/carddisp.pl?gene=HLA-B
CCK	Cholecystokinin	Protein Coding	48	GC03M042274	7.304950714	https://www.genecards.org/cgi-bin/carddisp.pl?gene=CCK
TBXAS1	Thromboxane A Synthase 1	Protein Coding	56	GC07P139777	7.303420544	https://www.genecards.org/cgi-bin/carddisp.pl?ge

						ne=TBXAS1
ACY1	Aminoacylase 1	Protein Coding	52	GC03P051983	7.282396793	https://www.genecards.org/cgi-bin/carddisp.pl?gene=ACY1
CYP4V2	Cytochrome P450 Family 4 Subfamily V Member 2	Protein Coding	48	GC04P186191	7.213627338	https://www.genecards.org/cgi-bin/carddisp.pl?gene=CYP4V2
KCNJ5	Potassium Inwardly Rectifying Channel Subfamily J Member 5	Protein Coding	53	GC11P128891	7.193699837	https://www.genecards.org/cgi-bin/carddisp.pl?gene=KCNJ5
MIR29C	MicroRNA 29c	RNA Gene	21	GC01M207811	7.183753967	https://www.genecards.org/cgi-bin/carddisp.pl?gene=MIR29C
SLC2A3	Solute Carrier Family 2 Member 3	Protein Coding	55	GC12M007919	7.150427818	https://www.genecards.org/cgi-bin/carddisp.pl?gene=SLC2A3
CBR1	Carbonyl Reductase 1	Protein Coding	51	GC21P036069	7.138096809	https://www.genecards.org/cgi-bin/carddisp.pl?gene=CBR1
IL2	Interleukin 2	Protein Coding	53	GC04M122451	7.137097359	https://www.genecards.org/cgi-bin/carddisp.pl?gene=IL2
SLC6A3	Solute Carrier Family 6 Member 3	Protein Coding	57	GC05M001392	7.135263443	https://www.genecards.org/cgi-bin/carddisp.pl?gene=SLC6A3
PLA2G1B	Phospholipase A2 Group IB	Protein Coding	51	GC12M120322	7.106545925	https://www.genecards.org/cgi-bin/carddisp.pl?gene=PLA2G1B
RORA	RAR Related Orphan Receptor A	Protein Coding	55	GC15M060488	7.092875957	https://www.genecards.org/cgi-bin/carddisp.pl?gene=RORA
SULT2A1	Sulfotransferase Family 2A Member 1	Protein Coding	50	GC19M047870	7.092458248	https://www.genecards.org/cgi-bin/carddisp.pl?gene=SULT2A1
IL13	Interleukin 13	Protein Coding	50	GC05P132656	7.088688374	https://www.genecards.org/cgi-bin/carddisp.pl?gene=IL13
PGK1	Phosphoglycerate Kinase 1	Protein Coding	56	GC0XP078032	7.080644131	https://www.genecards.org/cgi-bin/carddisp.pl?gene=PGK1
CD4	CD4 Molecule	Protein Coding	57	GC12P006786	7.079792976	https://www.genecards.org/cgi-bin/carddisp.pl?gene=CD4
TYR	Tyrosinase	Protein Coding	55	GC11P089177	7.075667381	https://www.genecards.org/cgi-bin/carddisp.pl?ge

						ne=TYR
FAAH	Fatty Acid Amide Hydrolase	Protein Coding	54	GC01P046394	7.074492455	https://www.genecards.org/cgi-bin/carddisp.pl?gene=FAAH
CCL5	C-C Motif Chemokine Ligand 5	Protein Coding	51	GC17M035871	7.05864048	https://www.genecards.org/cgi-bin/carddisp.pl?gene=CCL5
HMBS	Hydroxymethylbilane Synthase	Protein Coding	51	GC11P119084	7.051350594	https://www.genecards.org/cgi-bin/carddisp.pl?gene=HMBS
KCNJ10	Potassium Inwardly Rectifying Channel Subfamily J Member 10	Protein Coding	52	GC01M159998	7.050767422	https://www.genecards.org/cgi-bin/carddisp.pl?gene=KCNJ10
CACNA1S	Calcium Voltage-Gated Channel Subunit Alpha1 S	Protein Coding	55	GC01M201008	7.044938564	https://www.genecards.org/cgi-bin/carddisp.pl?gene=CACNA1S
EIF2AK3	Eukaryotic Translation Initiation Factor 2 Alpha Kinase 3	Protein Coding	55	GC02M088556	7.033459187	https://www.genecards.org/cgi-bin/carddisp.pl?gene=EIF2AK3
ACSL4	Acyl-CoA Synthetase Long Chain Family Member 4	Protein Coding	52	GC0XM109624	7.020869732	https://www.genecards.org/cgi-bin/carddisp.pl?gene=ACSL4
PTS	6-Pyruvoyltetrahydropterin Synthase	Protein Coding	51	GC11P112226	6.987995625	https://www.genecards.org/cgi-bin/carddisp.pl?gene=PTS
ACTB	Actin Beta	Protein Coding	56	GC07M005527	6.978267193	https://www.genecards.org/cgi-bin/carddisp.pl?gene=ACTB
FBP2	Fructose-Bisphosphatase 2	Protein Coding	50	GC09M094558	6.97452116	https://www.genecards.org/cgi-bin/carddisp.pl?gene=FBP2
SULT1A2	Sulfotransferase Family 1A Member 2	Protein Coding	48	GC16M028591	6.969522476	https://www.genecards.org/cgi-bin/carddisp.pl?gene=SULT1A2
FXN	Frataxin	Protein Coding	52	GC09P069035	6.963536739	https://www.genecards.org/cgi-bin/carddisp.pl?gene=FXN
FGFR3	Fibroblast Growth Factor Receptor 3	Protein Coding	63	GC04P001795	6.953905582	https://www.genecards.org/cgi-bin/carddisp.pl?gene=FGFR3
ALAS2	5'-Aminolevulinate Synthase 2	Protein Coding	51	GC0XM055009	6.94052887	https://www.genecards.org/cgi-bin/carddisp.pl?gene=ALAS2
SULT1E1	Sulfotransferase Family 1E Member 1	Protein Coding	50	GC04M069823	6.935936451	https://www.genecards.org/cgi-bin/carddisp.pl?ge

						ne=SULT1E1
GOT1	Glutamic-Oxaloacetic Transaminase 1	Protein Coding	52	GC10M099396	6.932769299	https://www.genecards.org/cgi-bin/carddisp.pl?gene=GOT1
FTCD-AS1	FTCD Antisense RNA 1	RNA Gene	18	GC21P046151	6.917957783	https://www.genecards.org/cgi-bin/carddisp.pl?gene=FTCD-AS1
PARP1	Poly(ADP-Ribose) Polymerase 1	Protein Coding	58	GC01M226360	6.91354847	https://www.genecards.org/cgi-bin/carddisp.pl?gene=PARP1
TARDBP	TAR DNA Binding Protein	Protein Coding	53	GC01P011013	6.906384468	https://www.genecards.org/cgi-bin/carddisp.pl?gene=TARDBP
IL1A	Interleukin 1 Alpha	Protein Coding	52	GC02M112773	6.887263298	https://www.genecards.org/cgi-bin/carddisp.pl?gene=IL1A
HPGD	15-Hydroxyprostaglandin Dehydrogenase	Protein Coding	53	GC04M174490	6.878279209	https://www.genecards.org/cgi-bin/carddisp.pl?gene=HPGD
CCR6	C-C Motif Chemokine Receptor 6	Protein Coding	51	GC06P167111	6.876591682	https://www.genecards.org/cgi-bin/carddisp.pl?gene=CCR6
CSN1S1	Casein Alpha S1	Protein Coding	43	GC04P069932	6.874129295	https://www.genecards.org/cgi-bin/carddisp.pl?gene=CSN1S1
APOC1	Apolipoprotein C1	Protein Coding	46	GC19P044914	6.873025417	https://www.genecards.org/cgi-bin/carddisp.pl?gene=APOC1
IDO1	Indoleamine 2,3-Dioxygenase 1	Protein Coding	53	GC08P039891	6.860894203	https://www.genecards.org/cgi-bin/carddisp.pl?gene=IDO1
FOS	Fos Proto-Oncogene, AP-1 Transcription Factor Subunit	Protein Coding	57	GC14P075278	6.856826782	https://www.genecards.org/cgi-bin/carddisp.pl?gene=FOS
PIK3CG	Phosphatidylinositol-4,5-Bisphosphate 3-Kinase Catalytic Subunit Gamma	Protein Coding	55	GC07P106865	6.855944633	https://www.genecards.org/cgi-bin/carddisp.pl?gene=PIK3CG
ADORA2A	Adenosine A2a Receptor	Protein Coding	53	GC22P024417	6.848574162	https://www.genecards.org/cgi-bin/carddisp.pl?gene=ADORA2A
MAPK14	Mitogen-Activated Protein Kinase 14	Protein Coding	58	GC06P092286	6.802408218	https://www.genecards.org/cgi-bin/carddisp.pl?gene=MAPK14
GUCY2C	Guanylate Cyclase 2C	Protein Coding	52	GC12M014612	6.80224371	https://www.genecards.org/cgi-bin/carddisp.pl?ge

						ne=GUCY2C
MAPK10	Mitogen-Activated Protein Kinase 10	Protein Coding	56	GC04M085990	6.799727917	https://www.genecards.org/cgi-bin/carddisp.pl?gene=MAPK10
AGT	Angiotensinogen	Protein Coding	56	GC01M230690	6.794569969	https://www.genecards.org/cgi-bin/carddisp.pl?gene=AGT
IL18	Interleukin 18	Protein Coding	50	GC11M112143	6.792854786	https://www.genecards.org/cgi-bin/carddisp.pl?gene=IL18
BPGM	Bisphosphoglycerate Mutase	Protein Coding	52	GC07P134646	6.783220291	https://www.genecards.org/cgi-bin/carddisp.pl?gene=BPGM
SLPI	Secretory Leukocyte Peptidase Inhibitor	Protein Coding	47	GC20M045252	6.7794981	https://www.genecards.org/cgi-bin/carddisp.pl?gene=SLPI
LIAS	Lipoic Acid Synthetase	Protein Coding	48	GC04P039499	6.778037071	https://www.genecards.org/cgi-bin/carddisp.pl?gene=LIAS
AMT	Aminomethyltransferase	Protein Coding	51	GC03M051568	6.773910046	https://www.genecards.org/cgi-bin/carddisp.pl?gene=AMT
GPI	Glucose-6-Phosphate Isomerase	Protein Coding	55	GC19P034359	6.765838146	https://www.genecards.org/cgi-bin/carddisp.pl?gene=GPI
MAPK8	Mitogen-Activated Protein Kinase 8	Protein Coding	57	GC10P048306	6.759390831	https://www.genecards.org/cgi-bin/carddisp.pl?gene=MAPK8
BRAF	B-Raf Proto-Oncogene, Serine/Threonine Kinase	Protein Coding	60	GC07M140742	6.748792171	https://www.genecards.org/cgi-bin/carddisp.pl?gene=BRAF
YARS2	Tyrosyl-TRNA Synthetase 2	Protein Coding	51	GC12M032819	6.745001793	https://www.genecards.org/cgi-bin/carddisp.pl?gene=YARS2
RNASE3	Ribonuclease A Family Member 3	Protein Coding	46	GC14P020891	6.725999355	https://www.genecards.org/cgi-bin/carddisp.pl?gene=RNASE3
GSK3B	Glycogen Synthase Kinase 3 Beta	Protein Coding	57	GC03M119821	6.701512337	https://www.genecards.org/cgi-bin/carddisp.pl?gene=GSK3B
BMP2	Bone Morphogenetic Protein 2	Protein Coding	53	GC20P006767	6.691226959	https://www.genecards.org/cgi-bin/carddisp.pl?gene=BMP2
MMP3	Matrix Metallopeptidase 3	Protein Coding	57	GC11M102835	6.690543175	https://www.genecards.org/cgi-bin/carddisp.pl?ge

						ne=MMP3
FABP1	Fatty Acid Binding Protein 1	Protein Coding	49	GC02M088122	6.688127518	https://www.genecards.org/cgi-bin/carddisp.pl?gene=FABP1
ALDH3A1	Aldehyde Dehydrogenase 3 Family Member A1	Protein Coding	52	GC17M019737	6.668470383	https://www.genecards.org/cgi-bin/carddisp.pl?gene=ALDH3A1
HSPG2	Heparan Sulfate Proteoglycan 2	Protein Coding	54	GC01M021822	6.655029297	https://www.genecards.org/cgi-bin/carddisp.pl?gene=HSPG2
MMP2	Matrix Metallopeptidase 2	Protein Coding	60	GC16P055390	6.633100986	https://www.genecards.org/cgi-bin/carddisp.pl?gene=MMP2
ERCC1	ERCC Excision Repair 1, Endonuclease Non-Catalytic Subunit	Protein Coding	52	GC19M070729	6.630684376	https://www.genecards.org/cgi-bin/carddisp.pl?gene=ERCC1
CD38	CD38 Molecule	Protein Coding	53	GC04P019223	6.6181674	https://www.genecards.org/cgi-bin/carddisp.pl?gene=CD38
TIMP1	TIMP Metallopeptidase Inhibitor 1	Protein Coding	52	GC0XP047711	6.613132477	https://www.genecards.org/cgi-bin/carddisp.pl?gene=TIMP1
GMPS	Guanine Monophosphate Synthase	Protein Coding	51	GC03P155870	6.612122536	https://www.genecards.org/cgi-bin/carddisp.pl?gene=GMPS
INPPL1	Inositol Polyphosphate Phosphatase Like 1	Protein Coding	55	GC11P072223	6.6092906	https://www.genecards.org/cgi-bin/carddisp.pl?gene=INPPL1
TSC2	TSC Complex Subunit 2	Protein Coding	56	GC16P013513	6.607493401	https://www.genecards.org/cgi-bin/carddisp.pl?gene=TSC2
STS	Steroid Sulfatase	Protein Coding	52	GC0XP007146	6.60575819	https://www.genecards.org/cgi-bin/carddisp.pl?gene=STS
PAX4	Paired Box 4	Protein Coding	47	GC07M127610	6.605308533	https://www.genecards.org/cgi-bin/carddisp.pl?gene=PAX4
ALDH1A2	Aldehyde Dehydrogenase 1 Family Member A2	Protein Coding	55	GC15M121963	6.603302479	https://www.genecards.org/cgi-bin/carddisp.pl?gene=ALDH1A2
PTHLH	Parathyroid Hormone Like Hormone	Protein Coding	52	GC12M027959	6.598110199	https://www.genecards.org/cgi-bin/carddisp.pl?gene=PTHLH
BAAT	Bile Acid-CoA:Amino Acid N-Acyltransferase	Protein Coding	50	GC09M101354	6.590001106	https://www.genecards.org/cgi-bin/carddisp.pl?ge

						ne=BAAT
CHKA	Choline Kinase Alpha	Protein Coding	49	GC11M068052	6.575095654	https://www.genecards.org/cgi-bin/carddisp.pl?gene=CHKA
ELN	Elastin	Protein Coding	50	GC07P074027	6.564837456	https://www.genecards.org/cgi-bin/carddisp.pl?gene=ELN
WDR72	WD Repeat Domain 72	Protein Coding	43	GC15M125660	6.55697155	https://www.genecards.org/cgi-bin/carddisp.pl?gene=WDR72
HTT	Huntingtin	Protein Coding	51	GC04P003041	6.549586296	https://www.genecards.org/cgi-bin/carddisp.pl?gene=HTT
STAT1	Signal Transducer And Activator Of Transcription 1	Protein Coding	60	GC02M190908	6.545604706	https://www.genecards.org/cgi-bin/carddisp.pl?gene=STAT1
ALOX15	Arachidonate 15-Lipoxygenase	Protein Coding	52	GC17M004630	6.537812233	https://www.genecards.org/cgi-bin/carddisp.pl?gene=ALOX15
FFAR4	Free Fatty Acid Receptor 4	Protein Coding	47	GC10P093566	6.531475067	https://www.genecards.org/cgi-bin/carddisp.pl?gene=FFAR4
CSF1	Colony Stimulating Factor 1	Protein Coding	51	GC01P109911	6.528945923	https://www.genecards.org/cgi-bin/carddisp.pl?gene=CSF1
PRKN	Parkin RBR E3 Ubiquitin Protein Ligase	Protein Coding	53	GC06M161348	6.517373085	https://www.genecards.org/cgi-bin/carddisp.pl?gene=PRKN
SOAT2	Sterol O-Acyltransferase 2	Protein Coding	50	GC12P053103	6.513618469	https://www.genecards.org/cgi-bin/carddisp.pl?gene=SOAT2
NTS	Neurotensin	Protein Coding	48	GC12P085876	6.508972645	https://www.genecards.org/cgi-bin/carddisp.pl?gene=NTS
HSD17B1	Hydroxysteroid 17-Beta Dehydrogenase 1	Protein Coding	48	GC17P061989	6.502941608	https://www.genecards.org/cgi-bin/carddisp.pl?gene=HSD17B1
KNG1	Kininogen 1	Protein Coding	55	GC03P186717	6.496859074	https://www.genecards.org/cgi-bin/carddisp.pl?gene=KNG1
HBA1	Hemoglobin Subunit Alpha 1	Protein Coding	49	GC16P013428	6.478796959	https://www.genecards.org/cgi-bin/carddisp.pl?gene=HBA1
ESR2	Estrogen Receptor 2	Protein Coding	56	GC14M064084	6.464812279	https://www.genecards.org/cgi-bin/carddisp.pl?ge

						ne=ESR2
ERBB2	Erb-B2 Receptor Tyrosine Kinase 2	Protein Coding	62	GC17P039687	6.462425709	https://www.genecards.org/cgi-bin/carddisp.pl?gene=ERBB2
HP	Haptoglobin	Protein Coding	52	GC16P072372	6.458934307	https://www.genecards.org/cgi-bin/carddisp.pl?gene=HP
PTGIS	Prostaglandin I2 Synthase	Protein Coding	52	GC20M049503	6.456087112	https://www.genecards.org/cgi-bin/carddisp.pl?gene=PTGIS
ATF4	Activating Transcription Factor 4	Protein Coding	52	GC22P039519	6.442789555	https://www.genecards.org/cgi-bin/carddisp.pl?gene=ATF4
THBD	Thrombomodulin	Protein Coding	53	GC20M023026	6.435276985	https://www.genecards.org/cgi-bin/carddisp.pl?gene=THBD
DIO2	Iodothyronine Deiodinase 2	Protein Coding	46	GC14M080197	6.413453579	https://www.genecards.org/cgi-bin/carddisp.pl?gene=DIO2
PDK1	Pyruvate Dehydrogenase Kinase 1	Protein Coding	53	GC02P172555	6.402394295	https://www.genecards.org/cgi-bin/carddisp.pl?gene=PDK1
IFNB1	Interferon Beta 1	Protein Coding	50	GC09M021077	6.401349068	https://www.genecards.org/cgi-bin/carddisp.pl?gene=IFNB1
LRP1	LDL Receptor Related Protein 1	Protein Coding	53	GC12P057128	6.399954319	https://www.genecards.org/cgi-bin/carddisp.pl?gene=LRP1
VCP	Valosin Containing Protein	Protein Coding	57	GC09M035056	6.39056921	https://www.genecards.org/cgi-bin/carddisp.pl?gene=VCP
MOGS	Mannosyl-Oligosaccharide Glucosidase	Protein Coding	50	GC02M074461	6.386434078	https://www.genecards.org/cgi-bin/carddisp.pl?gene=MOGS
MIR3944	MicroRNA 3944	RNA Gene	15	GC10M133371	6.384291649	https://www.genecards.org/cgi-bin/carddisp.pl?gene=MIR3944
GPAM	Glycerol-3-Phosphate Acyltransferase, Mitochondrial	Protein Coding	50	GC10M112148	6.383892059	https://www.genecards.org/cgi-bin/carddisp.pl?gene=GPAM
PPT1	Palmitoyl-Protein Thioesterase 1	Protein Coding	53	GC01M040103	6.374232292	https://www.genecards.org/cgi-bin/carddisp.pl?gene=PPT1
CTSD	Cathepsin D	Protein Coding	59	GC11M001752	6.372982979	https://www.genecards.org/cgi-bin/carddisp.pl?ge

						ne=CTSD
HRAS	HRas Proto-Oncogene, GTPase	Protein Coding	59	GC11M004421	6.359884739	https://www.genecards.org/cgi-bin/carddisp.pl?gene=HRAS
PLAT	Plasminogen Activator, Tissue Type	Protein Coding	57	GC08M042174	6.354998589	https://www.genecards.org/cgi-bin/carddisp.pl?gene=PLAT
GRIA3	Glutamate Ionotropic Receptor AMPA Type Subunit 3	Protein Coding	58	GC0XP123184	6.331629753	https://www.genecards.org/cgi-bin/carddisp.pl?gene=GRIA3
AVP	Arginine Vasopressin	Protein Coding	51	GC20M003082	6.322710514	https://www.genecards.org/cgi-bin/carddisp.pl?gene=AVP
EGFR	Epidermal Growth Factor Receptor	Protein Coding	62	GC07P055019	6.309800625	https://www.genecards.org/cgi-bin/carddisp.pl?gene=EGFR
PLG	Plasminogen	Protein Coding	56	GC06P160702	6.293350697	https://www.genecards.org/cgi-bin/carddisp.pl?gene=PLG
ACHE	Acetylcholinesterase (Cartwright Blood Group)	Protein Coding	53	GC07M100889	6.264330864	https://www.genecards.org/cgi-bin/carddisp.pl?gene=ACHE
CASP3	Caspase 3	Protein Coding	57	GC04M184627	6.237726212	https://www.genecards.org/cgi-bin/carddisp.pl?gene=CASP3
ST3GAL4	ST3 Beta-Galactoside Alpha-2,3-Sialyltransferase 4	Protein Coding	50	GC11P126355	6.23571682	https://www.genecards.org/cgi-bin/carddisp.pl?gene=ST3GAL4
SLC30A8	Solute Carrier Family 30 Member 8	Protein Coding	48	GC08P116950	6.219080925	https://www.genecards.org/cgi-bin/carddisp.pl?gene=SLC30A8
DLAT	Dihydrolipoamide S-Acetyltransferase	Protein Coding	53	GC11P112026	6.217043877	https://www.genecards.org/cgi-bin/carddisp.pl?gene=DLAT
PDGFRB	Platelet Derived Growth Factor Receptor Beta	Protein Coding	60	GC05M150113	6.213683605	https://www.genecards.org/cgi-bin/carddisp.pl?gene=PDGFRB
GYG1	Glycogenin 1	Protein Coding	53	GC03P148991	6.206664085	https://www.genecards.org/cgi-bin/carddisp.pl?gene=GYG1
GPHN	Gephyrin	Protein Coding	55	GC14P066507	6.201871395	https://www.genecards.org/cgi-bin/carddisp.pl?gene=GPHN
ALDH3B1	Aldehyde Dehydrogenase 3 Family Member B1	Protein Coding	47	GC11P071077	6.159162521	https://www.genecards.org/cgi-bin/carddisp.pl?ge

						ne=ALDH3B1
PIEZO2	Piezo Type Mechanosensitive Ion Channel Component 2	Protein Coding	43	GC18M010670	6.156918049	https://www.genecards.org/cgi-bin/carddisp.pl?gene=PIEZO2
HTR1A	5-Hydroxytryptamine Receptor 1A	Protein Coding	53	GC05M063960	6.154148102	https://www.genecards.org/cgi-bin/carddisp.pl?gene=HTR1A
UGT2B17	UDP Glucuronosyltransferase Family 2 Member B17	Protein Coding	47	GC04M068537	6.152544975	https://www.genecards.org/cgi-bin/carddisp.pl?gene=UGT2B17
RUNX2	RUNX Family Transcription Factor 2	Protein Coding	53	GC06P092371	6.151573658	https://www.genecards.org/cgi-bin/carddisp.pl?gene=RUNX2
ALOX5AP	Arachidonate 5-Lipoxygenase Activating Protein	Protein Coding	51	GC13P030713	6.141064167	https://www.genecards.org/cgi-bin/carddisp.pl?gene=ALOX5AP
PHGDH	Phosphoglycerate Dehydrogenase	Protein Coding	57	GC01P119660	6.127547741	https://www.genecards.org/cgi-bin/carddisp.pl?gene=PHGDH
WWOX	WW Domain Containing Oxidoreductase	Protein Coding	52	GC16P078099	6.116097927	https://www.genecards.org/cgi-bin/carddisp.pl?gene=WWOX
ALDH1A3	Aldehyde Dehydrogenase 1 Family Member A3	Protein Coding	52	GC15P100877	6.111147881	https://www.genecards.org/cgi-bin/carddisp.pl?gene=ALDH1A3
PLCB1	Phospholipase C Beta 1	Protein Coding	53	GC20P008061	6.108633518	https://www.genecards.org/cgi-bin/carddisp.pl?gene=PLCB1
HSD3B1	Hydroxy-Delta-5-Steroid Dehydrogenase, 3 Beta- And Steroid Delta-Isomerase 1	Protein Coding	49	GC01P119507	6.108185291	https://www.genecards.org/cgi-bin/carddisp.pl?gene=HSD3B1
HMGA1	High Mobility Group AT-Hook 1	Protein Coding	53	GC06P092259	6.101529121	https://www.genecards.org/cgi-bin/carddisp.pl?gene=HMGA1
ESRRA	Estrogen Related Receptor Alpha	Protein Coding	53	GC11P064305	6.093959808	https://www.genecards.org/cgi-bin/carddisp.pl?gene=ESRRA
FLT3	Fms Related Receptor Tyrosine Kinase 3	Protein Coding	60	GC13M028003	6.085923672	https://www.genecards.org/cgi-bin/carddisp.pl?gene=FLT3
CPT1B	Carnitine Palmitoyltransferase 1B	Protein Coding	51	GC22M061624	6.08046484	https://www.genecards.org/cgi-bin/carddisp.pl?gene=CPT1B
GFAP	Glial Fibrillary Acidic Protein	Protein Coding	55	GC17M051925	6.074894428	https://www.genecards.org/cgi-bin/carddisp.pl?ge

						ne=GFAP
CHAT	Choline O-Acetyltransferase	Protein Coding	55	GC10P049609	6.072665691	https://www.genecards.org/cgi-bin/carddisp.pl?gene=CHAT
DRD2	Dopamine Receptor D2	Protein Coding	56	GC11M113409	6.065560818	https://www.genecards.org/cgi-bin/carddisp.pl?gene=DRD2
ABCC3	ATP Binding Cassette Subfamily C Member 3	Protein Coding	53	GC17P050634	6.057796955	https://www.genecards.org/cgi-bin/carddisp.pl?gene=ABCC3
SLC7A5	Solute Carrier Family 7 Member 5	Protein Coding	51	GC16M087830	6.049738884	https://www.genecards.org/cgi-bin/carddisp.pl?gene=SLC7A5
NNMT	Nicotinamide N-Methyltransferase	Protein Coding	51	GC11P114257	6.028770924	https://www.genecards.org/cgi-bin/carddisp.pl?gene=NNMT
FABP5	Fatty Acid Binding Protein 5	Protein Coding	49	GC08P081282	6.027059078	https://www.genecards.org/cgi-bin/carddisp.pl?gene=FABP5
F9	Coagulation Factor IX	Protein Coding	54	GC0XP139530	6.001158714	https://www.genecards.org/cgi-bin/carddisp.pl?gene=F9
AGTR1	Angiotensin II Receptor Type 1	Protein Coding	58	GC03P148697	5.997434139	https://www.genecards.org/cgi-bin/carddisp.pl?gene=AGTR1
AIFM1	Apoptosis Inducing Factor Mitochondria Associated 1	Protein Coding	56	GC0XM130129	5.993622303	https://www.genecards.org/cgi-bin/carddisp.pl?gene=AIFM1
TG	Thyroglobulin	Protein Coding	51	GC08P132866	5.986973286	https://www.genecards.org/cgi-bin/carddisp.pl?gene=TG
LOX	Lysyl Oxidase	Protein Coding	54	GC05M122063	5.978069305	https://www.genecards.org/cgi-bin/carddisp.pl?gene=LOX
SCP2	Sterol Carrier Protein 2	Protein Coding	52	GC01P052927	5.971069813	https://www.genecards.org/cgi-bin/carddisp.pl?gene=SCP2
FOLH1	Folate Hydrolase 1	Protein Coding	54	GC11M099394	5.953541756	https://www.genecards.org/cgi-bin/carddisp.pl?gene=FOLH1
ANXA5	Annexin A5	Protein Coding	52	GC04M121667	5.934454441	https://www.genecards.org/cgi-bin/carddisp.pl?gene=ANXA5
F3	Coagulation Factor III, Tissue Factor	Protein Coding	52	GC01M094610	5.908566475	https://www.genecards.org/cgi-bin/carddisp.pl?ge

						ne=F3
CREB1	CAMP Responsive Element Binding Protein 1	Protein Coding	56	GC02P207529	5.901594639	https://www.genecards.org/cgi-bin/carddisp.pl?gene=CREB1
MAP2K1	Mitogen-Activated Protein Kinase Kinase 1	Protein Coding	60	GC15P066386	5.901299477	https://www.genecards.org/cgi-bin/carddisp.pl?gene=MAP2K1
HGSNAT	Heparan-Alpha-Glucosaminide N-Acetyltransferase	Protein Coding	43	GC08P043140	5.899586678	https://www.genecards.org/cgi-bin/carddisp.pl?gene=HGSNAT
CCL11	C-C Motif Chemokine Ligand 11	Protein Coding	52	GC17P034285	5.89659977	https://www.genecards.org/cgi-bin/carddisp.pl?gene=CCL11
SST	Somatostatin	Protein Coding	47	GC03M187668	5.892080307	https://www.genecards.org/cgi-bin/carddisp.pl?gene=SST
ABCB7	ATP Binding Cassette Subfamily B Member 7	Protein Coding	50	GC0XM075053	5.879004955	https://www.genecards.org/cgi-bin/carddisp.pl?gene=ABCB7
NFKBIA	NFKB Inhibitor Alpha	Protein Coding	57	GC14M035401	5.857986927	https://www.genecards.org/cgi-bin/carddisp.pl?gene=NFKBIA
ASNS	Asparagine Synthetase (Glutamine-Hydrolyzing)	Protein Coding	54	GC07M097854	5.841819763	https://www.genecards.org/cgi-bin/carddisp.pl?gene=ASNS
HNRNPA1	Heterogeneous Nuclear Ribonucleoprotein A1	Protein Coding	55	GC12P054280	5.838124752	https://www.genecards.org/cgi-bin/carddisp.pl?gene=HNRNPA1
ARSH	Arylsulfatase Family Member H	Protein Coding	39	GC0XP003006	5.814909458	https://www.genecards.org/cgi-bin/carddisp.pl?gene=ARSH
UGT2B4	UDP Glucuronosyltransferase Family 2 Member B4	Protein Coding	47	GC04M069484	5.801847935	https://www.genecards.org/cgi-bin/carddisp.pl?gene=UGT2B4
GAD1	Glutamate Decarboxylase 1	Protein Coding	56	GC02P170813	5.800939083	https://www.genecards.org/cgi-bin/carddisp.pl?gene=GAD1
GLO1	Glyoxalase I	Protein Coding	52	GC06M071882	5.783227921	https://www.genecards.org/cgi-bin/carddisp.pl?gene=GLO1
TPH2	Tryptophan Hydroxylase 2	Protein Coding	56	GC12P071938	5.7809062	https://www.genecards.org/cgi-bin/carddisp.pl?gene=TPH2
CTSA	Cathepsin A	Protein Coding	53	GC20P045890	5.778818607	https://www.genecards.org/cgi-bin/carddisp.pl?ge

						ne=CTSA
CRH	Corticotropin Releasing Hormone	Protein Coding	50	GC08M066176	5.773647785	https://www.genecards.org/cgi-bin/carddisp.pl?gene=CRH
EP300	E1A Binding Protein P300	Protein Coding	59	GC22P045272	5.772609711	https://www.genecards.org/cgi-bin/carddisp.pl?gene=EP300
FGF2	Fibroblast Growth Factor 2	Protein Coding	52	GC04P122826	5.771065235	https://www.genecards.org/cgi-bin/carddisp.pl?gene=FGF2
MGLL	Monoglyceride Lipase	Protein Coding	51	GC03M127689	5.765919685	https://www.genecards.org/cgi-bin/carddisp.pl?gene=MGLL
MAPK3	Mitogen-Activated Protein Kinase 3	Protein Coding	56	GC16M038440	5.753620148	https://www.genecards.org/cgi-bin/carddisp.pl?gene=MAPK3
ABCC6	ATP Binding Cassette Subfamily C Member 6	Protein Coding	53	GC16M016148	5.726374149	https://www.genecards.org/cgi-bin/carddisp.pl?gene=ABCC6
AGPAT1	1-Acylglycerol-3-Phosphate O-Acyltransferase 1	Protein Coding	47	GC06M032168	5.723912716	https://www.genecards.org/cgi-bin/carddisp.pl?gene=AGPAT1
DMD	Dystrophin	Protein Coding	52	GC0XM031097	5.719425678	https://www.genecards.org/cgi-bin/carddisp.pl?gene=DMD
SULT2B1	Sulfotransferase Family 2B Member 1	Protein Coding	52	GC19P048552	5.709708691	https://www.genecards.org/cgi-bin/carddisp.pl?gene=SULT2B1
MET	MET Proto-Oncogene, Receptor Tyrosine Kinase	Protein Coding	61	GC07P116672	5.706713676	https://www.genecards.org/cgi-bin/carddisp.pl?gene=MET
MAPT	Microtubule Associated Protein Tau	Protein Coding	57	GC17P045894	5.706644535	https://www.genecards.org/cgi-bin/carddisp.pl?gene=MAPT
PTPN22	Protein Tyrosine Phosphatase Non-Receptor Type 22	Protein Coding	54	GC01M113813	5.70640564	https://www.genecards.org/cgi-bin/carddisp.pl?gene=PTPN22
PGAM2	Phosphoglycerate Mutase 2	Protein Coding	51	GC07M044062	5.703668118	https://www.genecards.org/cgi-bin/carddisp.pl?gene=PGAM2
EGF	Epidermal Growth Factor	Protein Coding	58	GC04P109912	5.701561451	https://www.genecards.org/cgi-bin/carddisp.pl?gene=EGF
ACSM1	Acyl-CoA Synthetase Medium Chain Family Member 1	Protein Coding	46	GC16M020634	5.699780464	https://www.genecards.org/cgi-bin/carddisp.pl?ge

						ne=ACSM1
PSEN1	Presenilin 1	Protein Coding	60	GC14P073136	5.696412563	https://www.genecards.org/cgi-bin/carddisp.pl?gene=PSEN1
F5	Coagulation Factor V	Protein Coding	53	GC01M169511	5.685031414	https://www.genecards.org/cgi-bin/carddisp.pl?gene=F5
SLC5A4	Solute Carrier Family 5 Member 4	Protein Coding	46	GC22M032218	5.675738335	https://www.genecards.org/cgi-bin/carddisp.pl?gene=SLC5A4
NT5E	5'-Nucleotidase Ecto	Protein Coding	59	GC06P085449	5.674291611	https://www.genecards.org/cgi-bin/carddisp.pl?gene=NT5E
DCK	Deoxycytidine Kinase	Protein Coding	50	GC04P070992	5.673641205	https://www.genecards.org/cgi-bin/carddisp.pl?gene=DCK
SCN4A	Sodium Voltage-Gated Channel Alpha Subunit 4	Protein Coding	52	GC17M063938	5.664630413	https://www.genecards.org/cgi-bin/carddisp.pl?gene=SCN4A
THRA	Thyroid Hormone Receptor Alpha	Protein Coding	55	GC17P040058	5.65968132	https://www.genecards.org/cgi-bin/carddisp.pl?gene=THRA
HLA-DRB1	Major Histocompatibility Complex, Class II, DR Beta 1	Protein Coding	53	GC06M071772	5.655965805	https://www.genecards.org/cgi-bin/carddisp.pl?gene=HLA-DRB1
HDAC9	Histone Deacetylase 9	Protein Coding	56	GC07P018086	5.649218082	https://www.genecards.org/cgi-bin/carddisp.pl?gene=HDAC9
CREBBP	CREB Binding Protein	Protein Coding	60	GC16M008258	5.642336845	https://www.genecards.org/cgi-bin/carddisp.pl?gene=CREBBP
KARS1	Lysyl-TRNA Synthetase 1	Protein Coding	52	GC16M075800	5.635137081	https://www.genecards.org/cgi-bin/carddisp.pl?gene=KARS1
EIF2S1	Eukaryotic Translation Initiation Factor 2 Subunit Alpha	Protein Coding	50	GC14P067359	5.618702412	https://www.genecards.org/cgi-bin/carddisp.pl?gene=EIF2S1
FABP3	Fatty Acid Binding Protein 3	Protein Coding	50	GC01M031365	5.583105087	https://www.genecards.org/cgi-bin/carddisp.pl?gene=FABP3
LPXN	Leupaxin	Protein Coding	47	GC11M099445	5.580239773	https://www.genecards.org/cgi-bin/carddisp.pl?gene=LPXN
VDAC1	Voltage Dependent Anion Channel 1	Protein Coding	53	GC05M133975	5.569249153	https://www.genecards.org/cgi-bin/carddisp.pl?ge

						ne=VDAC1
EHMT2	Euchromatic Histone Lysine Methyltransferase 2	Protein Coding	52	GC06M031879	5.56857872	https://www.genecards.org/cgi-bin/carddisp.pl?gene=EHMT2
HNMT	Histamine N-Methyltransferase	Protein Coding	52	GC02P137964	5.568031788	https://www.genecards.org/cgi-bin/carddisp.pl?gene=HNMT
IL1RN	Interleukin 1 Receptor Antagonist	Protein Coding	57	GC02P123802	5.564380646	https://www.genecards.org/cgi-bin/carddisp.pl?gene=IL1RN
HLA-A	Major Histocompatibility Complex, Class I, A	Protein Coding	52	GC06P092113	5.557308197	https://www.genecards.org/cgi-bin/carddisp.pl?gene=HLA-A
FGFR2	Fibroblast Growth Factor Receptor 2	Protein Coding	63	GC10M121478	5.551764965	https://www.genecards.org/cgi-bin/carddisp.pl?gene=FGFR2
SMARCA4	SWI/SNF Related, Matrix Associated, Actin Dependent Regulator Of Chromatin, Subfamily A, Member 4	Protein Coding	57	GC19P010932	5.540520191	https://www.genecards.org/cgi-bin/carddisp.pl?gene=SMARCA4
GNRH1	Gonadotropin Releasing Hormone 1	Protein Coding	47	GC08M025419	5.540391922	https://www.genecards.org/cgi-bin/carddisp.pl?gene=GNRH1
ARNT	Aryl Hydrocarbon Receptor Nuclear Translocator	Protein Coding	51	GC01M150809	5.535038948	https://www.genecards.org/cgi-bin/carddisp.pl?gene=ARNT
ANPEP	Alanyl Aminopeptidase, Membrane	Protein Coding	57	GC15M089784	5.533632755	https://www.genecards.org/cgi-bin/carddisp.pl?gene=ANPEP
PRKCA	Protein Kinase C Alpha	Protein Coding	57	GC17P066302	5.513727188	https://www.genecards.org/cgi-bin/carddisp.pl?gene=PRKCA
CFH	Complement Factor H	Protein Coding	54	GC01P196621	5.510847569	https://www.genecards.org/cgi-bin/carddisp.pl?gene=CFH
SMPD2	Sphingomyelin Phosphodiesterase 2	Protein Coding	47	GC06P109440	5.505710602	https://www.genecards.org/cgi-bin/carddisp.pl?gene=SMPD2
KIT	KIT Proto-Oncogene, Receptor Tyrosine Kinase	Protein Coding	60	GC04P054657	5.501556873	https://www.genecards.org/cgi-bin/carddisp.pl?gene=KIT
TERT	Telomerase Reverse Transcriptase	Protein Coding	58	GC05M001253	5.497232914	https://www.genecards.org/cgi-bin/carddisp.pl?gene=TERT
MBOAT7	Membrane Bound O-Acyltransferase Domain Containing 7	Protein Coding	46	GC19M054173	5.496452332	https://www.genecards.org/cgi-bin/carddisp.pl?ge

						ne=MBOAT7
TK1	Thymidine Kinase 1	Protein Coding	52	GC17M078175	5.495685577	https://www.genecards.org/cgi-bin/carddisp.pl?gene=TK1
MTM1	Myotubularin 1	Protein Coding	50	GC0XP150562	5.492345333	https://www.genecards.org/cgi-bin/carddisp.pl?gene=MTM1
SPTLC1	Serine Palmitoyltransferase Long Chain Base Subunit 1	Protein Coding	53	GC09M096508	5.490951538	https://www.genecards.org/cgi-bin/carddisp.pl?gene=SPTLC1
NFKB1	Nuclear Factor Kappa B Subunit 1	Protein Coding	60	GC04P102501	5.488479137	https://www.genecards.org/cgi-bin/carddisp.pl?gene=NFKB1
ERCC5	ERCC Excision Repair 5, Endonuclease	Protein Coding	51	GC13P102845	5.477799416	https://www.genecards.org/cgi-bin/carddisp.pl?gene=ERCC5
SP1	Sp1 Transcription Factor	Protein Coding	53	GC12P053380	5.475884914	https://www.genecards.org/cgi-bin/carddisp.pl?gene=SP1
HNRNPK	Heterogeneous Nuclear Ribonucleoprotein K	Protein Coding	52	GC09M096203	5.475253105	https://www.genecards.org/cgi-bin/carddisp.pl?gene=HNRNPK
NPPB	Natriuretic Peptide B	Protein Coding	50	GC01M011858	5.447532177	https://www.genecards.org/cgi-bin/carddisp.pl?gene=NPPB
TNFRSF1B	TNF Receptor Superfamily Member 1B	Protein Coding	56	GC01P012301	5.443855286	https://www.genecards.org/cgi-bin/carddisp.pl?gene=TNFRSF1B
SLC22A2	Solute Carrier Family 22 Member 2	Protein Coding	50	GC06M160195	5.439086437	https://www.genecards.org/cgi-bin/carddisp.pl?gene=SLC22A2
SLC25A3	Solute Carrier Family 25 Member 3	Protein Coding	51	GC12P098593	5.434689522	https://www.genecards.org/cgi-bin/carddisp.pl?gene=SLC25A3
ABL1	ABL Proto-Oncogene 1, Non-Receptor Tyrosine Kinase	Protein Coding	59	GC09P130713	5.431687832	https://www.genecards.org/cgi-bin/carddisp.pl?gene=ABL1
SCARB2	Scavenger Receptor Class B Member 2	Protein Coding	53	GC04M076158	5.430082798	https://www.genecards.org/cgi-bin/carddisp.pl?gene=SCARB2
ENPP2	Ectonucleotide Pyrophosphatase/Phosphodiesterase 2	Protein Coding	52	GC08M119556	5.429513454	https://www.genecards.org/cgi-bin/carddisp.pl?gene=ENPP2
DNM2	Dynamin 2	Protein Coding	56	GC19P010718	5.426211834	https://www.genecards.org/cgi-bin/carddisp.pl?ge

						ne=DNM2
ENO3	Enolase 3	Protein Coding	55	GC17P004948	5.420696259	https://www.genecards.org/cgi-bin/carddisp.pl?gene=ENO3
IGFBP2	Insulin Like Growth Factor Binding Protein 2	Protein Coding	51	GC02P216632	5.415290833	https://www.genecards.org/cgi-bin/carddisp.pl?gene=IGFBP2
AGA	Aspartylglucosaminidase	Protein Coding	52	GC04M177430	5.402294636	https://www.genecards.org/cgi-bin/carddisp.pl?gene=AGA
UROS	Uroporphyrinogen III Synthase	Protein Coding	48	GC10M125784	5.398243904	https://www.genecards.org/cgi-bin/carddisp.pl?gene=UROS
PDE5A	Phosphodiesterase 5A	Protein Coding	52	GC04M119494	5.394932747	https://www.genecards.org/cgi-bin/carddisp.pl?gene=PDE5A
BLVRB	Biliverdin Reductase B	Protein Coding	46	GC19M040447	5.39385891	https://www.genecards.org/cgi-bin/carddisp.pl?gene=BLVRB
SERPINC1	Serpin Family C Member 1	Protein Coding	56	GC01M174653	5.383127689	https://www.genecards.org/cgi-bin/carddisp.pl?gene=SERPINC1
PLA2G5	Phospholipase A2 Group V	Protein Coding	48	GC01P020028	5.379155159	https://www.genecards.org/cgi-bin/carddisp.pl?gene=PLA2G5
CD40LG	CD40 Ligand	Protein Coding	57	GC0XP136649	5.369486809	https://www.genecards.org/cgi-bin/carddisp.pl?gene=CD40LG
NCOA2	Nuclear Receptor Coactivator 2	Protein Coding	52	GC08M070109	5.368606567	https://www.genecards.org/cgi-bin/carddisp.pl?gene=NCOA2
CEL	Carboxyl Ester Lipase	Protein Coding	52	GC09P133061	5.360735893	https://www.genecards.org/cgi-bin/carddisp.pl?gene=CEL
ADH5	Alcohol Dehydrogenase 5 (Class III), Chi Polypeptide	Protein Coding	53	GC04M099070	5.359493732	https://www.genecards.org/cgi-bin/carddisp.pl?gene=ADH5
ACSL1	Acyl-CoA Synthetase Long Chain Family Member 1	Protein Coding	52	GC04M184755	5.358801365	https://www.genecards.org/cgi-bin/carddisp.pl?gene=ACSL1
VWF	Von Willebrand Factor	Protein Coding	55	GC12M005949	5.354763508	https://www.genecards.org/cgi-bin/carddisp.pl?gene=VWF
FSHR	Follicle Stimulating Hormone Receptor	Protein Coding	54	GC02M048962	5.346429825	https://www.genecards.org/cgi-bin/carddisp.pl?ge

						ne=FSHR
SAA1	Serum Amyloid A1	Protein Coding	46	GC11P018579	5.33243227	https://www.genecards.org/cgi-bin/carddisp.pl?gene=SAA1
PSTPIP1	Proline-Serine-Threonine Phosphatase Interacting Protein 1	Protein Coding	50	GC15P076993	5.325975895	https://www.genecards.org/cgi-bin/carddisp.pl?gene=PSTPIP1
PDHB	Pyruvate Dehydrogenase E1 Subunit Beta	Protein Coding	52	GC03M058428	5.320480824	https://www.genecards.org/cgi-bin/carddisp.pl?gene=PDHB
DAO	D-Amino Acid Oxidase	Protein Coding	51	GC12P108859	5.318780422	https://www.genecards.org/cgi-bin/carddisp.pl?gene=DAO
SHC1	SHC Adaptor Protein 1	Protein Coding	50	GC01M154962	5.312389851	https://www.genecards.org/cgi-bin/carddisp.pl?gene=SHC1
PTH1R	Parathyroid Hormone 1 Receptor	Protein Coding	57	GC03P046877	5.307143688	https://www.genecards.org/cgi-bin/carddisp.pl?gene=PTH1R
GRIN2A	Glutamate Ionotropic Receptor NMDA Type Subunit 2A	Protein Coding	59	GC16M009753	5.30063343	https://www.genecards.org/cgi-bin/carddisp.pl?gene=GRIN2A
FABP12	Fatty Acid Binding Protein 12	Protein Coding	38	GC08M081524	5.29920435	https://www.genecards.org/cgi-bin/carddisp.pl?gene=FABP12
IL6R	Interleukin 6 Receptor	Protein Coding	57	GC01P154405	5.290779114	https://www.genecards.org/cgi-bin/carddisp.pl?gene=IL6R
RAD51	RAD51 Recombinase	Protein Coding	57	GC15P040694	5.285461426	https://www.genecards.org/cgi-bin/carddisp.pl?gene=RAD51
HSP90AA1	Heat Shock Protein 90 Alpha Family Class A Member 1	Protein Coding	57	GC14M102080	5.282570362	https://www.genecards.org/cgi-bin/carddisp.pl?gene=HSP90AA1
STAT6	Signal Transducer And Activator Of Transcription 6	Protein Coding	57	GC12M057095	5.278360844	https://www.genecards.org/cgi-bin/carddisp.pl?gene=STAT6
APEX1	Apurinic/Apyrimidinic Endodeoxyribonuclease 1	Protein Coding	52	GC14P020455	5.276880264	https://www.genecards.org/cgi-bin/carddisp.pl?gene=APEX1
SLC22A12	Solute Carrier Family 22 Member 12	Protein Coding	51	GC11P064609	5.276699543	https://www.genecards.org/cgi-bin/carddisp.pl?gene=SLC22A12
TP63	Tumor Protein P63	Protein Coding	53	GC03P189598	5.272655487	https://www.genecards.org/cgi-bin/carddisp.pl?ge

						ne=TP63
DDIT3	DNA Damage Inducible Transcript 3	Protein Coding	52	GC12M057516	5.258716583	https://www.genecards.org/cgi-bin/carddisp.pl?gene=DDIT3
APOH	Apolipoprotein H	Protein Coding	51	GC17M066212	5.254735947	https://www.genecards.org/cgi-bin/carddisp.pl?gene=APOH
KMO	Kynurenine 3-Monooxygenase	Protein Coding	53	GC01P241532	5.254042149	https://www.genecards.org/cgi-bin/carddisp.pl?gene=KMO
CTSB	Cathepsin B	Protein Coding	59	GC08M011842	5.253155708	https://www.genecards.org/cgi-bin/carddisp.pl?gene=CTSB
SRC	SRC Proto-Oncogene, Non-Receptor Tyrosine Kinase	Protein Coding	58	GC20P037344	5.242369652	https://www.genecards.org/cgi-bin/carddisp.pl?gene=SRC
DGKB	Diacylglycerol Kinase Beta	Protein Coding	50	GC07M014145	5.21872282	https://www.genecards.org/cgi-bin/carddisp.pl?gene=DGKB
GATA6	GATA Binding Protein 6	Protein Coding	53	GC18P022169	5.218309879	https://www.genecards.org/cgi-bin/carddisp.pl?gene=GATA6
IL3	Interleukin 3	Protein Coding	52	GC05P132060	5.218032837	https://www.genecards.org/cgi-bin/carddisp.pl?gene=IL3
CD46	CD46 Molecule	Protein Coding	55	GC01P207752	5.215385437	https://www.genecards.org/cgi-bin/carddisp.pl?gene=CD46
F10	Coagulation Factor X	Protein Coding	57	GC13P113122	5.205245495	https://www.genecards.org/cgi-bin/carddisp.pl?gene=F10
FLNA	Filamin A	Protein Coding	55	GC0XM154348	5.204764843	https://www.genecards.org/cgi-bin/carddisp.pl?gene=FLNA
ACOT1	Acyl-CoA Thioesterase 1	Protein Coding	42	GC14P073493	5.202662945	https://www.genecards.org/cgi-bin/carddisp.pl?gene=ACOT1
PIK3CB	Phosphatidylinositol-4,5-Bisphosphate 3-Kinase Catalytic Subunit Beta	Protein Coding	55	GC03M138652	5.201896667	https://www.genecards.org/cgi-bin/carddisp.pl?gene=PIK3CB
CDKN2A	Cyclin Dependent Kinase Inhibitor 2A	Protein Coding	57	GC09M021967	5.197295666	https://www.genecards.org/cgi-bin/carddisp.pl?gene=CDKN2A
ALOX15B	Arachidonate 15-Lipoxygenase Type B	Protein Coding	48	GC17P008039	5.185217857	https://www.genecards.org/cgi-bin/carddisp.pl?ge

						ne=ALOX15B
GSK3A	Glycogen Synthase Kinase 3 Alpha	Protein Coding	56	GC19M070637	5.179674149	https://www.genecards.org/cgi-bin/carddisp.pl?gene=GSK3A
AQP2	Aquaporin 2	Protein Coding	53	GC12P049950	5.167989254	https://www.genecards.org/cgi-bin/carddisp.pl?gene=AQP2
RPS6KB1	Ribosomal Protein S6 Kinase B1	Protein Coding	55	GC17P059893	5.164863586	https://www.genecards.org/cgi-bin/carddisp.pl?gene=RPS6KB1
CXCL10	C-X-C Motif Chemokine Ligand 10	Protein Coding	51	GC04M076021	5.147573948	https://www.genecards.org/cgi-bin/carddisp.pl?gene=CXCL10
APBB1	Amyloid Beta Precursor Protein Binding Family B Member 1	Protein Coding	51	GC11M006936	5.13942337	https://www.genecards.org/cgi-bin/carddisp.pl?gene=APBB1
FDX1	Ferredoxin 1	Protein Coding	45	GC11P110429	5.134989262	https://www.genecards.org/cgi-bin/carddisp.pl?gene=FDX1
CD44	CD44 Molecule (Indian Blood Group)	Protein Coding	55	GC11P035139	5.127356052	https://www.genecards.org/cgi-bin/carddisp.pl?gene=CD44
IL4I1	Interleukin 4 Induced 1	Protein Coding	46	GC19M070913	5.122427464	https://www.genecards.org/cgi-bin/carddisp.pl?gene=IL4I1
IL17A	Interleukin 17A	Protein Coding	50	GC06P052186	5.119236469	https://www.genecards.org/cgi-bin/carddisp.pl?gene=IL17A
AOC3	Amine Oxidase Copper Containing 3	Protein Coding	52	GC17P042851	5.119176865	https://www.genecards.org/cgi-bin/carddisp.pl?gene=AOC3
KAT5	Lysine Acetyltransferase 5	Protein Coding	57	GC11P065711	5.108136654	https://www.genecards.org/cgi-bin/carddisp.pl?gene=KAT5
HGF	Hepatocyte Growth Factor	Protein Coding	58	GC07M081699	5.10415411	https://www.genecards.org/cgi-bin/carddisp.pl?gene=HGF
XRCC1	X-Ray Repair Cross Complementing 1	Protein Coding	51	GC19M043543	5.097961426	https://www.genecards.org/cgi-bin/carddisp.pl?gene=XRCC1
FFAR1	Free Fatty Acid Receptor 1	Protein Coding	47	GC19P073870	5.093933582	https://www.genecards.org/cgi-bin/carddisp.pl?gene=FFAR1
EPAS1	Endothelial PAS Domain Protein 1	Protein Coding	57	GC02P046293	5.093504906	https://www.genecards.org/cgi-bin/carddisp.pl?ge

						ne=EPAS1
ABCC5	ATP Binding Cassette Subfamily C Member 5	Protein Coding	51	GC03M183919	5.09292078	https://www.genecards.org/cgi-bin/carddisp.pl?gene=ABCC5
ITGB2	Integrin Subunit Beta 2	Protein Coding	57	GC21M044885	5.083768845	https://www.genecards.org/cgi-bin/carddisp.pl?gene=ITGB2
ACOT8	Acyl-CoA Thioesterase 8	Protein Coding	44	GC20M045841	5.071051598	https://www.genecards.org/cgi-bin/carddisp.pl?gene=ACOT8
APPL1	Adaptor Protein, Phosphotyrosine Interacting With PH Domain And Leucine Zipper 1	Protein Coding	51	GC03P057227	5.065500736	https://www.genecards.org/cgi-bin/carddisp.pl?gene=APPL1
HMOX2	Heme Oxygenase 2	Protein Coding	53	GC16P004474	5.051269531	https://www.genecards.org/cgi-bin/carddisp.pl?gene=HMOX2
CSNK2B	Casein Kinase 2 Beta	Protein Coding	55	GC06P092157	5.048334599	https://www.genecards.org/cgi-bin/carddisp.pl?gene=CSNK2B
SRD5A3	Steroid 5 Alpha-Reductase 3	Protein Coding	50	GC04P055346	5.043519974	https://www.genecards.org/cgi-bin/carddisp.pl?gene=SRD5A3
BCR	BCR Activator Of RhoGEF And GTPase	Protein Coding	59	GC22P023179	5.043236256	https://www.genecards.org/cgi-bin/carddisp.pl?gene=BCR
CPT1C	Carnitine Palmitoyltransferase 1C	Protein Coding	50	GC19P049690	5.040052414	https://www.genecards.org/cgi-bin/carddisp.pl?gene=CPT1C
NGF	Nerve Growth Factor	Protein Coding	58	GC01M115285	5.035580635	https://www.genecards.org/cgi-bin/carddisp.pl?gene=NGF
RARA	Retinoic Acid Receptor Alpha	Protein Coding	57	GC17P040309	5.034917831	https://www.genecards.org/cgi-bin/carddisp.pl?gene=RARA
OSBP	Oxysterol Binding Protein	Protein Coding	47	GC11M099460	5.02068615	https://www.genecards.org/cgi-bin/carddisp.pl?gene=OSBP
RELA	RELA Proto-Oncogene, NF-KB Subunit	Protein Coding	59	GC11M065653	5.020265102	https://www.genecards.org/cgi-bin/carddisp.pl?gene=RELA
GPBAR1	G Protein-Coupled Bile Acid Receptor 1	Protein Coding	47	GC02P218259	5.01853466	https://www.genecards.org/cgi-bin/carddisp.pl?gene=GPBAR1
SLC22A6	Solute Carrier Family 22 Member 6	Protein Coding	51	GC11M099607	5.0164814	https://www.genecards.org/cgi-bin/carddisp.pl?ge

						ne=SLC22A6
SLC16A2	Solute Carrier Family 16 Member 2	Protein Coding	50	GC0XP074510	5.015535355	https://www.genecards.org/cgi-bin/carddisp.pl?gene=SLC16A2
RET	Ret Proto-Oncogene	Protein Coding	61	GC10P043237	5.005290031	https://www.genecards.org/cgi-bin/carddisp.pl?gene=RET
DHODH	Dihydroorotate Dehydrogenase (Quinone)	Protein Coding	53	GC16P072008	5.001195908	https://www.genecards.org/cgi-bin/carddisp.pl?gene=DHODH
PLCG1	Phospholipase C Gamma 1	Protein Coding	53	GC20P041136	4.999927521	https://www.genecards.org/cgi-bin/carddisp.pl?gene=PLCG1
IL6ST	Interleukin 6 Cytokine Family Signal Transducer	Protein Coding	56	GC05M055935	4.997031212	https://www.genecards.org/cgi-bin/carddisp.pl?gene=IL6ST
SPHK1	Sphingosine Kinase 1	Protein Coding	53	GC17P076376	4.993997097	https://www.genecards.org/cgi-bin/carddisp.pl?gene=SPHK1
SLC3A2	Solute Carrier Family 3 Member 2	Protein Coding	50	GC11P062856	4.993354797	https://www.genecards.org/cgi-bin/carddisp.pl?gene=SLC3A2
MIF	Macrophage Migration Inhibitory Factor	Protein Coding	55	GC22P023894	4.985460281	https://www.genecards.org/cgi-bin/carddisp.pl?gene=MIF
GIP	Gastric Inhibitory Polypeptide	Protein Coding	44	GC17M048958	4.984392643	https://www.genecards.org/cgi-bin/carddisp.pl?gene=GIP
GART	Phosphoribosylglycinamide Formyltransferase, Phosphoribosylglycinamide Synthetase, Phosphoribosylaminoimidazole Synthetase	Protein Coding	50	GC21M033503	4.982872963	https://www.genecards.org/cgi-bin/carddisp.pl?gene=GART
SELP	Selectin P	Protein Coding	52	GC01M169558	4.982637405	https://www.genecards.org/cgi-bin/carddisp.pl?gene=SELP
RPS27A	Ribosomal Protein S27a	Protein Coding	48	GC02P055231	4.981519699	https://www.genecards.org/cgi-bin/carddisp.pl?gene=RPS27A
GAST	Gastrin	Protein Coding	46	GC17P041712	4.976635933	https://www.genecards.org/cgi-bin/carddisp.pl?gene=GAST
TNFRSF1A	TNF Receptor Superfamily Member 1A	Protein Coding	57	GC12M006328	4.969126225	https://www.genecards.org/cgi-bin/carddisp.pl?gene=TNFRSF1A

HLA-DQB1	Major Histocompatibility Complex, Class II, DQ Beta 1	Protein Coding	48	GC06M071781	4.959109306	https://www.genecards.org/cgi-bin/carddisp.pl?gene=HLA-DQB1
COASY	Coenzyme A Synthase	Protein Coding	52	GC17P042561	4.956408024	https://www.genecards.org/cgi-bin/carddisp.pl?gene=COASY
MYD88	MYD88 Innate Immune Signal Transduction Adaptor	Protein Coding	56	GC03P038152	4.956139088	https://www.genecards.org/cgi-bin/carddisp.pl?gene=MYD88
CTSG	Cathepsin G	Protein Coding	52	GC14M024573	4.955893993	https://www.genecards.org/cgi-bin/carddisp.pl?gene=CTSG
PRKACA	Protein Kinase CAMP-Activated Catalytic Subunit Alpha	Protein Coding	59	GC19M014672	4.948847294	https://www.genecards.org/cgi-bin/carddisp.pl?gene=PRKACA
KDR	Kinase Insert Domain Receptor	Protein Coding	60	GC04M055078	4.945917606	https://www.genecards.org/cgi-bin/carddisp.pl?gene=KDR
DGAT2	Diacylglycerol O-Acyltransferase 2	Protein Coding	49	GC11P075759	4.939157963	https://www.genecards.org/cgi-bin/carddisp.pl?gene=DGAT2
HDAC1	Histone Deacetylase 1	Protein Coding	57	GC01P032292	4.924383163	https://www.genecards.org/cgi-bin/carddisp.pl?gene=HDAC1
BTK	Bruton Tyrosine Kinase	Protein Coding	60	GC0XM101349	4.922725201	https://www.genecards.org/cgi-bin/carddisp.pl?gene=BTK
VIM	Vimentin	Protein Coding	57	GC10P017227	4.918421745	https://www.genecards.org/cgi-bin/carddisp.pl?gene=VIM
CHEK2	Checkpoint Kinase 2	Protein Coding	61	GC22M028687	4.904974937	https://www.genecards.org/cgi-bin/carddisp.pl?gene=CHEK2
CALR	Calreticulin	Protein Coding	58	GC19P012938	4.903204918	https://www.genecards.org/cgi-bin/carddisp.pl?gene=CALR
HMGCS1	3-Hydroxy-3-Methylglutaryl-CoA Synthase 1	Protein Coding	48	GC05M044196	4.901966095	https://www.genecards.org/cgi-bin/carddisp.pl?gene=HMGCS1
GAD2	Glutamate Decarboxylase 2	Protein Coding	51	GC10P026216	4.901376247	https://www.genecards.org/cgi-bin/carddisp.pl?gene=GAD2
CYB5R3	Cytochrome B5 Reductase 3	Protein Coding	52	GC22M061501	4.898460865	https://www.genecards.org/cgi-bin/carddisp.pl?gene=CYB5R3

ALAS1	5'-Aminolevulinate Synthase 1	Protein Coding	50	GC03P052198	4.893675804	https://www.genecards.org/cgi-bin/carddisp.pl?gene=ALAS1
BAX	BCL2 Associated X, Apoptosis Regulator	Protein Coding	57	GC19P048954	4.886703491	https://www.genecards.org/cgi-bin/carddisp.pl?gene=BAX
ACOT4	Acyl-CoA Thioesterase 4	Protein Coding	42	GC14P073592	4.880039692	https://www.genecards.org/cgi-bin/carddisp.pl?gene=ACOT4
SPR	Sepiapterin Reductase	Protein Coding	56	GC02P072850	4.879478931	https://www.genecards.org/cgi-bin/carddisp.pl?gene=SPR
VHL	Von Hippel-Lindau Tumor Suppressor	Protein Coding	54	GC03P013681	4.872748375	https://www.genecards.org/cgi-bin/carddisp.pl?gene=VHL
SETD2	SET Domain Containing 2, Histone Lysine Methyltransferase	Protein Coding	53	GC03M047033	4.872437	https://www.genecards.org/cgi-bin/carddisp.pl?gene=SETD2
HBA2	Hemoglobin Subunit Alpha 2	Protein Coding	48	GC16P013429	4.87237978	https://www.genecards.org/cgi-bin/carddisp.pl?gene=HBA2
ACSL6	Acyl-CoA Synthetase Long Chain Family Member 6	Protein Coding	47	GC05M131949	4.870249748	https://www.genecards.org/cgi-bin/carddisp.pl?gene=ACSL6
IDE	Insulin Degrading Enzyme	Protein Coding	55	GC10M092451	4.864925861	https://www.genecards.org/cgi-bin/carddisp.pl?gene=IDE
HSPA5	Heat Shock Protein Family A (Hsp70) Member 5	Protein Coding	55	GC09M125234	4.862672329	https://www.genecards.org/cgi-bin/carddisp.pl?gene=HSPA5
ALDH3B2	Aldehyde Dehydrogenase 3 Family Member B2	Protein Coding	45	GC11M067662	4.860623837	https://www.genecards.org/cgi-bin/carddisp.pl?gene=ALDH3B2
CLCN1	Chloride Voltage-Gated Channel 1	Protein Coding	50	GC07P143316	4.854913712	https://www.genecards.org/cgi-bin/carddisp.pl?gene=CLCN1
NGFR	Nerve Growth Factor Receptor	Protein Coding	54	GC17P049495	4.847626686	https://www.genecards.org/cgi-bin/carddisp.pl?gene=NGFR
KRT18	Keratin 18	Protein Coding	56	GC12P052948	4.843678474	https://www.genecards.org/cgi-bin/carddisp.pl?gene=KRT18
HBG2	Hemoglobin Subunit Gamma 2	Protein Coding	47	GC11M006835	4.837977886	https://www.genecards.org/cgi-bin/carddisp.pl?gene=HBG2

PSPH	Phosphoserine Phosphatase	Protein Coding	52	GC07M061179	4.837378979	https://www.genecards.org/cgi-bin/carddisp.pl?gene=PSPH
GNPAT	Glyceronephosphate O-Acyltransferase	Protein Coding	51	GC01P231241	4.835204601	https://www.genecards.org/cgi-bin/carddisp.pl?gene=GNPAT
BACE1	Beta-Secretase 1	Protein Coding	53	GC11M117285	4.827996254	https://www.genecards.org/cgi-bin/carddisp.pl?gene=BACE1
IGHE	Immunoglobulin Heavy Constant Epsilon	Protein Coding	36	GC14M113319	4.824530125	https://www.genecards.org/cgi-bin/carddisp.pl?gene=IGHE
CASK	Calcium/Calmodulin Dependent Serine Protein Kinase	Protein Coding	56	GC0XM041514	4.822626591	https://www.genecards.org/cgi-bin/carddisp.pl?gene=CASK
DLST	Dihydrolipoamide S-Succinyltransferase	Protein Coding	52	GC14P074881	4.821403027	https://www.genecards.org/cgi-bin/carddisp.pl?gene=DLST
FLG	Filaggrin	Protein Coding	47	GC01M152828	4.818543434	https://www.genecards.org/cgi-bin/carddisp.pl?gene=FLG
HYOU1	Hypoxia Up-Regulated 1	Protein Coding	52	GC11M119272	4.812234402	https://www.genecards.org/cgi-bin/carddisp.pl?gene=HYOU1
CDKL5	Cyclin Dependent Kinase Like 5	Protein Coding	50	GC0XP018425	4.80866766	https://www.genecards.org/cgi-bin/carddisp.pl?gene=CDKL5
AOC1	Amine Oxidase Copper Containing 1	Protein Coding	49	GC07P150824	4.807901382	https://www.genecards.org/cgi-bin/carddisp.pl?gene=AOC1
GRIN1	Glutamate Ionotropic Receptor NMDA Type Subunit 1	Protein Coding	57	GC09P137184	4.80106926	https://www.genecards.org/cgi-bin/carddisp.pl?gene=GRIN1
FUT2	Fucosyltransferase 2	Protein Coding	52	GC19P048695	4.799116611	https://www.genecards.org/cgi-bin/carddisp.pl?gene=FUT2
SLCO1B3	Solute Carrier Organic Anion Transporter Family Member 1B3	Protein Coding	51	GC12P020810	4.793128967	https://www.genecards.org/cgi-bin/carddisp.pl?gene=SLCO1B3
TACR1	Tachykinin Receptor 1	Protein Coding	51	GC02M075203	4.782237053	https://www.genecards.org/cgi-bin/carddisp.pl?gene=TACR1
PIK3CD	Phosphatidylinositol-4,5-Bisphosphate 3-Kinase Catalytic Subunit Delta	Protein Coding	60	GC01P009628	4.779503822	https://www.genecards.org/cgi-bin/carddisp.pl?gene=PIK3CD

AIRE	Autoimmune Regulator	Protein Coding	50	GC21P044285	4.776328087	https://www.genecards.org/cgi-bin/carddisp.pl?gene=AIRE
ACSM2B	Acyl-CoA Synthetase Medium Chain Family Member 2B	Protein Coding	40	GC16M020547	4.77520895	https://www.genecards.org/cgi-bin/carddisp.pl?gene=ACSM2B
ATOX1	Antioxidant 1 Copper Chaperone	Protein Coding	46	GC05M151743	4.772132397	https://www.genecards.org/cgi-bin/carddisp.pl?gene=ATOX1
LCN2	Lipocalin 2	Protein Coding	52	GC09P128149	4.771922112	https://www.genecards.org/cgi-bin/carddisp.pl?gene=LCN2
HSD17B2	Hydroxysteroid 17-Beta Dehydrogenase 2	Protein Coding	50	GC16P082068	4.765637875	https://www.genecards.org/cgi-bin/carddisp.pl?gene=HSD17B2
ACP1	Acid Phosphatase 1	Protein Coding	50	GC02P000402	4.764500618	https://www.genecards.org/cgi-bin/carddisp.pl?gene=ACP1
HSD17B7	Hydroxysteroid 17-Beta Dehydrogenase 7	Protein Coding	50	GC01P162790	4.741034031	https://www.genecards.org/cgi-bin/carddisp.pl?gene=HSD17B7
CDK1	Cyclin Dependent Kinase 1	Protein Coding	54	GC10P060772	4.737948895	https://www.genecards.org/cgi-bin/carddisp.pl?gene=CDK1
ANGPT2	Angiopoietin 2	Protein Coding	55	GC08M006499	4.737078667	https://www.genecards.org/cgi-bin/carddisp.pl?gene=ANGPT2
HSPB1	Heat Shock Protein Family B (Small) Member 1	Protein Coding	59	GC07P076302	4.732555866	https://www.genecards.org/cgi-bin/carddisp.pl?gene=HSPB1
SAT1	Spermidine/Spermine N1-Acetyltransferase 1	Protein Coding	51	GC0XP023784	4.724458218	https://www.genecards.org/cgi-bin/carddisp.pl?gene=SAT1
HSPA8	Heat Shock Protein Family A (Hsp70) Member 8	Protein Coding	56	GC11M123057	4.723155022	https://www.genecards.org/cgi-bin/carddisp.pl?gene=HSPA8
TXNRD2	Thioredoxin Reductase 2	Protein Coding	51	GC22M019863	4.720499992	https://www.genecards.org/cgi-bin/carddisp.pl?gene=TXNRD2
KCNQ1	Potassium Voltage-Gated Channel Subfamily Q Member 1	Protein Coding	57	GC11P002444	4.712696552	https://www.genecards.org/cgi-bin/carddisp.pl?gene=KCNQ1
HDC	Histidine Decarboxylase	Protein Coding	51	GC15M050241	4.712660789	https://www.genecards.org/cgi-bin/carddisp.pl?gene=HDC

GRIN2B	Glutamate Ionotropic Receptor NMDA Type Subunit 2B	Protein Coding	59	GC12M013437	4.70554781	https://www.genecards.org/cgi-bin/carddisp.pl?gene=GRIN2B
ACE2	Angiotensin Converting Enzyme 2	Protein Coding	56	GC0XM015494	4.698474884	https://www.genecards.org/cgi-bin/carddisp.pl?gene=ACE2
RAF1	Raf-1 Proto-Oncogene, Serine/Threonine Kinase	Protein Coding	61	GC03M012583	4.69260931	https://www.genecards.org/cgi-bin/carddisp.pl?gene=RAF1
NTRK2	Neurotrophic Receptor Tyrosine Kinase 2	Protein Coding	60	GC09P084668	4.689634323	https://www.genecards.org/cgi-bin/carddisp.pl?gene=NTRK2
TIMM8A	Translocase Of Inner Mitochondrial Membrane 8A	Protein Coding	48	GC0XM101345	4.683206081	https://www.genecards.org/cgi-bin/carddisp.pl?gene=TIMM8A
DNAH8	Dynein Axonemal Heavy Chain 8	Protein Coding	44	GC06P092313	4.681497574	https://www.genecards.org/cgi-bin/carddisp.pl?gene=DNAH8
CROT	Carnitine O-Octanoyltransferase	Protein Coding	46	GC07P087369	4.68108654	https://www.genecards.org/cgi-bin/carddisp.pl?gene=CROT
YAP1	Yes1 Associated Transcriptional Regulator	Protein Coding	55	GC11P102110	4.661038876	https://www.genecards.org/cgi-bin/carddisp.pl?gene=YAP1
LPCAT1	Lysophosphatidylcholine Acyltransferase 1	Protein Coding	42	GC05M001456	4.657835007	https://www.genecards.org/cgi-bin/carddisp.pl?gene=LPCAT1
IL7	Interleukin 7	Protein Coding	50	GC08M078689	4.655595779	https://www.genecards.org/cgi-bin/carddisp.pl?gene=IL7
ANGPT1	Angiopoietin 1	Protein Coding	53	GC08M107246	4.639920712	https://www.genecards.org/cgi-bin/carddisp.pl?gene=ANGPT1
SFTPD	Surfactant Protein D	Protein Coding	51	GC10M079937	4.638412476	https://www.genecards.org/cgi-bin/carddisp.pl?gene=SFTPD
SLCO1A2	Solute Carrier Organic Anion Transporter Family Member 1A2	Protein Coding	44	GC12M021264	4.631290436	https://www.genecards.org/cgi-bin/carddisp.pl?gene=SLCO1A2
ELOVL2	ELOVL Fatty Acid Elongase 2	Protein Coding	45	GC06M010980	4.627637386	https://www.genecards.org/cgi-bin/carddisp.pl?gene=ELOVL2
HSPA4	Heat Shock Protein Family A (Hsp70) Member 4	Protein Coding	49	GC05P133054	4.618183136	https://www.genecards.org/cgi-bin/carddisp.pl?gene=HSPA4

CSNK2A1	Casein Kinase 2 Alpha 1	Protein Coding	57	GC20M000472	4.617533207	https://www.genecards.org/cgi-bin/carddisp.pl?gene=CSNK2A1
SLC4A4	Solute Carrier Family 4 Member 4	Protein Coding	53	GC04P071063	4.614068985	https://www.genecards.org/cgi-bin/carddisp.pl?gene=SLC4A4
CNDP1	Carnosine Dipeptidase 1	Protein Coding	50	GC18P074534	4.612370491	https://www.genecards.org/cgi-bin/carddisp.pl?gene=CNDP1
LTA4H	Leukotriene A4 Hydrolase	Protein Coding	53	GC12M096000	4.609683037	https://www.genecards.org/cgi-bin/carddisp.pl?gene=LTA4H
CHGA	Chromogranin A	Protein Coding	50	GC14P092950	4.607313633	https://www.genecards.org/cgi-bin/carddisp.pl?gene=CHGA
KCNH2	Potassium Voltage-Gated Channel Subfamily H Member 2	Protein Coding	57	GC07M150944	4.600515842	https://www.genecards.org/cgi-bin/carddisp.pl?gene=KCNH2
CYBA	Cytochrome B-245 Alpha Chain	Protein Coding	54	GC16M088643	4.597821712	https://www.genecards.org/cgi-bin/carddisp.pl?gene=CYBA
ACSM2A	Acyl-CoA Synthetase Medium Chain Family Member 2A	Protein Coding	39	GC16P020463	4.596745014	https://www.genecards.org/cgi-bin/carddisp.pl?gene=ACSM2A
PTGES	Prostaglandin E Synthase	Protein Coding	48	GC09M129738	4.596619606	https://www.genecards.org/cgi-bin/carddisp.pl?gene=PTGES
TFAP2A	Transcription Factor AP-2 Alpha	Protein Coding	53	GC06M010393	4.594346046	https://www.genecards.org/cgi-bin/carddisp.pl?gene=TFAP2A
SLC6A14	Solute Carrier Family 6 Member 14	Protein Coding	48	GC0XP116436	4.591845036	https://www.genecards.org/cgi-bin/carddisp.pl?gene=SLC6A14
HTR2C	5-Hydroxytryptamine Receptor 2C	Protein Coding	52	GC0XP114584	4.588009834	https://www.genecards.org/cgi-bin/carddisp.pl?gene=HTR2C
PRMT1	Protein Arginine Methyltransferase 1	Protein Coding	55	GC19P049675	4.583292007	https://www.genecards.org/cgi-bin/carddisp.pl?gene=PRMT1
CAMK2G	Calcium/Calmodulin Dependent Protein Kinase II Gamma	Protein Coding	54	GC10M073812	4.57988739	https://www.genecards.org/cgi-bin/carddisp.pl?gene=CAMK2G
HSPD1	Heat Shock Protein Family D (Hsp60) Member 1	Protein Coding	55	GC02M197486	4.576894283	https://www.genecards.org/cgi-bin/carddisp.pl?gene=HSPD1

SLC7A11	Solute Carrier Family 7 Member 11	Protein Coding	51	GC04M138164	4.569381237	https://www.genecards.org/cgi-bin/carddisp.pl?gene=SLC7A11
NCOA1	Nuclear Receptor Coactivator 1	Protein Coding	51	GC02P024492	4.565952778	https://www.genecards.org/cgi-bin/carddisp.pl?gene=NCOA1
SLC6A4	Solute Carrier Family 6 Member 4	Protein Coding	55	GC17M030194	4.565765858	https://www.genecards.org/cgi-bin/carddisp.pl?gene=SLC6A4
SMN1	Survival Of Motor Neuron 1, Telomeric	Protein Coding	47	GC05P070924	4.564593792	https://www.genecards.org/cgi-bin/carddisp.pl?gene=SMN1
TLR2	Toll Like Receptor 2	Protein Coding	59	GC04P153684	4.562651634	https://www.genecards.org/cgi-bin/carddisp.pl?gene=TLR2
P2RX7	Purinergic Receptor P2X 7	Protein Coding	54	GC12P126979	4.556344986	https://www.genecards.org/cgi-bin/carddisp.pl?gene=P2RX7
RHOA	Ras Homolog Family Member A	Protein Coding	57	GC03M049359	4.555493832	https://www.genecards.org/cgi-bin/carddisp.pl?gene=RHOA
MLH1	MutL Homolog 1	Protein Coding	55	GC03P036993	4.549809933	https://www.genecards.org/cgi-bin/carddisp.pl?gene=MLH1
ADRB1	Adrenoceptor Beta 1	Protein Coding	54	GC10P114044	4.549460411	https://www.genecards.org/cgi-bin/carddisp.pl?gene=ADRB1
TIMP2	TIMP Metallopeptidase Inhibitor 2	Protein Coding	48	GC17M078852	4.54147768	https://www.genecards.org/cgi-bin/carddisp.pl?gene=TIMP2
DBI	Diazepam Binding Inhibitor, Acyl-CoA Binding Protein	Protein Coding	51	GC02P119366	4.539021969	https://www.genecards.org/cgi-bin/carddisp.pl?gene=DBI
CDH1	Cadherin 1	Protein Coding	56	GC16P068737	4.532455921	https://www.genecards.org/cgi-bin/carddisp.pl?gene=CDH1
CDKN2B	Cyclin Dependent Kinase Inhibitor 2B	Protein Coding	53	GC09M022002	4.53066206	https://www.genecards.org/cgi-bin/carddisp.pl?gene=CDKN2B
PLCD1	Phospholipase C Delta 1	Protein Coding	55	GC03M038008	4.52986908	https://www.genecards.org/cgi-bin/carddisp.pl?gene=PLCD1
CYSLTR1	Cysteinyl Leukotriene Receptor 1	Protein Coding	50	GC0XM078271	4.527486801	https://www.genecards.org/cgi-bin/carddisp.pl?gene=CYSLTR1

SPTLC2	Serine Palmitoyltransferase Long Chain Base Subunit 2	Protein Coding	54	GC14M077505	4.527154922	https://www.genecards.org/cgi-bin/carddisp.pl?gene=SPTLC2
ARAF	A-Raf Proto-Oncogene, Serine/Threonine Kinase	Protein Coding	53	GC0XP047710	4.521902084	https://www.genecards.org/cgi-bin/carddisp.pl?gene=ARAF
DGKQ	Diacylglycerol Kinase Theta	Protein Coding	48	GC04M000958	4.516810894	https://www.genecards.org/cgi-bin/carddisp.pl?gene=DGKQ
ACAA1	Acetyl-CoA Acyltransferase 1	Protein Coding	48	GC03M038103	4.513883114	https://www.genecards.org/cgi-bin/carddisp.pl?gene=ACAA1
BLVRA	Biliverdin Reductase A	Protein Coding	51	GC07P043758	4.509799004	https://www.genecards.org/cgi-bin/carddisp.pl?gene=BLVRA
RPA1	Replication Protein A1	Protein Coding	53	GC17P001829	4.499402523	https://www.genecards.org/cgi-bin/carddisp.pl?gene=RPA1
CDKN1A	Cyclin Dependent Kinase Inhibitor 1A	Protein Coding	55	GC06P092294	4.492255211	https://www.genecards.org/cgi-bin/carddisp.pl?gene=CDKN1A
LTF	Lactotransferrin	Protein Coding	52	GC03M046435	4.481446266	https://www.genecards.org/cgi-bin/carddisp.pl?gene=LTF
SOCS3	Suppressor Of Cytokine Signaling 3	Protein Coding	50	GC17M078356	4.479187489	https://www.genecards.org/cgi-bin/carddisp.pl?gene=SOCS3
COL2A1	Collagen Type II Alpha 1 Chain	Protein Coding	56	GC12M047972	4.477406502	https://www.genecards.org/cgi-bin/carddisp.pl?gene=COL2A1
WT1	WT1 Transcription Factor	Protein Coding	55	GC11M032365	4.475149155	https://www.genecards.org/cgi-bin/carddisp.pl?gene=WT1
LDHB	Lactate Dehydrogenase B	Protein Coding	53	GC12M021635	4.47364521	https://www.genecards.org/cgi-bin/carddisp.pl?gene=LDHB
ACSBG1	Acyl-CoA Synthetase Bubblegum Family Member 1	Protein Coding	48	GC15M078167	4.471997738	https://www.genecards.org/cgi-bin/carddisp.pl?gene=ACSBG1
SNAP25	Synaptosome Associated Protein 25	Protein Coding	55	GC20P010193	4.466922283	https://www.genecards.org/cgi-bin/carddisp.pl?gene=SNAP25
NME1	NME/NM23 Nucleoside Diphosphate Kinase 1	Protein Coding	51	GC17P062974	4.462903023	https://www.genecards.org/cgi-bin/carddisp.pl?gene=NME1

ADRA2A	Adrenoceptor Alpha 2A	Protein Coding	52	GC10P111077	4.449795246	https://www.genecards.org/cgi-bin/carddisp.pl?gene=ADRA2A
DYRK1A	Dual Specificity Tyrosine Phosphorylation Regulated Kinase 1A	Protein Coding	57	GC21P037365	4.449550152	https://www.genecards.org/cgi-bin/carddisp.pl?gene=DYRK1A
FASLG	Fas Ligand	Protein Coding	55	GC01P172628	4.443905354	https://www.genecards.org/cgi-bin/carddisp.pl?gene=FASLG
ACSL3	Acyl-CoA Synthetase Long Chain Family Member 3	Protein Coding	49	GC02P222860	4.439405918	https://www.genecards.org/cgi-bin/carddisp.pl?gene=ACSL3
AGER	Advanced Glycosylation End-Product Specific Receptor	Protein Coding	53	GC06M032180	4.438507557	https://www.genecards.org/cgi-bin/carddisp.pl?gene=AGER
CXCR4	C-X-C Motif Chemokine Receptor 4	Protein Coding	59	GC02M136114	4.436937332	https://www.genecards.org/cgi-bin/carddisp.pl?gene=CXCR4
CBL	Cbl Proto-Oncogene	Protein Coding	59	GC11P119206	4.430374146	https://www.genecards.org/cgi-bin/carddisp.pl?gene=CBL
THBS1	Thrombospondin 1	Protein Coding	52	GC15P039581	4.425669193	https://www.genecards.org/cgi-bin/carddisp.pl?gene=THBS1
IFNA1	Interferon Alpha 1	Protein Coding	45	GC09P021611	4.422715187	https://www.genecards.org/cgi-bin/carddisp.pl?gene=IFNA1
SRR	Serine Racemase	Protein Coding	48	GC17P002303	4.418429375	https://www.genecards.org/cgi-bin/carddisp.pl?gene=SRR
ADPRH	ADP-Ribosylarginine Hydrolase	Protein Coding	42	GC03P119579	4.417760849	https://www.genecards.org/cgi-bin/carddisp.pl?gene=ADPRH
CCN2	Cellular Communication Network Factor 2	Protein Coding	53	GC06M131948	4.404472828	https://www.genecards.org/cgi-bin/carddisp.pl?gene=CCN2
TGFA	Transforming Growth Factor Alpha	Protein Coding	53	GC02M070447	4.400311947	https://www.genecards.org/cgi-bin/carddisp.pl?gene=TGFA
PAX5	Paired Box 5	Protein Coding	52	GC09M036879	4.399440765	https://www.genecards.org/cgi-bin/carddisp.pl?gene=PAX5
RUNX1	RUNX Family Transcription Factor 1	Protein Coding	56	GC21M034787	4.396518707	https://www.genecards.org/cgi-bin/carddisp.pl?gene=RUNX1

ENG	Endoglin	Protein Coding	55	GC09M127815	4.393945217	https://www.genecards.org/cgi-bin/carddisp.pl?gene=ENG
PDGFB	Platelet Derived Growth Factor Subunit B	Protein Coding	58	GC22M062326	4.381302357	https://www.genecards.org/cgi-bin/carddisp.pl?gene=PDGFB
EDNRA	Endothelin Receptor Type A	Protein Coding	56	GC04P147480	4.378360271	https://www.genecards.org/cgi-bin/carddisp.pl?gene=EDNRA
SLC27A2	Solute Carrier Family 27 Member 2	Protein Coding	52	GC15P050182	4.3697896	https://www.genecards.org/cgi-bin/carddisp.pl?gene=SLC27A2
NCOA3	Nuclear Receptor Coactivator 3	Protein Coding	54	GC20P047501	4.368255615	https://www.genecards.org/cgi-bin/carddisp.pl?gene=NCOA3
CDK2	Cyclin Dependent Kinase 2	Protein Coding	58	GC12P055966	4.363362789	https://www.genecards.org/cgi-bin/carddisp.pl?gene=CDK2
GAL	Galanin And GMAP Prepropeptide	Protein Coding	51	GC11P071097	4.357871056	https://www.genecards.org/cgi-bin/carddisp.pl?gene=GAL
PLCG2	Phospholipase C Gamma 2	Protein Coding	58	GC16P081773	4.349803448	https://www.genecards.org/cgi-bin/carddisp.pl?gene=PLCG2
LTA	Lymphotoxin Alpha	Protein Coding	49	GC06P092152	4.348684311	https://www.genecards.org/cgi-bin/carddisp.pl?gene=LTA
CLU	Clusterin	Protein Coding	54	GC08M027596	4.345746994	https://www.genecards.org/cgi-bin/carddisp.pl?gene=CLU
MUC1	Mucin 1, Cell Surface Associated	Protein Coding	55	GC01M155185	4.336580753	https://www.genecards.org/cgi-bin/carddisp.pl?gene=MUC1
SMAD4	SMAD Family Member 4	Protein Coding	59	GC18P051028	4.335887909	https://www.genecards.org/cgi-bin/carddisp.pl?gene=SMAD4
FN1	Fibronectin 1	Protein Coding	57	GC02M215360	4.331896305	https://www.genecards.org/cgi-bin/carddisp.pl?gene=FN1
TGFBR1	Transforming Growth Factor Beta Receptor 1	Protein Coding	61	GC09P099104	4.330105305	https://www.genecards.org/cgi-bin/carddisp.pl?gene=TGFBR1
BSG	Basigin (Ok Blood Group)	Protein Coding	51	GC19P000571	4.324801445	https://www.genecards.org/cgi-bin/carddisp.pl?gene=BSG

	RRM2	Ribonucleotide Reductase Regulatory Subunit M2	Protein Coding	55	GC02P010123	4.324131966	https://www.genecards.org/cgi-bin/carddisp.pl?gene=RRM2
	PARK7	Parkinsonism Associated Deglycase	Protein Coding	54	GC01P008140	4.317549706	https://www.genecards.org/cgi-bin/carddisp.pl?gene=PARK7
	TGFBR2	Transforming Growth Factor Beta Receptor 2	Protein Coding	59	GC03P030623	4.308754444	https://www.genecards.org/cgi-bin/carddisp.pl?gene=TGFBR2
	HDAC2	Histone Deacetylase 2	Protein Coding	59	GC06M113933	4.305524826	https://www.genecards.org/cgi-bin/carddisp.pl?gene=HDAC2
	PRNP	Prion Protein	Protein Coding	55	GC20P004686	4.301037312	https://www.genecards.org/cgi-bin/carddisp.pl?gene=PRNP
	RRM1	Ribonucleotide Reductase Catalytic Subunit M1	Protein Coding	56	GC11P004115	4.299415112	https://www.genecards.org/cgi-bin/carddisp.pl?gene=RRM1
	PRKCD	Protein Kinase C Delta	Protein Coding	59	GC03P053156	4.297080994	https://www.genecards.org/cgi-bin/carddisp.pl?gene=PRKCD
	SLC26A4	Solute Carrier Family 26 Member 4	Protein Coding	49	GC07P107660	4.296963692	https://www.genecards.org/cgi-bin/carddisp.pl?gene=SLC26A4
	PRDX5	Peroxiredoxin 5	Protein Coding	51	GC11P064460	4.295131683	https://www.genecards.org/cgi-bin/carddisp.pl?gene=PRDX5
	SEMA4D	Semaphorin 4D	Protein Coding	53	GC09M089360	4.28213644	https://www.genecards.org/cgi-bin/carddisp.pl?gene=SEMA4D
	NKX2-1	NK2 Homeobox 1	Protein Coding	53	GC14M036516	4.278104782	https://www.genecards.org/cgi-bin/carddisp.pl?gene=NKX2-1
	NEU3	Neuraminidase 3	Protein Coding	45	GC11P078455	4.275635242	https://www.genecards.org/cgi-bin/carddisp.pl?gene=NEU3
	WRN	WRN RecQ Like Helicase	Protein Coding	53	GC08P031033	4.274279118	https://www.genecards.org/cgi-bin/carddisp.pl?gene=WRN
	TBK1	TANK Binding Kinase 1	Protein Coding	56	GC12P064451	4.270060539	https://www.genecards.org/cgi-bin/carddisp.pl?gene=TBK1
1	B4GALNT	Beta-1,4-N-Acetyl-Galactosaminyltransferase 1	Protein Coding	53	GC12M057623	4.263757229	https://www.genecards.org/cgi-bin/carddisp.pl?gene=B4GALNT1

CD19	CD19 Molecule	Protein Coding	58	GC16P042650	4.263446808	https://www.genecards.org/cgi-bin/carddisp.pl?gene=CD19
IKBKB	Inhibitor Of Nuclear Factor Kappa B Kinase Subunit Beta	Protein Coding	61	GC08P042271	4.261201859	https://www.genecards.org/cgi-bin/carddisp.pl?gene=IKBKB
ACOT12	Acyl-CoA Thioesterase 12	Protein Coding	44	GC05M081309	4.259821892	https://www.genecards.org/cgi-bin/carddisp.pl?gene=ACOT12
NRAS	NRAS Proto-Oncogene, GTPase	Protein Coding	57	GC01M114704	4.258516312	https://www.genecards.org/cgi-bin/carddisp.pl?gene=NRAS
ABCD2	ATP Binding Cassette Subfamily D Member 2	Protein Coding	47	GC12M039530	4.252993107	https://www.genecards.org/cgi-bin/carddisp.pl?gene=ABCD2
FAR1	Fatty Acyl-CoA Reductase 1	Protein Coding	49	GC11P013668	4.249789238	https://www.genecards.org/cgi-bin/carddisp.pl?gene=FAR1
SLC1A3	Solute Carrier Family 1 Member 3	Protein Coding	56	GC05P036654	4.249479294	https://www.genecards.org/cgi-bin/carddisp.pl?gene=SLC1A3
PTK2B	Protein Tyrosine Kinase 2 Beta	Protein Coding	55	GC08P027311	4.24893856	https://www.genecards.org/cgi-bin/carddisp.pl?gene=PTK2B
PRKG1	Protein Kinase CGMP-Dependent 1	Protein Coding	57	GC10P050991	4.246487141	https://www.genecards.org/cgi-bin/carddisp.pl?gene=PRKG1
PIKFYVE	Phosphoinositide Kinase, FYVE-Type Zinc Finger Containing	Protein Coding	55	GC02P208266	4.238278866	https://www.genecards.org/cgi-bin/carddisp.pl?gene=PIKFYVE
NTRK1	Neurotrophic Receptor Tyrosine Kinase 1	Protein Coding	57	GC01P156815	4.233635426	https://www.genecards.org/cgi-bin/carddisp.pl?gene=NTRK1
PLAU	Plasminogen Activator, Urokinase	Protein Coding	59	GC10P073909	4.2326684	https://www.genecards.org/cgi-bin/carddisp.pl?gene=PLAU
ITGAM	Integrin Subunit Alpha M	Protein Coding	55	GC16P042850	4.231232643	https://www.genecards.org/cgi-bin/carddisp.pl?gene=ITGAM
HPX	Hemopexin	Protein Coding	48	GC11M006939	4.224319935	https://www.genecards.org/cgi-bin/carddisp.pl?gene=HPX
VIP	Vasoactive Intestinal Peptide	Protein Coding	51	GC06P152750	4.223753452	https://www.genecards.org/cgi-bin/carddisp.pl?gene=VIP

SYNJ1	Synaptojanin 1	Protein Coding	52	GC21M032628	4.215205193	https://www.genecards.org/cgi-bin/carddisp.pl?gene=SYNJ1
PRSS1	Serine Protease 1	Protein Coding	51	GC07P149953	4.214827061	https://www.genecards.org/cgi-bin/carddisp.pl?gene=PRSS1
IDO2	Indoleamine 2,3-Dioxygenase 2	Protein Coding	45	GC08P039976	4.204556465	https://www.genecards.org/cgi-bin/carddisp.pl?gene=IDO2
OARD1	O-Acyl-ADP-Ribose Deacylase 1	Protein Coding	39	GC06M071913	4.190365314	https://www.genecards.org/cgi-bin/carddisp.pl?gene=OARD1
PSEN2	Presenilin 2	Protein Coding	56	GC01P226870	4.188182831	https://www.genecards.org/cgi-bin/carddisp.pl?gene=PSEN2
GPC3	Glypican 3	Protein Coding	53	GC0XM133535	4.185760498	https://www.genecards.org/cgi-bin/carddisp.pl?gene=GPC3
HDAC3	Histone Deacetylase 3	Protein Coding	56	GC05M141620	4.183124065	https://www.genecards.org/cgi-bin/carddisp.pl?gene=HDAC3
PTGES2	Prostaglandin E Synthase 2	Protein Coding	50	GC09M128120	4.17619276	https://www.genecards.org/cgi-bin/carddisp.pl?gene=PTGES2
PTPMT1	Protein Tyrosine Phosphatase Mitochondrial 1	Protein Coding	44	GC11P047568	4.171000481	https://www.genecards.org/cgi-bin/carddisp.pl?gene=PTPMT1
P4HB	Prolyl 4-Hydroxylase Subunit Beta	Protein Coding	56	GC17M081843	4.170737743	https://www.genecards.org/cgi-bin/carddisp.pl?gene=P4HB
XK	X-Linked Kx Blood Group Antigen, Kell And VPS13A Binding Protein	Protein Coding	47	GC0XP037685	4.168406487	https://www.genecards.org/cgi-bin/carddisp.pl?gene=XK
CDK4	Cyclin Dependent Kinase 4	Protein Coding	61	GC12M057787	4.165514946	https://www.genecards.org/cgi-bin/carddisp.pl?gene=CDK4
SPTLC3	Serine Palmitoyltransferase Long Chain Base Subunit 3	Protein Coding	46	GC20P013008	4.162569523	https://www.genecards.org/cgi-bin/carddisp.pl?gene=SPTLC3
XPC	XPC Complex Subunit, DNA Damage Recognition And Repair Factor	Protein Coding	52	GC03M021831	4.16207695	https://www.genecards.org/cgi-bin/carddisp.pl?gene=XPC
SLC1A1	Solute Carrier Family 1 Member 1	Protein Coding	56	GC09P004490	4.161443233	https://www.genecards.org/cgi-bin/carddisp.pl?gene=SLC1A1

OGT	O-Linked N-Acetylglucosamine (GlcNAc) Transferase	Protein Coding	51	GC0XP071534	4.159537315	https://www.genecards.org/cgi-bin/carddisp.pl?gene=OGT
SMAD3	SMAD Family Member 3	Protein Coding	59	GC15P067063	4.158631802	https://www.genecards.org/cgi-bin/carddisp.pl?gene=SMAD3
ELOVL1	ELOVL Fatty Acid Elongase 1	Protein Coding	48	GC01M043363	4.157157898	https://www.genecards.org/cgi-bin/carddisp.pl?gene=ELOVL1
TRPV4	Transient Receptor Potential Cation Channel Subfamily V Member 4	Protein Coding	56	GC12M109783	4.151106358	https://www.genecards.org/cgi-bin/carddisp.pl?gene=TRPV4
GSTA2	Glutathione S-Transferase Alpha 2	Protein Coding	47	GC06M052750	4.144515514	https://www.genecards.org/cgi-bin/carddisp.pl?gene=GSTA2
M6PR	Mannose-6-Phosphate Receptor, Cation Dependent	Protein Coding	50	GC12M009029	4.144075394	https://www.genecards.org/cgi-bin/carddisp.pl?gene=M6PR
SLCO2B1	Solute Carrier Organic Anion Transporter Family Member 2B1	Protein Coding	51	GC11P078457	4.143146515	https://www.genecards.org/cgi-bin/carddisp.pl?gene=SLCO2B1
ENO2	Enolase 2	Protein Coding	54	GC12P006913	4.141520023	https://www.genecards.org/cgi-bin/carddisp.pl?gene=ENO2
CD40	CD40 Molecule	Protein Coding	57	GC20P046118	4.141496658	https://www.genecards.org/cgi-bin/carddisp.pl?gene=CD40
NAT8L	N-Acetyltransferase 8 Like	Protein Coding	44	GC04P002061	4.136281013	https://www.genecards.org/cgi-bin/carddisp.pl?gene=NAT8L
CD8A	CD8a Molecule	Protein Coding	54	GC02M086784	4.132395267	https://www.genecards.org/cgi-bin/carddisp.pl?gene=CD8A
FGF19	Fibroblast Growth Factor 19	Protein Coding	49	GC11M099882	4.129551411	https://www.genecards.org/cgi-bin/carddisp.pl?gene=FGF19
ACAA2	Acetyl-CoA Acyltransferase 2	Protein Coding	51	GC18M049782	4.127201557	https://www.genecards.org/cgi-bin/carddisp.pl?gene=ACAA2
DCN	Decorin	Protein Coding	54	GC12M091140	4.126251698	https://www.genecards.org/cgi-bin/carddisp.pl?gene=DCN
XIAP	X-Linked Inhibitor Of Apoptosis	Protein Coding	59	GC0XP123859	4.12197876	https://www.genecards.org/cgi-bin/carddisp.pl?gene=XIAP

MDM2	MDM2 Proto-Oncogene	Protein Coding	59	GC12P068808	4.121714115	https://www.genecards.org/cgi-bin/carddisp.pl?gene=MDM2
OPRM1	Opioid Receptor Mu 1	Protein Coding	55	GC06P154123	4.121528625	https://www.genecards.org/cgi-bin/carddisp.pl?gene=OPRM1
FHIT	Fragile Histidine Triad Diadenosine Triphosphatase	Protein Coding	51	GC03M059747	4.118954182	https://www.genecards.org/cgi-bin/carddisp.pl?gene=FHIT
LRP6	LDL Receptor Related Protein 6	Protein Coding	55	GC12M022658	4.107475281	https://www.genecards.org/cgi-bin/carddisp.pl?gene=LRP6
SLC22A11	Solute Carrier Family 22 Member 11	Protein Coding	47	GC11P064573	4.107285976	https://www.genecards.org/cgi-bin/carddisp.pl?gene=SLC22A11
ACTA1	Actin Alpha 1, Skeletal Muscle	Protein Coding	55	GC01M229647	4.103856087	https://www.genecards.org/cgi-bin/carddisp.pl?gene=ACTA1
ITGB3	Integrin Subunit Beta 3	Protein Coding	59	GC17P062247	4.099756241	https://www.genecards.org/cgi-bin/carddisp.pl?gene=ITGB3
NPM1	Nucleophosmin 1	Protein Coding	57	GC05P171387	4.094702721	https://www.genecards.org/cgi-bin/carddisp.pl?gene=NPM1
SGK1	Serum/Glucocorticoid Regulated Kinase 1	Protein Coding	56	GC06M134169	4.088385105	https://www.genecards.org/cgi-bin/carddisp.pl?gene=SGK1
IL1R1	Interleukin 1 Receptor Type 1	Protein Coding	53	GC02P102136	4.08834362	https://www.genecards.org/cgi-bin/carddisp.pl?gene=IL1R1
DLG4	Discs Large MAGUK Scaffold Protein 4	Protein Coding	55	GC17M007226	4.085865021	https://www.genecards.org/cgi-bin/carddisp.pl?gene=DLG4
PRTN3	Proteinase 3	Protein Coding	52	GC19P000840	4.081354618	https://www.genecards.org/cgi-bin/carddisp.pl?gene=PRTN3
PPA1	Inorganic Pyrophosphatase 1	Protein Coding	50	GC10M070202	4.078914165	https://www.genecards.org/cgi-bin/carddisp.pl?gene=PPA1
PDHA2	Pyruvate Dehydrogenase E1 Subunit Alpha 2	Protein Coding	50	GC04P095840	4.076750755	https://www.genecards.org/cgi-bin/carddisp.pl?gene=PDHA2
OXT	Oxytocin/Neurophysin I Prepropeptide	Protein Coding	44	GC20P004488	4.070765495	https://www.genecards.org/cgi-bin/carddisp.pl?gene=OXT

LMNB1	Lamin B1	Protein Coding	53	GC05P126776	4.070357323	https://www.genecards.org/cgi-bin/carddisp.pl?gene=LMNB1
INPP5D	Inositol Polyphosphate-5-Phosphatase D	Protein Coding	51	GC02P233059	4.067728996	https://www.genecards.org/cgi-bin/carddisp.pl?gene=INPP5D
NCOR2	Nuclear Receptor Corepressor 2	Protein Coding	50	GC12M124324	4.064220428	https://www.genecards.org/cgi-bin/carddisp.pl?gene=NCOR2
XBP1	X-Box Binding Protein 1	Protein Coding	52	GC22M028794	4.061617851	https://www.genecards.org/cgi-bin/carddisp.pl?gene=XBP1
CHUK	Component Of Inhibitor Of Nuclear Factor Kappa B Kinase Complex	Protein Coding	58	GC10M100202	4.053830147	https://www.genecards.org/cgi-bin/carddisp.pl?gene=CHUK
SERPINA7	Serpin Family A Member 7	Protein Coding	48	GC0XM106032	4.048786163	https://www.genecards.org/cgi-bin/carddisp.pl?gene=SERPINA7
RB1	RB Transcriptional Corepressor 1	Protein Coding	55	GC13P048303	4.047911167	https://www.genecards.org/cgi-bin/carddisp.pl?gene=RB1
NOTCH1	Notch Receptor 1	Protein Coding	60	GC09M137729	4.046423912	https://www.genecards.org/cgi-bin/carddisp.pl?gene=NOTCH1
TSC1	TSC Complex Subunit 1	Protein Coding	54	GC09M132891	4.045608997	https://www.genecards.org/cgi-bin/carddisp.pl?gene=TSC1
CEBPB	CCAAT Enhancer Binding Protein Beta	Protein Coding	50	GC20P050190	4.044969082	https://www.genecards.org/cgi-bin/carddisp.pl?gene=CEBPB
PLD1	Phospholipase D1	Protein Coding	55	GC03M171600	4.041944981	https://www.genecards.org/cgi-bin/carddisp.pl?gene=PLD1
UGT2B10	UDP Glucuronosyltransferase Family 2 Member B10	Protein Coding	47	GC04P068816	4.030697823	https://www.genecards.org/cgi-bin/carddisp.pl?gene=UGT2B10
WDR45	WD Repeat Domain 45	Protein Coding	46	GC0XM049074	4.027177334	https://www.genecards.org/cgi-bin/carddisp.pl?gene=WDR45
RIPK1	Receptor Interacting Serine/Threonine Kinase 1	Protein Coding	56	GC06P003333	4.020352364	https://www.genecards.org/cgi-bin/carddisp.pl?gene=RIPK1
EIF2AK4	Eukaryotic Translation Initiation Factor 2 Alpha Kinase 4	Protein Coding	52	GC15P039934	4.020245075	https://www.genecards.org/cgi-bin/carddisp.pl?gene=EIF2AK4

CERS1	Ceramide Synthase 1	Protein Coding	49	GC19M018868	4.019072056	https://www.genecards.org/cgi-bin/carddisp.pl?gene=CERS1
ENTPD1	Ectonucleoside Triphosphate Diphosphohydrolase 1	Protein Coding	57	GC10P095711	4.01881361	https://www.genecards.org/cgi-bin/carddisp.pl?gene=ENTPD1
CYP51A1	Cytochrome P450 Family 51 Subfamily A Member 1	Protein Coding	50	GC07M092112	4.017743111	https://www.genecards.org/cgi-bin/carddisp.pl?gene=CYP51A1
MAP3K5	Mitogen-Activated Protein Kinase Kinase Kinase 5	Protein Coding	55	GC06M136557	4.017704487	https://www.genecards.org/cgi-bin/carddisp.pl?gene=MAP3K5
SLC36A1	Solute Carrier Family 36 Member 1	Protein Coding	46	GC05P151344	4.017536163	https://www.genecards.org/cgi-bin/carddisp.pl?gene=SLC36A1
HMMR	Hyaluronan Mediated Motility Receptor	Protein Coding	50	GC05P163480	4.016493797	https://www.genecards.org/cgi-bin/carddisp.pl?gene=HMMR
PRKCB	Protein Kinase C Beta	Protein Coding	55	GC16P024574	4.015511513	https://www.genecards.org/cgi-bin/carddisp.pl?gene=PRKCB
MOGAT2	Monoacylglycerol O-Acyltransferase 2	Protein Coding	43	GC11P075717	4.011301994	https://www.genecards.org/cgi-bin/carddisp.pl?gene=MOGAT2
APOM	Apolipoprotein M	Protein Coding	47	GC06P092161	3.997857571	https://www.genecards.org/cgi-bin/carddisp.pl?gene=APOM
THRB	Thyroid Hormone Receptor Beta	Protein Coding	56	GC03M024117	3.983554363	https://www.genecards.org/cgi-bin/carddisp.pl?gene=THRB
CKB	Creatine Kinase B	Protein Coding	51	GC14M103519	3.977226257	https://www.genecards.org/cgi-bin/carddisp.pl?gene=CKB
RAPSN	Receptor Associated Protein Of The Synapse	Protein Coding	48	GC11M099370	3.971043825	https://www.genecards.org/cgi-bin/carddisp.pl?gene=RAPSN
GRP	Gastrin Releasing Peptide	Protein Coding	47	GC18P059220	3.969022274	https://www.genecards.org/cgi-bin/carddisp.pl?gene=GRP
KCNMA1	Potassium Calcium-Activated Channel Subfamily M Alpha 1	Protein Coding	56	GC10M076869	3.950283051	https://www.genecards.org/cgi-bin/carddisp.pl?gene=KCNMA1
KLK3	Kallikrein Related Peptidase 3	Protein Coding	53	GC19P050854	3.946335554	https://www.genecards.org/cgi-bin/carddisp.pl?gene=KLK3

CDK5	Cyclin Dependent Kinase 5	Protein Coding	60	GC07M151053	3.944981575	https://www.genecards.org/cgi-bin/carddisp.pl?gene=CDK5
SLC12A5	Solute Carrier Family 12 Member 5	Protein Coding	55	GC20P046021	3.943710566	https://www.genecards.org/cgi-bin/carddisp.pl?gene=SLC12A5
CSF3	Colony Stimulating Factor 3	Protein Coding	47	GC17P040015	3.937470913	https://www.genecards.org/cgi-bin/carddisp.pl?gene=CSF3
PDE3B	Phosphodiesterase 3B	Protein Coding	50	GC11P014643	3.933916092	https://www.genecards.org/cgi-bin/carddisp.pl?gene=PDE3B
IFNA2	Interferon Alpha 2	Protein Coding	50	GC09M021384	3.93334651	https://www.genecards.org/cgi-bin/carddisp.pl?gene=IFNA2
SLC1A4	Solute Carrier Family 1 Member 4	Protein Coding	52	GC02P064988	3.931900501	https://www.genecards.org/cgi-bin/carddisp.pl?gene=SLC1A4
AKT3	AKT Serine/Threonine Kinase 3	Protein Coding	61	GC01M243488	3.922803879	https://www.genecards.org/cgi-bin/carddisp.pl?gene=AKT3
H19	H19 Imprinted Maternally Expressed Transcript	RNA Gene	30	GC11M001995	3.921946287	https://www.genecards.org/cgi-bin/carddisp.pl?gene=H19
CTLA4	Cytotoxic T-Lymphocyte Associated Protein 4	Protein Coding	54	GC02P203854	3.916200161	https://www.genecards.org/cgi-bin/carddisp.pl?gene=CTLA4
RAC1	Rac Family Small GTPase 1	Protein Coding	55	GC07P006377	3.913594246	https://www.genecards.org/cgi-bin/carddisp.pl?gene=RAC1
H4C16	H4 Histone 16	Protein Coding	42	GC12M022801	3.911123037	https://www.genecards.org/cgi-bin/carddisp.pl?gene=H4C16
GPX3	Glutathione Peroxidase 3	Protein Coding	47	GC05P150997	3.910354376	https://www.genecards.org/cgi-bin/carddisp.pl?gene=GPX3
MYO5B	Myosin VB	Protein Coding	50	GC18M049822	3.909657717	https://www.genecards.org/cgi-bin/carddisp.pl?gene=MYO5B
STUB1	STIP1 Homology And U-Box Containing Protein 1	Protein Coding	53	GC16P013453	3.907438517	https://www.genecards.org/cgi-bin/carddisp.pl?gene=STUB1
E2F1	E2F Transcription Factor 1	Protein Coding	50	GC20M033675	3.905562401	https://www.genecards.org/cgi-bin/carddisp.pl?gene=E2F1

IL15	Interleukin 15	Protein Coding	49	GC04P141636	3.902137041	https://www.genecards.org/cgi-bin/carddisp.pl?gene=IL15
HTR2A	5-Hydroxytryptamine Receptor 2A	Protein Coding	55	GC13M046831	3.901376009	https://www.genecards.org/cgi-bin/carddisp.pl?gene=HTR2A
LPO	Lactoperoxidase	Protein Coding	46	GC17P058218	3.900479317	https://www.genecards.org/cgi-bin/carddisp.pl?gene=LPO
LAMP1	Lysosomal Associated Membrane Protein 1	Protein Coding	50	GC13P113297	3.897934437	https://www.genecards.org/cgi-bin/carddisp.pl?gene=LAMP1
IKBKG	Inhibitor Of Nuclear Factor Kappa B Kinase Regulatory Subunit Gamma	Protein Coding	55	GC0XP154541	3.894810677	https://www.genecards.org/cgi-bin/carddisp.pl?gene=IKBKG
ADM	Adrenomedullin	Protein Coding	51	GC11P010304	3.88862443	https://www.genecards.org/cgi-bin/carddisp.pl?gene=ADM
MSH6	MutS Homolog 6	Protein Coding	55	GC02P047695	3.887536764	https://www.genecards.org/cgi-bin/carddisp.pl?gene=MSH6
CA9	Carbonic Anhydrase 9	Protein Coding	53	GC09P035673	3.883813143	https://www.genecards.org/cgi-bin/carddisp.pl?gene=CA9
TUBB1	Tubulin Beta 1 Class VI	Protein Coding	53	GC20P059020	3.882990837	https://www.genecards.org/cgi-bin/carddisp.pl?gene=TUBB1
GATA3	GATA Binding Protein 3	Protein Coding	56	GC10P008045	3.879091263	https://www.genecards.org/cgi-bin/carddisp.pl?gene=GATA3
GRB2	Growth Factor Receptor Bound Protein 2	Protein Coding	52	GC17M075318	3.878803253	https://www.genecards.org/cgi-bin/carddisp.pl?gene=GRB2
ESRRB	Estrogen Related Receptor Beta	Protein Coding	54	GC14P076310	3.874730587	https://www.genecards.org/cgi-bin/carddisp.pl?gene=ESRRB
SERPINF1	Serpin Family F Member 1	Protein Coding	53	GC17P001766	3.874513626	https://www.genecards.org/cgi-bin/carddisp.pl?gene=SERPINF1
IL7R	Interleukin 7 Receptor	Protein Coding	52	GC05P035852	3.873098135	https://www.genecards.org/cgi-bin/carddisp.pl?gene=IL7R
CD55	CD55 Molecule (Cromer Blood Group)	Protein Coding	56	GC01P207321	3.870641232	https://www.genecards.org/cgi-bin/carddisp.pl?gene=CD55

NPR3	Natriuretic Peptide Receptor 3	Protein Coding	52	GC05P032689	3.868728161	https://www.genecards.org/cgi-bin/carddisp.pl?gene=NPR3
HNRNPC	Heterogeneous Nuclear Ribonucleoprotein C	Protein Coding	48	GC14M023657	3.863192081	https://www.genecards.org/cgi-bin/carddisp.pl?gene=HNRNPC
PTK2	Protein Tyrosine Kinase 2	Protein Coding	55	GC08M140657	3.861723185	https://www.genecards.org/cgi-bin/carddisp.pl?gene=PTK2
NME2	NME/NM23 Nucleoside Diphosphate Kinase 2	Protein Coding	52	GC17P051165	3.860429049	https://www.genecards.org/cgi-bin/carddisp.pl?gene=NME2
SLC16A10	Solute Carrier Family 16 Member 10	Protein Coding	44	GC06P111087	3.857141495	https://www.genecards.org/cgi-bin/carddisp.pl?gene=SLC16A10
IRAK1	Interleukin 1 Receptor Associated Kinase 1	Protein Coding	56	GC0XM154010	3.856891394	https://www.genecards.org/cgi-bin/carddisp.pl?gene=IRAK1
IGFBP5	Insulin Like Growth Factor Binding Protein 5	Protein Coding	49	GC02M216672	3.853482246	https://www.genecards.org/cgi-bin/carddisp.pl?gene=IGFBP5
KHDRBS1	KH RNA Binding Domain Containing, Signal Transduction Associated 1	Protein Coding	47	GC01P032013	3.852282047	https://www.genecards.org/cgi-bin/carddisp.pl?gene=KHDRBS1
GARS1	Glycyl-TRNA Synthetase 1	Protein Coding	52	GC07P030580	3.85188961	https://www.genecards.org/cgi-bin/carddisp.pl?gene=GARS1
ACSM5	Acyl-CoA Synthetase Medium Chain Family Member 5	Protein Coding	44	GC16P020410	3.850166559	https://www.genecards.org/cgi-bin/carddisp.pl?gene=ACSM5
FGA	Fibrinogen Alpha Chain	Protein Coding	56	GC04M154583	3.84565568	https://www.genecards.org/cgi-bin/carddisp.pl?gene=FGA
ADCYAP1	Adenylate Cyclase Activating Polypeptide 1	Protein Coding	47	GC18P000895	3.83665657	https://www.genecards.org/cgi-bin/carddisp.pl?gene=ADCYAP1
KEAP1	Kelch Like ECH Associated Protein 1	Protein Coding	56	GC19M010486	3.835975885	https://www.genecards.org/cgi-bin/carddisp.pl?gene=KEAP1
PDE4A	Phosphodiesterase 4A	Protein Coding	51	GC19P010416	3.835870028	https://www.genecards.org/cgi-bin/carddisp.pl?gene=PDE4A
CCKAR	Cholecystokinin A Receptor	Protein Coding	52	GC04M026483	3.835656404	https://www.genecards.org/cgi-bin/carddisp.pl?gene=CCKAR

PRKACG	Protein Kinase CAMP-Activated Catalytic Subunit Gamma	Protein Coding	53	GC09M069046	3.832050562	https://www.genecards.org/cgi-bin/carddisp.pl?gene=PRKACG
ACSL5	Acyl-CoA Synthetase Long Chain Family Member 5	Protein Coding	49	GC10P112374	3.831627607	https://www.genecards.org/cgi-bin/carddisp.pl?gene=ACSL5
PNMT	Phenylethanolamine N-Methyltransferase	Protein Coding	50	GC17P039667	3.831362724	https://www.genecards.org/cgi-bin/carddisp.pl?gene=PNMT
CGB5	Chorionic Gonadotropin Subunit Beta 5	Protein Coding	37	GC19P049043	3.828410149	https://www.genecards.org/cgi-bin/carddisp.pl?gene=CGB5
GUK1	Guanylate Kinase 1	Protein Coding	47	GC01P228139	3.826385498	https://www.genecards.org/cgi-bin/carddisp.pl?gene=GUK1
AGMO	Alkylglycerol Monooxygenase	Protein Coding	42	GC07M015117	3.824967861	https://www.genecards.org/cgi-bin/carddisp.pl?gene=AGMO
SYK	Spleen Associated Tyrosine Kinase	Protein Coding	56	GC09P092704	3.822367668	https://www.genecards.org/cgi-bin/carddisp.pl?gene=SYK
CERS6	Ceramide Synthase 6	Protein Coding	47	GC02P168455	3.821056366	https://www.genecards.org/cgi-bin/carddisp.pl?gene=CERS6
SEC14L2	SEC14 Like Lipid Binding 2	Protein Coding	46	GC22P030396	3.81583333	https://www.genecards.org/cgi-bin/carddisp.pl?gene=SEC14L2
SORT1	Sortilin 1	Protein Coding	51	GC01M109310	3.81338954	https://www.genecards.org/cgi-bin/carddisp.pl?gene=SORT1
RARB	Retinoic Acid Receptor Beta	Protein Coding	56	GC03P024689	3.813275337	https://www.genecards.org/cgi-bin/carddisp.pl?gene=RARB
LRP8	LDL Receptor Related Protein 8	Protein Coding	50	GC01M053243	3.812624216	https://www.genecards.org/cgi-bin/carddisp.pl?gene=LRP8
ITGB1	Integrin Subunit Beta 1	Protein Coding	57	GC10M033645	3.809351444	https://www.genecards.org/cgi-bin/carddisp.pl?gene=ITGB1
SRY	Sex Determining Region Y	Protein Coding	42	GC0YM002698	3.805636406	https://www.genecards.org/cgi-bin/carddisp.pl?gene=SRY
MGST1	Microsomal Glutathione S-Transferase 1	Protein Coding	47	GC12P016347	3.804653168	https://www.genecards.org/cgi-bin/carddisp.pl?gene=MGST1

CANX	Calnexin	Protein Coding	51	GC05P179678	3.798567057	https://www.genecards.org/cgi-bin/carddisp.pl?gene=CANX
PLA2G2E	Phospholipase A2 Group IIE	Protein Coding	43	GC01M019920	3.789566994	https://www.genecards.org/cgi-bin/carddisp.pl?gene=PLA2G2E
SULT1B1	Sulfotransferase Family 1B Member 1	Protein Coding	47	GC04M069721	3.787377834	https://www.genecards.org/cgi-bin/carddisp.pl?gene=SULT1B1
NR4A2	Nuclear Receptor Subfamily 4 Group A Member 2	Protein Coding	53	GC02M156324	3.786156893	https://www.genecards.org/cgi-bin/carddisp.pl?gene=NR4A2
SMN2	Survival Of Motor Neuron 2, Centromeric	Protein Coding	46	GC05P070049	3.781511784	https://www.genecards.org/cgi-bin/carddisp.pl?gene=SMN2
BTBD10	BTB Domain Containing 10	Protein Coding	43	GC11M013387	3.778814316	https://www.genecards.org/cgi-bin/carddisp.pl?gene=BTBD10
ADAR	Adenosine Deaminase RNA Specific	Protein Coding	52	GC01M154599	3.766012192	https://www.genecards.org/cgi-bin/carddisp.pl?gene=ADAR
CTSK	Cathepsin K	Protein Coding	57	GC01M152758	3.764894962	https://www.genecards.org/cgi-bin/carddisp.pl?gene=CTSK
TRAF6	TNF Receptor Associated Factor 6	Protein Coding	52	GC11M036467	3.761487722	https://www.genecards.org/cgi-bin/carddisp.pl?gene=TRAF6
CNR2	Cannabinoid Receptor 2	Protein Coding	51	GC01M023870	3.759726048	https://www.genecards.org/cgi-bin/carddisp.pl?gene=CNR2
DGKA	Diacylglycerol Kinase Alpha	Protein Coding	51	GC12P055927	3.755910397	https://www.genecards.org/cgi-bin/carddisp.pl?gene=DGKA
CYP3A43	Cytochrome P450 Family 3 Subfamily A Member 43	Protein Coding	46	GC07P099833	3.754785776	https://www.genecards.org/cgi-bin/carddisp.pl?gene=CYP3A43
DRD4	Dopamine Receptor D4	Protein Coding	52	GC11P002096	3.753496647	https://www.genecards.org/cgi-bin/carddisp.pl?gene=DRD4
PCSK2	Proprotein Convertase Subtilisin/Kexin Type 2	Protein Coding	50	GC20P017226	3.745601177	https://www.genecards.org/cgi-bin/carddisp.pl?gene=PCSK2
STAT5B	Signal Transducer And Activator Of Transcription 5B	Protein Coding	56	GC17M042199	3.744742155	https://www.genecards.org/cgi-bin/carddisp.pl?gene=STAT5B

TJP2	Tight Junction Protein 2	Protein Coding	51	GC09P069121	3.744441032	https://www.genecards.org/cgi-bin/carddisp.pl?gene=TJP2
KCNJ2	Potassium Inwardly Rectifying Channel Subfamily J Member 2	Protein Coding	55	GC17P070168	3.729247332	https://www.genecards.org/cgi-bin/carddisp.pl?gene=KCNJ2
GPAT3	Glycerol-3-Phosphate Acyltransferase 3	Protein Coding	47	GC04P083536	3.729138851	https://www.genecards.org/cgi-bin/carddisp.pl?gene=GPAT3
SIK2	Salt Inducible Kinase 2	Protein Coding	52	GC11P111989	3.725149632	https://www.genecards.org/cgi-bin/carddisp.pl?gene=SIK2
TWIST1	Twist Family BHLH Transcription Factor 1	Protein Coding	52	GC07M019020	3.721744061	https://www.genecards.org/cgi-bin/carddisp.pl?gene=TWIST1
GLRX	Glutaredoxin	Protein Coding	50	GC05M095752	3.718975544	https://www.genecards.org/cgi-bin/carddisp.pl?gene=GLRX
PAM	Peptidylglycine Alpha-Amidating Monooxygenase	Protein Coding	50	GC05P102753	3.717186213	https://www.genecards.org/cgi-bin/carddisp.pl?gene=PAM
SELL	Selectin L	Protein Coding	50	GC01M169690	3.716027021	https://www.genecards.org/cgi-bin/carddisp.pl?gene=SELL
SULT4A1	Sulfotransferase Family 4A Member 1	Protein Coding	46	GC22M043824	3.715360165	https://www.genecards.org/cgi-bin/carddisp.pl?gene=SULT4A1
HNRNPD	Heterogeneous Nuclear Ribonucleoprotein D	Protein Coding	50	GC04M082352	3.702080965	https://www.genecards.org/cgi-bin/carddisp.pl?gene=HNRNPD
CERS2	Ceramide Synthase 2	Protein Coding	48	GC01M150960	3.701366425	https://www.genecards.org/cgi-bin/carddisp.pl?gene=CERS2
GPAT2	Glycerol-3-Phosphate Acyltransferase 2, Mitochondrial	Protein Coding	39	GC02M098745	3.699226379	https://www.genecards.org/cgi-bin/carddisp.pl?gene=GPAT2
CASP1	Caspase 1	Protein Coding	56	GC11M105025	3.698204041	https://www.genecards.org/cgi-bin/carddisp.pl?gene=CASP1
CAV2	Caveolin 2	Protein Coding	50	GC07P116287	3.696254253	https://www.genecards.org/cgi-bin/carddisp.pl?gene=CAV2
PSAT1	Phosphoserine Aminotransferase 1	Protein Coding	54	GC09P078297	3.693089008	https://www.genecards.org/cgi-bin/carddisp.pl?gene=PSAT1

TGM2	Transglutaminase 2	Protein Coding	54	GC20M038127	3.692184448	https://www.genecards.org/cgi-bin/carddisp.pl?gene=TGM2
TLR3	Toll Like Receptor 3	Protein Coding	58	GC04P186059	3.689142704	https://www.genecards.org/cgi-bin/carddisp.pl?gene=TLR3
WARS1	Tryptophanyl-TRNA Synthetase 1	Protein Coding	52	GC14M103181	3.687915325	https://www.genecards.org/cgi-bin/carddisp.pl?gene=WARS1
GSTA4	Glutathione S-Transferase Alpha 4	Protein Coding	47	GC06M052977	3.68756938	https://www.genecards.org/cgi-bin/carddisp.pl?gene=GSTA4
HSD17B12	Hydroxysteroid 17-Beta Dehydrogenase 12	Protein Coding	46	GC11P043652	3.686990738	https://www.genecards.org/cgi-bin/carddisp.pl?gene=HSD17B12
LIPF	Lipase F, Gastric Type	Protein Coding	49	GC10P088664	3.683813572	https://www.genecards.org/cgi-bin/carddisp.pl?gene=LIPF
SCD5	Stearoyl-CoA Desaturase 5	Protein Coding	48	GC04M082629	3.682816505	https://www.genecards.org/cgi-bin/carddisp.pl?gene=SCD5
PTGES3	Prostaglandin E Synthase 3	Protein Coding	51	GC12M057070	3.682389259	https://www.genecards.org/cgi-bin/carddisp.pl?gene=PTGES3
AZIN2	Antizyme Inhibitor 2	Protein Coding	47	GC01P033081	3.678210258	https://www.genecards.org/cgi-bin/carddisp.pl?gene=AZIN2
BMP4	Bone Morphogenetic Protein 4	Protein Coding	55	GC14M053949	3.675657272	https://www.genecards.org/cgi-bin/carddisp.pl?gene=BMP4
PLCB4	Phospholipase C Beta 4	Protein Coding	52	GC20P009024	3.674196005	https://www.genecards.org/cgi-bin/carddisp.pl?gene=PLCB4
MBL2	Mannose Binding Lectin 2	Protein Coding	53	GC10M052760	3.673710585	https://www.genecards.org/cgi-bin/carddisp.pl?gene=MBL2
AGTR2	Angiotensin II Receptor Type 2	Protein Coding	51	GC0XP116170	3.668705702	https://www.genecards.org/cgi-bin/carddisp.pl?gene=AGTR2
RPL11	Ribosomal Protein L11	Protein Coding	55	GC01P023691	3.668517351	https://www.genecards.org/cgi-bin/carddisp.pl?gene=RPL11
TRPV1	Transient Receptor Potential Cation Channel Subfamily V Member 1	Protein Coding	55	GC17M003565	3.657725573	https://www.genecards.org/cgi-bin/carddisp.pl?gene=TRPV1

CNTF	Ciliary Neurotrophic Factor	Protein Coding	47	GC11P058622	3.656933069	https://www.genecards.org/cgi-bin/carddisp.pl?gene=CNTF
IL2RA	Interleukin 2 Receptor Subunit Alpha	Protein Coding	57	GC10M006010	3.654792786	https://www.genecards.org/cgi-bin/carddisp.pl?gene=IL2RA
ACSM4	Acyl-CoA Synthetase Medium Chain Family Member 4	Protein Coding	38	GC12P007304	3.649587154	https://www.genecards.org/cgi-bin/carddisp.pl?gene=ACSM4
EIF4EBP1	Eukaryotic Translation Initiation Factor 4E Binding Protein 1	Protein Coding	53	GC08P038607	3.646658659	https://www.genecards.org/cgi-bin/carddisp.pl?gene=EIF4EBP1
BIRC5	Baculoviral IAP Repeat Containing 5	Protein Coding	52	GC17P078214	3.645214796	https://www.genecards.org/cgi-bin/carddisp.pl?gene=BIRC5
CALM1	Calmodulin 1	Protein Coding	52	GC14P090396	3.641564131	https://www.genecards.org/cgi-bin/carddisp.pl?gene=CALM1
ETV6	ETS Variant Transcription Factor 6	Protein Coding	52	GC12P011649	3.639504433	https://www.genecards.org/cgi-bin/carddisp.pl?gene=ETV6
BMPR2	Bone Morphogenetic Protein Receptor Type 2	Protein Coding	57	GC02P202376	3.636691809	https://www.genecards.org/cgi-bin/carddisp.pl?gene=BMPR2
IL1RAPL2	Interleukin 1 Receptor Accessory Protein Like 2	Protein Coding	46	GC0XP104566	3.632210255	https://www.genecards.org/cgi-bin/carddisp.pl?gene=IL1RAPL2
SLC6A2	Solute Carrier Family 6 Member 2	Protein Coding	54	GC16P055656	3.631602526	https://www.genecards.org/cgi-bin/carddisp.pl?gene=SLC6A2
PCBP2	Poly(RC) Binding Protein 2	Protein Coding	48	GC12P053452	3.630653381	https://www.genecards.org/cgi-bin/carddisp.pl?gene=PCBP2
PIK3R2	Phosphoinositide-3-Kinase Regulatory Subunit 2	Protein Coding	56	GC19P018153	3.626128674	https://www.genecards.org/cgi-bin/carddisp.pl?gene=PIK3R2
ACOT2	Acyl-CoA Thioesterase 2	Protein Coding	44	GC14P073567	3.625527143	https://www.genecards.org/cgi-bin/carddisp.pl?gene=ACOT2
ACP3	Acid Phosphatase 3	Protein Coding	50	GC03P135034	3.619726658	https://www.genecards.org/cgi-bin/carddisp.pl?gene=ACP3
C4A	Complement C4A (Rodgers Blood Group)	Protein Coding	50	GC06P092175	3.611579657	https://www.genecards.org/cgi-bin/carddisp.pl?gene=C4A

GPAT4	Glycerol-3-Phosphate Acyltransferase 4	Protein Coding	44	GC08P041577	3.610387325	https://www.genecards.org/cgi-bin/carddisp.pl?gene=GPAT4
THPO	Thrombopoietin	Protein Coding	50	GC03M184371	3.601413965	https://www.genecards.org/cgi-bin/carddisp.pl?gene=THPO
HSD17B8	Hydroxysteroid 17-Beta Dehydrogenase 8	Protein Coding	46	GC06P092209	3.600556135	https://www.genecards.org/cgi-bin/carddisp.pl?gene=HSD17B8
PGAM1	Phosphoglycerate Mutase 1	Protein Coding	51	GC10P097426	3.594974279	https://www.genecards.org/cgi-bin/carddisp.pl?gene=PGAM1
ANXA1	Annexin A1	Protein Coding	54	GC09P073151	3.584882736	https://www.genecards.org/cgi-bin/carddisp.pl?gene=ANXA1
CYP20A1	Cytochrome P450 Family 20 Subfamily A Member 1	Protein Coding	44	GC02P203238	3.581699133	https://www.genecards.org/cgi-bin/carddisp.pl?gene=CYP20A1
CD79B	CD79b Molecule	Protein Coding	54	GC17M063928	3.581664562	https://www.genecards.org/cgi-bin/carddisp.pl?gene=CD79B
PLEK	Pleckstrin	Protein Coding	47	GC02P068365	3.565039635	https://www.genecards.org/cgi-bin/carddisp.pl?gene=PLEK
UGT2B11	UDP Glucuronosyltransferase Family 2 Member B11	Protein Coding	44	GC04M069199	3.564004183	https://www.genecards.org/cgi-bin/carddisp.pl?gene=UGT2B11
NOX4	NADPH Oxidase 4	Protein Coding	50	GC11M089324	3.563686609	https://www.genecards.org/cgi-bin/carddisp.pl?gene=NOX4
ATP2A3	ATPase Sarcoplasmic/Endoplasmic Reticulum Ca2+ Transporting 3	Protein Coding	52	GC17M003923	3.559276819	https://www.genecards.org/cgi-bin/carddisp.pl?gene=ATP2A3
SOCS1	Suppressor Of Cytokine Signaling 1	Protein Coding	52	GC16M012467	3.558186293	https://www.genecards.org/cgi-bin/carddisp.pl?gene=SOCS1
TRH	Thyrotropin Releasing Hormone	Protein Coding	48	GC03P129974	3.556975603	https://www.genecards.org/cgi-bin/carddisp.pl?gene=TRH
CA1	Carbonic Anhydrase 1	Protein Coding	54	GC08M085327	3.556869507	https://www.genecards.org/cgi-bin/carddisp.pl?gene=CA1
NR4A3	Nuclear Receptor Subfamily 4 Group A Member 3	Protein Coding	49	GC09P099821	3.555757046	https://www.genecards.org/cgi-bin/carddisp.pl?gene=NR4A3

PRDX6	Peroxiredoxin 6	Protein Coding	53	GC01P173477	3.552488565	https://www.genecards.org/cgi-bin/carddisp.pl?gene=PRDX6
SH2B1	SH2B Adaptor Protein 1	Protein Coding	48	GC16P042648	3.546960115	https://www.genecards.org/cgi-bin/carddisp.pl?gene=SH2B1
GRIK2	Glutamate Ionotropic Receptor Kainate Type Subunit 2	Protein Coding	56	GC06P100962	3.546540976	https://www.genecards.org/cgi-bin/carddisp.pl?gene=GRIK2
SPARC	Secreted Protein Acidic And Cysteine Rich	Protein Coding	56	GC05M151661	3.546322346	https://www.genecards.org/cgi-bin/carddisp.pl?gene=SPARC
TGFB2	Transforming Growth Factor Beta 2	Protein Coding	58	GC01P218345	3.546247482	https://www.genecards.org/cgi-bin/carddisp.pl?gene=TGFB2
SCT	Secretin	Protein Coding	39	GC11M000626	3.542740107	https://www.genecards.org/cgi-bin/carddisp.pl?gene=SCT
ACSM6	Acyl-CoA Synthetase Medium Chain Family Member 6	Protein Coding	37	GC10P095194	3.540477037	https://www.genecards.org/cgi-bin/carddisp.pl?gene=ACSM6
STAT5A	Signal Transducer And Activator Of Transcription 5A	Protein Coding	55	GC17P042287	3.540235758	https://www.genecards.org/cgi-bin/carddisp.pl?gene=STAT5A
ELOVL6	ELOVL Fatty Acid Elongase 6	Protein Coding	48	GC04M110045	3.538087368	https://www.genecards.org/cgi-bin/carddisp.pl?gene=ELOVL6
TBXA2R	Thromboxane A2 Receptor	Protein Coding	56	GC19M003594	3.5368433	https://www.genecards.org/cgi-bin/carddisp.pl?gene=TBXA2R
LYN	LYN Proto-Oncogene, Src Family Tyrosine Kinase	Protein Coding	55	GC08P055879	3.53446126	https://www.genecards.org/cgi-bin/carddisp.pl?gene=LYN
AKR1B15	Aldo-Keto Reductase Family 1 Member B15	Protein Coding	37	GC07P134549	3.529265165	https://www.genecards.org/cgi-bin/carddisp.pl?gene=AKR1B15
F8	Coagulation Factor VIII	Protein Coding	54	GC0XM154835	3.527348995	https://www.genecards.org/cgi-bin/carddisp.pl?gene=F8
LGALS1	Galectin 1	Protein Coding	50	GC22P037675	3.524164677	https://www.genecards.org/cgi-bin/carddisp.pl?gene=LGALS1
UGT2B28	UDP Glucuronosyltransferase Family 2 Member B28	Protein Coding	45	GC04P069280	3.52409482	https://www.genecards.org/cgi-bin/carddisp.pl?gene=UGT2B28

SLC27A5	Solute Carrier Family 27 Member 5	Protein Coding	48	GC19M058479	3.523803711	https://www.genecards.org/cgi-bin/carddisp.pl?gene=SLC27A5
LGALS3	Galectin 3	Protein Coding	52	GC14P055124	3.522946119	https://www.genecards.org/cgi-bin/carddisp.pl?gene=LGALS3
EEF2	Eukaryotic Translation Elongation Factor 2	Protein Coding	55	GC19M003976	3.522862434	https://www.genecards.org/cgi-bin/carddisp.pl?gene=EEF2
CGA	Glycoprotein Hormones, Alpha Polypeptide	Protein Coding	51	GC06M087085	3.522000551	https://www.genecards.org/cgi-bin/carddisp.pl?gene=CGA
HAO1	Hydroxyacid Oxidase 1	Protein Coding	48	GC20M008508	3.519673824	https://www.genecards.org/cgi-bin/carddisp.pl?gene=HAO1
CD34	CD34 Molecule	Protein Coding	52	GC01M207880	3.518568277	https://www.genecards.org/cgi-bin/carddisp.pl?gene=CD34
TOP1	DNA Topoisomerase I	Protein Coding	55	GC20P041028	3.516348362	https://www.genecards.org/cgi-bin/carddisp.pl?gene=TOP1
PSMC6	Proteasome 26S Subunit, ATPase 6	Protein Coding	46	GC14P052707	3.516238689	https://www.genecards.org/cgi-bin/carddisp.pl?gene=PSMC6
HAS2	Hyaluronan Synthase 2	Protein Coding	47	GC08M121594	3.513864279	https://www.genecards.org/cgi-bin/carddisp.pl?gene=HAS2
HSPA1A	Heat Shock Protein Family A (Hsp70) Member 1A	Protein Coding	53	GC06P092171	3.513021469	https://www.genecards.org/cgi-bin/carddisp.pl?gene=HSPA1A
DLK1	Delta Like Non-Canonical Notch Ligand 1	Protein Coding	51	GC14P110708	3.512380123	https://www.genecards.org/cgi-bin/carddisp.pl?gene=DLK1
UBC	Ubiquitin C	Protein Coding	49	GC12M124911	3.51091814	https://www.genecards.org/cgi-bin/carddisp.pl?gene=UBC
AGRN	Agrin	Protein Coding	52	GC01P001020	3.509435892	https://www.genecards.org/cgi-bin/carddisp.pl?gene=AGRN
CA8	Carbonic Anhydrase 8	Protein Coding	53	GC08M060187	3.495261669	https://www.genecards.org/cgi-bin/carddisp.pl?gene=CA8
HNRNPU	Heterogeneous Nuclear Ribonucleoprotein U	Protein Coding	50	GC01M244844	3.492166042	https://www.genecards.org/cgi-bin/carddisp.pl?gene=HNRNPU

TOP2A	DNA Topoisomerase II Alpha	Protein Coding	57	GC17M040388	3.486743212	https://www.genecards.org/cgi-bin/carddisp.pl?gene=TOP2A
TNFSF10	TNF Superfamily Member 10	Protein Coding	51	GC03M172505	3.47996521	https://www.genecards.org/cgi-bin/carddisp.pl?gene=TNFSF10
INPP5J	Inositol Polyphosphate-5-Phosphatase J	Protein Coding	48	GC22P044897	3.474000692	https://www.genecards.org/cgi-bin/carddisp.pl?gene=INPP5J
SLC22A1	Solute Carrier Family 22 Member 1	Protein Coding	51	GC06P160121	3.473587513	https://www.genecards.org/cgi-bin/carddisp.pl?gene=SLC22A1
HMGB1	High Mobility Group Box 1	Protein Coding	54	GC13M030456	3.468208075	https://www.genecards.org/cgi-bin/carddisp.pl?gene=HMGB1
MYH9	Myosin Heavy Chain 9	Protein Coding	55	GC22M036281	3.46468854	https://www.genecards.org/cgi-bin/carddisp.pl?gene=MYH9
OLR1	Oxidized Low Density Lipoprotein Receptor 1	Protein Coding	50	GC12M022629	3.458401203	https://www.genecards.org/cgi-bin/carddisp.pl?gene=OLR1
GP1BA	Glycoprotein Ib Platelet Subunit Alpha	Protein Coding	54	GC17P004932	3.456385374	https://www.genecards.org/cgi-bin/carddisp.pl?gene=GP1BA
GRIK1	Glutamate Ionotropic Receptor Kainate Type Subunit 1	Protein Coding	53	GC21M029536	3.454291821	https://www.genecards.org/cgi-bin/carddisp.pl?gene=GRIK1
ERBB4	Erb-B2 Receptor Tyrosine Kinase 4	Protein Coding	61	GC02M211375	3.447304487	https://www.genecards.org/cgi-bin/carddisp.pl?gene=ERBB4
NFKB2	Nuclear Factor Kappa B Subunit 2	Protein Coding	59	GC10P102394	3.43983674	https://www.genecards.org/cgi-bin/carddisp.pl?gene=NFKB2
ELOVL4	ELOVL Fatty Acid Elongase 4	Protein Coding	52	GC06M079914	3.439374208	https://www.genecards.org/cgi-bin/carddisp.pl?gene=ELOVL4
CD28	CD28 Molecule	Protein Coding	55	GC02P203706	3.433174372	https://www.genecards.org/cgi-bin/carddisp.pl?gene=CD28
THEM5	Thioesterase Superfamily Member 5	Protein Coding	41	GC01M152803	3.431970835	https://www.genecards.org/cgi-bin/carddisp.pl?gene=THEM5
PDZK1	PDZ Domain Containing 1	Protein Coding	47	GC01M145670	3.431917667	https://www.genecards.org/cgi-bin/carddisp.pl?gene=PDZK1

SLC51A	Solute Carrier Family 51 Subunit Alpha	Protein Coding	45	GC03P196211	3.431206226	https://www.genecards.org/cgi-bin/carddisp.pl?gene=SLC51A
ATF3	Activating Transcription Factor 3	Protein Coding	51	GC01P212565	3.43088913	https://www.genecards.org/cgi-bin/carddisp.pl?gene=ATF3
PKD1	Polycystin 1, Transient Receptor Potential Channel Interacting	Protein Coding	52	GC16M008093	3.430216074	https://www.genecards.org/cgi-bin/carddisp.pl?gene=PKD1
HARS1	Histidyl-TRNA Synthetase 1	Protein Coding	51	GC05M140673	3.425354958	https://www.genecards.org/cgi-bin/carddisp.pl?gene=HARS1
COL1A2	Collagen Type I Alpha 2 Chain	Protein Coding	54	GC07P094394	3.421122074	https://www.genecards.org/cgi-bin/carddisp.pl?gene=COL1A2
FGB	Fibrinogen Beta Chain	Protein Coding	53	GC04P154604	3.418199778	https://www.genecards.org/cgi-bin/carddisp.pl?gene=FGB
CEACAM5	CEA Cell Adhesion Molecule 5	Protein Coding	50	GC19P072938	3.411329746	https://www.genecards.org/cgi-bin/carddisp.pl?gene=CEACAM5
CDKN1B	Cyclin Dependent Kinase Inhibitor 1B	Protein Coding	54	GC12P023759	3.411257982	https://www.genecards.org/cgi-bin/carddisp.pl?gene=CDKN1B
PSMB8	Proteasome 20S Subunit Beta 8	Protein Coding	56	GC06M032840	3.408988237	https://www.genecards.org/cgi-bin/carddisp.pl?gene=PSMB8
DRD1	Dopamine Receptor D1	Protein Coding	51	GC05M175440	3.405468225	https://www.genecards.org/cgi-bin/carddisp.pl?gene=DRD1
IL11	Interleukin 11	Protein Coding	47	GC19M055364	3.404547215	https://www.genecards.org/cgi-bin/carddisp.pl?gene=IL11
SLC8A1	Solute Carrier Family 8 Member A1	Protein Coding	51	GC02M040078	3.403540134	https://www.genecards.org/cgi-bin/carddisp.pl?gene=SLC8A1
HDAC4	Histone Deacetylase 4	Protein Coding	59	GC02M239048	3.39973402	https://www.genecards.org/cgi-bin/carddisp.pl?gene=HDAC4
PTPN2	Protein Tyrosine Phosphatase Non-Receptor Type 2	Protein Coding	54	GC18M027584	3.393139839	https://www.genecards.org/cgi-bin/carddisp.pl?gene=PTPN2
MMP14	Matrix Metallopeptidase 14	Protein Coding	57	GC14P034667	3.391114712	https://www.genecards.org/cgi-bin/carddisp.pl?gene=MMP14

YARS1	Tyrosyl-TRNA Synthetase 1	Protein Coding	50	GC01M032776	3.390839338	https://www.genecards.org/cgi-bin/carddisp.pl?gene=YARS1
PRLR	Prolactin Receptor	Protein Coding	56	GC05M035048	3.390128136	https://www.genecards.org/cgi-bin/carddisp.pl?gene=PRLR
TUBB	Tubulin Beta Class I	Protein Coding	57	GC06P092134	3.388349056	https://www.genecards.org/cgi-bin/carddisp.pl?gene=TUBB
IRF1	Interferon Regulatory Factor 1	Protein Coding	53	GC05M132440	3.387298346	https://www.genecards.org/cgi-bin/carddisp.pl?gene=IRF1
PABPC1	Poly(A) Binding Protein Cytoplasmic 1	Protein Coding	49	GC08M100685	3.384418249	https://www.genecards.org/cgi-bin/carddisp.pl?gene=PABPC1
BCL2L1	BCL2 Like 1	Protein Coding	55	GC20M031664	3.38163662	https://www.genecards.org/cgi-bin/carddisp.pl?gene=BCL2L1
CYBB	Cytochrome B-245 Beta Chain	Protein Coding	56	GC0XP037780	3.380608082	https://www.genecards.org/cgi-bin/carddisp.pl?gene=CYBB
OXTR	Oxytocin Receptor	Protein Coding	53	GC03M008767	3.37797904	https://www.genecards.org/cgi-bin/carddisp.pl?gene=OXTR
CERS5	Ceramide Synthase 5	Protein Coding	46	GC12M050129	3.376144171	https://www.genecards.org/cgi-bin/carddisp.pl?gene=CERS5
AWAT2	Acyl-CoA Wax Alcohol Acyltransferase 2	Protein Coding	38	GC0XM070040	3.374199867	https://www.genecards.org/cgi-bin/carddisp.pl?gene=AWAT2
RAC2	Rac Family Small GTPase 2	Protein Coding	58	GC22M061462	3.370952129	https://www.genecards.org/cgi-bin/carddisp.pl?gene=RAC2
IGF2BP3	Insulin Like Growth Factor 2 MRNA Binding Protein 3	Protein Coding	48	GC07M023354	3.36882925	https://www.genecards.org/cgi-bin/carddisp.pl?gene=IGF2BP3
PBX1	PBX Homeobox 1	Protein Coding	55	GC01P164524	3.368083	https://www.genecards.org/cgi-bin/carddisp.pl?gene=PBX1
DLG1	Discs Large MAGUK Scaffold Protein 1	Protein Coding	50	GC03M197042	3.367190838	https://www.genecards.org/cgi-bin/carddisp.pl?gene=DLG1
HSP90B1	Heat Shock Protein 90 Beta Family Member 1	Protein Coding	53	GC12P103930	3.367063761	https://www.genecards.org/cgi-bin/carddisp.pl?gene=HSP90B1

SLC38A3	Solute Carrier Family 38 Member 3	Protein Coding	45	GC03P050205	3.364940166	https://www.genecards.org/cgi-bin/carddisp.pl?gene=SLC38A3
RHD	Rh Blood Group D Antigen	Protein Coding	48	GC01P025272	3.362401962	https://www.genecards.org/cgi-bin/carddisp.pl?gene=RHD
PLD2	Phospholipase D2	Protein Coding	53	GC17P004808	3.361949444	https://www.genecards.org/cgi-bin/carddisp.pl?gene=PLD2
PIM1	Pim-1 Proto-Oncogene, Serine/Threonine Kinase	Protein Coding	56	GC06P092302	3.3618958	https://www.genecards.org/cgi-bin/carddisp.pl?gene=PIM1
CDH13	Cadherin 13	Protein Coding	51	GC16P082626	3.359909773	https://www.genecards.org/cgi-bin/carddisp.pl?gene=CDH13
PTPRC	Protein Tyrosine Phosphatase Receptor Type C	Protein Coding	59	GC01P198607	3.355715752	https://www.genecards.org/cgi-bin/carddisp.pl?gene=PTPRC
ESD	Esterase D	Protein Coding	50	GC13M046771	3.354079723	https://www.genecards.org/cgi-bin/carddisp.pl?gene=ESD
GPER1	G Protein-Coupled Estrogen Receptor 1	Protein Coding	48	GC07P003391	3.351549625	https://www.genecards.org/cgi-bin/carddisp.pl?gene=GPER1
MAP2K2	Mitogen-Activated Protein Kinase Kinase 2	Protein Coding	61	GC19M004090	3.350414038	https://www.genecards.org/cgi-bin/carddisp.pl?gene=MAP2K2
GRIP1	Glutamate Receptor Interacting Protein 1	Protein Coding	50	GC12M066347	3.350006342	https://www.genecards.org/cgi-bin/carddisp.pl?gene=GRIP1
UCHL1	Ubiquitin C-Terminal Hydrolase L1	Protein Coding	59	GC04P041256	3.342181206	https://www.genecards.org/cgi-bin/carddisp.pl?gene=UCHL1
TP73	Tumor Protein P73	Protein Coding	52	GC01P003652	3.339853287	https://www.genecards.org/cgi-bin/carddisp.pl?gene=TP73
EEF1A1	Eukaryotic Translation Elongation Factor 1 Alpha 1	Protein Coding	50	GC06M074523	3.338254929	https://www.genecards.org/cgi-bin/carddisp.pl?gene=EEF1A1
ELOVL5	ELOVL Fatty Acid Elongase 5	Protein Coding	51	GC06M053267	3.337735653	https://www.genecards.org/cgi-bin/carddisp.pl?gene=ELOVL5
GRM7	Glutamate Metabotropic Receptor 7	Protein Coding	54	GC03P006770	3.334997654	https://www.genecards.org/cgi-bin/carddisp.pl?gene=GRM7

HBEGF	Heparin Binding EGF Like Growth Factor	Protein Coding	50	GC05M140332	3.331227303	https://www.genecards.org/cgi-bin/carddisp.pl?gene=HBEGF
SOS1	SOS Ras/Rac Guanine Nucleotide Exchange Factor 1	Protein Coding	56	GC02M039054	3.329431534	https://www.genecards.org/cgi-bin/carddisp.pl?gene=SOS1
BRS3	Bombesin Receptor Subtype 3	Protein Coding	48	GC0XP136500	3.328694344	https://www.genecards.org/cgi-bin/carddisp.pl?gene=BRS3
CD59	CD59 Molecule (CD59 Blood Group)	Protein Coding	53	GC11M033704	3.327222586	https://www.genecards.org/cgi-bin/carddisp.pl?gene=CD59
CASP9	Caspase 9	Protein Coding	53	GC01M015491	3.326052904	https://www.genecards.org/cgi-bin/carddisp.pl?gene=CASP9
KRT8	Keratin 8	Protein Coding	53	GC12M052897	3.321171284	https://www.genecards.org/cgi-bin/carddisp.pl?gene=KRT8
OGA	O-GlcNAcase	Protein Coding	47	GC10M101785	3.319125414	https://www.genecards.org/cgi-bin/carddisp.pl?gene=OGA
PDIA3	Protein Disulfide Isomerase Family A Member 3	Protein Coding	51	GC15P043746	3.316093206	https://www.genecards.org/cgi-bin/carddisp.pl?gene=PDIA3
SLC18A2	Solute Carrier Family 18 Member A2	Protein Coding	53	GC10P117241	3.314780474	https://www.genecards.org/cgi-bin/carddisp.pl?gene=SLC18A2
LIF	LIF Interleukin 6 Family Cytokine	Protein Coding	50	GC22M030240	3.313483238	https://www.genecards.org/cgi-bin/carddisp.pl?gene=LIF
ADAM10	ADAM Metallopeptidase Domain 10	Protein Coding	60	GC15M058588	3.312374353	https://www.genecards.org/cgi-bin/carddisp.pl?gene=ADAM10
HTR3A	5-Hydroxytryptamine Receptor 3A	Protein Coding	53	GC11P113975	3.311017513	https://www.genecards.org/cgi-bin/carddisp.pl?gene=HTR3A
PDE3A	Phosphodiesterase 3A	Protein Coding	56	GC12P023897	3.31002903	https://www.genecards.org/cgi-bin/carddisp.pl?gene=PDE3A
ACTN4	Actinin Alpha 4	Protein Coding	53	GC19P038647	3.30234313	https://www.genecards.org/cgi-bin/carddisp.pl?gene=ACTN4
PDGFRA	Platelet Derived Growth Factor Receptor Alpha	Protein Coding	60	GC04P054229	3.300298452	https://www.genecards.org/cgi-bin/carddisp.pl?gene=PDGFRA

CD5	CD5 Molecule	Protein Coding	49	GC11P061118	3.298510313	https://www.genecards.org/cgi-bin/carddisp.pl?gene=CD5
MKKS	MKKS Centrosomal Shuttling Protein	Protein Coding	46	GC20M010424	3.296190262	https://www.genecards.org/cgi-bin/carddisp.pl?gene=MKKS
MBP	Myelin Basic Protein	Protein Coding	51	GC18M076978	3.292306185	https://www.genecards.org/cgi-bin/carddisp.pl?gene=MBP
GATA1	GATA Binding Protein 1	Protein Coding	53	GC0XP048786	3.289469004	https://www.genecards.org/cgi-bin/carddisp.pl?gene=GATA1
PRLH	Prolactin Releasing Hormone	Protein Coding	37	GC02P237566	3.283510208	https://www.genecards.org/cgi-bin/carddisp.pl?gene=PRLH
FLT1	Fms Related Receptor Tyrosine Kinase 1	Protein Coding	57	GC13M028300	3.281445742	https://www.genecards.org/cgi-bin/carddisp.pl?gene=FLT1
NR4A1	Nuclear Receptor Subfamily 4 Group A Member 1	Protein Coding	54	GC12P052022	3.275763988	https://www.genecards.org/cgi-bin/carddisp.pl?gene=NR4A1
DUSP1	Dual Specificity Phosphatase 1	Protein Coding	52	GC05M172768	3.268606186	https://www.genecards.org/cgi-bin/carddisp.pl?gene=DUSP1
NMT1	N-Myristoyltransferase 1	Protein Coding	50	GC17P062083	3.262718201	https://www.genecards.org/cgi-bin/carddisp.pl?gene=NMT1
SMAD2	SMAD Family Member 2	Protein Coding	59	GC18M047809	3.257688046	https://www.genecards.org/cgi-bin/carddisp.pl?gene=SMAD2
F2RL1	F2R Like Trypsin Receptor 1	Protein Coding	51	GC05P076818	3.256936073	https://www.genecards.org/cgi-bin/carddisp.pl?gene=F2RL1
MT3	Metallothionein 3	Protein Coding	44	GC16P056589	3.255939245	https://www.genecards.org/cgi-bin/carddisp.pl?gene=MT3
AGPAT3	1-Acylglycerol-3-Phosphate O-Acyltransferase 3	Protein Coding	46	GC21P043865	3.250079155	https://www.genecards.org/cgi-bin/carddisp.pl?gene=AGPAT3
LRRK2	Leucine Rich Repeat Kinase 2	Protein Coding	56	GC12P040196	3.246121168	https://www.genecards.org/cgi-bin/carddisp.pl?gene=LRRK2
SGCB	Sarcoglycan Beta	Protein Coding	47	GC04M052019	3.243461609	https://www.genecards.org/cgi-bin/carddisp.pl?gene=SGCB

CRHR2	Corticotropin Releasing Hormone Receptor 2	Protein Coding	50	GC07M030651	3.241627216	https://www.genecards.org/cgi-bin/carddisp.pl?gene=CRHR2
PROS1	Protein S	Protein Coding	55	GC03M093873	3.237989426	https://www.genecards.org/cgi-bin/carddisp.pl?gene=PROS1
TRIM63	Tripartite Motif Containing 63	Protein Coding	48	GC01M026066	3.235760689	https://www.genecards.org/cgi-bin/carddisp.pl?gene=TRIM63
IL16	Interleukin 16	Protein Coding	48	GC15P081159	3.232122421	https://www.genecards.org/cgi-bin/carddisp.pl?gene=IL16
DRD3	Dopamine Receptor D3	Protein Coding	51	GC03M114128	3.227733612	https://www.genecards.org/cgi-bin/carddisp.pl?gene=DRD3
ALK	ALK Receptor Tyrosine Kinase	Protein Coding	59	GC02M029190	3.226660728	https://www.genecards.org/cgi-bin/carddisp.pl?gene=ALK
NF1	Neurofibromin 1	Protein Coding	54	GC17P031094	3.22521162	https://www.genecards.org/cgi-bin/carddisp.pl?gene=NF1
BUD23	BUD23 RRNA Methyltransferase And Ribosome Maturation Factor	Protein Coding	44	GC07P076357	3.224797249	https://www.genecards.org/cgi-bin/carddisp.pl?gene=BUD23
ATXN3	Ataxin 3	Protein Coding	52	GC14M103083	3.224014997	https://www.genecards.org/cgi-bin/carddisp.pl?gene=ATXN3
EIF4E	Eukaryotic Translation Initiation Factor 4E	Protein Coding	56	GC04M098879	3.223826408	https://www.genecards.org/cgi-bin/carddisp.pl?gene=EIF4E
JAK1	Janus Kinase 1	Protein Coding	60	GC01M064833	3.22107625	https://www.genecards.org/cgi-bin/carddisp.pl?gene=JAK1
SAT2	Spermidine/Spermine N1-Acetyltransferase Family Member 2	Protein Coding	43	GC17M007626	3.216407537	https://www.genecards.org/cgi-bin/carddisp.pl?gene=SAT2
CHRM3	Cholinergic Receptor Muscarinic 3	Protein Coding	55	GC01P239386	3.215517282	https://www.genecards.org/cgi-bin/carddisp.pl?gene=CHRM3
KRT14	Keratin 14	Protein Coding	53	GC17M041582	3.214873791	https://www.genecards.org/cgi-bin/carddisp.pl?gene=KRT14
LALBA	Lactalbumin Alpha	Protein Coding	45	GC12M048567	3.212843418	https://www.genecards.org/cgi-bin/carddisp.pl?gene=LALBA

TAC1	Tachykinin Precursor 1	Protein Coding	48	GC07P097731	3.212556362	https://www.genecards.org/cgi-bin/carddisp.pl?gene=TAC1
SMAD6	SMAD Family Member 6	Protein Coding	53	GC15P066702	3.210130453	https://www.genecards.org/cgi-bin/carddisp.pl?gene=SMAD6
CYSLTR2	Cysteinyl Leukotriene Receptor 2	Protein Coding	54	GC13P048653	3.206436157	https://www.genecards.org/cgi-bin/carddisp.pl?gene=CYSLTR2
MAG	Myelin Associated Glycoprotein	Protein Coding	54	GC19P035292	3.204558611	https://www.genecards.org/cgi-bin/carddisp.pl?gene=MAG
KHDRBS3	KH RNA Binding Domain Containing, Signal Transduction Associated 3	Protein Coding	45	GC08P135457	3.195662022	https://www.genecards.org/cgi-bin/carddisp.pl?gene=KHDRBS3
ACTN1	Actinin Alpha 1	Protein Coding	56	GC14M068874	3.193297863	https://www.genecards.org/cgi-bin/carddisp.pl?gene=ACTN1
TTF2	Transcription Termination Factor 2	Protein Coding	46	GC01P117060	3.191391945	https://www.genecards.org/cgi-bin/carddisp.pl?gene=TTF2
SLC22A8	Solute Carrier Family 22 Member 8	Protein Coding	49	GC11M099608	3.190941095	https://www.genecards.org/cgi-bin/carddisp.pl?gene=SLC22A8
UBE3A	Ubiquitin Protein Ligase E3A	Protein Coding	53	GC15M025333	3.18576026	https://www.genecards.org/cgi-bin/carddisp.pl?gene=UBE3A
CSF1R	Colony Stimulating Factor 1 Receptor	Protein Coding	59	GC05M150053	3.183142424	https://www.genecards.org/cgi-bin/carddisp.pl?gene=CSF1R
SLC1A2	Solute Carrier Family 1 Member 2	Protein Coding	56	GC11M035267	3.181488752	https://www.genecards.org/cgi-bin/carddisp.pl?gene=SLC1A2
ANG	Angiogenin	Protein Coding	51	GC14P034388	3.176154375	https://www.genecards.org/cgi-bin/carddisp.pl?gene=ANG
RPA2	Replication Protein A2	Protein Coding	50	GC01M028119	3.170302391	https://www.genecards.org/cgi-bin/carddisp.pl?gene=RPA2
SERPING1	Serpin Family G Member 1	Protein Coding	54	GC11P057597	3.169795513	https://www.genecards.org/cgi-bin/carddisp.pl?gene=SERPING1
PTGER3	Prostaglandin E Receptor 3	Protein Coding	53	GC01M070852	3.167470455	https://www.genecards.org/cgi-bin/carddisp.pl?gene=PTGER3

JAK3	Janus Kinase 3	Protein Coding	58	GC19M017824	3.165206909	https://www.genecards.org/cgi-bin/carddisp.pl?gene=JAK3
CDC42	Cell Division Cycle 42	Protein Coding	57	GC01P022052	3.15984416	https://www.genecards.org/cgi-bin/carddisp.pl?gene=CDC42
WNT5A	Wnt Family Member 5A	Protein Coding	56	GC03M055465	3.15939784	https://www.genecards.org/cgi-bin/carddisp.pl?gene=WNT5A
S100A9	S100 Calcium Binding Protein A9	Protein Coding	50	GC01P153357	3.159015179	https://www.genecards.org/cgi-bin/carddisp.pl?gene=S100A9
CYGB	Cytoglobin	Protein Coding	45	GC17M076527	3.154545784	https://www.genecards.org/cgi-bin/carddisp.pl?gene=CYGB
KMT2A	Lysine Methyltransferase 2A	Protein Coding	54	GC11P118436	3.151390553	https://www.genecards.org/cgi-bin/carddisp.pl?gene=KMT2A
PDGFRL	Platelet Derived Growth Factor Receptor Like	Protein Coding	50	GC08P017576	3.149273634	https://www.genecards.org/cgi-bin/carddisp.pl?gene=PDGFRL
GRK2	G Protein-Coupled Receptor Kinase 2	Protein Coding	53	GC11P067266	3.148638248	https://www.genecards.org/cgi-bin/carddisp.pl?gene=GRK2
KITLG	KIT Ligand	Protein Coding	53	GC12M088492	3.148565292	https://www.genecards.org/cgi-bin/carddisp.pl?gene=KITLG
CDH2	Cadherin 2	Protein Coding	59	GC18M028088	3.1409235	https://www.genecards.org/cgi-bin/carddisp.pl?gene=CDH2
CSNK2A2	Casein Kinase 2 Alpha 2	Protein Coding	53	GC16M058157	3.137202501	https://www.genecards.org/cgi-bin/carddisp.pl?gene=CSNK2A2
POMGNT2	Protein O-Linked Mannose N-Acetylglucosaminyltransferase 2 (Beta 1,4-)	Protein Coding	44	GC03M043121	3.133676291	https://www.genecards.org/cgi-bin/carddisp.pl?gene=POMGNT2
ANXA2	Annexin A2	Protein Coding	55	GC15M060347	3.130560637	https://www.genecards.org/cgi-bin/carddisp.pl?gene=ANXA2
DDR1	Discoidin Domain Receptor Tyrosine Kinase 1	Protein Coding	53	GC06P092137	3.126752853	https://www.genecards.org/cgi-bin/carddisp.pl?gene=DDR1
CDK6	Cyclin Dependent Kinase 6	Protein Coding	59	GC07M092604	3.125250101	https://www.genecards.org/cgi-bin/carddisp.pl?gene=CDK6

MLN	Motilin	Protein Coding	40	GC06M033794	3.12496376	https://www.genecards.org/cgi-bin/carddisp.pl?gene=MLN
GSN	Gelsolin	Protein Coding	56	GC09P121201	3.119424343	https://www.genecards.org/cgi-bin/carddisp.pl?gene=GSN
PTCH1	Patched 1	Protein Coding	57	GC09M095442	3.118463278	https://www.genecards.org/cgi-bin/carddisp.pl?gene=PTCH1
SCG5	Secretogranin V	Protein Coding	43	GC15P032641	3.115576029	https://www.genecards.org/cgi-bin/carddisp.pl?gene=SCG5
ITPR1	Inositol 1,4,5-Trisphosphate Receptor Type 1	Protein Coding	55	GC03P004486	3.114220381	https://www.genecards.org/cgi-bin/carddisp.pl?gene=ITPR1
ELOVL7	ELOVL Fatty Acid Elongase 7	Protein Coding	44	GC05M060751	3.107929945	https://www.genecards.org/cgi-bin/carddisp.pl?gene=ELOVL7
CPQ	Carboxypeptidase Q	Protein Coding	44	GC08P096645	3.10552454	https://www.genecards.org/cgi-bin/carddisp.pl?gene=CPQ
STC2	Stanniocalcin 2	Protein Coding	48	GC05M173314	3.101755142	https://www.genecards.org/cgi-bin/carddisp.pl?gene=STC2
SLC15A1	Solute Carrier Family 15 Member 1	Protein Coding	50	GC13M098683	3.098032951	https://www.genecards.org/cgi-bin/carddisp.pl?gene=SLC15A1
CA10	Carbonic Anhydrase 10	Protein Coding	46	GC17M051630	3.09686017	https://www.genecards.org/cgi-bin/carddisp.pl?gene=CA10
TPR	Translocated Promoter Region, Nuclear Basket Protein	Protein Coding	50	GC01M186323	3.096314192	https://www.genecards.org/cgi-bin/carddisp.pl?gene=TPR
CALB1	Calbindin 1	Protein Coding	49	GC08M090058	3.095009327	https://www.genecards.org/cgi-bin/carddisp.pl?gene=CALB1
REL	REL Proto-Oncogene, NF-KB Subunit	Protein Coding	53	GC02P060881	3.093712807	https://www.genecards.org/cgi-bin/carddisp.pl?gene=REL
DDX5	DEAD-Box Helicase 5	Protein Coding	52	GC17M064498	3.092330456	https://www.genecards.org/cgi-bin/carddisp.pl?gene=DDX5
PDCD1	Programmed Cell Death 1	Protein Coding	55	GC02M241849	3.091902733	https://www.genecards.org/cgi-bin/carddisp.pl?gene=PDCD1

MSR1	Macrophage Scavenger Receptor 1	Protein Coding	52	GC08M016107	3.090070248	https://www.genecards.org/cgi-bin/carddisp.pl?gene=MSR1
RAN	RAN, Member RAS Oncogene Family	Protein Coding	50	GC12P130871	3.086040258	https://www.genecards.org/cgi-bin/carddisp.pl?gene=RAN
CDC25A	Cell Division Cycle 25A	Protein Coding	54	GC03M048195	3.084305286	https://www.genecards.org/cgi-bin/carddisp.pl?gene=CDC25A
ZDHHC5	Zinc Finger DHHC-Type Palmitoyltransferase 5	Protein Coding	44	GC11P057670	3.082505703	https://www.genecards.org/cgi-bin/carddisp.pl?gene=ZDHHC5
HPR	Haptoglobin-Related Protein	Protein Coding	46	GC16P072373	3.08214283	https://www.genecards.org/cgi-bin/carddisp.pl?gene=HPR
ACOT7	Acyl-CoA Thioesterase 7	Protein Coding	47	GC01M007591	3.081958532	https://www.genecards.org/cgi-bin/carddisp.pl?gene=ACOT7
PAK1	P21 (RAC1) Activated Kinase 1	Protein Coding	57	GC11M100100	3.080955982	https://www.genecards.org/cgi-bin/carddisp.pl?gene=PAK1
HYAL2	Hyaluronidase 2	Protein Coding	51	GC03M050317	3.079611301	https://www.genecards.org/cgi-bin/carddisp.pl?gene=HYAL2
DUSP19	Dual Specificity Phosphatase 19	Protein Coding	44	GC02P183078	3.077805996	https://www.genecards.org/cgi-bin/carddisp.pl?gene=DUSP19
MAPK7	Mitogen-Activated Protein Kinase 7	Protein Coding	54	GC17P061131	3.077512503	https://www.genecards.org/cgi-bin/carddisp.pl?gene=MAPK7
BAD	BCL2 Associated Agonist Of Cell Death	Protein Coding	51	GC11M099650	3.075921774	https://www.genecards.org/cgi-bin/carddisp.pl?gene=BAD
ADCY5	Adenylate Cyclase 5	Protein Coding	55	GC03M123282	3.074261189	https://www.genecards.org/cgi-bin/carddisp.pl?gene=ADCY5
ADAMTSL1	ADAMTS Like 1	Protein Coding	49	GC09P017906	3.073168039	https://www.genecards.org/cgi-bin/carddisp.pl?gene=ADAMTSL1
CCR5	C-C Motif Chemokine Receptor 5	Protein Coding	53	GC03P047615	3.073025703	https://www.genecards.org/cgi-bin/carddisp.pl?gene=CCR5
GZMB	Granzyme B	Protein Coding	54	GC14M024630	3.072257996	https://www.genecards.org/cgi-bin/carddisp.pl?gene=GZMB

HRH1	Histamine Receptor H1	Protein Coding	52	GC03P013698	3.071516037	https://www.genecards.org/cgi-bin/carddisp.pl?gene=HRH1
CERS3	Ceramide Synthase 3	Protein Coding	47	GC15M122864	3.067425251	https://www.genecards.org/cgi-bin/carddisp.pl?gene=CERS3
NEFH	Neurofilament Heavy Chain	Protein Coding	52	GC22P029480	3.063281059	https://www.genecards.org/cgi-bin/carddisp.pl?gene=NEFH
RPS6	Ribosomal Protein S6	Protein Coding	50	GC09M019375	3.061184645	https://www.genecards.org/cgi-bin/carddisp.pl?gene=RPS6
POU1F1	POU Class 1 Homeobox 1	Protein Coding	48	GC03M087259	3.055360556	https://www.genecards.org/cgi-bin/carddisp.pl?gene=POU1F1
AANAT	Aralkylamine N-Acetyltransferase	Protein Coding	46	GC17P076453	3.053804159	https://www.genecards.org/cgi-bin/carddisp.pl?gene=AANAT
GNAQ	G Protein Subunit Alpha Q	Protein Coding	55	GC09M077716	3.051543951	https://www.genecards.org/cgi-bin/carddisp.pl?gene=GNAQ
LCLAT1	Lysocardiolipin Acyltransferase 1	Protein Coding	43	GC02P030447	3.03848815	https://www.genecards.org/cgi-bin/carddisp.pl?gene=LCLAT1
IL2RG	Interleukin 2 Receptor Subunit Gamma	Protein Coding	55	GC0XM071108	3.037034273	https://www.genecards.org/cgi-bin/carddisp.pl?gene=IL2RG
NOX1	NADPH Oxidase 1	Protein Coding	50	GC0XM100843	3.033960581	https://www.genecards.org/cgi-bin/carddisp.pl?gene=NOX1
LPCAT3	Lysophosphatidylcholine Acyltransferase 3	Protein Coding	44	GC12M006976	3.032484293	https://www.genecards.org/cgi-bin/carddisp.pl?gene=LPCAT3
DSPP	Dentin Sialophosphoprotein	Protein Coding	45	GC04P087608	3.03011179	https://www.genecards.org/cgi-bin/carddisp.pl?gene=DSPP
EDNRB	Endothelin Receptor Type B	Protein Coding	56	GC13M077895	3.028537989	https://www.genecards.org/cgi-bin/carddisp.pl?gene=EDNRB
CCN1	Cellular Communication Network Factor 1	Protein Coding	47	GC01P085581	3.02657032	https://www.genecards.org/cgi-bin/carddisp.pl?gene=CCN1
UTS2	Urotensin 2	Protein Coding	46	GC01M007843	3.025096655	https://www.genecards.org/cgi-bin/carddisp.pl?gene=UTS2

CCND2	Cyclin D2	Protein Coding	55	GC12P023493	3.024245024	https://www.genecards.org/cgi-bin/carddisp.pl?gene=CCND2
RAB5A	RAB5A, Member RAS Oncogene Family	Protein Coding	51	GC03P019948	3.02043581	https://www.genecards.org/cgi-bin/carddisp.pl?gene=RAB5A
MKI67	Marker Of Proliferation Ki-67	Protein Coding	51	GC10M128096	3.018312931	https://www.genecards.org/cgi-bin/carddisp.pl?gene=MKI67
EIF2AK1	Eukaryotic Translation Initiation Factor 2 Alpha Kinase 1	Protein Coding	51	GC07M006022	3.017688274	https://www.genecards.org/cgi-bin/carddisp.pl?gene=EIF2AK1
MAP3K7	Mitogen-Activated Protein Kinase Kinase Kinase 7	Protein Coding	57	GC06M090513	3.015327454	https://www.genecards.org/cgi-bin/carddisp.pl?gene=MAP3K7
CERS4	Ceramide Synthase 4	Protein Coding	48	GC19P008206	3.009953976	https://www.genecards.org/cgi-bin/carddisp.pl?gene=CERS4
F2R	Coagulation Factor II Thrombin Receptor	Protein Coding	53	GC05P076716	3.008503437	https://www.genecards.org/cgi-bin/carddisp.pl?gene=F2R
CR1	Complement C3b/C4b Receptor 1 (Knops Blood Group)	Protein Coding	51	GC01P207496	3.006123543	https://www.genecards.org/cgi-bin/carddisp.pl?gene=CR1
CCNA2	Cyclin A2	Protein Coding	52	GC04M121816	3.00469923	https://www.genecards.org/cgi-bin/carddisp.pl?gene=CCNA2
MSBP1	Minisatellite Binding Protein 1	Protein Coding	6	GC00U990213	3.003406048	https://www.genecards.org/cgi-bin/carddisp.pl?gene=MSBP1
EIF4B	Eukaryotic Translation Initiation Factor 4B	Protein Coding	48	GC12P053006	3.003115654	https://www.genecards.org/cgi-bin/carddisp.pl?gene=EIF4B
IYD	Iodotyrosine Deiodinase	Protein Coding	47	GC06P150368	3.001287222	https://www.genecards.org/cgi-bin/carddisp.pl?gene=IYD
NSF	N-Ethylmaleimide Sensitive Factor, Vesicle Fusing ATPase	Protein Coding	51	GC17P046590	2.995528221	https://www.genecards.org/cgi-bin/carddisp.pl?gene=NSF
LPCAT2	Lysophosphatidylcholine Acyltransferase 2	Protein Coding	48	GC16P055510	2.994910717	https://www.genecards.org/cgi-bin/carddisp.pl?gene=LPCAT2
MGAT5	Alpha-1,6-Mannosylglycoprotein 6-Beta-N-Acetylglucosaminyltransferase	Protein Coding	48	GC02P134119	2.994092226	https://www.genecards.org/cgi-bin/carddisp.pl?gene=MGAT5

TGFBI	Transforming Growth Factor Beta Induced	Protein Coding	53	GC05P136027	2.993627071	https://www.genecards.org/cgi-bin/carddisp.pl?gene=TGFBI
TGIF1	TGFB Induced Factor Homeobox 1	Protein Coding	53	GC18P003411	2.990906	https://www.genecards.org/cgi-bin/carddisp.pl?gene=TGIF1
ARHGAP1	Rho GTPase Activating Protein 1	Protein Coding	49	GC11M099353	2.990369797	https://www.genecards.org/cgi-bin/carddisp.pl?gene=ARHGAP1
FHL2	Four And A Half LIM Domains 2	Protein Coding	53	GC02M105357	2.988026142	https://www.genecards.org/cgi-bin/carddisp.pl?gene=FHL2
ELOVL3	ELOVL Fatty Acid Elongase 3	Protein Coding	41	GC10P102226	2.985116005	https://www.genecards.org/cgi-bin/carddisp.pl?gene=ELOVL3
GRM5	Glutamate Metabotropic Receptor 5	Protein Coding	53	GC11M088504	2.984042168	https://www.genecards.org/cgi-bin/carddisp.pl?gene=GRM5
HDAC6	Histone Deacetylase 6	Protein Coding	61	GC0XP048801	2.98350811	https://www.genecards.org/cgi-bin/carddisp.pl?gene=HDAC6
AQP9	Aquaporin 9	Protein Coding	49	GC15P058138	2.982341766	https://www.genecards.org/cgi-bin/carddisp.pl?gene=AQP9
CD274	CD274 Molecule	Protein Coding	52	GC09P005450	2.978649139	https://www.genecards.org/cgi-bin/carddisp.pl?gene=CD274
LNPEP	Leucyl And Cystinyl Aminopeptidase	Protein Coding	52	GC05P096935	2.976631403	https://www.genecards.org/cgi-bin/carddisp.pl?gene=LNPEP
L1CAM	L1 Cell Adhesion Molecule	Protein Coding	54	GC0XM153864	2.975522995	https://www.genecards.org/cgi-bin/carddisp.pl?gene=L1CAM
PDPK1	3-Phosphoinositide Dependent Protein Kinase 1	Protein Coding	55	GC16P002537	2.972537518	https://www.genecards.org/cgi-bin/carddisp.pl?gene=PDPK1
DES	Desmin	Protein Coding	55	GC02P219418	2.970502853	https://www.genecards.org/cgi-bin/carddisp.pl?gene=DES
PPBP	Pro-Platelet Basic Protein	Protein Coding	49	GC04M073986	2.964915276	https://www.genecards.org/cgi-bin/carddisp.pl?gene=PPBP
BLM	BLM RecQ Like Helicase	Protein Coding	56	GC15P090717	2.963648319	https://www.genecards.org/cgi-bin/carddisp.pl?gene=BLM

S100B	S100 Calcium Binding Protein B	Protein Coding	51	GC21M051379	2.961237431	https://www.genecards.org/cgi-bin/carddisp.pl?gene=S100B
RAB6A	RAB6A, Member RAS Oncogene Family	Protein Coding	47	GC11M099960	2.959060907	https://www.genecards.org/cgi-bin/carddisp.pl?gene=RAB6A
HELLS	Helicase, Lymphoid Specific	Protein Coding	52	GC10P094814	2.958610773	https://www.genecards.org/cgi-bin/carddisp.pl?gene=HELLS
PLAUR	Plasminogen Activator, Urokinase Receptor	Protein Coding	52	GC19M043646	2.953312874	https://www.genecards.org/cgi-bin/carddisp.pl?gene=PLAUR
ATF2	Activating Transcription Factor 2	Protein Coding	54	GC02M175072	2.950472832	https://www.genecards.org/cgi-bin/carddisp.pl?gene=ATF2
PTX3	Pentraxin 3	Protein Coding	50	GC03P157436	2.950279713	https://www.genecards.org/cgi-bin/carddisp.pl?gene=PTX3
CCND3	Cyclin D3	Protein Coding	53	GC06M041934	2.947537661	https://www.genecards.org/cgi-bin/carddisp.pl?gene=CCND3
ACSBG2	Acyl-CoA Synthetase Bubblegum Family Member 2	Protein Coding	44	GC19P006135	2.947389603	https://www.genecards.org/cgi-bin/carddisp.pl?gene=ACSBG2
ITGA5	Integrin Subunit Alpha 5	Protein Coding	56	GC12M055005	2.947187424	https://www.genecards.org/cgi-bin/carddisp.pl?gene=ITGA5
MBOAT2	Membrane Bound O-Acyltransferase Domain Containing 2	Protein Coding	42	GC02M008853	2.943485022	https://www.genecards.org/cgi-bin/carddisp.pl?gene=MBOAT2
PTPA	Protein Phosphatase 2 Phosphatase Activator	Protein Coding	50	GC09P129119	2.941158295	https://www.genecards.org/cgi-bin/carddisp.pl?gene=PTPA
THOP1	Thimet Oligopeptidase 1	Protein Coding	48	GC19P002785	2.939968348	https://www.genecards.org/cgi-bin/carddisp.pl?gene=THOP1
ITGA2	Integrin Subunit Alpha 2	Protein Coding	53	GC05P052989	2.937961817	https://www.genecards.org/cgi-bin/carddisp.pl?gene=ITGA2
BCL6	BCL6 Transcription Repressor	Protein Coding	52	GC03M187721	2.929877281	https://www.genecards.org/cgi-bin/carddisp.pl?gene=BCL6
NPY4R	Neuropeptide Y Receptor Y4	Protein Coding	46	GC10M046461	2.929151297	https://www.genecards.org/cgi-bin/carddisp.pl?gene=NPY4R

BAIAP2L1	BAR/IMD Domain Containing Adaptor Protein 2 Like 1	Protein Coding	46	GC07M098294	2.926037312	https://www.genecards.org/cgi-bin/carddisp.pl?gene=BAIAP2L1
MAPK9	Mitogen-Activated Protein Kinase 9	Protein Coding	56	GC05M180263	2.924700737	https://www.genecards.org/cgi-bin/carddisp.pl?gene=MAPK9
PRKD1	Protein Kinase D1	Protein Coding	56	GC14M029576	2.923382521	https://www.genecards.org/cgi-bin/carddisp.pl?gene=PRKD1
ADAM17	ADAM Metallopeptidase Domain 17	Protein Coding	58	GC02M009488	2.92128253	https://www.genecards.org/cgi-bin/carddisp.pl?gene=ADAM17
PRKCE	Protein Kinase C Epsilon	Protein Coding	55	GC02P045651	2.918762207	https://www.genecards.org/cgi-bin/carddisp.pl?gene=PRKCE
CBX5	Chromobox 5	Protein Coding	50	GC12M054230	2.918049335	https://www.genecards.org/cgi-bin/carddisp.pl?gene=CBX5
CRYAB	Crystallin Alpha B	Protein Coding	53	GC11M111908	2.917105913	https://www.genecards.org/cgi-bin/carddisp.pl?gene=CRYAB
WARS2	Tryptophanyl TRNA Synthetase 2, Mitochondrial	Protein Coding	51	GC01M119031	2.916296005	https://www.genecards.org/cgi-bin/carddisp.pl?gene=WARS2
SLC22A7	Solute Carrier Family 22 Member 7	Protein Coding	48	GC06P092349	2.914253712	https://www.genecards.org/cgi-bin/carddisp.pl?gene=SLC22A7
ADORA1	Adenosine A1 Receptor	Protein Coding	53	GC01P203090	2.91362977	https://www.genecards.org/cgi-bin/carddisp.pl?gene=ADORA1
VTN	Vitronectin	Protein Coding	51	GC17M051189	2.913039684	https://www.genecards.org/cgi-bin/carddisp.pl?gene=VTN
PTPN3	Protein Tyrosine Phosphatase Non-Receptor Type 3	Protein Coding	51	GC09M109375	2.905050516	https://www.genecards.org/cgi-bin/carddisp.pl?gene=PTPN3
SULT1A4	Sulfotransferase Family 1A Member 4	Protein Coding	37	GC16P042717	2.904377937	https://www.genecards.org/cgi-bin/carddisp.pl?gene=SULT1A4
TIMP3	TIMP Metallopeptidase Inhibitor 3	Protein Coding	50	GC22P044970	2.901615143	https://www.genecards.org/cgi-bin/carddisp.pl?gene=TIMP3
SP3	Sp3 Transcription Factor	Protein Coding	50	GC02M173882	2.90152359	https://www.genecards.org/cgi-bin/carddisp.pl?gene=SP3

PLK1	Polo Like Kinase 1	Protein Coding	58	GC16P024566	2.901216269	https://www.genecards.org/cgi-bin/carddisp.pl?gene=PLK1
CSNK1A1	Casein Kinase 1 Alpha 1	Protein Coding	55	GC05M149492	2.89967227	https://www.genecards.org/cgi-bin/carddisp.pl?gene=CSNK1A1
GRB14	Growth Factor Receptor Bound Protein 14	Protein Coding	50	GC02M164492	2.891060591	https://www.genecards.org/cgi-bin/carddisp.pl?gene=GRB14
CXCR2	C-X-C Motif Chemokine Receptor 2	Protein Coding	57	GC02P218125	2.887881041	https://www.genecards.org/cgi-bin/carddisp.pl?gene=CXCR2
ITK	IL2 Inducible T Cell Kinase	Protein Coding	59	GC05P157158	2.886702299	https://www.genecards.org/cgi-bin/carddisp.pl?gene=ITK
GNAO1	G Protein Subunit Alpha O1	Protein Coding	52	GC16P056231	2.884417772	https://www.genecards.org/cgi-bin/carddisp.pl?gene=GNAO1
AVPR2	Arginine Vasopressin Receptor 2	Protein Coding	53	GC0XP153902	2.882421732	https://www.genecards.org/cgi-bin/carddisp.pl?gene=AVPR2
SLC12A2	Solute Carrier Family 12 Member 2	Protein Coding	56	GC05P128083	2.881573439	https://www.genecards.org/cgi-bin/carddisp.pl?gene=SLC12A2
CXCR1	C-X-C Motif Chemokine Receptor 1	Protein Coding	50	GC02M218162	2.877828598	https://www.genecards.org/cgi-bin/carddisp.pl?gene=CXCR1
TRAF3	TNF Receptor Associated Factor 3	Protein Coding	53	GC14P110622	2.876880646	https://www.genecards.org/cgi-bin/carddisp.pl?gene=TRAF3
BIRC3	Baculoviral IAP Repeat Containing 3	Protein Coding	53	GC11P102317	2.874317646	https://www.genecards.org/cgi-bin/carddisp.pl?gene=BIRC3
EPHA4	EPH Receptor A4	Protein Coding	57	GC02M221418	2.871276379	https://www.genecards.org/cgi-bin/carddisp.pl?gene=EPHA4
MYLK	Myosin Light Chain Kinase	Protein Coding	57	GC03M123610	2.868624449	https://www.genecards.org/cgi-bin/carddisp.pl?gene=MYLK
AGPAT5	1-Acylglycerol-3-Phosphate O-Acyltransferase 5	Protein Coding	48	GC08P006708	2.867540121	https://www.genecards.org/cgi-bin/carddisp.pl?gene=AGPAT5
CDO1	Cysteine Dioxygenase Type 1	Protein Coding	47	GC05M115804	2.867174625	https://www.genecards.org/cgi-bin/carddisp.pl?gene=CDO1

FURIN	Furin, Paired Basic Amino Acid Cleaving Enzyme	Protein Coding	54	GC15P090868	2.866317272	https://www.genecards.org/cgi-bin/carddisp.pl?gene=FURIN
INHA	Inhibin Subunit Alpha	Protein Coding	49	GC02P219569	2.866305113	https://www.genecards.org/cgi-bin/carddisp.pl?gene=INHA
CFL1	Cofilin 1	Protein Coding	52	GC11M065823	2.865230083	https://www.genecards.org/cgi-bin/carddisp.pl?gene=CFL1
MCL1	MCL1 Apoptosis Regulator, BCL2 Family Member	Protein Coding	55	GC01M152731	2.86384058	https://www.genecards.org/cgi-bin/carddisp.pl?gene=MCL1
GLYAT	Glycine-N-Acyltransferase	Protein Coding	46	GC11M099446	2.863129139	https://www.genecards.org/cgi-bin/carddisp.pl?gene=GLYAT
HBG1	Hemoglobin Subunit Gamma 1	Protein Coding	47	GC11M006834	2.862811327	https://www.genecards.org/cgi-bin/carddisp.pl?gene=HBG1
A2M	Alpha-2-Macroglobulin	Protein Coding	52	GC12M009067	2.858912945	https://www.genecards.org/cgi-bin/carddisp.pl?gene=A2M
PTGER4	Prostaglandin E Receptor 4	Protein Coding	52	GC05P040679	2.858474731	https://www.genecards.org/cgi-bin/carddisp.pl?gene=PTGER4
PCNA	Proliferating Cell Nuclear Antigen	Protein Coding	57	GC20M005114	2.857391119	https://www.genecards.org/cgi-bin/carddisp.pl?gene=PCNA
ITGAL	Integrin Subunit Alpha L	Protein Coding	53	GC16P030472	2.857074738	https://www.genecards.org/cgi-bin/carddisp.pl?gene=ITGAL
FCGR3A	Fc Gamma Receptor IIIa	Protein Coding	53	GC01M161541	2.850360155	https://www.genecards.org/cgi-bin/carddisp.pl?gene=FCGR3A
IGFBP6	Insulin Like Growth Factor Binding Protein 6	Protein Coding	50	GC12P053097	2.844712496	https://www.genecards.org/cgi-bin/carddisp.pl?gene=IGFBP6
TXN2	Thioredoxin 2	Protein Coding	50	GC22M036467	2.843144894	https://www.genecards.org/cgi-bin/carddisp.pl?gene=TXN2
CBX1	Chromobox 1	Protein Coding	48	GC17M048070	2.839906454	https://www.genecards.org/cgi-bin/carddisp.pl?gene=CBX1
PIAS4	Protein Inhibitor Of Activated STAT 4	Protein Coding	48	GC19P004007	2.83911252	https://www.genecards.org/cgi-bin/carddisp.pl?gene=PIAS4

MMP13	Matrix Metallopeptidase 13	Protein Coding	57	GC11M102942	2.838996172	https://www.genecards.org/cgi-bin/carddisp.pl?gene=MMP13
MYCN	MYCN Proto-Oncogene, BHLH Transcription Factor	Protein Coding	53	GC02P015954	2.838101864	https://www.genecards.org/cgi-bin/carddisp.pl?gene=MYCN
NPY1R	Neuropeptide Y Receptor Y1	Protein Coding	53	GC04M163323	2.837838888	https://www.genecards.org/cgi-bin/carddisp.pl?gene=NPY1R
TNFRSF10A	TNF Receptor Superfamily Member 10a	Protein Coding	52	GC08M023190	2.837100029	https://www.genecards.org/cgi-bin/carddisp.pl?gene=TNFRSF10A
KRIT1	KRIT1 Ankyrin Repeat Containing	Protein Coding	48	GC07M092198	2.832033157	https://www.genecards.org/cgi-bin/carddisp.pl?gene=KRIT1
SULT1C4	Sulfotransferase Family 1C Member 4	Protein Coding	43	GC02P108377	2.830641747	https://www.genecards.org/cgi-bin/carddisp.pl?gene=SULT1C4
MEFV	MEFV Innate Immunity Regulator, Pyrin	Protein Coding	51	GC16M008228	2.823741913	https://www.genecards.org/cgi-bin/carddisp.pl?gene=MEFV
PREP	Prolyl Endopeptidase	Protein Coding	48	GC06M105277	2.822573662	https://www.genecards.org/cgi-bin/carddisp.pl?gene=PREP
NF2	NF2, Moesin-Ezrin-Radixin Like (MERLIN) Tumor Suppressor	Protein Coding	55	GC22P029603	2.821547747	https://www.genecards.org/cgi-bin/carddisp.pl?gene=NF2
S100A8	S100 Calcium Binding Protein A8	Protein Coding	48	GC01M153391	2.820464373	https://www.genecards.org/cgi-bin/carddisp.pl?gene=S100A8
ATR	ATR Serine/Threonine Kinase	Protein Coding	59	GC03M142449	2.820094347	https://www.genecards.org/cgi-bin/carddisp.pl?gene=ATR
CCL4	C-C Motif Chemokine Ligand 4	Protein Coding	47	GC17P036103	2.819907188	https://www.genecards.org/cgi-bin/carddisp.pl?gene=CCL4
SERPINB1	Serpin Family B Member 1	Protein Coding	47	GC06M002852	2.815357924	https://www.genecards.org/cgi-bin/carddisp.pl?gene=SERPINB1
LIMK1	LIM Domain Kinase 1	Protein Coding	56	GC07P074082	2.813730001	https://www.genecards.org/cgi-bin/carddisp.pl?gene=LIMK1
PSMC4	Proteasome 26S Subunit, ATPase 4	Protein Coding	47	GC19P072849	2.812140942	https://www.genecards.org/cgi-bin/carddisp.pl?gene=PSMC4

YWHAH	Tyrosine 3-Monooxygenase/Tryptophan 5-Monooxygenase Activation Protein Eta	Protein Coding	53	GC22P031944	2.810483932	https://www.genecards.org/cgi-bin/carddisp.pl?gene=YWHAH
PECAM1	Platelet And Endothelial Cell Adhesion Molecule 1	Protein Coding	47	GC17M064319	2.80936718	https://www.genecards.org/cgi-bin/carddisp.pl?gene=PECAM1
FADD	Fas Associated Via Death Domain	Protein Coding	52	GC11P070203	2.808387756	https://www.genecards.org/cgi-bin/carddisp.pl?gene=FADD
AKAP13	A-Kinase Anchoring Protein 13	Protein Coding	49	GC15P123897	2.804094791	https://www.genecards.org/cgi-bin/carddisp.pl?gene=AKAP13
GRM1	Glutamate Metabotropic Receptor 1	Protein Coding	57	GC06P145973	2.799233913	https://www.genecards.org/cgi-bin/carddisp.pl?gene=GRM1
FEN1	Flap Structure-Specific Endonuclease 1	Protein Coding	53	GC11P061793	2.798650742	https://www.genecards.org/cgi-bin/carddisp.pl?gene=FEN1
SLC1A7	Solute Carrier Family 1 Member 7	Protein Coding	50	GC01M053087	2.797800779	https://www.genecards.org/cgi-bin/carddisp.pl?gene=SLC1A7
MTMR2	Myotubularin Related Protein 2	Protein Coding	52	GC11M100315	2.797639847	https://www.genecards.org/cgi-bin/carddisp.pl?gene=MTMR2
ERBB3	Erb-B2 Receptor Tyrosine Kinase 3	Protein Coding	60	GC12P057479	2.795985222	https://www.genecards.org/cgi-bin/carddisp.pl?gene=ERBB3
CXCL12	C-X-C Motif Chemokine Ligand 12	Protein Coding	51	GC10M044370	2.794643164	https://www.genecards.org/cgi-bin/carddisp.pl?gene=CXCL12
IBSP	Integrin Binding Sialoprotein	Protein Coding	45	GC04P087799	2.793280363	https://www.genecards.org/cgi-bin/carddisp.pl?gene=IBSP
CIITA	Class II Major Histocompatibility Complex Transactivator	Protein Coding	51	GC16P013730	2.792242765	https://www.genecards.org/cgi-bin/carddisp.pl?gene=CIITA
ACP2	Acid Phosphatase 2, Lysosomal	Protein Coding	49	GC11M099364	2.791862249	https://www.genecards.org/cgi-bin/carddisp.pl?gene=ACP2
CKS1B	CDC28 Protein Kinase Regulatory Subunit 1B	Protein Coding	48	GC01P154974	2.788207769	https://www.genecards.org/cgi-bin/carddisp.pl?gene=CKS1B
P2RY12	Purinergic Receptor P2Y12	Protein Coding	55	GC03M151336	2.787358761	https://www.genecards.org/cgi-bin/carddisp.pl?gene=P2RY12

RPS6KA1	Ribosomal Protein S6 Kinase A1	Protein Coding	57	GC01P026540	2.782677889	https://www.genecards.org/cgi-bin/carddisp.pl?gene=RPS6KA1
SULT1C3	Sulfotransferase Family 1C Member 3	Protein Coding	41	GC02P108230	2.780189991	https://www.genecards.org/cgi-bin/carddisp.pl?gene=SULT1C3
MICB	MHC Class I Polypeptide-Related Sequence B	Protein Coding	47	GC06P092149	2.778273106	https://www.genecards.org/cgi-bin/carddisp.pl?gene=MICB
ST3GAL5	ST3 Beta-Galactoside Alpha-2,3-Sialyltransferase 5	Protein Coding	53	GC02M085839	2.777647018	https://www.genecards.org/cgi-bin/carddisp.pl?gene=ST3GAL5
PIEZO1	Piezo Type Mechanosensitive Ion Channel Component 1	Protein Coding	47	GC16M088715	2.772140026	https://www.genecards.org/cgi-bin/carddisp.pl?gene=PIEZO1
MCAT	Malonyl-CoA-Acyl Carrier Protein Transacylase	Protein Coding	48	GC22M043132	2.77162981	https://www.genecards.org/cgi-bin/carddisp.pl?gene=MCAT
HIBADH	3-Hydroxyisobutyrate Dehydrogenase	Protein Coding	47	GC07M027525	2.771614313	https://www.genecards.org/cgi-bin/carddisp.pl?gene=HIBADH
SRSF2	Serine And Arginine Rich Splicing Factor 2	Protein Coding	47	GC17M076734	2.771296501	https://www.genecards.org/cgi-bin/carddisp.pl?gene=SRSF2
ETS1	ETS Proto-Oncogene 1, Transcription Factor	Protein Coding	53	GC11M128458	2.770843744	https://www.genecards.org/cgi-bin/carddisp.pl?gene=ETS1
PF4	Platelet Factor 4	Protein Coding	47	GC04M073980	2.770424366	https://www.genecards.org/cgi-bin/carddisp.pl?gene=PF4
ALPI	Alkaline Phosphatase, Intestinal	Protein Coding	53	GC02P232456	2.770240307	https://www.genecards.org/cgi-bin/carddisp.pl?gene=ALPI
PLCB3	Phospholipase C Beta 3	Protein Coding	56	GC11P064251	2.770110607	https://www.genecards.org/cgi-bin/carddisp.pl?gene=PLCB3
YBX1	Y-Box Binding Protein 1	Protein Coding	46	GC01P042682	2.769355774	https://www.genecards.org/cgi-bin/carddisp.pl?gene=YBX1
PANK3	Pantothenate Kinase 3	Protein Coding	44	GC05M168549	2.766651154	https://www.genecards.org/cgi-bin/carddisp.pl?gene=PANK3
RPL13A	Ribosomal Protein L13a	Protein Coding	48	GC19P049487	2.766079187	https://www.genecards.org/cgi-bin/carddisp.pl?gene=RPL13A

SUCLG2	Succinate-CoA Ligase GDP-Forming Subunit Beta	Protein Coding	49	GC03M067358	2.763974905	https://www.genecards.org/cgi-bin/carddisp.pl?gene=SUCLG2
FST	Follistatin	Protein Coding	55	GC05P053480	2.76219511	https://www.genecards.org/cgi-bin/carddisp.pl?gene=FST
IKBKE	Inhibitor Of Nuclear Factor Kappa B Kinase Subunit Epsilon	Protein Coding	52	GC01P206470	2.76121664	https://www.genecards.org/cgi-bin/carddisp.pl?gene=IKBKE
AWAT1	Acyl-CoA Wax Alcohol Acyltransferase 1	Protein Coding	32	GC0XP070234	2.760582924	https://www.genecards.org/cgi-bin/carddisp.pl?gene=AWAT1
IL2RB	Interleukin 2 Receptor Subunit Beta	Protein Coding	56	GC22M061459	2.75886631	https://www.genecards.org/cgi-bin/carddisp.pl?gene=IL2RB
IRF3	Interferon Regulatory Factor 3	Protein Coding	51	GC19M049659	2.755576134	https://www.genecards.org/cgi-bin/carddisp.pl?gene=IRF3
EPHA2	EPH Receptor A2	Protein Coding	60	GC01M016124	2.755475283	https://www.genecards.org/cgi-bin/carddisp.pl?gene=EPHA2
ADRA2B	Adrenoceptor Alpha 2B	Protein Coding	51	GC02M096112	2.752860546	https://www.genecards.org/cgi-bin/carddisp.pl?gene=ADRA2B
PRLHR	Prolactin Releasing Hormone Receptor	Protein Coding	46	GC10M118590	2.752020121	https://www.genecards.org/cgi-bin/carddisp.pl?gene=PRLHR
FOSL1	FOS Like 1, AP-1 Transcription Factor Subunit	Protein Coding	51	GC11M099737	2.751973152	https://www.genecards.org/cgi-bin/carddisp.pl?gene=FOSL1
SENP2	SUMO Specific Peptidase 2	Protein Coding	47	GC03P185582	2.750661612	https://www.genecards.org/cgi-bin/carddisp.pl?gene=SENP2
TPM3	Tropomyosin 3	Protein Coding	53	GC01M154127	2.744642019	https://www.genecards.org/cgi-bin/carddisp.pl?gene=TPM3
LCK	LCK Proto-Oncogene, Src Family Tyrosine Kinase	Protein Coding	59	GC01P032251	2.742602825	https://www.genecards.org/cgi-bin/carddisp.pl?gene=LCK
FAR2	Fatty Acyl-CoA Reductase 2	Protein Coding	45	GC12P029145	2.741351843	https://www.genecards.org/cgi-bin/carddisp.pl?gene=FAR2
PTAFR	Platelet Activating Factor Receptor	Protein Coding	48	GC01M028147	2.740926266	https://www.genecards.org/cgi-bin/carddisp.pl?gene=PTAFR

POLB	DNA Polymerase Beta	Protein Coding	51	GC08P042338	2.739527225	https://www.genecards.org/cgi-bin/carddisp.pl?gene=POLB
IL4R	Interleukin 4 Receptor	Protein Coding	55	GC16P042490	2.739160299	https://www.genecards.org/cgi-bin/carddisp.pl?gene=IL4R
HSD17B6	Hydroxysteroid 17-Beta Dehydrogenase 6	Protein Coding	48	GC12P056752	2.739024639	https://www.genecards.org/cgi-bin/carddisp.pl?gene=HSD17B6
KHSRP	KH-Type Splicing Regulatory Protein	Protein Coding	48	GC19M006413	2.736800432	https://www.genecards.org/cgi-bin/carddisp.pl?gene=KHSRP
PTPRN2	Protein Tyrosine Phosphatase Receptor Type N2	Protein Coding	52	GC07M157539	2.735739231	https://www.genecards.org/cgi-bin/carddisp.pl?gene=PTPRN2
NCL	Nucleolin	Protein Coding	51	GC02M231453	2.735020161	https://www.genecards.org/cgi-bin/carddisp.pl?gene=NCL
CD47	CD47 Molecule	Protein Coding	51	GC03M108043	2.731025934	https://www.genecards.org/cgi-bin/carddisp.pl?gene=CD47
CTSL	Cathepsin L	Protein Coding	53	GC09P087725	2.730602264	https://www.genecards.org/cgi-bin/carddisp.pl?gene=CTSL
RACK1	Receptor For Activated C Kinase 1	Protein Coding	50	GC05M181933	2.726868153	https://www.genecards.org/cgi-bin/carddisp.pl?gene=RACK1
APLNR	Apelin Receptor	Protein Coding	51	GC11M057233	2.725875854	https://www.genecards.org/cgi-bin/carddisp.pl?gene=APLNR
PIAS1	Protein Inhibitor Of Activated STAT 1	Protein Coding	51	GC15P068054	2.72550869	https://www.genecards.org/cgi-bin/carddisp.pl?gene=PIAS1
ARF1	ADP Ribosylation Factor 1	Protein Coding	51	GC01P228082	2.724269629	https://www.genecards.org/cgi-bin/carddisp.pl?gene=ARF1
MOGAT1	Monoacylglycerol O-Acyltransferase 1	Protein Coding	41	GC02P222671	2.721960545	https://www.genecards.org/cgi-bin/carddisp.pl?gene=MOGAT1
BMP7	Bone Morphogenetic Protein 7	Protein Coding	52	GC20M057168	2.72125721	https://www.genecards.org/cgi-bin/carddisp.pl?gene=BMP7
CCNB1	Cyclin B1	Protein Coding	53	GC05P069167	2.720210075	https://www.genecards.org/cgi-bin/carddisp.pl?gene=CCNB1

TYK2	Tyrosine Kinase 2	Protein Coding	60	GC19M010350	2.719821692	https://www.genecards.org/cgi-bin/carddisp.pl?gene=TYK2
CFLAR	CASP8 And FADD Like Apoptosis Regulator	Protein Coding	51	GC02P201122	2.718475819	https://www.genecards.org/cgi-bin/carddisp.pl?gene=CFLAR
RECK	Reversion Inducing Cysteine Rich Protein With Kazal Motifs	Protein Coding	47	GC09P036036	2.718043327	https://www.genecards.org/cgi-bin/carddisp.pl?gene=RECK
MUC5AC	Mucin 5AC, Oligomeric Mucus/Gel-Forming	Protein Coding	46	GC11P002129	2.716766119	https://www.genecards.org/cgi-bin/carddisp.pl?gene=MUC5AC
CAMKK2	Calcium/Calmodulin Dependent Protein Kinase Kinase 2	Protein Coding	54	GC12M122474	2.714353085	https://www.genecards.org/cgi-bin/carddisp.pl?gene=CAMKK2
LMOD1	Leiomodin 1	Protein Coding	47	GC01M201896	2.712001324	https://www.genecards.org/cgi-bin/carddisp.pl?gene=LMOD1
CD81	CD81 Molecule	Protein Coding	53	GC11P002563	2.708006859	https://www.genecards.org/cgi-bin/carddisp.pl?gene=CD81
SYP	Synaptophysin	Protein Coding	52	GC0XM049187	2.701478481	https://www.genecards.org/cgi-bin/carddisp.pl?gene=SYP
FCGR2A	Fc Gamma Receptor IIa	Protein Coding	55	GC01P161505	2.700136185	https://www.genecards.org/cgi-bin/carddisp.pl?gene=FCGR2A
FMOD	Fibromodulin	Protein Coding	48	GC01M203340	2.698026896	https://www.genecards.org/cgi-bin/carddisp.pl?gene=FMOD
MBOAT1	Membrane Bound O-Acyltransferase Domain Containing 1	Protein Coding	42	GC06M020102	2.697533131	https://www.genecards.org/cgi-bin/carddisp.pl?gene=MBOAT1
PAK2	P21 (RAC1) Activated Kinase 2	Protein Coding	55	GC03P196739	2.692825556	https://www.genecards.org/cgi-bin/carddisp.pl?gene=PAK2
KRT19	Keratin 19	Protein Coding	50	GC17M041523	2.688460112	https://www.genecards.org/cgi-bin/carddisp.pl?gene=KRT19
SH2B3	SH2B Adaptor Protein 3	Protein Coding	54	GC12P111405	2.684833288	https://www.genecards.org/cgi-bin/carddisp.pl?gene=SH2B3
PCBP1	Poly(RC) Binding Protein 1	Protein Coding	48	GC02P070087	2.683332443	https://www.genecards.org/cgi-bin/carddisp.pl?gene=PCBP1

LEF1	Lymphoid Enhancer Binding Factor 1	Protein Coding	55	GC04M108047	2.683016062	https://www.genecards.org/cgi-bin/carddisp.pl?gene=LEF1
CDX2	Caudal Type Homeobox 2	Protein Coding	48	GC13M027962	2.681468248	https://www.genecards.org/cgi-bin/carddisp.pl?gene=CDX2
ACTC1	Actin Alpha Cardiac Muscle 1	Protein Coding	51	GC15M034790	2.680232525	https://www.genecards.org/cgi-bin/carddisp.pl?gene=ACTC1
LPAR1	Lysophosphatidic Acid Receptor 1	Protein Coding	53	GC09M110873	2.678920269	https://www.genecards.org/cgi-bin/carddisp.pl?gene=LPAR1
KRT7	Keratin 7	Protein Coding	48	GC12P052232	2.6780653	https://www.genecards.org/cgi-bin/carddisp.pl?gene=KRT7
HAS3	Hyaluronan Synthase 3	Protein Coding	48	GC16P069105	2.677442789	https://www.genecards.org/cgi-bin/carddisp.pl?gene=HAS3
FYN	FYN Proto-Oncogene, Src Family Tyrosine Kinase	Protein Coding	53	GC06M111660	2.676315069	https://www.genecards.org/cgi-bin/carddisp.pl?gene=FYN
MAP2K7	Mitogen-Activated Protein Kinase Kinase 7	Protein Coding	52	GC19P007903	2.675574064	https://www.genecards.org/cgi-bin/carddisp.pl?gene=MAP2K7
HSPA9	Heat Shock Protein Family A (Hsp70) Member 9	Protein Coding	54	GC05M138554	2.675514698	https://www.genecards.org/cgi-bin/carddisp.pl?gene=HSPA9
TFG	Trafficking From ER To Golgi Regulator	Protein Coding	51	GC03P100709	2.672077417	https://www.genecards.org/cgi-bin/carddisp.pl?gene=TFG
COL3A1	Collagen Type III Alpha 1 Chain	Protein Coding	54	GC02P188974	2.669884205	https://www.genecards.org/cgi-bin/carddisp.pl?gene=COL3A1
MST1	Macrophage Stimulating 1	Protein Coding	53	GC03M049683	2.668335438	https://www.genecards.org/cgi-bin/carddisp.pl?gene=MST1
TBP	TATA-Box Binding Protein	Protein Coding	55	GC06P170554	2.667933941	https://www.genecards.org/cgi-bin/carddisp.pl?gene=TBP
RYR3	Ryanodine Receptor 3	Protein Coding	48	GC15P033310	2.665052652	https://www.genecards.org/cgi-bin/carddisp.pl?gene=RYR3
GJA1	Gap Junction Protein Alpha 1	Protein Coding	57	GC06P121436	2.663125038	https://www.genecards.org/cgi-bin/carddisp.pl?gene=GJA1

ANK1	Ankyrin 1	Protein Coding	50	GC08M041653	2.662607908	https://www.genecards.org/cgi-bin/carddisp.pl?gene=ANK1
SYT1	Synaptotagmin 1	Protein Coding	55	GC12P078863	2.662100077	https://www.genecards.org/cgi-bin/carddisp.pl?gene=SYT1
BLK	BLK Proto-Oncogene, Src Family Tyrosine Kinase	Protein Coding	56	GC08P011486	2.66164875	https://www.genecards.org/cgi-bin/carddisp.pl?gene=BLK
SMAD7	SMAD Family Member 7	Protein Coding	51	GC18M048919	2.660331011	https://www.genecards.org/cgi-bin/carddisp.pl?gene=SMAD7
LBP	Lipopolysaccharide Binding Protein	Protein Coding	51	GC20P038346	2.656612635	https://www.genecards.org/cgi-bin/carddisp.pl?gene=LBP
ZAP70	Zeta Chain Of T Cell Receptor Associated Protein Kinase 70	Protein Coding	59	GC02P097773	2.655203342	https://www.genecards.org/cgi-bin/carddisp.pl?gene=ZAP70
JUP	Junction Plakoglobin	Protein Coding	55	GC17M041754	2.653947115	https://www.genecards.org/cgi-bin/carddisp.pl?gene=JUP
UBE2K	Ubiquitin Conjugating Enzyme E2 K	Protein Coding	48	GC04P039700	2.653835773	https://www.genecards.org/cgi-bin/carddisp.pl?gene=UBE2K
FMR1	Fragile X Messenger Ribonucleoprotein 1	Protein Coding	51	GC0XP147956	2.65137434	https://www.genecards.org/cgi-bin/carddisp.pl?gene=FMR1
MAP2K4	Mitogen-Activated Protein Kinase Kinase 4	Protein Coding	53	GC17P012020	2.649491787	https://www.genecards.org/cgi-bin/carddisp.pl?gene=MAP2K4
GPR42	G Protein-Coupled Receptor 42	Protein Coding	30	GC19P073881	2.649134159	https://www.genecards.org/cgi-bin/carddisp.pl?gene=GPR42
ST6GAL1	ST6 Beta-Galactoside Alpha-2,6-Sialyltransferase 1	Protein Coding	51	GC03P186930	2.644136429	https://www.genecards.org/cgi-bin/carddisp.pl?gene=ST6GAL1
NPHS1	NPHS1 Adhesion Molecule, Nephrin	Protein Coding	53	GC19M035825	2.644048214	https://www.genecards.org/cgi-bin/carddisp.pl?gene=NPHS1
XPO1	Exportin 1	Protein Coding	55	GC02M061445	2.641967058	https://www.genecards.org/cgi-bin/carddisp.pl?gene=XPO1
TFF3	Trefoil Factor 3	Protein Coding	47	GC21M042311	2.641746759	https://www.genecards.org/cgi-bin/carddisp.pl?gene=TFF3

TMPO	Thymopoietin	Protein Coding	53	GC12P098515	2.639974356	https://www.genecards.org/cgi-bin/carddisp.pl?gene=TMPO
GNAI1	G Protein Subunit Alpha I1	Protein Coding	53	GC07P079769	2.639129162	https://www.genecards.org/cgi-bin/carddisp.pl?gene=GNAI1
REST	RE1 Silencing Transcription Factor	Protein Coding	49	GC04P056907	2.639044046	https://www.genecards.org/cgi-bin/carddisp.pl?gene=REST
PPP1R1B	Protein Phosphatase 1 Regulatory Inhibitor Subunit 1B	Protein Coding	49	GC17P039626	2.632069588	https://www.genecards.org/cgi-bin/carddisp.pl?gene=PPP1R1B
IL12RB1	Interleukin 12 Receptor Subunit Beta 1	Protein Coding	52	GC19M018058	2.624730587	https://www.genecards.org/cgi-bin/carddisp.pl?gene=IL12RB1
GHRHR	Growth Hormone Releasing Hormone Receptor	Protein Coding	49	GC07P030938	2.624176502	https://www.genecards.org/cgi-bin/carddisp.pl?gene=GHRHR
EPHB4	EPH Receptor B4	Protein Coding	60	GC07M102433	2.621126175	https://www.genecards.org/cgi-bin/carddisp.pl?gene=EPHB4
ADRA2C	Adrenoceptor Alpha 2C	Protein Coding	52	GC04P003766	2.617765427	https://www.genecards.org/cgi-bin/carddisp.pl?gene=ADRA2C
GLYATL1	Glycine-N-Acyltransferase Like 1	Protein Coding	40	GC11P058906	2.615096807	https://www.genecards.org/cgi-bin/carddisp.pl?gene=GLYATL1
MICA	MHC Class I Polypeptide-Related Sequence A	Protein Coding	46	GC06P031399	2.612233639	https://www.genecards.org/cgi-bin/carddisp.pl?gene=MICA
TP53BP1	Tumor Protein P53 Binding Protein 1	Protein Coding	50	GC15M043403	2.604650736	https://www.genecards.org/cgi-bin/carddisp.pl?gene=TP53BP1
AGPAT4	1-Acylglycerol-3-Phosphate O-Acyltransferase 4	Protein Coding	47	GC06M161129	2.601899624	https://www.genecards.org/cgi-bin/carddisp.pl?gene=AGPAT4
ROCK1	Rho Associated Coiled-Coil Containing Protein Kinase 1	Protein Coding	56	GC18M020946	2.60150075	https://www.genecards.org/cgi-bin/carddisp.pl?gene=ROCK1
NRP1	Neuropilin 1	Protein Coding	54	GC10M033177	2.599191189	https://www.genecards.org/cgi-bin/carddisp.pl?gene=NRP1
MAPKAPK2	MAPK Activated Protein Kinase 2	Protein Coding	54	GC01P206684	2.598197699	https://www.genecards.org/cgi-bin/carddisp.pl?gene=MAPKAPK2

CCKBR	Cholecystokinin B Receptor	Protein Coding	51	GC11P006259	2.589323282	https://www.genecards.org/cgi-bin/carddisp.pl?gene=CCKBR
TXK	TXK Tyrosine Kinase	Protein Coding	51	GC04M048066	2.589127541	https://www.genecards.org/cgi-bin/carddisp.pl?gene=TXK
THEM4	Thioesterase Superfamily Member 4	Protein Coding	47	GC01M151870	2.588637114	https://www.genecards.org/cgi-bin/carddisp.pl?gene=THEM4
TLR9	Toll Like Receptor 9	Protein Coding	53	GC03M052222	2.587840796	https://www.genecards.org/cgi-bin/carddisp.pl?gene=TLR9
PRKCQ	Protein Kinase C Theta	Protein Coding	55	GC10M006591	2.587505102	https://www.genecards.org/cgi-bin/carddisp.pl?gene=PRKCQ
PTPRN	Protein Tyrosine Phosphatase Receptor Type N	Protein Coding	51	GC02M219289	2.586440563	https://www.genecards.org/cgi-bin/carddisp.pl?gene=PTPRN
LHCGR	Luteinizing Hormone/Choriogonadotropin Receptor	Protein Coding	53	GC02M048686	2.585967064	https://www.genecards.org/cgi-bin/carddisp.pl?gene=LHCGR
OSM	Oncostatin M	Protein Coding	50	GC22M030262	2.585475683	https://www.genecards.org/cgi-bin/carddisp.pl?gene=OSM
PITPNM1	Phosphatidylinositol Transfer Protein Membrane Associated 1	Protein Coding	46	GC11M099817	2.578641653	https://www.genecards.org/cgi-bin/carddisp.pl?gene=PITPNM1
BBOX1	Gamma-Butyrobetaine Hydroxylase 1	Protein Coding	46	GC11P027019	2.571772099	https://www.genecards.org/cgi-bin/carddisp.pl?gene=BBOX1
USP8	Ubiquitin Specific Peptidase 8	Protein Coding	53	GC15P050424	2.568456888	https://www.genecards.org/cgi-bin/carddisp.pl?gene=USP8
MYB	MYB Proto-Oncogene, Transcription Factor	Protein Coding	55	GC06P135180	2.568442822	https://www.genecards.org/cgi-bin/carddisp.pl?gene=MYB
PRF1	Perforin 1	Protein Coding	53	GC10M070597	2.566235065	https://www.genecards.org/cgi-bin/carddisp.pl?gene=PRF1
KIF5B	Kinesin Family Member 5B	Protein Coding	51	GC10M033629	2.563094139	https://www.genecards.org/cgi-bin/carddisp.pl?gene=KIF5B
SLC9A2	Solute Carrier Family 9 Member A2	Protein Coding	48	GC02P102620	2.559026003	https://www.genecards.org/cgi-bin/carddisp.pl?gene=SLC9A2

CXCR3	C-X-C Motif Chemokine Receptor 3	Protein Coding	51	GC0XM071615	2.55614996	https://www.genecards.org/cgi-bin/carddisp.pl?gene=CXCR3
RPL15	Ribosomal Protein L15	Protein Coding	50	GC03P023916	2.555617809	https://www.genecards.org/cgi-bin/carddisp.pl?gene=RPL15
DDX20	DEAD-Box Helicase 20	Protein Coding	49	GC01P111755	2.543823242	https://www.genecards.org/cgi-bin/carddisp.pl?gene=DDX20
HSF1	Heat Shock Transcription Factor 1	Protein Coding	53	GC08P144291	2.543515205	https://www.genecards.org/cgi-bin/carddisp.pl?gene=HSF1
TRAPPC10	Trafficking Protein Particle Complex Subunit 10	Protein Coding	45	GC21P044012	2.542541742	https://www.genecards.org/cgi-bin/carddisp.pl?gene=TRAPPC10
TYRP1	Tyrosinase Related Protein 1	Protein Coding	54	GC09P012683	2.541297197	https://www.genecards.org/cgi-bin/carddisp.pl?gene=TYRP1
LGMN	Legumain	Protein Coding	49	GC14M092703	2.540930271	https://www.genecards.org/cgi-bin/carddisp.pl?gene=LGMN
IVL	Involucrin	Protein Coding	45	GC01P153037	2.537791967	https://www.genecards.org/cgi-bin/carddisp.pl?gene=IVL
NEB	Nebulin	Protein Coding	47	GC02M151485	2.537733078	https://www.genecards.org/cgi-bin/carddisp.pl?gene=NEB
SAFB	Scaffold Attachment Factor B	Protein Coding	46	GC19P005623	2.537121773	https://www.genecards.org/cgi-bin/carddisp.pl?gene=SAFB
FGF7	Fibroblast Growth Factor 7	Protein Coding	50	GC15P049423	2.536988258	https://www.genecards.org/cgi-bin/carddisp.pl?gene=FGF7
SLC7A1	Solute Carrier Family 7 Member 1	Protein Coding	49	GC13M029509	2.536151409	https://www.genecards.org/cgi-bin/carddisp.pl?gene=SLC7A1
NR2F2	Nuclear Receptor Subfamily 2 Group F Member 2	Protein Coding	56	GC15P096325	2.534425974	https://www.genecards.org/cgi-bin/carddisp.pl?gene=NR2F2
TFPI	Tissue Factor Pathway Inhibitor	Protein Coding	53	GC02M187464	2.532000542	https://www.genecards.org/cgi-bin/carddisp.pl?gene=TFPI
AREG	Amphiregulin	Protein Coding	50	GC04P074445	2.528548479	https://www.genecards.org/cgi-bin/carddisp.pl?gene=AREG

CTSC	Cathepsin C	Protein Coding	54	GC11M100229	2.527719021	https://www.genecards.org/cgi-bin/carddisp.pl?gene=CTSC
CCR3	C-C Motif Chemokine Receptor 3	Protein Coding	52	GC03P047610	2.519971371	https://www.genecards.org/cgi-bin/carddisp.pl?gene=CCR3
ECE1	Endothelin Converting Enzyme 1	Protein Coding	55	GC01M021217	2.518996477	https://www.genecards.org/cgi-bin/carddisp.pl?gene=ECE1
CRKL	CRK Like Proto-Oncogene, Adaptor Protein	Protein Coding	53	GC22P020917	2.518113375	https://www.genecards.org/cgi-bin/carddisp.pl?gene=CRKL
CRHR1	Corticotropin Releasing Hormone Receptor 1	Protein Coding	51	GC17P045784	2.517398357	https://www.genecards.org/cgi-bin/carddisp.pl?gene=CRHR1
AASDHPPT	Aminoadipate-Semialdehyde Dehydrogenase-Phosphopantetheinyl Transferase	Protein Coding	44	GC11P106075	2.514085293	https://www.genecards.org/cgi-bin/carddisp.pl?gene=AASDHPPT
FCGR3B	Fc Gamma Receptor IIIb	Protein Coding	50	GC01M161623	2.514079332	https://www.genecards.org/cgi-bin/carddisp.pl?gene=FCGR3B
ELOC	Elongin C	Protein Coding	48	GC08M073939	2.512938499	https://www.genecards.org/cgi-bin/carddisp.pl?gene=ELOC
CA3	Carbonic Anhydrase 3	Protein Coding	48	GC08P085373	2.505985737	https://www.genecards.org/cgi-bin/carddisp.pl?gene=CA3
DDX1	DEAD-Box Helicase 1	Protein Coding	50	GC02P015591	2.50277853	https://www.genecards.org/cgi-bin/carddisp.pl?gene=DDX1
SOCS2	Suppressor Of Cytokine Signaling 2	Protein Coding	50	GC12P093569	2.50228858	https://www.genecards.org/cgi-bin/carddisp.pl?gene=SOCS2
BAZ2B	Bromodomain Adjacent To Zinc Finger Domain 2B	Protein Coding	47	GC02M159318	2.50156641	https://www.genecards.org/cgi-bin/carddisp.pl?gene=BAZ2B
KISS1R	KISS1 Receptor	Protein Coding	53	GC19P000917	2.501128435	https://www.genecards.org/cgi-bin/carddisp.pl?gene=KISS1R
ENPP7	Ectonucleotide Pyrophosphatase/Phosphodiesterase 7	Protein Coding	44	GC17P079730	2.501049042	https://www.genecards.org/cgi-bin/carddisp.pl?gene=ENPP7
RPS6KA3	Ribosomal Protein S6 Kinase A3	Protein Coding	59	GC0XM020149	2.500823259	https://www.genecards.org/cgi-bin/carddisp.pl?gene=RPS6KA3

TEK	TEK Receptor Tyrosine Kinase	Protein Coding	58	GC09P027109	2.500086546	https://www.genecards.org/cgi-bin/carddisp.pl?gene=TEK
CSK	C-Terminal Src Kinase	Protein Coding	53	GC15P074782	2.496485472	https://www.genecards.org/cgi-bin/carddisp.pl?gene=CSK
MPL	MPL Proto-Oncogene, Thrombopoietin Receptor	Protein Coding	55	GC01P043337	2.496141434	https://www.genecards.org/cgi-bin/carddisp.pl?gene=MPL
PAPPA	Pappalysin 1	Protein Coding	50	GC09P119606	2.495812654	https://www.genecards.org/cgi-bin/carddisp.pl?gene=PAPPA
ARRB2	Arrestin Beta 2	Protein Coding	51	GC17P004711	2.491093874	https://www.genecards.org/cgi-bin/carddisp.pl?gene=ARRB2
SPI1	Spi-1 Proto-Oncogene	Protein Coding	50	GC11M099368	2.48957324	https://www.genecards.org/cgi-bin/carddisp.pl?gene=SPI1
EPOR	Erythropoietin Receptor	Protein Coding	56	GC19M011377	2.486668587	https://www.genecards.org/cgi-bin/carddisp.pl?gene=EPOR
PRKCG	Protein Kinase C Gamma	Protein Coding	58	GC19P053879	2.486482143	https://www.genecards.org/cgi-bin/carddisp.pl?gene=PRKCG
ROS1	ROS Proto-Oncogene 1, Receptor Tyrosine Kinase	Protein Coding	53	GC06M117287	2.483052492	https://www.genecards.org/cgi-bin/carddisp.pl?gene=ROS1
SULT1C2	Sulfotransferase Family 1C Member 2	Protein Coding	48	GC02P108288	2.480620861	https://www.genecards.org/cgi-bin/carddisp.pl?gene=SULT1C2
IL33	Interleukin 33	Protein Coding	46	GC09P007685	2.480547905	https://www.genecards.org/cgi-bin/carddisp.pl?gene=IL33
C5AR1	Complement C5a Receptor 1	Protein Coding	51	GC19P047290	2.479447842	https://www.genecards.org/cgi-bin/carddisp.pl?gene=C5AR1
TMPRSS15	Transmembrane Serine Protease 15	Protein Coding	51	GC21M018269	2.477105141	https://www.genecards.org/cgi-bin/carddisp.pl?gene=TMPRSS15
CHI3L1	Chitinase 3 Like 1	Protein Coding	51	GC01M203148	2.477016211	https://www.genecards.org/cgi-bin/carddisp.pl?gene=CHI3L1
DAXX	Death Domain Associated Protein	Protein Coding	50	GC06M033318	2.475946665	https://www.genecards.org/cgi-bin/carddisp.pl?gene=DAXX

MTF1	Metal Regulatory Transcription Factor 1	Protein Coding	47	GC01M037810	2.473935127	https://www.genecards.org/cgi-bin/carddisp.pl?gene=MTF1
ITIH4	Inter-Alpha-Trypsin Inhibitor Heavy Chain 4	Protein Coding	50	GC03M052812	2.473416805	https://www.genecards.org/cgi-bin/carddisp.pl?gene=ITIH4
GIPC1	GIPC PDZ Domain Containing Family Member 1	Protein Coding	51	GC19M014689	2.472416639	https://www.genecards.org/cgi-bin/carddisp.pl?gene=GIPC1
ILK	Integrin Linked Kinase	Protein Coding	52	GC11P006604	2.47030139	https://www.genecards.org/cgi-bin/carddisp.pl?gene=ILK
KRT16	Keratin 16	Protein Coding	50	GC17M041609	2.468029022	https://www.genecards.org/cgi-bin/carddisp.pl?gene=KRT16
NPR1	Natriuretic Peptide Receptor 1	Protein Coding	53	GC01P153922	2.459864855	https://www.genecards.org/cgi-bin/carddisp.pl?gene=NPR1
CRK	CRK Proto-Oncogene, Adaptor Protein	Protein Coding	50	GC17M001420	2.459316969	https://www.genecards.org/cgi-bin/carddisp.pl?gene=CRK
VAMP2	Vesicle Associated Membrane Protein 2	Protein Coding	50	GC17M011050	2.459011793	https://www.genecards.org/cgi-bin/carddisp.pl?gene=VAMP2
PDYN	Prodynorphin	Protein Coding	51	GC20M001978	2.456013918	https://www.genecards.org/cgi-bin/carddisp.pl?gene=PDYN
PTGER1	Prostaglandin E Receptor 1	Protein Coding	47	GC19M014687	2.453777313	https://www.genecards.org/cgi-bin/carddisp.pl?gene=PTGER1
PAX2	Paired Box 2	Protein Coding	53	GC10P100735	2.451199532	https://www.genecards.org/cgi-bin/carddisp.pl?gene=PAX2
HSPB2	Heat Shock Protein Family B (Small) Member 2	Protein Coding	46	GC11P112012	2.450934172	https://www.genecards.org/cgi-bin/carddisp.pl?gene=HSPB2
BLMH	Bleomycin Hydrolase	Protein Coding	50	GC17M030248	2.450750828	https://www.genecards.org/cgi-bin/carddisp.pl?gene=BLMH
AIF1	Allograft Inflammatory Factor 1	Protein Coding	45	GC06P092153	2.450322866	https://www.genecards.org/cgi-bin/carddisp.pl?gene=AIF1
NRG3	Neuregulin 3	Protein Coding	50	GC10P094473	2.449428082	https://www.genecards.org/cgi-bin/carddisp.pl?gene=NRG3

VEGFC	Vascular Endothelial Growth Factor C	Protein Coding	53	GC04M176683	2.448032379	https://www.genecards.org/cgi-bin/carddisp.pl?gene=VEGFC
PIGW	Phosphatidylinositol Glycan Anchor Biosynthesis Class W	Protein Coding	43	GC17P036534	2.446613312	https://www.genecards.org/cgi-bin/carddisp.pl?gene=PIGW
SPRY2	Sprouty RTK Signaling Antagonist 2	Protein Coding	52	GC13M080335	2.445711136	https://www.genecards.org/cgi-bin/carddisp.pl?gene=SPRY2
IQGAP1	IQ Motif Containing GTPase Activating Protein 1	Protein Coding	50	GC15P090388	2.445117474	https://www.genecards.org/cgi-bin/carddisp.pl?gene=IQGAP1
PVALB	Parvalbumin	Protein Coding	47	GC22M036800	2.444863796	https://www.genecards.org/cgi-bin/carddisp.pl?gene=PVALB
AMDHD1	Amidohydrolase Domain Containing 1	Protein Coding	43	GC12P095943	2.444291115	https://www.genecards.org/cgi-bin/carddisp.pl?gene=AMDHD1
BAK1	BCL2 Antagonist/Killer 1	Protein Coding	52	GC06M033572	2.443449974	https://www.genecards.org/cgi-bin/carddisp.pl?gene=BAK1
NRG1	Neuregulin 1	Protein Coding	55	GC08P031639	2.442706108	https://www.genecards.org/cgi-bin/carddisp.pl?gene=NRG1
CPB2	Carboxypeptidase B2	Protein Coding	51	GC13M046053	2.441707611	https://www.genecards.org/cgi-bin/carddisp.pl?gene=CPB2
EZR	Ezrin	Protein Coding	53	GC06M158765	2.441425562	https://www.genecards.org/cgi-bin/carddisp.pl?gene=EZR
AACS	Acetoacetyl-CoA Synthetase	Protein Coding	46	GC12P125065	2.440374613	https://www.genecards.org/cgi-bin/carddisp.pl?gene=AACS
BACH2	BTB Domain And CNC Homolog 2	Protein Coding	50	GC06M089926	2.43955183	https://www.genecards.org/cgi-bin/carddisp.pl?gene=BACH2
NTF3	Neurotrophin 3	Protein Coding	51	GC12P023503	2.429748535	https://www.genecards.org/cgi-bin/carddisp.pl?gene=NTF3
APLP2	Amyloid Beta Precursor Like Protein 2	Protein Coding	49	GC11P130069	2.429710865	https://www.genecards.org/cgi-bin/carddisp.pl?gene=APLP2
EIF4G1	Eukaryotic Translation Initiation Factor 4 Gamma 1	Protein Coding	54	GC03P184314	2.428409338	https://www.genecards.org/cgi-bin/carddisp.pl?gene=EIF4G1

MAP2	Microtubule Associated Protein 2	Protein Coding	51	GC02P209424	2.428044796	https://www.genecards.org/cgi-bin/carddisp.pl?gene=MAP2
PGF	Placental Growth Factor	Protein Coding	50	GC14M074941	2.426606417	https://www.genecards.org/cgi-bin/carddisp.pl?gene=PGF
WEE1	WEE1 G2 Checkpoint Kinase	Protein Coding	54	GC11P009573	2.426380873	https://www.genecards.org/cgi-bin/carddisp.pl?gene=WEE1
FCGRT	Fc Gamma Receptor And Transporter	Protein Coding	50	GC19P049506	2.42273283	https://www.genecards.org/cgi-bin/carddisp.pl?gene=FCGRT
CCL27	C-C Motif Chemokine Ligand 27	Protein Coding	42	GC09M034662	2.421966791	https://www.genecards.org/cgi-bin/carddisp.pl?gene=CCL27
CD9	CD9 Molecule	Protein Coding	51	GC12P023510	2.421341419	https://www.genecards.org/cgi-bin/carddisp.pl?gene=CD9
MSN	Moesin	Protein Coding	55	GC0XP065588	2.420360327	https://www.genecards.org/cgi-bin/carddisp.pl?gene=MSN
LOC110806262	Solute Carrier Family 6 Member 4 Gene Promoter	Functional Element	3	GC17P030235	2.417027235	https://www.genecards.org/cgi-bin/carddisp.pl?gene=LOC110806262
BDKRB2	Bradykinin Receptor B2	Protein Coding	52	GC14P096205	2.415146828	https://www.genecards.org/cgi-bin/carddisp.pl?gene=BDKRB2
GGCT	Gamma-Glutamylcyclotransferase	Protein Coding	46	GC07M030496	2.414832115	https://www.genecards.org/cgi-bin/carddisp.pl?gene=GGCT
MPZ	Myelin Protein Zero	Protein Coding	52	GC01M161304	2.414045811	https://www.genecards.org/cgi-bin/carddisp.pl?gene=MPZ
BMPR1B	Bone Morphogenetic Protein Receptor Type 1B	Protein Coding	56	GC04P094757	2.412981272	https://www.genecards.org/cgi-bin/carddisp.pl?gene=BMPR1B
CHGB	Chromogranin B	Protein Coding	48	GC20P005911	2.411474943	https://www.genecards.org/cgi-bin/carddisp.pl?gene=CHGB
ITGA3	Integrin Subunit Alpha 3	Protein Coding	55	GC17P050055	2.40312171	https://www.genecards.org/cgi-bin/carddisp.pl?gene=ITGA3
TPT1	Tumor Protein, Translationally-Controlled 1	Protein Coding	53	GC13M045333	2.400903463	https://www.genecards.org/cgi-bin/carddisp.pl?gene=TPT1

PIK3R3	Phosphoinositide-3-Kinase Regulatory Subunit 3	Protein Coding	50	GC01M046041	2.399698496	https://www.genecards.org/cgi-bin/carddisp.pl?gene=PIK3R3
FSHB	Follicle Stimulating Hormone Subunit Beta	Protein Coding	51	GC11P030210	2.397188187	https://www.genecards.org/cgi-bin/carddisp.pl?gene=FSHB
GABBR1	Gamma-Aminobutyric Acid Type B Receptor Subunit 1	Protein Coding	53	GC06M029555	2.396708727	https://www.genecards.org/cgi-bin/carddisp.pl?gene=GABBR1
PICK1	Protein Interacting With PRKCA 1	Protein Coding	50	GC22P038056	2.396637917	https://www.genecards.org/cgi-bin/carddisp.pl?gene=PICK1
CAMK4	Calcium/Calmodulin Dependent Protein Kinase IV	Protein Coding	52	GC05P111223	2.395980835	https://www.genecards.org/cgi-bin/carddisp.pl?gene=CAMK4
SND1	Staphylococcal Nuclease And Tudor Domain Containing 1	Protein Coding	48	GC07P127652	2.395704985	https://www.genecards.org/cgi-bin/carddisp.pl?gene=SND1
HDAC7	Histone Deacetylase 7	Protein Coding	53	GC12M047782	2.395572424	https://www.genecards.org/cgi-bin/carddisp.pl?gene=HDAC7
SERPINB3	Serpin Family B Member 3	Protein Coding	47	GC18M063655	2.395550489	https://www.genecards.org/cgi-bin/carddisp.pl?gene=SERPINB3
EGR1	Early Growth Response 1	Protein Coding	52	GC05P138465	2.392300129	https://www.genecards.org/cgi-bin/carddisp.pl?gene=EGR1
IRS4	Insulin Receptor Substrate 4	Protein Coding	47	GC0XM108720	2.391109467	https://www.genecards.org/cgi-bin/carddisp.pl?gene=IRS4
EIF3A	Eukaryotic Translation Initiation Factor 3 Subunit A	Protein Coding	51	GC10M119034	2.389075279	https://www.genecards.org/cgi-bin/carddisp.pl?gene=EIF3A
GDNF	Glial Cell Derived Neurotrophic Factor	Protein Coding	56	GC05M037812	2.386838436	https://www.genecards.org/cgi-bin/carddisp.pl?gene=GDNF
ARRB1	Arrestin Beta 1	Protein Coding	51	GC11M100029	2.385669708	https://www.genecards.org/cgi-bin/carddisp.pl?gene=ARRB1
KRT5	Keratin 5	Protein Coding	52	GC12M052514	2.385361433	https://www.genecards.org/cgi-bin/carddisp.pl?gene=KRT5
SLC22A9	Solute Carrier Family 22 Member 9	Protein Coding	43	GC11P063388	2.381622076	https://www.genecards.org/cgi-bin/carddisp.pl?gene=SLC22A9

MUC16	Mucin 16, Cell Surface Associated	Protein Coding	44	GC19M008848	2.38140893	https://www.genecards.org/cgi-bin/carddisp.pl?gene=MUC16
ITCH	Itchy E3 Ubiquitin Protein Ligase	Protein Coding	53	GC20P034363	2.380997658	https://www.genecards.org/cgi-bin/carddisp.pl?gene=ITCH
VCL	Vinculin	Protein Coding	54	GC10P073995	2.380882263	https://www.genecards.org/cgi-bin/carddisp.pl?gene=VCL
TUBA1B	Tubulin Alpha 1b	Protein Coding	51	GC12M049127	2.375025034	https://www.genecards.org/cgi-bin/carddisp.pl?gene=TUBA1B
EEF1D	Eukaryotic Translation Elongation Factor 1 Delta	Protein Coding	47	GC08M143579	2.374794006	https://www.genecards.org/cgi-bin/carddisp.pl?gene=EEF1D
CAMP	Cathelicidin Antimicrobial Peptide	Protein Coding	46	GC03P049961	2.373255253	https://www.genecards.org/cgi-bin/carddisp.pl?gene=CAMP
ITGB4	Integrin Subunit Beta 4	Protein Coding	56	GC17P075721	2.372350454	https://www.genecards.org/cgi-bin/carddisp.pl?gene=ITGB4
ASIC1	Acid Sensing Ion Channel Subunit 1	Protein Coding	52	GC12P050057	2.370299101	https://www.genecards.org/cgi-bin/carddisp.pl?gene=ASIC1
GTF2I	General Transcription Factor IIi	Protein Coding	47	GC07P076418	2.37008667	https://www.genecards.org/cgi-bin/carddisp.pl?gene=GTF2I
CYB561	Cytochrome B561	Protein Coding	48	GC17M063432	2.368448734	https://www.genecards.org/cgi-bin/carddisp.pl?gene=CYB561
MINPP1	Multiple Inositol-Polyphosphate Phosphatase 1	Protein Coding	53	GC10P087504	2.367403507	https://www.genecards.org/cgi-bin/carddisp.pl?gene=MINPP1
STMN1	Stathmin 1	Protein Coding	51	GC01M025884	2.365449429	https://www.genecards.org/cgi-bin/carddisp.pl?gene=STMN1
CISH	Cytokine Inducible SH2 Containing Protein	Protein Coding	52	GC03M051766	2.364514351	https://www.genecards.org/cgi-bin/carddisp.pl?gene=CISH
PELP1	Proline, Glutamate And Leucine Rich Protein 1	Protein Coding	46	GC17M004669	2.364390135	https://www.genecards.org/cgi-bin/carddisp.pl?gene=PELP1
CXADR	CXADR Ig-Like Cell Adhesion Molecule	Protein Coding	50	GC21P017513	2.361170053	https://www.genecards.org/cgi-bin/carddisp.pl?gene=CXADR

CTTN	Cortactin	Protein Coding	50	GC11P070398	2.358932734	https://www.genecards.org/cgi-bin/carddisp.pl?gene=CTTN
FOLR3	Folate Receptor Gamma	Protein Coding	46	GC11P072114	2.358604431	https://www.genecards.org/cgi-bin/carddisp.pl?gene=FOLR3
HAT1	Histone Acetyltransferase 1	Protein Coding	49	GC02P171922	2.356448412	https://www.genecards.org/cgi-bin/carddisp.pl?gene=HAT1
IFI27	Interferon Alpha Inducible Protein 27	Protein Coding	44	GC14P094104	2.356122971	https://www.genecards.org/cgi-bin/carddisp.pl?gene=IFI27
RYR2	Ryanodine Receptor 2	Protein Coding	53	GC01P237042	2.352290869	https://www.genecards.org/cgi-bin/carddisp.pl?gene=RYR2
RHO	Rhodopsin	Protein Coding	55	GC03P134990	2.350380659	https://www.genecards.org/cgi-bin/carddisp.pl?gene=RHO
HAVCR1	Hepatitis A Virus Cellular Receptor 1	Protein Coding	50	GC05M157028	2.349440813	https://www.genecards.org/cgi-bin/carddisp.pl?gene=HAVCR1
OR51E2	Olfactory Receptor Family 51 Subfamily E Member 2	Protein Coding	38	GC11M004680	2.346513271	https://www.genecards.org/cgi-bin/carddisp.pl?gene=OR51E2
CA14	Carbonic Anhydrase 14	Protein Coding	50	GC01P150436	2.342910767	https://www.genecards.org/cgi-bin/carddisp.pl?gene=CA14
BCL2L11	BCL2 Like 11	Protein Coding	52	GC02P111119	2.341534853	https://www.genecards.org/cgi-bin/carddisp.pl?gene=BCL2L11
IRF5	Interferon Regulatory Factor 5	Protein Coding	53	GC07P128937	2.339133978	https://www.genecards.org/cgi-bin/carddisp.pl?gene=IRF5
GPRC6A	G Protein-Coupled Receptor Class C Group 6 Member A	Protein Coding	44	GC06M116793	2.336325645	https://www.genecards.org/cgi-bin/carddisp.pl?gene=GPRC6A
S1PR1	Sphingosine-1-Phosphate Receptor 1	Protein Coding	52	GC01P101236	2.336192608	https://www.genecards.org/cgi-bin/carddisp.pl?gene=S1PR1
H2AX	H2A.X Variant Histone	Protein Coding	50	GC11M119285	2.332493782	https://www.genecards.org/cgi-bin/carddisp.pl?gene=H2AX
MITF	Melanocyte Inducing Transcription Factor	Protein Coding	55	GC03P069788	2.331109762	https://www.genecards.org/cgi-bin/carddisp.pl?gene=MITF

RGS2	Regulator Of G Protein Signaling 2	Protein Coding	48	GC01P192809	2.330634117	https://www.genecards.org/cgi-bin/carddisp.pl?gene=RGS2
LPAR3	Lysophosphatidic Acid Receptor 3	Protein Coding	50	GC01M084811	2.325584888	https://www.genecards.org/cgi-bin/carddisp.pl?gene=LPAR3
TGFB3	Transforming Growth Factor Beta 3	Protein Coding	54	GC14M075958	2.324998617	https://www.genecards.org/cgi-bin/carddisp.pl?gene=TGFB3
PTP4A2	Protein Tyrosine Phosphatase 4A2	Protein Coding	48	GC01M031910	2.324296236	https://www.genecards.org/cgi-bin/carddisp.pl?gene=PTP4A2
TNFRSF10B	TNF Receptor Superfamily Member 10b	Protein Coding	57	GC08M023020	2.323630333	https://www.genecards.org/cgi-bin/carddisp.pl?gene=TNFRSF10B
SLC15A2	Solute Carrier Family 15 Member 2	Protein Coding	47	GC03P121894	2.316164017	https://www.genecards.org/cgi-bin/carddisp.pl?gene=SLC15A2
CLEC7A	C-Type Lectin Domain Containing 7A	Protein Coding	52	GC12M022628	2.315096617	https://www.genecards.org/cgi-bin/carddisp.pl?gene=CLEC7A
OXSM	3-Oxoacyl-ACP Synthase, Mitochondrial	Protein Coding	44	GC03P025782	2.312613249	https://www.genecards.org/cgi-bin/carddisp.pl?gene=OXSM
GNRHR	Gonadotropin Releasing Hormone Receptor	Protein Coding	54	GC04M067737	2.305584192	https://www.genecards.org/cgi-bin/carddisp.pl?gene=GNRHR
PTPRD	Protein Tyrosine Phosphatase Receptor Type D	Protein Coding	52	GC09M008307	2.301552773	https://www.genecards.org/cgi-bin/carddisp.pl?gene=PTPRD
NPHS2	NPHS2 Stomatin Family Member, Podocin	Protein Coding	50	GC01M179554	2.300552607	https://www.genecards.org/cgi-bin/carddisp.pl?gene=NPHS2
SLC9A3R1	SLC9A3 Regulator 1	Protein Coding	52	GC17P074749	2.29944706	https://www.genecards.org/cgi-bin/carddisp.pl?gene=SLC9A3R1
KRT1	Keratin 1	Protein Coding	53	GC12M052674	2.297845602	https://www.genecards.org/cgi-bin/carddisp.pl?gene=KRT1
EWSR1	EWS RNA Binding Protein 1	Protein Coding	51	GC22P044790	2.296663523	https://www.genecards.org/cgi-bin/carddisp.pl?gene=EWSR1
SMG1	SMG1 Nonsense Mediated MRNA Decay Associated PI3K Related Kinase	Protein Coding	46	GC16M019413	2.292959452	https://www.genecards.org/cgi-bin/carddisp.pl?gene=SMG1

CAMK2A	Calcium/Calmodulin Dependent Protein Kinase II Alpha	Protein Coding	59	GC05M150219	2.292059183	https://www.genecards.org/cgi-bin/carddisp.pl?gene=CAMK2A
CHRNA7	Cholinergic Receptor Nicotinic Alpha 7 Subunit	Protein Coding	52	GC15P031923	2.290010691	https://www.genecards.org/cgi-bin/carddisp.pl?gene=CHRNA7
RAP1A	RAP1A, Member Of RAS Oncogene Family	Protein Coding	51	GC01P111542	2.289759874	https://www.genecards.org/cgi-bin/carddisp.pl?gene=RAP1A
EIF2AK2	Eukaryotic Translation Initiation Factor 2 Alpha Kinase 2	Protein Coding	56	GC02M037099	2.28935051	https://www.genecards.org/cgi-bin/carddisp.pl?gene=EIF2AK2
IGHG1	Immunoglobulin Heavy Constant Gamma 1 (G1m Marker)	Protein Coding	40	GC14M105736	2.288361549	https://www.genecards.org/cgi-bin/carddisp.pl?gene=IGHG1
PKN2	Protein Kinase N2	Protein Coding	52	GC01P088684	2.288004875	https://www.genecards.org/cgi-bin/carddisp.pl?gene=PKN2
KRT17	Keratin 17	Protein Coding	51	GC17M041619	2.285661221	https://www.genecards.org/cgi-bin/carddisp.pl?gene=KRT17
PKN1	Protein Kinase N1	Protein Coding	56	GC19P014433	2.285643816	https://www.genecards.org/cgi-bin/carddisp.pl?gene=PKN1
TTL	Tubulin Tyrosine Ligase	Protein Coding	46	GC02P123790	2.280835867	https://www.genecards.org/cgi-bin/carddisp.pl?gene=TTL
GSTT2	Glutathione S-Transferase Theta 2 (Gene/Pseudogene)	Protein Coding	40	GC22P023980	2.280731201	https://www.genecards.org/cgi-bin/carddisp.pl?gene=GSTT2
NPPC	Natriuretic Peptide C	Protein Coding	48	GC02M231921	2.280587196	https://www.genecards.org/cgi-bin/carddisp.pl?gene=NPPC
CR2	Complement C3d Receptor 2	Protein Coding	53	GC01P207454	2.280513048	https://www.genecards.org/cgi-bin/carddisp.pl?gene=CR2
TUBB3	Tubulin Beta 3 Class III	Protein Coding	55	GC16P091607	2.279607296	https://www.genecards.org/cgi-bin/carddisp.pl?gene=TUBB3
PHOX2B	Paired Like Homeobox 2B	Protein Coding	51	GC04M041746	2.278419018	https://www.genecards.org/cgi-bin/carddisp.pl?gene=PHOX2B
VGF	VGF Nerve Growth Factor Inducible	Protein Coding	45	GC07M101162	2.278366566	https://www.genecards.org/cgi-bin/carddisp.pl?gene=VGF

SERPINH1	Serpin Family H Member 1	Protein Coding	53	GC11P075562	2.277401924	https://www.genecards.org/cgi-bin/carddisp.pl?gene=SERPINH1
GYPB	Glycophorin B (MNS Blood Group)	Protein Coding	43	GC04M143996	2.275601149	https://www.genecards.org/cgi-bin/carddisp.pl?gene=GYPB
WNT1	Wnt Family Member 1	Protein Coding	52	GC12P049566	2.275330067	https://www.genecards.org/cgi-bin/carddisp.pl?gene=WNT1
PTGER2	Prostaglandin E Receptor 2	Protein Coding	55	GC14P052314	2.275265217	https://www.genecards.org/cgi-bin/carddisp.pl?gene=PTGER2
JUNB	JunB Proto-Oncogene, AP-1 Transcription Factor Subunit	Protein Coding	48	GC19P012791	2.274892807	https://www.genecards.org/cgi-bin/carddisp.pl?gene=JUNB
CTNND1	Catenin Delta 1	Protein Coding	52	GC11P058206	2.270763636	https://www.genecards.org/cgi-bin/carddisp.pl?gene=CTNND1
VNN2	Vanin 2	Protein Coding	48	GC06M132743	2.269202709	https://www.genecards.org/cgi-bin/carddisp.pl?gene=VNN2
KAT2A	Lysine Acetyltransferase 2A	Protein Coding	55	GC17M042113	2.268852472	https://www.genecards.org/cgi-bin/carddisp.pl?gene=KAT2A
MST1R	Macrophage Stimulating 1 Receptor	Protein Coding	56	GC03M051598	2.268038988	https://www.genecards.org/cgi-bin/carddisp.pl?gene=MST1R
SRF	Serum Response Factor	Protein Coding	50	GC06P043171	2.267645597	https://www.genecards.org/cgi-bin/carddisp.pl?gene=SRF
GLCCI1	Glucocorticoid Induced 1	Protein Coding	43	GC07P007974	2.263047218	https://www.genecards.org/cgi-bin/carddisp.pl?gene=GLCCI1
RPL3	Ribosomal Protein L3	Protein Coding	48	GC22M061482	2.262486219	https://www.genecards.org/cgi-bin/carddisp.pl?gene=RPL3
CHEK1	Checkpoint Kinase 1	Protein Coding	56	GC11P125625	2.262406826	https://www.genecards.org/cgi-bin/carddisp.pl?gene=CHEK1
NPY2R	Neuropeptide Y Receptor Y2	Protein Coding	51	GC04P155173	2.261928558	https://www.genecards.org/cgi-bin/carddisp.pl?gene=NPY2R
SSTR2	Somatostatin Receptor 2	Protein Coding	53	GC17P073165	2.258113146	https://www.genecards.org/cgi-bin/carddisp.pl?gene=SSTR2

EPX	Eosinophil Peroxidase	Protein Coding	50	GC17P058192	2.257993698	https://www.genecards.org/cgi-bin/carddisp.pl?gene=EPX
CALCR	Calcitonin Receptor	Protein Coding	55	GC07M093424	2.256989479	https://www.genecards.org/cgi-bin/carddisp.pl?gene=CALCR
CD33	CD33 Molecule	Protein Coding	52	GC19P073323	2.256884336	https://www.genecards.org/cgi-bin/carddisp.pl?gene=CD33
PLA2G2D	Phospholipase A2 Group IID	Protein Coding	49	GC01M020111	2.250037909	https://www.genecards.org/cgi-bin/carddisp.pl?gene=PLA2G2D
KRT4	Keratin 4	Protein Coding	48	GC12M052806	2.247910023	https://www.genecards.org/cgi-bin/carddisp.pl?gene=KRT4
NCF2	Neutrophil Cytosolic Factor 2	Protein Coding	54	GC01M184057	2.243585348	https://www.genecards.org/cgi-bin/carddisp.pl?gene=NCF2
TRB	T Cell Receptor Beta Locus	Protein Coding	25	GC07P150792	2.242851019	https://www.genecards.org/cgi-bin/carddisp.pl?gene=TRB
GRM2	Glutamate Metabotropic Receptor 2	Protein Coding	52	GC03P051707	2.236067772	https://www.genecards.org/cgi-bin/carddisp.pl?gene=GRM2
AXL	AXL Receptor Tyrosine Kinase	Protein Coding	59	GC19P041219	2.235015631	https://www.genecards.org/cgi-bin/carddisp.pl?gene=AXL
FLT4	Fms Related Receptor Tyrosine Kinase 4	Protein Coding	59	GC05M180644	2.23460269	https://www.genecards.org/cgi-bin/carddisp.pl?gene=FLT4
RASA1	RAS P21 Protein Activator 1	Protein Coding	52	GC05P087267	2.232948542	https://www.genecards.org/cgi-bin/carddisp.pl?gene=RASA1
NOP2	NOP2 Nucleolar Protein	Protein Coding	44	GC12M006556	2.232879877	https://www.genecards.org/cgi-bin/carddisp.pl?gene=NOP2
ANXA6	Annexin A6	Protein Coding	49	GC05M151100	2.230432749	https://www.genecards.org/cgi-bin/carddisp.pl?gene=ANXA6
CD14	CD14 Molecule	Protein Coding	53	GC05M140631	2.229529858	https://www.genecards.org/cgi-bin/carddisp.pl?gene=CD14
SLC18A1	Solute Carrier Family 18 Member A1	Protein Coding	49	GC08M020144	2.224712849	https://www.genecards.org/cgi-bin/carddisp.pl?gene=SLC18A1

PCM1	Pericentriolar Material 1	Protein Coding	48	GC08P017922	2.223807573	https://www.genecards.org/cgi-bin/carddisp.pl?gene=PCM1
TSLP	Thymic Stromal Lymphopoietin	Protein Coding	46	GC05P111070	2.220189095	https://www.genecards.org/cgi-bin/carddisp.pl?gene=TSLP
ITGAV	Integrin Subunit Alpha V	Protein Coding	55	GC02P186589	2.220042944	https://www.genecards.org/cgi-bin/carddisp.pl?gene=ITGAV
CASP7	Caspase 7	Protein Coding	56	GC10P113679	2.215365887	https://www.genecards.org/cgi-bin/carddisp.pl?gene=CASP7
RRAS2	RAS Related 2	Protein Coding	55	GC11M014299	2.213180065	https://www.genecards.org/cgi-bin/carddisp.pl?gene=RRAS2
CANT1	Calcium Activated Nucleotidase 1	Protein Coding	51	GC17M078992	2.212469816	https://www.genecards.org/cgi-bin/carddisp.pl?gene=CANT1
FGF9	Fibroblast Growth Factor 9	Protein Coding	51	GC13P021671	2.206700563	https://www.genecards.org/cgi-bin/carddisp.pl?gene=FGF9
NECTIN2	Nectin Cell Adhesion Molecule 2	Protein Coding	51	GC19P073053	2.205529928	https://www.genecards.org/cgi-bin/carddisp.pl?gene=NECTIN2
P2RY2	Purinergic Receptor P2Y2	Protein Coding	51	GC11P073202	2.203983545	https://www.genecards.org/cgi-bin/carddisp.pl?gene=P2RY2
SLC5A12	Solute Carrier Family 5 Member 12	Protein Coding	43	GC11M026648	2.202407837	https://www.genecards.org/cgi-bin/carddisp.pl?gene=SLC5A12
MTA1	Metastasis Associated 1	Protein Coding	50	GC14P105419	2.200025558	https://www.genecards.org/cgi-bin/carddisp.pl?gene=MTA1
CD247	CD247 Molecule	Protein Coding	56	GC01M167399	2.198839188	https://www.genecards.org/cgi-bin/carddisp.pl?gene=CD247
CXCL1	C-X-C Motif Chemokine Ligand 1	Protein Coding	50	GC04P073869	2.19849968	https://www.genecards.org/cgi-bin/carddisp.pl?gene=CXCL1
AP2B1	Adaptor Related Protein Complex 2 Subunit Beta 1	Protein Coding	49	GC17P035578	2.197745085	https://www.genecards.org/cgi-bin/carddisp.pl?gene=AP2B1
PALLD	Palladin, Cytoskeletal Associated Protein	Protein Coding	49	GC04P168497	2.195952415	https://www.genecards.org/cgi-bin/carddisp.pl?gene=PALLD

KCNJ6	Potassium Inwardly Rectifying Channel Subfamily J Member 6	Protein Coding	53	GC21M037607	2.194558859	https://www.genecards.org/cgi-bin/carddisp.pl?gene=KCNJ6
PXN	Paxillin	Protein Coding	52	GC12M120210	2.194096565	https://www.genecards.org/cgi-bin/carddisp.pl?gene=PXN
FGF1	Fibroblast Growth Factor 1	Protein Coding	54	GC05M142555	2.19223547	https://www.genecards.org/cgi-bin/carddisp.pl?gene=FGF1
TOR1A	Torsin Family 1 Member A	Protein Coding	52	GC09M129812	2.189523697	https://www.genecards.org/cgi-bin/carddisp.pl?gene=TOR1A
IFNAR2	Interferon Alpha And Beta Receptor Subunit 2	Protein Coding	56	GC21P033229	2.188015223	https://www.genecards.org/cgi-bin/carddisp.pl?gene=IFNAR2
LORICRIN	Loricrin Cornified Envelope Precursor Protein	Protein Coding	41	GC01P153262	2.187447548	https://www.genecards.org/cgi-bin/carddisp.pl?gene=LORICRIN
UGT2A1	UDP Glucuronosyltransferase Family 2 Member A1 Complex Locus	Protein Coding	39	GC04M069588	2.187245607	https://www.genecards.org/cgi-bin/carddisp.pl?gene=UGT2A1
DNAJA3	DnaJ Heat Shock Protein Family (Hsp40) Member A3	Protein Coding	47	GC16P004425	2.186523199	https://www.genecards.org/cgi-bin/carddisp.pl?gene=DNAJA3
NPHP1	Nephrocystin 1	Protein Coding	50	GC02M110122	2.185429335	https://www.genecards.org/cgi-bin/carddisp.pl?gene=NPHP1
ELK1	ETS Transcription Factor ELK1	Protein Coding	50	GC0XM047635	2.183863878	https://www.genecards.org/cgi-bin/carddisp.pl?gene=ELK1
GAS6	Growth Arrest Specific 6	Protein Coding	51	GC13M113820	2.181773901	https://www.genecards.org/cgi-bin/carddisp.pl?gene=GAS6
RPL8	Ribosomal Protein L8	Protein Coding	47	GC08M145667	2.181601048	https://www.genecards.org/cgi-bin/carddisp.pl?gene=RPL8
NBAS	NBAS Subunit Of NRZ Tethering Complex	Protein Coding	47	GC02M014791	2.18016696	https://www.genecards.org/cgi-bin/carddisp.pl?gene=NBAS
HARS2	Histidyl-TRNA Synthetase 2, Mitochondrial	Protein Coding	51	GC05P146979	2.180040359	https://www.genecards.org/cgi-bin/carddisp.pl?gene=HARS2
TIAM1	TIAM Rac1 Associated GEF 1	Protein Coding	51	GC21M031118	2.179636002	https://www.genecards.org/cgi-bin/carddisp.pl?gene=TIAM1

TRAF2	TNF Receptor Associated Factor 2	Protein Coding	51	GC09P136881	2.178793192	https://www.genecards.org/cgi-bin/carddisp.pl?gene=TRAF2
RCAN1	Regulator Of Calcineurin 1	Protein Coding	49	GC21M034513	2.1757195	https://www.genecards.org/cgi-bin/carddisp.pl?gene=RCAN1
TUBG1	Tubulin Gamma 1	Protein Coding	53	GC17P042609	2.174733877	https://www.genecards.org/cgi-bin/carddisp.pl?gene=TUBG1
LAP3	Leucine Aminopeptidase 3	Protein Coding	48	GC04P019261	2.167632818	https://www.genecards.org/cgi-bin/carddisp.pl?gene=LAP3
MMP12	Matrix Metallopeptidase 12	Protein Coding	53	GC11M102862	2.162835836	https://www.genecards.org/cgi-bin/carddisp.pl?gene=MMP12
APEH	Acylaminoacyl-Peptide Hydrolase	Protein Coding	47	GC03P049673	2.162119865	https://www.genecards.org/cgi-bin/carddisp.pl?gene=APEH
MOGAT3	Monoacylglycerol O-Acyltransferase 3	Protein Coding	38	GC07M101192	2.160770655	https://www.genecards.org/cgi-bin/carddisp.pl?gene=MOGAT3
POU2F2	POU Class 2 Homeobox 2	Protein Coding	48	GC19M042086	2.157357693	https://www.genecards.org/cgi-bin/carddisp.pl?gene=POU2F2
KDM4A	Lysine Demethylase 4A	Protein Coding	51	GC01P043650	2.156814098	https://www.genecards.org/cgi-bin/carddisp.pl?gene=KDM4A
CD86	CD86 Molecule	Protein Coding	51	GC03P122055	2.156406641	https://www.genecards.org/cgi-bin/carddisp.pl?gene=CD86
GRPR	Gastrin Releasing Peptide Receptor	Protein Coding	50	GC0XP016141	2.155606985	https://www.genecards.org/cgi-bin/carddisp.pl?gene=GRPR
CHRM2	Cholinergic Receptor Muscarinic 2	Protein Coding	55	GC07P136868	2.15210104	https://www.genecards.org/cgi-bin/carddisp.pl?gene=CHRM2
MSRB1	Methionine Sulfoxide Reductase B1	Protein Coding	45	GC16M008078	2.151757956	https://www.genecards.org/cgi-bin/carddisp.pl?gene=MSRB1
SSBP1	Single Stranded DNA Binding Protein 1	Protein Coding	47	GC07P150787	2.150444269	https://www.genecards.org/cgi-bin/carddisp.pl?gene=SSBP1
CXCL5	C-X-C Motif Chemokine Ligand 5	Protein Coding	47	GC04M073995	2.147365332	https://www.genecards.org/cgi-bin/carddisp.pl?gene=CXCL5

PPP3CA	Protein Phosphatase 3 Catalytic Subunit Alpha	Protein Coding	59	GC04M101024	2.147106171	https://www.genecards.org/cgi-bin/carddisp.pl?gene=PPP3CA
GUCA2B	Guanylate Cyclase Activator 2B	Protein Coding	44	GC01P042153	2.145024538	https://www.genecards.org/cgi-bin/carddisp.pl?gene=GUCA2B
MIP	Major Intrinsic Protein Of Lens Fiber	Protein Coding	47	GC12M056449	2.14080596	https://www.genecards.org/cgi-bin/carddisp.pl?gene=MIP
GPR139	G Protein-Coupled Receptor 139	Protein Coding	41	GC16M020042	2.138799429	https://www.genecards.org/cgi-bin/carddisp.pl?gene=GPR139
COX4I2	Cytochrome C Oxidase Subunit 4I2	Protein Coding	47	GC20P031637	2.136496305	https://www.genecards.org/cgi-bin/carddisp.pl?gene=COX4I2
ITGA4	Integrin Subunit Alpha 4	Protein Coding	55	GC02P181456	2.135978699	https://www.genecards.org/cgi-bin/carddisp.pl?gene=ITGA4
SLC5A3	Solute Carrier Family 5 Member 3	Protein Coding	46	GC21P034139	2.13568759	https://www.genecards.org/cgi-bin/carddisp.pl?gene=SLC5A3
CXCL9	C-X-C Motif Chemokine Ligand 9	Protein Coding	47	GC04M076001	2.134976625	https://www.genecards.org/cgi-bin/carddisp.pl?gene=CXCL9
EEF2K	Eukaryotic Elongation Factor 2 Kinase	Protein Coding	53	GC16P024508	2.13412714	https://www.genecards.org/cgi-bin/carddisp.pl?gene=EEF2K
MMP7	Matrix Metallopeptidase 7	Protein Coding	53	GC11M102425	2.130843878	https://www.genecards.org/cgi-bin/carddisp.pl?gene=MMP7
KAT2B	Lysine Acetyltransferase 2B	Protein Coding	55	GC03P020043	2.130774498	https://www.genecards.org/cgi-bin/carddisp.pl?gene=KAT2B
CDC25C	Cell Division Cycle 25C	Protein Coding	54	GC05M138285	2.12878871	https://www.genecards.org/cgi-bin/carddisp.pl?gene=CDC25C
UTRN	Utrophin	Protein Coding	47	GC06P144285	2.123741865	https://www.genecards.org/cgi-bin/carddisp.pl?gene=UTRN
TYRO3	TYRO3 Protein Tyrosine Kinase	Protein Coding	56	GC15P041557	2.121773958	https://www.genecards.org/cgi-bin/carddisp.pl?gene=TYRO3
MAP2K3	Mitogen-Activated Protein Kinase Kinase 3	Protein Coding	55	GC17P061212	2.121331215	https://www.genecards.org/cgi-bin/carddisp.pl?gene=MAP2K3

DPYSL2	Dihydropyrimidinase Like 2	Protein Coding	52	GC08P026514	2.119753838	https://www.genecards.org/cgi-bin/carddisp.pl?gene=DPYSL2
HDAC5	Histone Deacetylase 5	Protein Coding	56	GC17M044076	2.118413448	https://www.genecards.org/cgi-bin/carddisp.pl?gene=HDAC5
TPST2	Tyrosylprotein Sulfotransferase 2	Protein Coding	47	GC22M061183	2.113404989	https://www.genecards.org/cgi-bin/carddisp.pl?gene=TPST2
MERTK	MER Proto-Oncogene, Tyrosine Kinase	Protein Coding	59	GC02P111898	2.112577915	https://www.genecards.org/cgi-bin/carddisp.pl?gene=MERTK
NGB	Neuroglobin	Protein Coding	43	GC14M077265	2.111859798	https://www.genecards.org/cgi-bin/carddisp.pl?gene=NGB
PTP4A1	Protein Tyrosine Phosphatase 4A1	Protein Coding	47	GC06P092552	2.11169529	https://www.genecards.org/cgi-bin/carddisp.pl?gene=PTP4A1
GIT2	GIT ArfGAP 2	Protein Coding	48	GC12M109929	2.111174107	https://www.genecards.org/cgi-bin/carddisp.pl?gene=GIT2
SH2D1A	SH2 Domain Containing 1A	Protein Coding	53	GC0XP124227	2.110352278	https://www.genecards.org/cgi-bin/carddisp.pl?gene=SH2D1A
SORBS1	Sorbin And SH3 Domain Containing 1	Protein Coding	48	GC10M095311	2.107699394	https://www.genecards.org/cgi-bin/carddisp.pl?gene=SORBS1
NR2E3	Nuclear Receptor Subfamily 2 Group E Member 3	Protein Coding	48	GC15P071792	2.107695341	https://www.genecards.org/cgi-bin/carddisp.pl?gene=NR2E3
RNPC3	RNA Binding Region (RNP1, RRM) Containing 3	Protein Coding	42	GC01P103525	2.104146481	https://www.genecards.org/cgi-bin/carddisp.pl?gene=RNPC3
PPT2	Palmitoyl-Protein Thioesterase 2	Protein Coding	44	GC06P032153	2.102693081	https://www.genecards.org/cgi-bin/carddisp.pl?gene=PPT2
EPHA3	EPH Receptor A3	Protein Coding	53	GC03P089077	2.101714849	https://www.genecards.org/cgi-bin/carddisp.pl?gene=EPHA3
CD69	CD69 Molecule	Protein Coding	49	GC12M022623	2.100873947	https://www.genecards.org/cgi-bin/carddisp.pl?gene=CD69
NTRK3	Neurotrophic Receptor Tyrosine Kinase 3	Protein Coding	59	GC15M087859	2.100211382	https://www.genecards.org/cgi-bin/carddisp.pl?gene=NTRK3

ARHGEF2	Rho/Rac Guanine Nucleotide Exchange Factor 2	Protein Coding	52	GC01M155946	2.094944477	https://www.genecards.org/cgi-bin/carddisp.pl?gene=ARHGEF2
TNFRSF9	TNF Receptor Superfamily Member 9	Protein Coding	50	GC01M007915	2.094399691	https://www.genecards.org/cgi-bin/carddisp.pl?gene=TNFRSF9
BID	BH3 Interacting Domain Death Agonist	Protein Coding	52	GC22M017734	2.09288168	https://www.genecards.org/cgi-bin/carddisp.pl?gene=BID
RRAS	RAS Related	Protein Coding	48	GC19M049635	2.091985941	https://www.genecards.org/cgi-bin/carddisp.pl?gene=RRAS
ITGA6	Integrin Subunit Alpha 6	Protein Coding	56	GC02P172427	2.089226961	https://www.genecards.org/cgi-bin/carddisp.pl?gene=ITGA6
MTPN	Myotrophin	Protein Coding	41	GC07M135926	2.08886981	https://www.genecards.org/cgi-bin/carddisp.pl?gene=MTPN
HCK	HCK Proto-Oncogene, Src Family Tyrosine Kinase	Protein Coding	54	GC20P032052	2.088297129	https://www.genecards.org/cgi-bin/carddisp.pl?gene=HCK
FKBP1A	FKBP Prolyl Isomerase 1A	Protein Coding	54	GC20M001369	2.087401867	https://www.genecards.org/cgi-bin/carddisp.pl?gene=FKBP1A
SLC18A3	Solute Carrier Family 18 Member A3	Protein Coding	50	GC10P049610	2.086946011	https://www.genecards.org/cgi-bin/carddisp.pl?gene=SLC18A3
ADI1	Acireductone Dioxygenase 1	Protein Coding	46	GC02M003501	2.086838961	https://www.genecards.org/cgi-bin/carddisp.pl?gene=ADI1
COL4A1	Collagen Type IV Alpha 1 Chain	Protein Coding	55	GC13M110148	2.084783077	https://www.genecards.org/cgi-bin/carddisp.pl?gene=COL4A1
MASP1	MBL Associated Serine Protease 1	Protein Coding	55	GC03M187216	2.084374428	https://www.genecards.org/cgi-bin/carddisp.pl?gene=MASP1
ADORA2B	Adenosine A2b Receptor	Protein Coding	55	GC17P017963	2.084364653	https://www.genecards.org/cgi-bin/carddisp.pl?gene=ADORA2B
DLG2	Discs Large MAGUK Scaffold Protein 2	Protein Coding	48	GC11M083455	2.081746817	https://www.genecards.org/cgi-bin/carddisp.pl?gene=DLG2
PRG2	Proteoglycan 2, Pro Eosinophil Major Basic Protein	Protein Coding	47	GC11M057386	2.079038382	https://www.genecards.org/cgi-bin/carddisp.pl?gene=PRG2

KHDRBS2	KH RNA Binding Domain Containing, Signal Transduction Associated 2	Protein Coding	43	GC06M061542	2.074846268	https://www.genecards.org/cgi-bin/carddisp.pl?gene=KHDRBS2
FOXP2	Forkhead Box P2	Protein Coding	50	GC07P114086	2.072494745	https://www.genecards.org/cgi-bin/carddisp.pl?gene=FOXP2
POLK	DNA Polymerase Kappa	Protein Coding	49	GC05P075511	2.071971893	https://www.genecards.org/cgi-bin/carddisp.pl?gene=POLK
FCGR1A	Fc Gamma Receptor Ia	Protein Coding	48	GC01P150388	2.06972146	https://www.genecards.org/cgi-bin/carddisp.pl?gene=FCGR1A
RPL23A	Ribosomal Protein L23a	Protein Coding	47	GC17P028719	2.064439297	https://www.genecards.org/cgi-bin/carddisp.pl?gene=RPL23A
ATN1	Atrophin 1	Protein Coding	50	GC12P023561	2.062030077	https://www.genecards.org/cgi-bin/carddisp.pl?gene=ATN1
SFTPA1	Surfactant Protein A1	Protein Coding	51	GC10P095475	2.061035395	https://www.genecards.org/cgi-bin/carddisp.pl?gene=SFTPA1
CSF3R	Colony Stimulating Factor 3 Receptor	Protein Coding	54	GC01M036466	2.059127331	https://www.genecards.org/cgi-bin/carddisp.pl?gene=CSF3R
MYO18A	Myosin XVIIIA	Protein Coding	44	GC17M051199	2.057760477	https://www.genecards.org/cgi-bin/carddisp.pl?gene=MYO18A
GP6	Glycoprotein VI Platelet	Protein Coding	53	GC19M055013	2.057397366	https://www.genecards.org/cgi-bin/carddisp.pl?gene=GP6
HUWE1	HECT, UBA And WWE Domain Containing E3 Ubiquitin Protein Ligase 1	Protein Coding	52	GC0XM053532	2.05684495	https://www.genecards.org/cgi-bin/carddisp.pl?gene=HUWE1
SLC9A3R2	SLC9A3 Regulator 2	Protein Coding	45	GC16P013511	2.05680871	https://www.genecards.org/cgi-bin/carddisp.pl?gene=SLC9A3R2
NUDT1	Nudix Hydrolase 1	Protein Coding	48	GC07P002242	2.056804657	https://www.genecards.org/cgi-bin/carddisp.pl?gene=NUDT1
CCL3	C-C Motif Chemokine Ligand 3	Protein Coding	46	GC17M036088	2.054622173	https://www.genecards.org/cgi-bin/carddisp.pl?gene=CCL3
PSIP1	PC4 And SRSF1 Interacting Protein 1	Protein Coding	47	GC09M015464	2.052438974	https://www.genecards.org/cgi-bin/carddisp.pl?gene=PSIP1

LMO2	LIM Domain Only 2	Protein Coding	47	GC11M033858	2.05214572	https://www.genecards.org/cgi-bin/carddisp.pl?gene=LMO2
PTPRF	Protein Tyrosine Phosphatase Receptor Type F	Protein Coding	56	GC01P043527	2.049630642	https://www.genecards.org/cgi-bin/carddisp.pl?gene=PTPRF
VEGFB	Vascular Endothelial Growth Factor B	Protein Coding	51	GC11P064234	2.048155546	https://www.genecards.org/cgi-bin/carddisp.pl?gene=VEGFB
CPA1	Carboxypeptidase A1	Protein Coding	51	GC07P130380	2.048068523	https://www.genecards.org/cgi-bin/carddisp.pl?gene=CPA1
SNAI2	Snail Family Transcriptional Repressor 2	Protein Coding	51	GC08M048917	2.047676325	https://www.genecards.org/cgi-bin/carddisp.pl?gene=SNAI2
CEACAM1	CEA Cell Adhesion Molecule 1	Protein Coding	51	GC19M042507	2.047606945	https://www.genecards.org/cgi-bin/carddisp.pl?gene=CEACAM1
TPST1	Tyrosylprotein Sulfotransferase 1	Protein Coding	47	GC07P071715	2.046896458	https://www.genecards.org/cgi-bin/carddisp.pl?gene=TPST1
C3AR1	Complement C3a Receptor 1	Protein Coding	50	GC12M008059	2.04667449	https://www.genecards.org/cgi-bin/carddisp.pl?gene=C3AR1
EIF6	Eukaryotic Translation Initiation Factor 6	Protein Coding	47	GC20M035278	2.044235945	https://www.genecards.org/cgi-bin/carddisp.pl?gene=EIF6
SUMO1	Small Ubiquitin Like Modifier 1	Protein Coding	53	GC02M202206	2.042767763	https://www.genecards.org/cgi-bin/carddisp.pl?gene=SUMO1
TIE1	Tyrosine Kinase With Immunoglobulin Like And EGF Like Domains 1	Protein Coding	52	GC01P043300	2.041883707	https://www.genecards.org/cgi-bin/carddisp.pl?gene=TIE1
CENPF	Centromere Protein F	Protein Coding	48	GC01P214603	2.041686535	https://www.genecards.org/cgi-bin/carddisp.pl?gene=CENPF
TNC	Tenascin C	Protein Coding	56	GC09M115019	2.041381836	https://www.genecards.org/cgi-bin/carddisp.pl?gene=TNC
SERPINB2	Serpin Family B Member 2	Protein Coding	50	GC18P063871	2.040825367	https://www.genecards.org/cgi-bin/carddisp.pl?gene=SERPINB2
SLC47A1	Solute Carrier Family 47 Member 1	Protein Coding	48	GC17P019495	2.040024042	https://www.genecards.org/cgi-bin/carddisp.pl?gene=SLC47A1

MCAM	Melanoma Cell Adhesion Molecule	Protein Coding	49	GC11M119308	2.03592515	https://www.genecards.org/cgi-bin/carddisp.pl?gene=MCAM
G3BP1	G3BP Stress Granule Assembly Factor 1	Protein Coding	47	GC05P151771	2.033505201	https://www.genecards.org/cgi-bin/carddisp.pl?gene=G3BP1
OPRD1	Opioid Receptor Delta 1	Protein Coding	50	GC01P028812	2.032579899	https://www.genecards.org/cgi-bin/carddisp.pl?gene=OPRD1
KRT10	Keratin 10	Protein Coding	50	GC17M040818	2.029624939	https://www.genecards.org/cgi-bin/carddisp.pl?gene=KRT10
DNM3	Dynamin 3	Protein Coding	50	GC01P171868	2.028045177	https://www.genecards.org/cgi-bin/carddisp.pl?gene=DNM3
NOG	Noggin	Protein Coding	53	GC17P056593	2.026612282	https://www.genecards.org/cgi-bin/carddisp.pl?gene=NOG
HIP1	Huntingtin Interacting Protein 1	Protein Coding	47	GC07M075533	2.025650978	https://www.genecards.org/cgi-bin/carddisp.pl?gene=HIP1
DMPK	DM1 Protein Kinase	Protein Coding	55	GC19M045769	2.02176404	https://www.genecards.org/cgi-bin/carddisp.pl?gene=DMPK
MUSK	Muscle Associated Receptor Tyrosine Kinase	Protein Coding	55	GC09P110668	2.02113533	https://www.genecards.org/cgi-bin/carddisp.pl?gene=MUSK
USP1	Ubiquitin Specific Peptidase 1	Protein Coding	51	GC01P062436	2.019223452	https://www.genecards.org/cgi-bin/carddisp.pl?gene=USP1
CCNG1	Cyclin G1	Protein Coding	47	GC05P163438	2.017747879	https://www.genecards.org/cgi-bin/carddisp.pl?gene=CCNG1
BLZF1	Basic Leucine Zipper Nuclear Factor 1	Protein Coding	45	GC01P169367	2.013850212	https://www.genecards.org/cgi-bin/carddisp.pl?gene=BLZF1
MYOD1	Myogenic Differentiation 1	Protein Coding	53	GC11P017741	2.008466005	https://www.genecards.org/cgi-bin/carddisp.pl?gene=MYOD1
SLX9	SLX9 Ribosome Biogenesis Factor	Protein Coding	35	GC21P044939	2.005723715	https://www.genecards.org/cgi-bin/carddisp.pl?gene=SLX9
LGALS4	Galectin 4	Protein Coding	44	GC19M070551	2.00537014	https://www.genecards.org/cgi-bin/carddisp.pl?gene=LGALS4

ZBTB16	Zinc Finger And BTB Domain Containing 16	Protein Coding	50	GC11P114059	2.005048513	https://www.genecards.org/cgi-bin/carddisp.pl?gene=ZBTB16
PIGR	Polymeric Immunoglobulin Receptor	Protein Coding	48	GC01M206928	2.003614426	https://www.genecards.org/cgi-bin/carddisp.pl?gene=PIGR
ROR2	Receptor Tyrosine Kinase Like Orphan Receptor 2	Protein Coding	55	GC09M096500	1.999219894	https://www.genecards.org/cgi-bin/carddisp.pl?gene=ROR2
CA7	Carbonic Anhydrase 7	Protein Coding	44	GC16P066844	1.998595476	https://www.genecards.org/cgi-bin/carddisp.pl?gene=CA7
IFNGR1	Interferon Gamma Receptor 1	Protein Coding	57	GC06M137197	1.997519255	https://www.genecards.org/cgi-bin/carddisp.pl?gene=IFNGR1
CCL7	C-C Motif Chemokine Ligand 7	Protein Coding	50	GC17P034270	1.996502876	https://www.genecards.org/cgi-bin/carddisp.pl?gene=CCL7
SELPLG	Selectin P Ligand	Protein Coding	51	GC12M108621	1.995840073	https://www.genecards.org/cgi-bin/carddisp.pl?gene=SELPLG
C4B	Complement C4B (Chido Blood Group)	Protein Coding	48	GC06P032014	1.993642092	https://www.genecards.org/cgi-bin/carddisp.pl?gene=C4B
WNT2	Wnt Family Member 2	Protein Coding	50	GC07M117313	1.993524075	https://www.genecards.org/cgi-bin/carddisp.pl?gene=WNT2
LMX1B	LIM Homeobox Transcription Factor 1 Beta	Protein Coding	51	GC09P126614	1.992806673	https://www.genecards.org/cgi-bin/carddisp.pl?gene=LMX1B
RHOH	Ras Homolog Family Member H	Protein Coding	51	GC04P040192	1.990023136	https://www.genecards.org/cgi-bin/carddisp.pl?gene=RHOH
IL9	Interleukin 9	Protein Coding	50	GC05M135891	1.989136219	https://www.genecards.org/cgi-bin/carddisp.pl?gene=IL9
GRB10	Growth Factor Receptor Bound Protein 10	Protein Coding	49	GC07M050590	1.986071348	https://www.genecards.org/cgi-bin/carddisp.pl?gene=GRB10
ROBO1	Roundabout Guidance Receptor 1	Protein Coding	51	GC03M078597	1.984625578	https://www.genecards.org/cgi-bin/carddisp.pl?gene=ROBO1
COL20A1	Collagen Type XX Alpha 1 Chain	Protein Coding	44	GC20P063293	1.983115435	https://www.genecards.org/cgi-bin/carddisp.pl?gene=COL20A1

PDCD6IP	Programmed Cell Death 6 Interacting Protein	Protein Coding	50	GC03P033798	1.98204422	https://www.genecards.org/cgi-bin/carddisp.pl?gene=PDCD6IP
DCT	Dopachrome Tautomerase	Protein Coding	52	GC13M094436	1.978040814	https://www.genecards.org/cgi-bin/carddisp.pl?gene=DCT
MARCKS	Myristoylated Alanine Rich Protein Kinase C Substrate	Protein Coding	46	GC06P113857	1.976188779	https://www.genecards.org/cgi-bin/carddisp.pl?gene=MARCKS
SMAD1	SMAD Family Member 1	Protein Coding	51	GC04P145481	1.973513365	https://www.genecards.org/cgi-bin/carddisp.pl?gene=SMAD1
CD63	CD63 Molecule	Protein Coding	50	GC12M055725	1.972302675	https://www.genecards.org/cgi-bin/carddisp.pl?gene=CD63
TOP2B	DNA Topoisomerase II Beta	Protein Coding	52	GC03M025598	1.970924854	https://www.genecards.org/cgi-bin/carddisp.pl?gene=TOP2B
GADD45A	Growth Arrest And DNA Damage Inducible Alpha	Protein Coding	50	GC01P067685	1.970365047	https://www.genecards.org/cgi-bin/carddisp.pl?gene=GADD45A
HLA-DMA	Major Histocompatibility Complex, Class II, DM Alpha	Protein Coding	46	GC06M071795	1.966271639	https://www.genecards.org/cgi-bin/carddisp.pl?gene=HLA-DMA
TNFRSF8	TNF Receptor Superfamily Member 8	Protein Coding	50	GC01P012063	1.965282679	https://www.genecards.org/cgi-bin/carddisp.pl?gene=TNFRSF8
PTPRJ	Protein Tyrosine Phosphatase Receptor Type J	Protein Coding	55	GC11P048002	1.964123964	https://www.genecards.org/cgi-bin/carddisp.pl?gene=PTPRJ
ADRA1A	Adrenoceptor Alpha 1A	Protein Coding	53	GC08M026747	1.963740349	https://www.genecards.org/cgi-bin/carddisp.pl?gene=ADRA1A
FKBP4	FKBP Prolyl Isomerase 4	Protein Coding	52	GC12P002795	1.963716984	https://www.genecards.org/cgi-bin/carddisp.pl?gene=FKBP4
POLR3K	RNA Polymerase III Subunit K	Protein Coding	47	GC16M000046	1.963641405	https://www.genecards.org/cgi-bin/carddisp.pl?gene=POLR3K
MYO5A	Myosin VA	Protein Coding	51	GC15M123722	1.962192774	https://www.genecards.org/cgi-bin/carddisp.pl?gene=MYO5A
FRK	Fyn Related Src Family Tyrosine Kinase	Protein Coding	51	GC06M115931	1.962022543	https://www.genecards.org/cgi-bin/carddisp.pl?gene=FRK

SLC6A6	Solute Carrier Family 6 Member 6	Protein Coding	52	GC03P014402	1.960782766	https://www.genecards.org/cgi-bin/carddisp.pl?gene=SLC6A6
SEMA3A	Semaphorin 3A	Protein Coding	53	GC07M083955	1.9588871	https://www.genecards.org/cgi-bin/carddisp.pl?gene=SEMA3A
FZD4	Frizzled Class Receptor 4	Protein Coding	56	GC11M086945	1.957341671	https://www.genecards.org/cgi-bin/carddisp.pl?gene=FZD4
NAA10	N-Alpha-Acetyltransferase 10, NatA Catalytic Subunit	Protein Coding	52	GC0XM153929	1.954634666	https://www.genecards.org/cgi-bin/carddisp.pl?gene=NAA10
S100A7	S100 Calcium Binding Protein A7	Protein Coding	46	GC01M153457	1.948243737	https://www.genecards.org/cgi-bin/carddisp.pl?gene=S100A7
FCER2	Fc Epsilon Receptor II	Protein Coding	50	GC19M007689	1.944807529	https://www.genecards.org/cgi-bin/carddisp.pl?gene=FCER2
VASP	Vasodilator Stimulated Phosphoprotein	Protein Coding	50	GC19P073070	1.944025874	https://www.genecards.org/cgi-bin/carddisp.pl?gene=VASP
TSG101	Tumor Susceptibility 101	Protein Coding	49	GC11M018468	1.943533421	https://www.genecards.org/cgi-bin/carddisp.pl?gene=TSG101
S100A1	S100 Calcium Binding Protein A1	Protein Coding	47	GC01P153627	1.942153215	https://www.genecards.org/cgi-bin/carddisp.pl?gene=S100A1
LAT	Linker For Activation Of T Cells	Protein Coding	53	GC16P042656	1.938898325	https://www.genecards.org/cgi-bin/carddisp.pl?gene=LAT
CCR1	C-C Motif Chemokine Receptor 1	Protein Coding	52	GC03M046218	1.938135386	https://www.genecards.org/cgi-bin/carddisp.pl?gene=CCR1
LYST	Lysosomal Trafficking Regulator	Protein Coding	45	GC01M235661	1.937011361	https://www.genecards.org/cgi-bin/carddisp.pl?gene=LYST
ALCAM	Activated Leukocyte Cell Adhesion Molecule	Protein Coding	50	GC03P105366	1.936097383	https://www.genecards.org/cgi-bin/carddisp.pl?gene=ALCAM
TJP1	Tight Junction Protein 1	Protein Coding	51	GC15M029699	1.935941458	https://www.genecards.org/cgi-bin/carddisp.pl?gene=TJP1
EFNB2	Ephrin B2	Protein Coding	50	GC13M106489	1.931266308	https://www.genecards.org/cgi-bin/carddisp.pl?gene=EFNB2

STAT2	Signal Transducer And Activator Of Transcription 2	Protein Coding	55	GC12M056341	1.929269314	https://www.genecards.org/cgi-bin/carddisp.pl?gene=STAT2
RPL19	Ribosomal Protein L19	Protein Coding	48	GC17P039200	1.925439119	https://www.genecards.org/cgi-bin/carddisp.pl?gene=RPL19
HTR1B	5-Hydroxytryptamine Receptor 1B	Protein Coding	50	GC06M077488	1.925381422	https://www.genecards.org/cgi-bin/carddisp.pl?gene=HTR1B
FER	FER Tyrosine Kinase	Protein Coding	52	GC05P108747	1.925205708	https://www.genecards.org/cgi-bin/carddisp.pl?gene=FER
RAPGEF3	Rap Guanine Nucleotide Exchange Factor 3	Protein Coding	51	GC12M047736	1.919921637	https://www.genecards.org/cgi-bin/carddisp.pl?gene=RAPGEF3
PATJ	PATJ Crumbs Cell Polarity Complex Component	Protein Coding	43	GC01P061743	1.919464111	https://www.genecards.org/cgi-bin/carddisp.pl?gene=PATJ
PSMD4	Proteasome 26S Subunit Ubiquitin Receptor, Non-ATPase 4	Protein Coding	50	GC01P151378	1.917000532	https://www.genecards.org/cgi-bin/carddisp.pl?gene=PSMD4
NRXN1	Neurexin 1	Protein Coding	55	GC02M049918	1.916447639	https://www.genecards.org/cgi-bin/carddisp.pl?gene=NRXN1
RANBP9	RAN Binding Protein 9	Protein Coding	48	GC06M013621	1.916115046	https://www.genecards.org/cgi-bin/carddisp.pl?gene=RANBP9
CEACAM3	CEA Cell Adhesion Molecule 3	Protein Coding	49	GC19P041796	1.915868282	https://www.genecards.org/cgi-bin/carddisp.pl?gene=CEACAM3
MAP3K11	Mitogen-Activated Protein Kinase Kinase Kinase 11	Protein Coding	53	GC11M099713	1.914820433	https://www.genecards.org/cgi-bin/carddisp.pl?gene=MAP3K11
FGF8	Fibroblast Growth Factor 8	Protein Coding	54	GC10M101770	1.91269207	https://www.genecards.org/cgi-bin/carddisp.pl?gene=FGF8
LHX3	LIM Homeobox 3	Protein Coding	47	GC09M136196	1.912455797	https://www.genecards.org/cgi-bin/carddisp.pl?gene=LHX3
MAP3K14	Mitogen-Activated Protein Kinase Kinase Kinase 14	Protein Coding	51	GC17M045263	1.910337448	https://www.genecards.org/cgi-bin/carddisp.pl?gene=MAP3K14
MPHOSPH8	M-Phase Phosphoprotein 8	Protein Coding	43	GC13P019633	1.903998017	https://www.genecards.org/cgi-bin/carddisp.pl?gene=MPHOSPH8

SOS2	SOS Ras/Rho Guanine Nucleotide Exchange Factor 2	Protein Coding	51	GC14M050117	1.903938532	https://www.genecards.org/cgi-bin/carddisp.pl?gene=SOS2
NCAM1	Neural Cell Adhesion Molecule 1	Protein Coding	55	GC11P112961	1.903778315	https://www.genecards.org/cgi-bin/carddisp.pl?gene=NCAM1
FPR1	Formyl Peptide Receptor 1	Protein Coding	54	GC19M051745	1.9031353	https://www.genecards.org/cgi-bin/carddisp.pl?gene=FPR1
CEACAM7	CEA Cell Adhesion Molecule 7	Protein Coding	44	GC19M041673	1.902526498	https://www.genecards.org/cgi-bin/carddisp.pl?gene=CEACAM7
BCAR1	BCAR1 Scaffold Protein, Cas Family Member	Protein Coding	50	GC16M075228	1.898793936	https://www.genecards.org/cgi-bin/carddisp.pl?gene=BCAR1
MAP3K1	Mitogen-Activated Protein Kinase Kinase Kinase 1	Protein Coding	56	GC05P056815	1.898443699	https://www.genecards.org/cgi-bin/carddisp.pl?gene=MAP3K1
TRPC3	Transient Receptor Potential Cation Channel Subfamily C Member 3	Protein Coding	54	GC04M121879	1.893781662	https://www.genecards.org/cgi-bin/carddisp.pl?gene=TRPC3
DDR2	Discoidin Domain Receptor Tyrosine Kinase 2	Protein Coding	59	GC01P162631	1.891518831	https://www.genecards.org/cgi-bin/carddisp.pl?gene=DDR2
RALA	RAS Like Proto-Oncogene A	Protein Coding	52	GC07P039622	1.891065836	https://www.genecards.org/cgi-bin/carddisp.pl?gene=RALA
CCR7	C-C Motif Chemokine Receptor 7	Protein Coding	51	GC17M051716	1.886298895	https://www.genecards.org/cgi-bin/carddisp.pl?gene=CCR7
KRT9	Keratin 9	Protein Coding	48	GC17M041565	1.88619864	https://www.genecards.org/cgi-bin/carddisp.pl?gene=KRT9
SSTR5	Somatostatin Receptor 5	Protein Coding	49	GC16P001072	1.884937763	https://www.genecards.org/cgi-bin/carddisp.pl?gene=SSTR5
SEC23IP	SEC23 Interacting Protein	Protein Coding	46	GC10P119892	1.882997751	https://www.genecards.org/cgi-bin/carddisp.pl?gene=SEC23IP
CD163	CD163 Molecule	Protein Coding	51	GC12M008027	1.878556132	https://www.genecards.org/cgi-bin/carddisp.pl?gene=CD163
DRD5	Dopamine Receptor D5	Protein Coding	53	GC04P009783	1.877865791	https://www.genecards.org/cgi-bin/carddisp.pl?gene=DRD5

UBE3C	Ubiquitin Protein Ligase E3C	Protein Coding	46	GC07P157138	1.877578497	https://www.genecards.org/cgi-bin/carddisp.pl?gene=UBE3C
CARNS1	Carnosine Synthase 1	Protein Coding	39	GC11P067414	1.877417564	https://www.genecards.org/cgi-bin/carddisp.pl?gene=CARNS1
ARF6	ADP Ribosylation Factor 6	Protein Coding	49	GC14P049895	1.875149727	https://www.genecards.org/cgi-bin/carddisp.pl?gene=ARF6
KTN1	Kinectin 1	Protein Coding	48	GC14P055559	1.87413168	https://www.genecards.org/cgi-bin/carddisp.pl?gene=KTN1
PRMT3	Protein Arginine Methyltransferase 3	Protein Coding	47	GC11P020409	1.873780608	https://www.genecards.org/cgi-bin/carddisp.pl?gene=PRMT3
NUCB1	Nucleobindin 1	Protein Coding	48	GC19P048900	1.873780489	https://www.genecards.org/cgi-bin/carddisp.pl?gene=NUCB1
CDH23	Cadherin Related 23	Protein Coding	51	GC10P071396	1.872978449	https://www.genecards.org/cgi-bin/carddisp.pl?gene=CDH23
FGF10	Fibroblast Growth Factor 10	Protein Coding	53	GC05M044340	1.872734308	https://www.genecards.org/cgi-bin/carddisp.pl?gene=FGF10
HGS	Hepatocyte Growth Factor-Regulated Tyrosine Kinase Substrate	Protein Coding	50	GC17P081683	1.870378494	https://www.genecards.org/cgi-bin/carddisp.pl?gene=HGS
ADRA1B	Adrenoceptor Alpha 1B	Protein Coding	53	GC05P159867	1.867448092	https://www.genecards.org/cgi-bin/carddisp.pl?gene=ADRA1B
BLNK	B Cell Linker	Protein Coding	53	GC10M096259	1.864344835	https://www.genecards.org/cgi-bin/carddisp.pl?gene=BLNK
PTPN13	Protein Tyrosine Phosphatase Non-Receptor Type 13	Protein Coding	51	GC04P086594	1.858952284	https://www.genecards.org/cgi-bin/carddisp.pl?gene=PTPN13
WASL	WASP Like Actin Nucleation Promoting Factor	Protein Coding	48	GC07M123681	1.858193636	https://www.genecards.org/cgi-bin/carddisp.pl?gene=WASL
RPL23	Ribosomal Protein L23	Protein Coding	47	GC17M038847	1.85684371	https://www.genecards.org/cgi-bin/carddisp.pl?gene=RPL23
NEDD4	NEDD4 E3 Ubiquitin Protein Ligase	Protein Coding	54	GC15M055826	1.856402278	https://www.genecards.org/cgi-bin/carddisp.pl?gene=NEDD4

GREM1	Gremlin 1, DAN Family BMP Antagonist	Protein Coding	50	GC15P046226	1.855420828	https://www.genecards.org/cgi-bin/carddisp.pl?gene=GREM1
CSN2	Casein Beta	Protein Coding	40	GC04M069955	1.854342461	https://www.genecards.org/cgi-bin/carddisp.pl?gene=CSN2
CALB2	Calbindin 2	Protein Coding	46	GC16P071358	1.852124453	https://www.genecards.org/cgi-bin/carddisp.pl?gene=CALB2
GLRB	Glycine Receptor Beta	Protein Coding	53	GC04P157076	1.851535916	https://www.genecards.org/cgi-bin/carddisp.pl?gene=GLRB
SULT6B1	Sulfotransferase Family 6B Member 1	Protein Coding	40	GC02M037167	1.847799659	https://www.genecards.org/cgi-bin/carddisp.pl?gene=SULT6B1
EDN3	Endothelin 3	Protein Coding	51	GC20P059300	1.847703934	https://www.genecards.org/cgi-bin/carddisp.pl?gene=EDN3
S100A4	S100 Calcium Binding Protein A4	Protein Coding	52	GC01M153543	1.846493006	https://www.genecards.org/cgi-bin/carddisp.pl?gene=S100A4
NOLC1	Nucleolar And Coiled-Body Phosphoprotein 1	Protein Coding	47	GC10P102152	1.845025539	https://www.genecards.org/cgi-bin/carddisp.pl?gene=NOLC1
THY1	Thy-1 Cell Surface Antigen	Protein Coding	50	GC11M119417	1.843530655	https://www.genecards.org/cgi-bin/carddisp.pl?gene=THY1
DLG3	Discs Large MAGUK Scaffold Protein 3	Protein Coding	49	GC0XP070444	1.843284726	https://www.genecards.org/cgi-bin/carddisp.pl?gene=DLG3
GRK6	G Protein-Coupled Receptor Kinase 6	Protein Coding	53	GC05P177403	1.840342522	https://www.genecards.org/cgi-bin/carddisp.pl?gene=GRK6
TNFAIP6	TNF Alpha Induced Protein 6	Protein Coding	47	GC02P151357	1.840254068	https://www.genecards.org/cgi-bin/carddisp.pl?gene=TNFAIP6
EREG	Epiregulin	Protein Coding	50	GC04P074366	1.837180853	https://www.genecards.org/cgi-bin/carddisp.pl?gene=EREG
PDGFA	Platelet Derived Growth Factor Subunit A	Protein Coding	50	GC07M000497	1.837180853	https://www.genecards.org/cgi-bin/carddisp.pl?gene=PDGFA
ABCC11	ATP Binding Cassette Subfamily C Member 11	Protein Coding	48	GC16M048166	1.835193396	https://www.genecards.org/cgi-bin/carddisp.pl?gene=ABCC11

RPL37	Ribosomal Protein L37	Protein Coding	45	GC05M040825	1.834323168	https://www.genecards.org/cgi-bin/carddisp.pl?gene=RPL37
PLCL1	Phospholipase C Like 1 (Inactive)	Protein Coding	46	GC02P197804	1.834127903	https://www.genecards.org/cgi-bin/carddisp.pl?gene=PLCL1
AQP1	Aquaporin 1 (Colton Blood Group)	Protein Coding	53	GC07P030911	1.833886862	https://www.genecards.org/cgi-bin/carddisp.pl?gene=AQP1
FLOT2	Flotillin 2	Protein Coding	47	GC17M051195	1.832614422	https://www.genecards.org/cgi-bin/carddisp.pl?gene=FLOT2
CREM	CAMP Responsive Element Modulator	Protein Coding	48	GC10P035126	1.831388235	https://www.genecards.org/cgi-bin/carddisp.pl?gene=CREM
NRP2	Neuropilin 2	Protein Coding	52	GC02P205681	1.830303192	https://www.genecards.org/cgi-bin/carddisp.pl?gene=NRP2
BDKRB1	Bradykinin Receptor B1	Protein Coding	48	GC14P096302	1.830173969	https://www.genecards.org/cgi-bin/carddisp.pl?gene=BDKRB1
DIABLO	Diablo IAP-Binding Mitochondrial Protein	Protein Coding	53	GC12M122497	1.829569101	https://www.genecards.org/cgi-bin/carddisp.pl?gene=DIABLO
OGDHL	Oxoglutarate Dehydrogenase L	Protein Coding	47	GC10M049734	1.827629805	https://www.genecards.org/cgi-bin/carddisp.pl?gene=OGDHL
ATP2C1	ATPase Secretory Pathway Ca2+ Transporting 1	Protein Coding	51	GC03P130850	1.827577353	https://www.genecards.org/cgi-bin/carddisp.pl?gene=ATP2C1
H1-2	H1.2 Linker Histone, Cluster Member	Protein Coding	46	GC06M026056	1.826867104	https://www.genecards.org/cgi-bin/carddisp.pl?gene=H1-2
HRH2	Histamine Receptor H2	Protein Coding	50	GC05P175659	1.824629188	https://www.genecards.org/cgi-bin/carddisp.pl?gene=HRH2
PSME3	Proteasome Activator Subunit 3	Protein Coding	47	GC17P042824	1.823475361	https://www.genecards.org/cgi-bin/carddisp.pl?gene=PSME3
PTPN12	Protein Tyrosine Phosphatase Non-Receptor Type 12	Protein Coding	55	GC07P077537	1.822618484	https://www.genecards.org/cgi-bin/carddisp.pl?gene=PTPN12
RPS6KA5	Ribosomal Protein S6 Kinase A5	Protein Coding	52	GC14M090847	1.822618484	https://www.genecards.org/cgi-bin/carddisp.pl?gene=RPS6KA5

CAPN1	Calpain 1	Protein Coding	57	GC11P070846	1.822430849	https://www.genecards.org/cgi-bin/carddisp.pl?gene=CAPN1
ARHGEF1	Rho Guanine Nucleotide Exchange Factor 1	Protein Coding	53	GC19P041883	1.820183396	https://www.genecards.org/cgi-bin/carddisp.pl?gene=ARHGEF1
SLC6A11	Solute Carrier Family 6 Member 11	Protein Coding	47	GC03P013696	1.819394827	https://www.genecards.org/cgi-bin/carddisp.pl?gene=SLC6A11
MYOZ2	Myozenin 2	Protein Coding	46	GC04P119135	1.819327593	https://www.genecards.org/cgi-bin/carddisp.pl?gene=MYOZ2
HCAR3	Hydroxycarboxylic Acid Receptor 3	Protein Coding	42	GC12M122714	1.818952322	https://www.genecards.org/cgi-bin/carddisp.pl?gene=HCAR3
NISCH	Nischarin	Protein Coding	50	GC03P052455	1.817929626	https://www.genecards.org/cgi-bin/carddisp.pl?gene=NISCH
NRG4	Neuregulin 4	Protein Coding	46	GC15M075935	1.813503981	https://www.genecards.org/cgi-bin/carddisp.pl?gene=NRG4
CHRM1	Cholinergic Receptor Muscarinic 1	Protein Coding	52	GC11M099605	1.81216979	https://www.genecards.org/cgi-bin/carddisp.pl?gene=CHRM1
PTPRA	Protein Tyrosine Phosphatase Receptor Type A	Protein Coding	52	GC20P002864	1.810213327	https://www.genecards.org/cgi-bin/carddisp.pl?gene=PTPRA
MAPK12	Mitogen-Activated Protein Kinase 12	Protein Coding	54	GC22M061616	1.809207439	https://www.genecards.org/cgi-bin/carddisp.pl?gene=MAPK12
BMX	BMX Non-Receptor Tyrosine Kinase	Protein Coding	51	GC0XP015392	1.80903244	https://www.genecards.org/cgi-bin/carddisp.pl?gene=BMX
PTPN6	Protein Tyrosine Phosphatase Non-Receptor Type 6	Protein Coding	56	GC12P023562	1.804846287	https://www.genecards.org/cgi-bin/carddisp.pl?gene=PTPN6
PTPRS	Protein Tyrosine Phosphatase Receptor Type S	Protein Coding	52	GC19M006881	1.800232887	https://www.genecards.org/cgi-bin/carddisp.pl?gene=PTPRS
CDH5	Cadherin 5	Protein Coding	52	GC16P066366	1.79877305	https://www.genecards.org/cgi-bin/carddisp.pl?gene=CDH5
IL10RA	Interleukin 10 Receptor Subunit Alpha	Protein Coding	53	GC11P117987	1.796459913	https://www.genecards.org/cgi-bin/carddisp.pl?gene=IL10RA

FES	FES Proto-Oncogene, Tyrosine Kinase	Protein Coding	54	GC15P090883	1.794123411	https://www.genecards.org/cgi-bin/carddisp.pl?gene=FES
EPS15	Epidermal Growth Factor Receptor Pathway Substrate 15	Protein Coding	51	GC01M051354	1.792013168	https://www.genecards.org/cgi-bin/carddisp.pl?gene=EPS15
TPTE	Transmembrane Phosphatase With Tensin Homology	Protein Coding	43	GC21P010521	1.791667461	https://www.genecards.org/cgi-bin/carddisp.pl?gene=TPTE
MAP1B	Microtubule Associated Protein 1B	Protein Coding	51	GC05P072107	1.789745569	https://www.genecards.org/cgi-bin/carddisp.pl?gene=MAP1B
DUSP2	Dual Specificity Phosphatase 2	Protein Coding	46	GC02M098753	1.78924489	https://www.genecards.org/cgi-bin/carddisp.pl?gene=DUSP2
MCM7	Minichromosome Maintenance Complex Component 7	Protein Coding	52	GC07M100092	1.788851023	https://www.genecards.org/cgi-bin/carddisp.pl?gene=MCM7
BBS9	Bardet-Biedl Syndrome 9	Protein Coding	45	GC07P033323	1.786626101	https://www.genecards.org/cgi-bin/carddisp.pl?gene=BBS9
LCP2	Lymphocyte Cytosolic Protein 2	Protein Coding	50	GC05M170246	1.783881903	https://www.genecards.org/cgi-bin/carddisp.pl?gene=LCP2
MAP4K4	Mitogen-Activated Protein Kinase Kinase Kinase Kinase 4	Protein Coding	54	GC02P101696	1.783666372	https://www.genecards.org/cgi-bin/carddisp.pl?gene=MAP4K4
MAGI3	Membrane Associated Guanylate Kinase, WW And PDZ Domain Containing 3	Protein Coding	40	GC01P113390	1.782776356	https://www.genecards.org/cgi-bin/carddisp.pl?gene=MAGI3
RELN	Reelin	Protein Coding	51	GC07M103471	1.78237772	https://www.genecards.org/cgi-bin/carddisp.pl?gene=RELN
STAT4	Signal Transducer And Activator Of Transcription 4	Protein Coding	53	GC02M191029	1.777585745	https://www.genecards.org/cgi-bin/carddisp.pl?gene=STAT4
TTK	TTK Protein Kinase	Protein Coding	55	GC06P080003	1.777347088	https://www.genecards.org/cgi-bin/carddisp.pl?gene=TTK
PNOC	Prepronociceptin	Protein Coding	44	GC08P028316	1.774571419	https://www.genecards.org/cgi-bin/carddisp.pl?gene=PNOC
ARHGEF7	Rho Guanine Nucleotide Exchange Factor 7	Protein Coding	49	GC13P111114	1.77340126	https://www.genecards.org/cgi-bin/carddisp.pl?gene=ARHGEF7

PIP5KL1	Phosphatidylinositol-4-Phosphate 5-Kinase Like 1	Protein Coding	41	GC09M127920	1.773319483	https://www.genecards.org/cgi-bin/carddisp.pl?gene=PIP5KL1
AP2M1	Adaptor Related Protein Complex 2 Subunit Mu 1	Protein Coding	52	GC03P184174	1.772878528	https://www.genecards.org/cgi-bin/carddisp.pl?gene=AP2M1
SIGLEC7	Sialic Acid Binding Ig Like Lectin 7	Protein Coding	48	GC19P051142	1.770597219	https://www.genecards.org/cgi-bin/carddisp.pl?gene=SIGLEC7
PTGFR	Prostaglandin F Receptor	Protein Coding	52	GC01P078303	1.767858982	https://www.genecards.org/cgi-bin/carddisp.pl?gene=PTGFR
OPRK1	Opioid Receptor Kappa 1	Protein Coding	52	GC08M053227	1.767608881	https://www.genecards.org/cgi-bin/carddisp.pl?gene=OPRK1
NTF4	Neurotrophin 4	Protein Coding	53	GC19M070877	1.765598536	https://www.genecards.org/cgi-bin/carddisp.pl?gene=NTF4
CD82	CD82 Molecule	Protein Coding	50	GC11P044586	1.765353203	https://www.genecards.org/cgi-bin/carddisp.pl?gene=CD82
BYSL	Bystin Like	Protein Coding	44	GC06P092335	1.764038563	https://www.genecards.org/cgi-bin/carddisp.pl?gene=BYSL
LRPAP1	LDL Receptor Related Protein Associated Protein 1	Protein Coding	50	GC04M003508	1.763132095	https://www.genecards.org/cgi-bin/carddisp.pl?gene=LRPAP1
DUSP4	Dual Specificity Phosphatase 4	Protein Coding	48	GC08M029334	1.76159811	https://www.genecards.org/cgi-bin/carddisp.pl?gene=DUSP4
SHROOM3	Shroom Family Member 3	Protein Coding	44	GC04P076435	1.760527134	https://www.genecards.org/cgi-bin/carddisp.pl?gene=SHROOM3
TUBA4A	Tubulin Alpha 4a	Protein Coding	53	GC02M219249	1.75995791	https://www.genecards.org/cgi-bin/carddisp.pl?gene=TUBA4A
SSTR1	Somatostatin Receptor 1	Protein Coding	50	GC14P038207	1.759299755	https://www.genecards.org/cgi-bin/carddisp.pl?gene=SSTR1
NES	Nestin	Protein Coding	47	GC01M156668	1.757391214	https://www.genecards.org/cgi-bin/carddisp.pl?gene=NES
POLI	DNA Polymerase Iota	Protein Coding	48	GC18P054274	1.755850792	https://www.genecards.org/cgi-bin/carddisp.pl?gene=POLI

DDX60L	DExD/H-Box 60 Like	Protein Coding	38	GC04M168356	1.755546331	https://www.genecards.org/cgi-bin/carddisp.pl?gene=DDX60L
C5	Complement C5	Protein Coding	53	GC09M120933	1.753865361	https://www.genecards.org/cgi-bin/carddisp.pl?gene=C5
IL18R1	Interleukin 18 Receptor 1	Protein Coding	50	GC02P102311	1.750156641	https://www.genecards.org/cgi-bin/carddisp.pl?gene=IL18R1
MYOG	Myogenin	Protein Coding	46	GC01M203083	1.747856379	https://www.genecards.org/cgi-bin/carddisp.pl?gene=MYOG
SEMA7A	Semaphorin 7A (John Milton Hagen Blood Group)	Protein Coding	51	GC15M074409	1.747343063	https://www.genecards.org/cgi-bin/carddisp.pl?gene=SEMA7A
CD80	CD80 Molecule	Protein Coding	51	GC03M119524	1.745079517	https://www.genecards.org/cgi-bin/carddisp.pl?gene=CD80
DUSP6	Dual Specificity Phosphatase 6	Protein Coding	55	GC12M089347	1.744764328	https://www.genecards.org/cgi-bin/carddisp.pl?gene=DUSP6
RPL26L1	Ribosomal Protein L26 Like 1	Protein Coding	40	GC05P172956	1.743719578	https://www.genecards.org/cgi-bin/carddisp.pl?gene=RPL26L1
NMUR1	Neuromedin U Receptor 1	Protein Coding	48	GC02M231986	1.743004799	https://www.genecards.org/cgi-bin/carddisp.pl?gene=NMUR1
KAT6A	Lysine Acetyltransferase 6A	Protein Coding	52	GC08M041929	1.74228096	https://www.genecards.org/cgi-bin/carddisp.pl?gene=KAT6A
SEMA3C	Semaphorin 3C	Protein Coding	51	GC07M080742	1.742024899	https://www.genecards.org/cgi-bin/carddisp.pl?gene=SEMA3C
SIGLEC5	Sialic Acid Binding Ig Like Lectin 5	Protein Coding	47	GC19M071022	1.737704039	https://www.genecards.org/cgi-bin/carddisp.pl?gene=SIGLEC5
H2BC12L	H2B Clustered Histone 12 Like	Protein Coding	34	GC21P043569	1.736407399	https://www.genecards.org/cgi-bin/carddisp.pl?gene=H2BC12L
GRIN3A	Glutamate Ionotropic Receptor NMDA Type Subunit 3A	Protein Coding	46	GC09M101569	1.736349583	https://www.genecards.org/cgi-bin/carddisp.pl?gene=GRIN3A
CORT	Cortistatin	Protein Coding	41	GC01P010529	1.734572053	https://www.genecards.org/cgi-bin/carddisp.pl?gene=CORT

RGS4	Regulator Of G Protein Signaling 4	Protein Coding	48	GC01P163038	1.734306216	https://www.genecards.org/cgi-bin/carddisp.pl?gene=RGS4
HAPLN1	Hyaluronan And Proteoglycan Link Protein 1	Protein Coding	48	GC05M083637	1.732725978	https://www.genecards.org/cgi-bin/carddisp.pl?gene=HAPLN1
SCG2	Secretogranin II	Protein Coding	46	GC02M223596	1.731810331	https://www.genecards.org/cgi-bin/carddisp.pl?gene=SCG2
GIT1	GIT ArfGAP 1	Protein Coding	49	GC17M029573	1.728217602	https://www.genecards.org/cgi-bin/carddisp.pl?gene=GIT1
RAB9A	RAB9A, Member RAS Oncogene Family	Protein Coding	45	GC0XP013707	1.727880001	https://www.genecards.org/cgi-bin/carddisp.pl?gene=RAB9A
TRIM27	Tripartite Motif Containing 27	Protein Coding	48	GC06M028903	1.726555586	https://www.genecards.org/cgi-bin/carddisp.pl?gene=TRIM27
PGAM4	Phosphoglycerate Mutase Family Member 4	Protein Coding	41	GC0XM077967	1.725485802	https://www.genecards.org/cgi-bin/carddisp.pl?gene=PGAM4
IFNAR1	Interferon Alpha And Beta Receptor Subunit 1	Protein Coding	55	GC21P033324	1.725248218	https://www.genecards.org/cgi-bin/carddisp.pl?gene=IFNAR1
HOXA10	Homeobox A10	Protein Coding	47	GC07M027673	1.723849893	https://www.genecards.org/cgi-bin/carddisp.pl?gene=HOXA10
MAP2K6	Mitogen-Activated Protein Kinase Kinase 6	Protein Coding	53	GC17P069414	1.720388412	https://www.genecards.org/cgi-bin/carddisp.pl?gene=MAP2K6
PENK	Proenkephalin	Protein Coding	45	GC08M056436	1.717689514	https://www.genecards.org/cgi-bin/carddisp.pl?gene=PENK
SNTA1	Syntrophin Alpha 1	Protein Coding	52	GC20M033407	1.717225552	https://www.genecards.org/cgi-bin/carddisp.pl?gene=SNTA1
IRF8	Interferon Regulatory Factor 8	Protein Coding	52	GC16P085898	1.716549635	https://www.genecards.org/cgi-bin/carddisp.pl?gene=IRF8
PTPDC1	Protein Tyrosine Phosphatase Domain Containing 1	Protein Coding	39	GC09P094030	1.710317612	https://www.genecards.org/cgi-bin/carddisp.pl?gene=PTPDC1
ZDHHC13	Zinc Finger DHHC-Type Palmitoyltransferase 13	Protein Coding	44	GC11P019095	1.710026741	https://www.genecards.org/cgi-bin/carddisp.pl?gene=ZDHHC13

SSTR4	Somatostatin Receptor 4	Protein Coding	47	GC20P023035	1.70855999	https://www.genecards.org/cgi-bin/carddisp.pl?gene=SSTR4
TLK2	Tousled Like Kinase 2	Protein Coding	52	GC17P062458	1.706749916	https://www.genecards.org/cgi-bin/carddisp.pl?gene=TLK2
MPIG6B	Megakaryocyte And Platelet Inhibitory Receptor G6b	Protein Coding	44	GC06P094327	1.706351757	https://www.genecards.org/cgi-bin/carddisp.pl?gene=MPIG6B
LIFR	LIF Receptor Subunit Alpha	Protein Coding	53	GC05M038475	1.704889536	https://www.genecards.org/cgi-bin/carddisp.pl?gene=LIFR
ZDHHC2	Zinc Finger DHHC-Type Palmitoyltransferase 2	Protein Coding	44	GC08P017156	1.704699993	https://www.genecards.org/cgi-bin/carddisp.pl?gene=ZDHHC2
ZFP36	ZFP36 Ring Finger Protein	Protein Coding	44	GC19P039406	1.703535914	https://www.genecards.org/cgi-bin/carddisp.pl?gene=ZFP36
IL32	Interleukin 32	Protein Coding	45	GC16P013549	1.702359438	https://www.genecards.org/cgi-bin/carddisp.pl?gene=IL32
FPR2	Formyl Peptide Receptor 2	Protein Coding	53	GC19P051752	1.70159018	https://www.genecards.org/cgi-bin/carddisp.pl?gene=FPR2
SNCG	Synuclein Gamma	Protein Coding	48	GC10P094523	1.700464964	https://www.genecards.org/cgi-bin/carddisp.pl?gene=SNCG
LOC102724560	Cystathionine Beta-Synthase Like	Protein Coding	10	GC21M006444	1.700024605	https://www.genecards.org/cgi-bin/carddisp.pl?gene=LOC102724560
DSG2	Desmoglein 2	Protein Coding	52	GC18P031498	1.697177649	https://www.genecards.org/cgi-bin/carddisp.pl?gene=DSG2
PDLIM5	PDZ And LIM Domain 5	Protein Coding	47	GC04P094451	1.693854332	https://www.genecards.org/cgi-bin/carddisp.pl?gene=PDLIM5
OPRL1	Opioid Related Nociceptin Receptor 1	Protein Coding	53	GC20P064080	1.692744732	https://www.genecards.org/cgi-bin/carddisp.pl?gene=OPRL1
SLC7A10	Solute Carrier Family 7 Member 10	Protein Coding	44	GC19M033208	1.691041231	https://www.genecards.org/cgi-bin/carddisp.pl?gene=SLC7A10
EPHB2	EPH Receptor B2	Protein Coding	59	GC01P022710	1.688824892	https://www.genecards.org/cgi-bin/carddisp.pl?gene=EPHB2

PITX2	Paired Like Homeodomain 2	Protein Coding	52	GC04M110617	1.687981844	https://www.genecards.org/cgi-bin/carddisp.pl?gene=PITX2
IL21	Interleukin 21	Protein Coding	51	GC04M122612	1.68727994	https://www.genecards.org/cgi-bin/carddisp.pl?gene=IL21
ZDHHC18	Zinc Finger DHHC-Type Palmitoyltransferase 18	Protein Coding	42	GC01P028385	1.686555862	https://www.genecards.org/cgi-bin/carddisp.pl?gene=ZDHHC18
DUSP3	Dual Specificity Phosphatase 3	Protein Coding	51	GC17M043766	1.68346417	https://www.genecards.org/cgi-bin/carddisp.pl?gene=DUSP3
VIPR1	Vasoactive Intestinal Peptide Receptor 1	Protein Coding	52	GC03P042490	1.677259088	https://www.genecards.org/cgi-bin/carddisp.pl?gene=VIPR1
RBL2	RB Transcriptional Corepressor Like 2	Protein Coding	52	GC16P053433	1.67690587	https://www.genecards.org/cgi-bin/carddisp.pl?gene=RBL2
OMD	Osteomodulin	Protein Coding	46	GC09M096534	1.674594641	https://www.genecards.org/cgi-bin/carddisp.pl?gene=OMD
EEA1	Early Endosome Antigen 1	Protein Coding	48	GC12M092770	1.672343969	https://www.genecards.org/cgi-bin/carddisp.pl?gene=EEA1
LYVE1	Lymphatic Vessel Endothelial Hyaluronan Receptor 1	Protein Coding	49	GC11M011131	1.659945846	https://www.genecards.org/cgi-bin/carddisp.pl?gene=LYVE1
CD2	CD2 Molecule	Protein Coding	51	GC01P116754	1.659570932	https://www.genecards.org/cgi-bin/carddisp.pl?gene=CD2
GAB2	GRB2 Associated Binding Protein 2	Protein Coding	50	GC11M078215	1.656756639	https://www.genecards.org/cgi-bin/carddisp.pl?gene=GAB2
SRP19	Signal Recognition Particle 19	Protein Coding	46	GC05P112862	1.65417254	https://www.genecards.org/cgi-bin/carddisp.pl?gene=SRP19
PTPRO	Protein Tyrosine Phosphatase Receptor Type O	Protein Coding	54	GC12P023827	1.647766829	https://www.genecards.org/cgi-bin/carddisp.pl?gene=PTPRO
LIPI	Lipase I	Protein Coding	42	GC21M014108	1.644866705	https://www.genecards.org/cgi-bin/carddisp.pl?gene=LIPI
PTGIR	Prostaglandin I2 Receptor	Protein Coding	54	GC19M070781	1.644169569	https://www.genecards.org/cgi-bin/carddisp.pl?gene=PTGIR

IL13RA1	Interleukin 13 Receptor Subunit Alpha 1	Protein Coding	48	GC0XP118727	1.643259287	https://www.genecards.org/cgi-bin/carddisp.pl?gene=IL13RA1
NRTN	Neurturin	Protein Coding	47	GC19P005805	1.643030405	https://www.genecards.org/cgi-bin/carddisp.pl?gene=NRTN
PTGDR2	Prostaglandin D2 Receptor 2	Protein Coding	51	GC11M060850	1.642745137	https://www.genecards.org/cgi-bin/carddisp.pl?gene=PTGDR2
TGOLN2	Trans-Golgi Network Protein 2	Protein Coding	44	GC02M085318	1.642578602	https://www.genecards.org/cgi-bin/carddisp.pl?gene=TGOLN2
EPHA7	EPH Receptor A7	Protein Coding	54	GC06M093240	1.64180851	https://www.genecards.org/cgi-bin/carddisp.pl?gene=EPHA7
LTK	Leukocyte Receptor Tyrosine Kinase	Protein Coding	49	GC15M041503	1.64180851	https://www.genecards.org/cgi-bin/carddisp.pl?gene=LTK
CCNT1	Cyclin T1	Protein Coding	47	GC12M048688	1.640436649	https://www.genecards.org/cgi-bin/carddisp.pl?gene=CCNT1
GRM3	Glutamate Metabotropic Receptor 3	Protein Coding	53	GC07P086643	1.639108658	https://www.genecards.org/cgi-bin/carddisp.pl?gene=GRM3
ICA1	Islet Cell Autoantigen 1	Protein Coding	47	GC07M008428	1.638249993	https://www.genecards.org/cgi-bin/carddisp.pl?gene=ICA1
OSMR	Oncostatin M Receptor	Protein Coding	52	GC05P038845	1.636943102	https://www.genecards.org/cgi-bin/carddisp.pl?gene=OSMR
ICAM3	Intercellular Adhesion Molecule 3	Protein Coding	50	GC19M010421	1.635703325	https://www.genecards.org/cgi-bin/carddisp.pl?gene=ICAM3
SFRP1	Secreted Frizzled Related Protein 1	Protein Coding	51	GC08M041262	1.635003924	https://www.genecards.org/cgi-bin/carddisp.pl?gene=SFRP1
KCNJ4	Potassium Inwardly Rectifying Channel Subfamily J Member 4	Protein Coding	50	GC22M038426	1.631995916	https://www.genecards.org/cgi-bin/carddisp.pl?gene=KCNJ4
LIMK2	LIM Domain Kinase 2	Protein Coding	52	GC22P031212	1.629135966	https://www.genecards.org/cgi-bin/carddisp.pl?gene=LIMK2
HAND2	Heart And Neural Crest Derivatives Expressed 2	Protein Coding	50	GC04M173524	1.628247738	https://www.genecards.org/cgi-bin/carddisp.pl?gene=HAND2

CDC37	Cell Division Cycle 37, HSP90 Cochaperone	Protein Coding	50	GC19M010391	1.62809515	https://www.genecards.org/cgi-bin/carddisp.pl?gene=CDC37
HOXA9	Homeobox A9	Protein Coding	45	GC07M027162	1.626486778	https://www.genecards.org/cgi-bin/carddisp.pl?gene=HOXA9
CAPN2	Calpain 2	Protein Coding	54	GC01P223701	1.625161171	https://www.genecards.org/cgi-bin/carddisp.pl?gene=CAPN2
BRD8	Bromodomain Containing 8	Protein Coding	45	GC05M138152	1.625022411	https://www.genecards.org/cgi-bin/carddisp.pl?gene=BRD8
ASCL1	Achaete-Scute Family BHLH Transcription Factor 1	Protein Coding	50	GC12P102957	1.621473312	https://www.genecards.org/cgi-bin/carddisp.pl?gene=ASCL1
EFNB1	Ephrin B1	Protein Coding	52	GC0XP068828	1.620697021	https://www.genecards.org/cgi-bin/carddisp.pl?gene=EFNB1
POU2AF1	POU Class 2 Homeobox Associating Factor 1	Protein Coding	44	GC11M111352	1.620057344	https://www.genecards.org/cgi-bin/carddisp.pl?gene=POU2AF1
HMGN2	High Mobility Group Nucleosomal Binding Domain 2	Protein Coding	46	GC01P026473	1.617171049	https://www.genecards.org/cgi-bin/carddisp.pl?gene=HMGN2
LGALS13	Galectin 13	Protein Coding	43	GC19P039602	1.616792679	https://www.genecards.org/cgi-bin/carddisp.pl?gene=LGALS13
SULF1	Sulfatase 1	Protein Coding	49	GC08P069466	1.616624832	https://www.genecards.org/cgi-bin/carddisp.pl?gene=SULF1
FGF4	Fibroblast Growth Factor 4	Protein Coding	50	GC11M099886	1.614102602	https://www.genecards.org/cgi-bin/carddisp.pl?gene=FGF4
AKAP1	A-Kinase Anchoring Protein 1	Protein Coding	47	GC17P057085	1.612334251	https://www.genecards.org/cgi-bin/carddisp.pl?gene=AKAP1
RYK	Receptor Like Tyrosine Kinase	Protein Coding	50	GC03M134065	1.611094236	https://www.genecards.org/cgi-bin/carddisp.pl?gene=RYK
CCNB2	Cyclin B2	Protein Coding	51	GC15P059105	1.610750675	https://www.genecards.org/cgi-bin/carddisp.pl?gene=CCNB2
TPPP3	Tubulin Polymerization Promoting Protein Family Member 3	Protein Coding	43	GC16M067389	1.609044194	https://www.genecards.org/cgi-bin/carddisp.pl?gene=TPPP3

CASP14	Caspase 14	Protein Coding	53	GC19P015049	1.608549476	https://www.genecards.org/cgi-bin/carddisp.pl?gene=CASP14
PTPRB	Protein Tyrosine Phosphatase Receptor Type B	Protein Coding	52	GC12M070516	1.608270049	https://www.genecards.org/cgi-bin/carddisp.pl?gene=PTPRB
KCND3	Potassium Voltage-Gated Channel Subfamily D Member 3	Protein Coding	53	GC01M111770	1.607928157	https://www.genecards.org/cgi-bin/carddisp.pl?gene=KCND3
TOM1	Target Of Myb1 Membrane Trafficking Protein	Protein Coding	49	GC22P035299	1.60710144	https://www.genecards.org/cgi-bin/carddisp.pl?gene=TOM1
C2CD5	C2 Calcium Dependent Domain Containing 5	Protein Coding	40	GC12M022448	1.606956601	https://www.genecards.org/cgi-bin/carddisp.pl?gene=C2CD5
TAC3	Tachykinin Precursor 3	Protein Coding	50	GC12M057108	1.605289936	https://www.genecards.org/cgi-bin/carddisp.pl?gene=TAC3
ERG	ETS Transcription Factor ERG	Protein Coding	53	GC21M038367	1.603666306	https://www.genecards.org/cgi-bin/carddisp.pl?gene=ERG
EN2	Engrailed Homeobox 2	Protein Coding	46	GC07P155459	1.602025986	https://www.genecards.org/cgi-bin/carddisp.pl?gene=EN2
SAG	S-Antigen Visual Arrestin	Protein Coding	51	GC02P233400	1.600292444	https://www.genecards.org/cgi-bin/carddisp.pl?gene=SAG
KIR3DL1	Killer Cell Immunoglobulin Like Receptor, Three Ig Domains And Long Cytoplasmic Tail 1	Protein Coding	46	GC19P073503	1.599633932	https://www.genecards.org/cgi-bin/carddisp.pl?gene=KIR3DL1
CHRNA4	Cholinergic Receptor Nicotinic Alpha 4 Subunit	Protein Coding	56	GC20M063343	1.597695589	https://www.genecards.org/cgi-bin/carddisp.pl?gene=CHRNA4
CCL26	C-C Motif Chemokine Ligand 26	Protein Coding	44	GC07M075769	1.596167326	https://www.genecards.org/cgi-bin/carddisp.pl?gene=CCL26
RNF41	Ring Finger Protein 41	Protein Coding	46	GC12M056202	1.593486309	https://www.genecards.org/cgi-bin/carddisp.pl?gene=RNF41
TLR5	Toll Like Receptor 5	Protein Coding	53	GC01M223109	1.591864109	https://www.genecards.org/cgi-bin/carddisp.pl?gene=TLR5
PI3	Peptidase Inhibitor 3	Protein Coding	44	GC20P045174	1.590837479	https://www.genecards.org/cgi-bin/carddisp.pl?gene=PI3

CD209	CD209 Molecule	Protein Coding	50	GC19M007739	1.590370893	https://www.genecards.org/cgi-bin/carddisp.pl?gene=CD209
MVP	Major Vault Protein	Protein Coding	47	GC16P042738	1.587863684	https://www.genecards.org/cgi-bin/carddisp.pl?gene=MVP
REG1A	Regenerating Family Member 1 Alpha	Protein Coding	46	GC02P079120	1.587365627	https://www.genecards.org/cgi-bin/carddisp.pl?gene=REG1A
TDP1	Tyrosyl-DNA Phosphodiesterase 1	Protein Coding	51	GC14P089954	1.585007429	https://www.genecards.org/cgi-bin/carddisp.pl?gene=TDP1
CHRNA1	Cholinergic Receptor Nicotinic Alpha 1 Subunit	Protein Coding	52	GC02M174747	1.583951354	https://www.genecards.org/cgi-bin/carddisp.pl?gene=CHRNA1
ANP32A	Acidic Nuclear Phosphoprotein 32 Family Member A	Protein Coding	50	GC15M068778	1.581532955	https://www.genecards.org/cgi-bin/carddisp.pl?gene=ANP32A
GAP43	Growth Associated Protein 43	Protein Coding	50	GC03P115623	1.578776121	https://www.genecards.org/cgi-bin/carddisp.pl?gene=GAP43
RBM14	RNA Binding Motif Protein 14	Protein Coding	45	GC11P070979	1.578552961	https://www.genecards.org/cgi-bin/carddisp.pl?gene=RBM14
CSTA	Cystatin A	Protein Coding	51	GC03P122325	1.577850103	https://www.genecards.org/cgi-bin/carddisp.pl?gene=CSTA
LMTK3	Lemur Tyrosine Kinase 3	Protein Coding	42	GC19M048485	1.575712204	https://www.genecards.org/cgi-bin/carddisp.pl?gene=LMTK3
SH3KBP1	SH3 Domain Containing Kinase Binding Protein 1	Protein Coding	48	GC0XM019552	1.574989557	https://www.genecards.org/cgi-bin/carddisp.pl?gene=SH3KBP1
SLIT3	Slit Guidance Ligand 3	Protein Coding	48	GC05M168661	1.573313236	https://www.genecards.org/cgi-bin/carddisp.pl?gene=SLIT3
WAS	WASP Actin Nucleation Promoting Factor	Protein Coding	55	GC0XP048676	1.572755575	https://www.genecards.org/cgi-bin/carddisp.pl?gene=WAS
FCGR2B	Fc Gamma Receptor IIb	Protein Coding	56	GC01P161823	1.570428848	https://www.genecards.org/cgi-bin/carddisp.pl?gene=FCGR2B
PTPRU	Protein Tyrosine Phosphatase Receptor Type U	Protein Coding	50	GC01P029236	1.567531228	https://www.genecards.org/cgi-bin/carddisp.pl?gene=PTPRU

ITSN1	Intersectin 1	Protein Coding	48	GC21P033642	1.564399242	https://www.genecards.org/cgi-bin/carddisp.pl?gene=ITSN1
ABI2	Abl Interactor 2	Protein Coding	45	GC02P203327	1.561905265	https://www.genecards.org/cgi-bin/carddisp.pl?gene=ABI2
VAV1	Vav Guanine Nucleotide Exchange Factor 1	Protein Coding	52	GC19P006772	1.559613466	https://www.genecards.org/cgi-bin/carddisp.pl?gene=VAV1
BCL3	BCL3 Transcription Coactivator	Protein Coding	48	GC19P044747	1.558552265	https://www.genecards.org/cgi-bin/carddisp.pl?gene=BCL3
STATH	Statherin	Protein Coding	38	GC04P069995	1.558440924	https://www.genecards.org/cgi-bin/carddisp.pl?gene=STATH
TOLLIP	Toll Interacting Protein	Protein Coding	51	GC11M001274	1.555968523	https://www.genecards.org/cgi-bin/carddisp.pl?gene=TOLLIP
IL22RA2	Interleukin 22 Receptor Subunit Alpha 2	Protein Coding	47	GC06M137143	1.553938866	https://www.genecards.org/cgi-bin/carddisp.pl?gene=IL22RA2
THBS4	Thrombospondin 4	Protein Coding	50	GC05P079991	1.549686193	https://www.genecards.org/cgi-bin/carddisp.pl?gene=THBS4
TRPA1	Transient Receptor Potential Cation Channel Subfamily A Member 1	Protein Coding	53	GC08M072019	1.548540115	https://www.genecards.org/cgi-bin/carddisp.pl?gene=TRPA1
S100A10	S100 Calcium Binding Protein A10	Protein Coding	50	GC01M152817	1.548104286	https://www.genecards.org/cgi-bin/carddisp.pl?gene=S100A10
ABL2	ABL Proto-Oncogene 2, Non-Receptor Tyrosine Kinase	Protein Coding	52	GC01M179143	1.548074484	https://www.genecards.org/cgi-bin/carddisp.pl?gene=ABL2
PIGF	Phosphatidylinositol Glycan Anchor Biosynthesis Class F	Protein Coding	46	GC02M046580	1.547854424	https://www.genecards.org/cgi-bin/carddisp.pl?gene=PIGF
EGR2	Early Growth Response 2	Protein Coding	51	GC10M062811	1.542626143	https://www.genecards.org/cgi-bin/carddisp.pl?gene=EGR2
ZKSCAN7	Zinc Finger With KRAB And SCAN Domains 7	Protein Coding	39	GC03P044556	1.539433479	https://www.genecards.org/cgi-bin/carddisp.pl?gene=ZKSCAN7
CHRND	Cholinergic Receptor Nicotinic Delta Subunit	Protein Coding	50	GC02P232525	1.539049506	https://www.genecards.org/cgi-bin/carddisp.pl?gene=CHRND

ELP3	Elongator Acetyltransferase Complex Subunit 3	Protein Coding	48	GC08P028089	1.538787603	https://www.genecards.org/cgi-bin/carddisp.pl?gene=ELP3
FCER1G	Fc Epsilon Receptor Ig	Protein Coding	48	GC01P161215	1.535698891	https://www.genecards.org/cgi-bin/carddisp.pl?gene=FCER1G
SHB	SH2 Domain Containing Adaptor Protein B	Protein Coding	46	GC09M040554	1.535517454	https://www.genecards.org/cgi-bin/carddisp.pl?gene=SHB
MSBP2	Minisatellite Binding Protein 2	Protein Coding	5	GC00U990214	1.533616304	https://www.genecards.org/cgi-bin/carddisp.pl?gene=MSBP2
WNT11	Wnt Family Member 11	Protein Coding	50	GC11M076186	1.532100201	https://www.genecards.org/cgi-bin/carddisp.pl?gene=WNT11
TBXT	T-Box Transcription Factor T	Protein Coding	50	GC06M166158	1.531953096	https://www.genecards.org/cgi-bin/carddisp.pl?gene=TBXT
MAP3K2	Mitogen-Activated Protein Kinase Kinase Kinase 2	Protein Coding	52	GC02M127298	1.531584501	https://www.genecards.org/cgi-bin/carddisp.pl?gene=MAP3K2
FGFRL1	Fibroblast Growth Factor Receptor Like 1	Protein Coding	50	GC04P001370	1.53033185	https://www.genecards.org/cgi-bin/carddisp.pl?gene=FGFRL1
CLEC12A	C-Type Lectin Domain Family 12 Member A	Protein Coding	46	GC12P009951	1.528077364	https://www.genecards.org/cgi-bin/carddisp.pl?gene=CLEC12A
PTPN9	Protein Tyrosine Phosphatase Non-Receptor Type 9	Protein Coding	46	GC15M075463	1.525749922	https://www.genecards.org/cgi-bin/carddisp.pl?gene=PTPN9
BAIAP2	BAR/IMD Domain Containing Adaptor Protein 2	Protein Coding	48	GC17P081035	1.525718451	https://www.genecards.org/cgi-bin/carddisp.pl?gene=BAIAP2
CA11	Carbonic Anhydrase 11	Protein Coding	44	GC19M070852	1.523382664	https://www.genecards.org/cgi-bin/carddisp.pl?gene=CA11
JPH4	Junctophilin 4	Protein Coding	41	GC14M023568	1.521375775	https://www.genecards.org/cgi-bin/carddisp.pl?gene=JPH4
GCAT	Glycine C-Acetyltransferase	Protein Coding	47	GC22P037807	1.521194458	https://www.genecards.org/cgi-bin/carddisp.pl?gene=GCAT
PMP22	Peripheral Myelin Protein 22	Protein Coding	50	GC17M015229	1.517963052	https://www.genecards.org/cgi-bin/carddisp.pl?gene=PMP22

MUC4	Mucin 4, Cell Surface Associated	Protein Coding	47	GC03M195746	1.517698765	https://www.genecards.org/cgi-bin/carddisp.pl?gene=MUC4
GAB1	GRB2 Associated Binding Protein 1	Protein Coding	51	GC04P143336	1.514995813	https://www.genecards.org/cgi-bin/carddisp.pl?gene=GAB1
FRS2	Fibroblast Growth Factor Receptor Substrate 2	Protein Coding	46	GC12P069471	1.51312685	https://www.genecards.org/cgi-bin/carddisp.pl?gene=FRS2
SV2A	Synaptic Vesicle Glycoprotein 2A	Protein Coding	50	GC01M149903	1.510024309	https://www.genecards.org/cgi-bin/carddisp.pl?gene=SV2A
MATK	Megakaryocyte-Associated Tyrosine Kinase	Protein Coding	51	GC19M003777	1.509203672	https://www.genecards.org/cgi-bin/carddisp.pl?gene=MATK
ABI1	Abl Interactor 1	Protein Coding	50	GC10M026746	1.507948875	https://www.genecards.org/cgi-bin/carddisp.pl?gene=ABI1
SCYL1	SCY1 Like Pseudokinase 1	Protein Coding	49	GC11P065525	1.507302999	https://www.genecards.org/cgi-bin/carddisp.pl?gene=SCYL1
CD22	CD22 Molecule	Protein Coding	53	GC19P035319	1.507148862	https://www.genecards.org/cgi-bin/carddisp.pl?gene=CD22
SNX9	Sorting Nexin 9	Protein Coding	47	GC06P157702	1.507115602	https://www.genecards.org/cgi-bin/carddisp.pl?gene=SNX9
ZYX	Zyxin	Protein Coding	52	GC07P143381	1.506281376	https://www.genecards.org/cgi-bin/carddisp.pl?gene=ZYX
DUSP13	Dual Specificity Phosphatase 13	Protein Coding	45	GC10M075094	1.505273819	https://www.genecards.org/cgi-bin/carddisp.pl?gene=DUSP13
PDPN	Podoplanin	Protein Coding	48	GC01P013583	1.504307032	https://www.genecards.org/cgi-bin/carddisp.pl?gene=PDPN
FKBP5	FKBP Prolyl Isomerase 5	Protein Coding	56	GC06M071862	1.503385782	https://www.genecards.org/cgi-bin/carddisp.pl?gene=FKBP5
HECTD2	HECT Domain E3 Ubiquitin Protein Ligase 2	Protein Coding	42	GC10P091409	1.502227306	https://www.genecards.org/cgi-bin/carddisp.pl?gene=HECTD2
SPN	Sialophorin	Protein Coding	47	GC16P029662	1.501608849	https://www.genecards.org/cgi-bin/carddisp.pl?gene=SPN

TNR	Tenascin R	Protein Coding	50	GC01M175291	1.499696016	https://www.genecards.org/cgi-bin/carddisp.pl?gene=TNR
KTI12	KTI12 Chromatin Associated Homolog	Protein Coding	36	GC01M052032	1.497702956	https://www.genecards.org/cgi-bin/carddisp.pl?gene=KTI12
MED30	Mediator Complex Subunit 30	Protein Coding	43	GC08P117521	1.497054815	https://www.genecards.org/cgi-bin/carddisp.pl?gene=MED30
RNASE1	Ribonuclease A Family Member 1, Pancreatic	Protein Coding	48	GC14M020801	1.49683392	https://www.genecards.org/cgi-bin/carddisp.pl?gene=RNASE1
P2RX3	Purinergic Receptor P2X 3	Protein Coding	50	GC11P057356	1.494965792	https://www.genecards.org/cgi-bin/carddisp.pl?gene=P2RX3
FYB1	FYN Binding Protein 1	Protein Coding	50	GC05M039105	1.491333246	https://www.genecards.org/cgi-bin/carddisp.pl?gene=FYB1
TGFB1I1	Transforming Growth Factor Beta 1 Induced Transcript 1	Protein Coding	48	GC16P042867	1.490437984	https://www.genecards.org/cgi-bin/carddisp.pl?gene=TGFB1I1
ACKR1	Atypical Chemokine Receptor 1 (Duffy Blood Group)	Protein Coding	48	GC01P159203	1.488973379	https://www.genecards.org/cgi-bin/carddisp.pl?gene=ACKR1
PTK7	Protein Tyrosine Kinase 7 (Inactive)	Protein Coding	52	GC06P043076	1.488434076	https://www.genecards.org/cgi-bin/carddisp.pl?gene=PTK7
CHRNB2	Cholinergic Receptor Nicotinic Beta 2 Subunit	Protein Coding	53	GC01P154568	1.48781085	https://www.genecards.org/cgi-bin/carddisp.pl?gene=CHRNB2
CTSW	Cathepsin W	Protein Coding	43	GC11P065879	1.486487627	https://www.genecards.org/cgi-bin/carddisp.pl?gene=CTSW
CCR4	C-C Motif Chemokine Receptor 4	Protein Coding	51	GC03P032951	1.486412525	https://www.genecards.org/cgi-bin/carddisp.pl?gene=CCR4
TRIP6	Thyroid Hormone Receptor Interactor 6	Protein Coding	46	GC07P100867	1.486283898	https://www.genecards.org/cgi-bin/carddisp.pl?gene=TRIP6
MPP1	MAGUK P55 Scaffold Protein 1	Protein Coding	46	GC0XM154779	1.485907793	https://www.genecards.org/cgi-bin/carddisp.pl?gene=MPP1
GLRA1	Glycine Receptor Alpha 1	Protein Coding	53	GC05M151822	1.483913183	https://www.genecards.org/cgi-bin/carddisp.pl?gene=GLRA1

EIF4H	Eukaryotic Translation Initiation Factor 4H	Protein Coding	48	GC07P074174	1.483606577	https://www.genecards.org/cgi-bin/carddisp.pl?gene=EIF4H
HDAC8	Histone Deacetylase 8	Protein Coding	55	GC0XM072329	1.483047009	https://www.genecards.org/cgi-bin/carddisp.pl?gene=HDAC8
MAP4K2	Mitogen-Activated Protein Kinase Kinase Kinase Kinase 2	Protein Coding	53	GC11M099661	1.471613169	https://www.genecards.org/cgi-bin/carddisp.pl?gene=MAP4K2
MRPL28	Mitochondrial Ribosomal Protein L28	Protein Coding	43	GC16M000366	1.47159481	https://www.genecards.org/cgi-bin/carddisp.pl?gene=MRPL28
AQP5	Aquaporin 5	Protein Coding	53	GC12P049961	1.471539617	https://www.genecards.org/cgi-bin/carddisp.pl?gene=AQP5
PPP2R3A	Protein Phosphatase 2 Regulatory Subunit B"Alpha	Protein Coding	43	GC03P135965	1.46496439	https://www.genecards.org/cgi-bin/carddisp.pl?gene=PPP2R3A
TRIM22	Tripartite Motif Containing 22	Protein Coding	46	GC11P005689	1.461960793	https://www.genecards.org/cgi-bin/carddisp.pl?gene=TRIM22
SIT1	Signaling Threshold Regulating Transmembrane Adaptor 1	Protein Coding	43	GC09M035640	1.460095882	https://www.genecards.org/cgi-bin/carddisp.pl?gene=SIT1
PDIA2	Protein Disulfide Isomerase Family A Member 2	Protein Coding	48	GC16P013436	1.459900379	https://www.genecards.org/cgi-bin/carddisp.pl?gene=PDIA2
ETV1	ETS Variant Transcription Factor 1	Protein Coding	51	GC07M013891	1.45987165	https://www.genecards.org/cgi-bin/carddisp.pl?gene=ETV1
VEGFD	Vascular Endothelial Growth Factor D	Protein Coding	47	GC0XM015345	1.458869696	https://www.genecards.org/cgi-bin/carddisp.pl?gene=VEGFD
LRIG1	Leucine Rich Repeats And Immunoglobulin Like Domains 1	Protein Coding	49	GC03M066379	1.458433747	https://www.genecards.org/cgi-bin/carddisp.pl?gene=LRIG1
ACTR3	Actin Related Protein 3	Protein Coding	47	GC02P113889	1.458372474	https://www.genecards.org/cgi-bin/carddisp.pl?gene=ACTR3
PTPRK	Protein Tyrosine Phosphatase Receptor Type K	Protein Coding	51	GC06M127949	1.45528698	https://www.genecards.org/cgi-bin/carddisp.pl?gene=PTPRK
DCTN2	Dynactin Subunit 2	Protein Coding	47	GC12M057771	1.454296112	https://www.genecards.org/cgi-bin/carddisp.pl?gene=DCTN2

TDGF1	Teratocarcinoma-Derived Growth Factor 1	Protein Coding	49	GC03P047633	1.454260468	https://www.genecards.org/cgi-bin/carddisp.pl?gene=TDGF1
TEC	Tec Protein Tyrosine Kinase	Protein Coding	52	GC04M048177	1.453444481	https://www.genecards.org/cgi-bin/carddisp.pl?gene=TEC
EIF3B	Eukaryotic Translation Initiation Factor 3 Subunit B	Protein Coding	45	GC07P002354	1.452422142	https://www.genecards.org/cgi-bin/carddisp.pl?gene=EIF3B
NRGN	Neurogranin	Protein Coding	44	GC11P124739	1.450027466	https://www.genecards.org/cgi-bin/carddisp.pl?gene=NRGN
FIP1L1	Factor Interacting With PAPOLA And CPSF1	Protein Coding	47	GC04P053435	1.449950576	https://www.genecards.org/cgi-bin/carddisp.pl?gene=FIP1L1
EFNA3	Ephrin A3	Protein Coding	51	GC01P155078	1.446974158	https://www.genecards.org/cgi-bin/carddisp.pl?gene=EFNA3
GLI2	GLI Family Zinc Finger 2	Protein Coding	56	GC02P120735	1.444097519	https://www.genecards.org/cgi-bin/carddisp.pl?gene=GLI2
CIT	Citron Rho-Interacting Serine/Threonine Kinase	Protein Coding	54	GC12M119687	1.443084955	https://www.genecards.org/cgi-bin/carddisp.pl?gene=CIT
KIFAP3	Kinesin Associated Protein 3	Protein Coding	46	GC01M169921	1.442029834	https://www.genecards.org/cgi-bin/carddisp.pl?gene=KIFAP3
GJB2	Gap Junction Protein Beta 2	Protein Coding	52	GC13M020187	1.441787243	https://www.genecards.org/cgi-bin/carddisp.pl?gene=GJB2
KAT7	Lysine Acetyltransferase 7	Protein Coding	49	GC17P049788	1.440423012	https://www.genecards.org/cgi-bin/carddisp.pl?gene=KAT7
DOK1	Docking Protein 1	Protein Coding	50	GC02P074549	1.440416574	https://www.genecards.org/cgi-bin/carddisp.pl?gene=DOK1
MIR26A2	MicroRNA 26a-2	RNA Gene	22	GC12M057824	1.43974936	https://www.genecards.org/cgi-bin/carddisp.pl?gene=MIR26A2
RDX	Radixin	Protein Coding	53	GC11M109864	1.43893826	https://www.genecards.org/cgi-bin/carddisp.pl?gene=RDX
CLUH	Clustered Mitochondria Homolog	Protein Coding	43	GC17M002689	1.43893826	https://www.genecards.org/cgi-bin/carddisp.pl?gene=CLUH

PTPRG	Protein Tyrosine Phosphatase Receptor Type G	Protein Coding	51	GC03P061561	1.437517405	https://www.genecards.org/cgi-bin/carddisp.pl?gene=PTPRG
TRMT6	TRNA Methyltransferase 6 Non-Catalytic Subunit	Protein Coding	40	GC20M005937	1.436141014	https://www.genecards.org/cgi-bin/carddisp.pl?gene=TRMT6
FOSB	FosB Proto-Oncogene, AP-1 Transcription Factor Subunit	Protein Coding	49	GC19P045467	1.435516	https://www.genecards.org/cgi-bin/carddisp.pl?gene=FOSB
EFNA2	Ephrin A2	Protein Coding	48	GC19P003719	1.432334781	https://www.genecards.org/cgi-bin/carddisp.pl?gene=EFNA2
HEMK1	HemK Methyltransferase Family Member 1	Protein Coding	42	GC03P050569	1.430846691	https://www.genecards.org/cgi-bin/carddisp.pl?gene=HEMK1
CLCF1	Cardiotrophin Like Cytokine Factor 1	Protein Coding	49	GC11M067364	1.427292824	https://www.genecards.org/cgi-bin/carddisp.pl?gene=CLCF1
CEBPZ	CCAAT Enhancer Binding Protein Zeta	Protein Coding	46	GC02M037201	1.425809264	https://www.genecards.org/cgi-bin/carddisp.pl?gene=CEBPZ
CRIP1	Cysteine Rich Protein 1	Protein Coding	43	GC14P105486	1.425279856	https://www.genecards.org/cgi-bin/carddisp.pl?gene=CRIP1
ICAM2	Intercellular Adhesion Molecule 2	Protein Coding	51	GC17M064002	1.424790382	https://www.genecards.org/cgi-bin/carddisp.pl?gene=ICAM2
PTK6	Protein Tyrosine Kinase 6	Protein Coding	54	GC20M063528	1.424619913	https://www.genecards.org/cgi-bin/carddisp.pl?gene=PTK6
RGS19	Regulator Of G Protein Signaling 19	Protein Coding	48	GC20M064073	1.423856258	https://www.genecards.org/cgi-bin/carddisp.pl?gene=RGS19
CALD1	Caldesmon 1	Protein Coding	50	GC07P134744	1.423612356	https://www.genecards.org/cgi-bin/carddisp.pl?gene=CALD1
CTRB1	Chymotrypsinogen B1	Protein Coding	44	GC16P075218	1.420039415	https://www.genecards.org/cgi-bin/carddisp.pl?gene=CTRB1
SIRPA	Signal Regulatory Protein Alpha	Protein Coding	52	GC20P001894	1.419333458	https://www.genecards.org/cgi-bin/carddisp.pl?gene=SIRPA
NDN	Necdin, MAGE Family Member	Protein Coding	48	GC15M024541	1.415067673	https://www.genecards.org/cgi-bin/carddisp.pl?gene=NDN

UBE2E2	Ubiquitin Conjugating Enzyme E2 E2	Protein Coding	46	GC03P023221	1.414947867	https://www.genecards.org/cgi-bin/carddisp.pl?gene=UBE2E2
CDY1	Chromodomain Y-Linked 1	Protein Coding	35	GC0YP026181	1.41431427	https://www.genecards.org/cgi-bin/carddisp.pl?gene=CDY1
HECA	Hdc Homolog, Cell Cycle Regulator	Protein Coding	39	GC06P139135	1.412382364	https://www.genecards.org/cgi-bin/carddisp.pl?gene=HECA
S1PR3	Sphingosine-1-Phosphate Receptor 3	Protein Coding	52	GC09P089518	1.411020994	https://www.genecards.org/cgi-bin/carddisp.pl?gene=S1PR3
ID1	Inhibitor Of DNA Binding 1	Protein Coding	47	GC20P031605	1.410467505	https://www.genecards.org/cgi-bin/carddisp.pl?gene=ID1
SLC38A5	Solute Carrier Family 38 Member 5	Protein Coding	44	GC0XM048458	1.409354568	https://www.genecards.org/cgi-bin/carddisp.pl?gene=SLC38A5
CCDC6	Coiled-Coil Domain Containing 6	Protein Coding	47	GC10M059788	1.406749368	https://www.genecards.org/cgi-bin/carddisp.pl?gene=CCDC6
TIRAP	TIR Domain Containing Adaptor Protein	Protein Coding	50	GC11P126284	1.405854225	https://www.genecards.org/cgi-bin/carddisp.pl?gene=TIRAP
ADAD1	Adenosine Deaminase Domain Containing 1	Protein Coding	41	GC04P122378	1.405854225	https://www.genecards.org/cgi-bin/carddisp.pl?gene=ADAD1
GLYATL2	Glycine-N-Acyltransferase Like 2	Protein Coding	38	GC11M099448	1.405657053	https://www.genecards.org/cgi-bin/carddisp.pl?gene=GLYATL2
GOLGA5	Golgin A5	Protein Coding	44	GC14P092794	1.404361129	https://www.genecards.org/cgi-bin/carddisp.pl?gene=GOLGA5
CXCL2	C-X-C Motif Chemokine Ligand 2	Protein Coding	46	GC04M074097	1.401093721	https://www.genecards.org/cgi-bin/carddisp.pl?gene=CXCL2
INSRR	Insulin Receptor Related Receptor	Protein Coding	50	GC01M156840	1.400798559	https://www.genecards.org/cgi-bin/carddisp.pl?gene=INSRR
UGT2A3	UDP Glucuronosyltransferase Family 2 Member A3	Protein Coding	44	GC04M068928	1.396822453	https://www.genecards.org/cgi-bin/carddisp.pl?gene=UGT2A3
GJB1	Gap Junction Protein Beta 1	Protein Coding	53	GC0XP071212	1.396408558	https://www.genecards.org/cgi-bin/carddisp.pl?gene=GJB1

AZU1	Azurocidin 1	Protein Coding	46	GC19P000825	1.392551899	https://www.genecards.org/cgi-bin/carddisp.pl?gene=AZU1
LILRB2	Leukocyte Immunoglobulin Like Receptor B2	Protein Coding	46	GC19M071132	1.392198324	https://www.genecards.org/cgi-bin/carddisp.pl?gene=LILRB2
PHOX2A	Paired Like Homeobox 2A	Protein Coding	48	GC11M072239	1.391584873	https://www.genecards.org/cgi-bin/carddisp.pl?gene=PHOX2A
ADARB1	Adenosine Deaminase RNA Specific B1	Protein Coding	51	GC21P045073	1.391404986	https://www.genecards.org/cgi-bin/carddisp.pl?gene=ADARB1
CD160	CD160 Molecule	Protein Coding	48	GC01P145719	1.386499882	https://www.genecards.org/cgi-bin/carddisp.pl?gene=CD160
CD226	CD226 Molecule	Protein Coding	48	GC18M069831	1.386499882	https://www.genecards.org/cgi-bin/carddisp.pl?gene=CD226
FLT3LG	Fms Related Receptor Tyrosine Kinase 3 Ligand	Protein Coding	46	GC19P073231	1.38465929	https://www.genecards.org/cgi-bin/carddisp.pl?gene=FLT3LG
SNX13	Sorting Nexin 13	Protein Coding	43	GC07M017798	1.38465929	https://www.genecards.org/cgi-bin/carddisp.pl?gene=SNX13
PTPRH	Protein Tyrosine Phosphatase Receptor Type H	Protein Coding	46	GC19M055181	1.382741332	https://www.genecards.org/cgi-bin/carddisp.pl?gene=PTPRH
CLDN4	Claudin 4	Protein Coding	46	GC07P073799	1.380390525	https://www.genecards.org/cgi-bin/carddisp.pl?gene=CLDN4
GET1	Guided Entry Of Tail-Anchored Proteins Factor 1	Protein Coding	41	GC21P039377	1.379169941	https://www.genecards.org/cgi-bin/carddisp.pl?gene=GET1
MFAP2	Microfibril Associated Protein 2	Protein Coding	47	GC01M016974	1.378049135	https://www.genecards.org/cgi-bin/carddisp.pl?gene=MFAP2
CASQ2	Calsequestrin 2	Protein Coding	51	GC01M115700	1.377944827	https://www.genecards.org/cgi-bin/carddisp.pl?gene=CASQ2
ANXA4	Annexin A4	Protein Coding	48	GC02P069644	1.377457976	https://www.genecards.org/cgi-bin/carddisp.pl?gene=ANXA4
TMPRSS2	Transmembrane Serine Protease 2	Protein Coding	55	GC21M041464	1.37644577	https://www.genecards.org/cgi-bin/carddisp.pl?gene=TMPRSS2

MYBL2	MYB Proto-Oncogene Like 2	Protein Coding	49	GC20P043667	1.375777125	https://www.genecards.org/cgi-bin/carddisp.pl?gene=MYBL2
ERC1	ELKS/RAB6-Interacting/CAST Family Member 1	Protein Coding	48	GC12P000972	1.375777125	https://www.genecards.org/cgi-bin/carddisp.pl?gene=ERC1
RASA2	RAS P21 Protein Activator 2	Protein Coding	46	GC03P141487	1.37325263	https://www.genecards.org/cgi-bin/carddisp.pl?gene=RASA2
CD244	CD244 Molecule	Protein Coding	50	GC01M160830	1.372381926	https://www.genecards.org/cgi-bin/carddisp.pl?gene=CD244
GTF2B	General Transcription Factor IIB	Protein Coding	47	GC01M088853	1.372071028	https://www.genecards.org/cgi-bin/carddisp.pl?gene=GTF2B
GFRA1	GDNF Family Receptor Alpha 1	Protein Coding	53	GC10M116056	1.371281862	https://www.genecards.org/cgi-bin/carddisp.pl?gene=GFRA1
BAG1	BAG Cochaperone 1	Protein Coding	47	GC09M033245	1.370138407	https://www.genecards.org/cgi-bin/carddisp.pl?gene=BAG1
CDC25B	Cell Division Cycle 25B	Protein Coding	52	GC20P004512	1.369985104	https://www.genecards.org/cgi-bin/carddisp.pl?gene=CDC25B
DEFB4A	Defensin Beta 4A	Protein Coding	42	GC08P007900	1.367897034	https://www.genecards.org/cgi-bin/carddisp.pl?gene=DEFB4A
SPAG9	Sperm Associated Antigen 9	Protein Coding	47	GC17M050962	1.366934896	https://www.genecards.org/cgi-bin/carddisp.pl?gene=SPAG9
ITGB5	Integrin Subunit Beta 5	Protein Coding	53	GC03M124761	1.364701748	https://www.genecards.org/cgi-bin/carddisp.pl?gene=ITGB5
EPS15L1	Epidermal Growth Factor Receptor Pathway Substrate 15 Like 1	Protein Coding	46	GC19M016333	1.364116907	https://www.genecards.org/cgi-bin/carddisp.pl?gene=EPS15L1
CTNNA1	Catenin Alpha 1	Protein Coding	52	GC05P138613	1.36270237	https://www.genecards.org/cgi-bin/carddisp.pl?gene=CTNNA1
TAGLN	Transgelin	Protein Coding	50	GC11P117199	1.360505223	https://www.genecards.org/cgi-bin/carddisp.pl?gene=TAGLN
ARHGDIB	Rho GDP Dissociation Inhibitor Beta	Protein Coding	48	GC12M014942	1.357345819	https://www.genecards.org/cgi-bin/carddisp.pl?gene=ARHGDIB

MSMB	Microseminoprotein Beta	Protein Coding	47	GC10M046033	1.356166363	https://www.genecards.org/cgi-bin/carddisp.pl?gene=MSMB
PTN	Pleiotrophin	Protein Coding	47	GC07M137227	1.355832815	https://www.genecards.org/cgi-bin/carddisp.pl?gene=PTN
MEIS1	Meis Homeobox 1	Protein Coding	48	GC02P066433	1.35497427	https://www.genecards.org/cgi-bin/carddisp.pl?gene=MEIS1
EPHA1	EPH Receptor A1	Protein Coding	54	GC07M143390	1.35342288	https://www.genecards.org/cgi-bin/carddisp.pl?gene=EPHA1
YES1	YES Proto-Oncogene 1, Src Family Tyrosine Kinase	Protein Coding	54	GC18M000721	1.35342288	https://www.genecards.org/cgi-bin/carddisp.pl?gene=YES1
LAIR1	Leukocyte Associated Immunoglobulin Like Receptor 1	Protein Coding	48	GC19M054351	1.353043079	https://www.genecards.org/cgi-bin/carddisp.pl?gene=LAIR1
TFPT	TCF3 Fusion Partner	Protein Coding	42	GC19M054107	1.352261066	https://www.genecards.org/cgi-bin/carddisp.pl?gene=TFPT
SH2B2	SH2B Adaptor Protein 2	Protein Coding	44	GC07P102286	1.35112071	https://www.genecards.org/cgi-bin/carddisp.pl?gene=SH2B2
GRIN2C	Glutamate Ionotropic Receptor NMDA Type Subunit 2C	Protein Coding	52	GC17M074842	1.350762725	https://www.genecards.org/cgi-bin/carddisp.pl?gene=GRIN2C
P2RY1	Purinergic Receptor P2Y1	Protein Coding	53	GC03P152835	1.347520828	https://www.genecards.org/cgi-bin/carddisp.pl?gene=P2RY1
IL3RA	Interleukin 3 Receptor Subunit Alpha	Protein Coding	51	GC0XP001336	1.346916914	https://www.genecards.org/cgi-bin/carddisp.pl?gene=IL3RA
IL1RAP	Interleukin 1 Receptor Accessory Protein	Protein Coding	53	GC03P190514	1.346740007	https://www.genecards.org/cgi-bin/carddisp.pl?gene=IL1RAP
EFNA1	Ephrin A1	Protein Coding	51	GC01P155127	1.343958974	https://www.genecards.org/cgi-bin/carddisp.pl?gene=EFNA1
GLI1	GLI Family Zinc Finger 1	Protein Coding	56	GC12P057616	1.343919992	https://www.genecards.org/cgi-bin/carddisp.pl?gene=GLI1
RHOD	Ras Homolog Family Member D	Protein Coding	43	GC11P071007	1.342387915	https://www.genecards.org/cgi-bin/carddisp.pl?gene=RHOD

PTPRM	Protein Tyrosine Phosphatase Receptor Type M	Protein Coding	51	GC18P007557	1.341762543	https://www.genecards.org/cgi-bin/carddisp.pl?gene=PTPRM
ZDHHC20	Zinc Finger DHHC-Type Palmitoyltransferase 20	Protein Coding	42	GC13M021570	1.341608047	https://www.genecards.org/cgi-bin/carddisp.pl?gene=ZDHHC20
CD72	CD72 Molecule	Protein Coding	46	GC09M035610	1.341193438	https://www.genecards.org/cgi-bin/carddisp.pl?gene=CD72
IRF2	Interferon Regulatory Factor 2	Protein Coding	49	GC04M184387	1.339345694	https://www.genecards.org/cgi-bin/carddisp.pl?gene=IRF2
ETF1	Eukaryotic Translation Termination Factor 1	Protein Coding	49	GC05M138506	1.33785367	https://www.genecards.org/cgi-bin/carddisp.pl?gene=ETF1
CD3G	CD3 Gamma Subunit Of T-Cell Receptor Complex	Protein Coding	54	GC11P118344	1.336899042	https://www.genecards.org/cgi-bin/carddisp.pl?gene=CD3G
CACNG2	Calcium Voltage-Gated Channel Auxiliary Subunit Gamma 2	Protein Coding	51	GC22M061447	1.336666226	https://www.genecards.org/cgi-bin/carddisp.pl?gene=CACNG2
CASP6	Caspase 6	Protein Coding	55	GC04M109688	1.335477829	https://www.genecards.org/cgi-bin/carddisp.pl?gene=CASP6
DST	Dystonin	Protein Coding	49	GC06M056457	1.333791614	https://www.genecards.org/cgi-bin/carddisp.pl?gene=DST
SIGLEC1	Sialic Acid Binding Ig Like Lectin 1	Protein Coding	47	GC20M003686	1.33364892	https://www.genecards.org/cgi-bin/carddisp.pl?gene=SIGLEC1
SPIB	Spi-B Transcription Factor	Protein Coding	46	GC19P050418	1.333257198	https://www.genecards.org/cgi-bin/carddisp.pl?gene=SPIB
CBLB	Cbl Proto-Oncogene B	Protein Coding	52	GC03M105655	1.332267404	https://www.genecards.org/cgi-bin/carddisp.pl?gene=CBLB
CBLC	Cbl Proto-Oncogene C	Protein Coding	44	GC19P044777	1.332267404	https://www.genecards.org/cgi-bin/carddisp.pl?gene=CBLC
ISG20	Interferon Stimulated Exonuclease Gene 20	Protein Coding	46	GC15P088635	1.330898881	https://www.genecards.org/cgi-bin/carddisp.pl?gene=ISG20
TGFBR3	Transforming Growth Factor Beta Receptor 3	Protein Coding	53	GC01M091680	1.329393387	https://www.genecards.org/cgi-bin/carddisp.pl?gene=TGFBR3

PTPRE	Protein Tyrosine Phosphatase Receptor Type E	Protein Coding	49	GC10P127907	1.328865051	https://www.genecards.org/cgi-bin/carddisp.pl?gene=PTPRE
HMGN1	High Mobility Group Nucleosome Binding Domain 1	Protein Coding	47	GC21M039342	1.328797698	https://www.genecards.org/cgi-bin/carddisp.pl?gene=HMGN1
LPAR2	Lysophosphatidic Acid Receptor 2	Protein Coding	52	GC19M019624	1.32547009	https://www.genecards.org/cgi-bin/carddisp.pl?gene=LPAR2
TM9SF2	Transmembrane 9 Superfamily Member 2	Protein Coding	42	GC13P099446	1.323809385	https://www.genecards.org/cgi-bin/carddisp.pl?gene=TM9SF2
CCL8	C-C Motif Chemokine Ligand 8	Protein Coding	46	GC17P034319	1.31999135	https://www.genecards.org/cgi-bin/carddisp.pl?gene=CCL8
NPSR1	Neuropeptide S Receptor 1	Protein Coding	47	GC07P034664	1.319486618	https://www.genecards.org/cgi-bin/carddisp.pl?gene=NPSR1
AGFG1	ArfGAP With FG Repeats 1	Protein Coding	47	GC02P227473	1.318310976	https://www.genecards.org/cgi-bin/carddisp.pl?gene=AGFG1
RECQL	RecQ Like Helicase	Protein Coding	48	GC12M021468	1.316822648	https://www.genecards.org/cgi-bin/carddisp.pl?gene=RECQL
GAK	Cyclin G Associated Kinase	Protein Coding	51	GC04M000849	1.314225674	https://www.genecards.org/cgi-bin/carddisp.pl?gene=GAK
MAP3K4	Mitogen-Activated Protein Kinase Kinase Kinase 4	Protein Coding	50	GC06P160991	1.314225674	https://www.genecards.org/cgi-bin/carddisp.pl?gene=MAP3K4
DUSP5	Dual Specificity Phosphatase 5	Protein Coding	48	GC10P110497	1.314225674	https://www.genecards.org/cgi-bin/carddisp.pl?gene=DUSP5
FSCN1	Fascin Actin-Bundling Protein 1	Protein Coding	51	GC07P005592	1.31301713	https://www.genecards.org/cgi-bin/carddisp.pl?gene=FSCN1
PAX3	Paired Box 3	Protein Coding	52	GC02M222199	1.312791586	https://www.genecards.org/cgi-bin/carddisp.pl?gene=PAX3
STYX	Serine/Threonine/Tyrosine Interacting Protein	Protein Coding	41	GC14P052730	1.310669065	https://www.genecards.org/cgi-bin/carddisp.pl?gene=STYX
GNPNAT1	Glucosamine-Phosphate N-Acetyltransferase 1	Protein Coding	46	GC14M052775	1.30973053	https://www.genecards.org/cgi-bin/carddisp.pl?gene=GNPNAT1

HOXA1	Homeobox A1	Protein Coding	48	GC07M027092	1.308444142	https://www.genecards.org/cgi-bin/carddisp.pl?gene=HOXA1
ZP3	Zona Pellucida Glycoprotein 3	Protein Coding	50	GC07P076837	1.305477381	https://www.genecards.org/cgi-bin/carddisp.pl?gene=ZP3
PAK3	P21 (RAC1) Activated Kinase 3	Protein Coding	57	GC0XP110944	1.301856756	https://www.genecards.org/cgi-bin/carddisp.pl?gene=PAK3
EFNA4	Ephrin A4	Protein Coding	50	GC01P155063	1.301198006	https://www.genecards.org/cgi-bin/carddisp.pl?gene=EFNA4
CD84	CD84 Molecule	Protein Coding	49	GC01M160541	1.301198006	https://www.genecards.org/cgi-bin/carddisp.pl?gene=CD84
CTNND2	Catenin Delta 2	Protein Coding	49	GC05M010971	1.297608376	https://www.genecards.org/cgi-bin/carddisp.pl?gene=CTNND2
CYTH1	Cytohesin 1	Protein Coding	48	GC17M078674	1.297608376	https://www.genecards.org/cgi-bin/carddisp.pl?gene=CYTH1
TESK1	Testis Associated Actin Remodelling Kinase 1	Protein Coding	48	GC09P035605	1.296860695	https://www.genecards.org/cgi-bin/carddisp.pl?gene=TESK1
NECTIN1	Nectin Cell Adhesion Molecule 1	Protein Coding	53	GC11M119624	1.296584606	https://www.genecards.org/cgi-bin/carddisp.pl?gene=NECTIN1
WASF2	WASP Family Member 2	Protein Coding	50	GC01M027404	1.296584606	https://www.genecards.org/cgi-bin/carddisp.pl?gene=WASF2
NCOA4	Nuclear Receptor Coactivator 4	Protein Coding	46	GC10M046005	1.295899391	https://www.genecards.org/cgi-bin/carddisp.pl?gene=NCOA4
TRIP10	Thyroid Hormone Receptor Interactor 10	Protein Coding	47	GC19P006737	1.292088509	https://www.genecards.org/cgi-bin/carddisp.pl?gene=TRIP10
PRSS3	Serine Protease 3	Protein Coding	50	GC09P033750	1.290347695	https://www.genecards.org/cgi-bin/carddisp.pl?gene=PRSS3
GRIN2D	Glutamate Ionotropic Receptor NMDA Type Subunit 2D	Protein Coding	55	GC19P073182	1.288183808	https://www.genecards.org/cgi-bin/carddisp.pl?gene=GRIN2D
KLRD1	Killer Cell Lectin Like Receptor D1	Protein Coding	48	GC12P010226	1.287742376	https://www.genecards.org/cgi-bin/carddisp.pl?gene=KLRD1

MAP4K1	Mitogen-Activated Protein Kinase Kinase Kinase Kinase 1	Protein Coding	50	GC19M038587	1.286914349	https://www.genecards.org/cgi-bin/carddisp.pl?gene=MAP4K1
KIF5A	Kinesin Family Member 5A	Protein Coding	51	GC12P057555	1.28510344	https://www.genecards.org/cgi-bin/carddisp.pl?gene=KIF5A
GYPC	Glycophorin C (Gerbich Blood Group)	Protein Coding	50	GC02P126655	1.281240463	https://www.genecards.org/cgi-bin/carddisp.pl?gene=GYPC
SORBS2	Sorbin And SH3 Domain Containing 2	Protein Coding	47	GC04M185585	1.275793552	https://www.genecards.org/cgi-bin/carddisp.pl?gene=SORBS2
EFS	Embryonal Fyn-Associated Substrate	Protein Coding	41	GC14M023356	1.274370074	https://www.genecards.org/cgi-bin/carddisp.pl?gene=EFS
PTPRT	Protein Tyrosine Phosphatase Receptor Type T	Protein Coding	51	GC20M042072	1.274230242	https://www.genecards.org/cgi-bin/carddisp.pl?gene=PTPRT
MAP3K13	Mitogen-Activated Protein Kinase Kinase Kinase 13	Protein Coding	48	GC03P185282	1.274230242	https://www.genecards.org/cgi-bin/carddisp.pl?gene=MAP3K13
ABCC10	ATP Binding Cassette Subfamily C Member 10	Protein Coding	45	GC06P043427	1.270961523	https://www.genecards.org/cgi-bin/carddisp.pl?gene=ABCC10
SH2D4B	SH2 Domain Containing 4B	Protein Coding	38	GC10P094472	1.270071507	https://www.genecards.org/cgi-bin/carddisp.pl?gene=SH2D4B
PLCXD3	Phosphatidylinositol Specific Phospholipase C X Domain Containing 3	Protein Coding	43	GC05M041306	1.269739866	https://www.genecards.org/cgi-bin/carddisp.pl?gene=PLCXD3
GMNN	Geminin DNA Replication Inhibitor	Protein Coding	51	GC06P024779	1.268566847	https://www.genecards.org/cgi-bin/carddisp.pl?gene=GMNN
NFE2	Nuclear Factor, Erythroid 2	Protein Coding	46	GC12M054292	1.266424775	https://www.genecards.org/cgi-bin/carddisp.pl?gene=NFE2
SLC15A4	Solute Carrier Family 15 Member 4	Protein Coding	44	GC12M128793	1.263706923	https://www.genecards.org/cgi-bin/carddisp.pl?gene=SLC15A4
RPL10L	Ribosomal Protein L10 Like	Protein Coding	42	GC14M046651	1.262371421	https://www.genecards.org/cgi-bin/carddisp.pl?gene=RPL10L
GRM6	Glutamate Metabotropic Receptor 6	Protein Coding	53	GC05M178978	1.260549188	https://www.genecards.org/cgi-bin/carddisp.pl?gene=GRM6

DOCK1	Dedicator Of Cytokinesis 1	Protein Coding	50	GC10P126905	1.260548592	https://www.genecards.org/cgi-bin/carddisp.pl?gene=DOCK1
GRIP2	Glutamate Receptor Interacting Protein 2	Protein Coding	42	GC03M021835	1.258551359	https://www.genecards.org/cgi-bin/carddisp.pl?gene=GRIP2
NRBF2	Nuclear Receptor Binding Factor 2	Protein Coding	43	GC10P063133	1.257070303	https://www.genecards.org/cgi-bin/carddisp.pl?gene=NRBF2
ADD2	Adducin 2	Protein Coding	47	GC02M070626	1.252891302	https://www.genecards.org/cgi-bin/carddisp.pl?gene=ADD2
CNTNAP1	Contactin Associated Protein 1	Protein Coding	50	GC17P042682	1.252706528	https://www.genecards.org/cgi-bin/carddisp.pl?gene=CNTNAP1
MDK	Midkine	Protein Coding	50	GC11P046380	1.25058341	https://www.genecards.org/cgi-bin/carddisp.pl?gene=MDK
ZBTB11	Zinc Finger And BTB Domain Containing 11	Protein Coding	42	GC03M101648	1.248044372	https://www.genecards.org/cgi-bin/carddisp.pl?gene=ZBTB11
CNTNAP2	Contactin Associated Protein 2	Protein Coding	51	GC07P146116	1.244960666	https://www.genecards.org/cgi-bin/carddisp.pl?gene=CNTNAP2
ZDHHC21	Zinc Finger DHHC-Type Palmitoyltransferase 21	Protein Coding	40	GC09M014590	1.242198229	https://www.genecards.org/cgi-bin/carddisp.pl?gene=ZDHHC21
TSC22D3	TSC22 Domain Family Member 3	Protein Coding	46	GC0XM107713	1.240224838	https://www.genecards.org/cgi-bin/carddisp.pl?gene=TSC22D3
KAT6B	Lysine Acetyltransferase 6B	Protein Coding	52	GC10P074873	1.239717484	https://www.genecards.org/cgi-bin/carddisp.pl?gene=KAT6B
DFFB	DNA Fragmentation Factor Subunit Beta	Protein Coding	50	GC01P003857	1.237127662	https://www.genecards.org/cgi-bin/carddisp.pl?gene=DFFB
CD6	CD6 Molecule	Protein Coding	48	GC11P060971	1.236596346	https://www.genecards.org/cgi-bin/carddisp.pl?gene=CD6
GRB7	Growth Factor Receptor Bound Protein 7	Protein Coding	50	GC17P061875	1.234689474	https://www.genecards.org/cgi-bin/carddisp.pl?gene=GRB7
CDYL	Chromodomain Y Like	Protein Coding	46	GC06P004706	1.230699301	https://www.genecards.org/cgi-bin/carddisp.pl?gene=CDYL

EPHB6	EPH Receptor B6	Protein Coding	51	GC07P149981	1.230352283	https://www.genecards.org/cgi-bin/carddisp.pl?gene=EPHB6
STAM	Signal Transducing Adaptor Molecule	Protein Coding	47	GC10P017698	1.228438377	https://www.genecards.org/cgi-bin/carddisp.pl?gene=STAM
CYRIB	CYFIP Related Rac1 Interactor B	Protein Coding	40	GC08M132565	1.221472621	https://www.genecards.org/cgi-bin/carddisp.pl?gene=CYRIB
GLRA2	Glycine Receptor Alpha 2	Protein Coding	52	GC0XP014448	1.221354008	https://www.genecards.org/cgi-bin/carddisp.pl?gene=GLRA2
SNU13	Small Nuclear Ribonucleoprotein 13	Protein Coding	46	GC22M061492	1.218278646	https://www.genecards.org/cgi-bin/carddisp.pl?gene=SNU13
TREM1	Triggering Receptor Expressed On Myeloid Cells 1	Protein Coding	48	GC06M041267	1.218127251	https://www.genecards.org/cgi-bin/carddisp.pl?gene=TREM1
ZDHHC1	Zinc Finger DHHC-Type Containing 1	Protein Coding	42	GC16M067394	1.217028856	https://www.genecards.org/cgi-bin/carddisp.pl?gene=ZDHHC1
NFASC	Neurofascin	Protein Coding	50	GC01P204828	1.212895989	https://www.genecards.org/cgi-bin/carddisp.pl?gene=NFASC
TYROBP	Transmembrane Immune Signaling Adaptor TYROBP	Protein Coding	50	GC19M035904	1.212895989	https://www.genecards.org/cgi-bin/carddisp.pl?gene=TYROBP
RHOQ	Ras Homolog Family Member Q	Protein Coding	48	GC02P046543	1.205293775	https://www.genecards.org/cgi-bin/carddisp.pl?gene=RHOQ
NEDD9	Neural Precursor Cell Expressed, Developmentally Down-Regulated 9	Protein Coding	48	GC06M011183	1.203222752	https://www.genecards.org/cgi-bin/carddisp.pl?gene=NEDD9
ZDHHC17	Zinc Finger DHHC-Type Palmitoyltransferase 17	Protein Coding	44	GC12P076763	1.20134306	https://www.genecards.org/cgi-bin/carddisp.pl?gene=ZDHHC17
MARCKSL 1	MARCKS Like 1	Protein Coding	43	GC01M032334	1.201022148	https://www.genecards.org/cgi-bin/carddisp.pl?gene=MARCKSL1
GUCA2A	Guanylate Cyclase Activator 2A	Protein Coding	43	GC01M042162	1.200126886	https://www.genecards.org/cgi-bin/carddisp.pl?gene=GUCA2A
SLC15A3	Solute Carrier Family 15 Member 3	Protein Coding	43	GC11M099519	1.196174622	https://www.genecards.org/cgi-bin/carddisp.pl?gene=SLC15A3

METTL6	Methyltransferase 6, Methylcytidine	Protein Coding	40	GC03M021855	1.196174622	https://www.genecards.org/cgi-bin/carddisp.pl?gene=METTL6
STX4	Syntaxin 4	Protein Coding	48	GC16P042831	1.193569422	https://www.genecards.org/cgi-bin/carddisp.pl?gene=STX4
DNASE1	Deoxyribonuclease 1	Protein Coding	50	GC16P003611	1.188389778	https://www.genecards.org/cgi-bin/carddisp.pl?gene=DNASE1
SIGMAR1	Sigma Non-Opioid Intracellular Receptor 1	Protein Coding	54	GC09M034634	1.185390472	https://www.genecards.org/cgi-bin/carddisp.pl?gene=SIGMAR1
CDH3	Cadherin 3	Protein Coding	54	GC16P068637	1.185385942	https://www.genecards.org/cgi-bin/carddisp.pl?gene=CDH3
BAMBI	BMP And Activin Membrane Bound Inhibitor	Protein Coding	50	GC10P028685	1.185385942	https://www.genecards.org/cgi-bin/carddisp.pl?gene=BAMBI
TLX1	T Cell Leukemia Homeobox 1	Protein Coding	46	GC10P101130	1.180054665	https://www.genecards.org/cgi-bin/carddisp.pl?gene=TLX1
KIR2DL1	Killer Cell Immunoglobulin Like Receptor, Two Ig Domains And Long Cytoplasmic Tail 1	Protein Coding	42	GC19P073500	1.180054665	https://www.genecards.org/cgi-bin/carddisp.pl?gene=KIR2DL1
FGR	FGR Proto-Oncogene, Src Family Tyrosine Kinase	Protein Coding	53	GC01M028092	1.177772641	https://www.genecards.org/cgi-bin/carddisp.pl?gene=FGR
ARHGEF12	Rho Guanine Nucleotide Exchange Factor 12	Protein Coding	49	GC11P120336	1.177476287	https://www.genecards.org/cgi-bin/carddisp.pl?gene=ARHGEF12
ERVK-6	Endogenous Retrovirus Group K Member 6, Envelope	Protein Coding	21	GC07U903184	1.177476287	https://www.genecards.org/cgi-bin/carddisp.pl?gene=ERVK-6
CDY2A	Chromodomain Y-Linked 2A	Protein Coding	31	GC0YP018025	1.177319527	https://www.genecards.org/cgi-bin/carddisp.pl?gene=CDY2A
KAT8	Lysine Acetyltransferase 8	Protein Coding	51	GC16P042841	1.171679974	https://www.genecards.org/cgi-bin/carddisp.pl?gene=KAT8
UBFD1	Ubiquitin Family Domain Containing 1	Protein Coding	39	GC16P023557	1.170049906	https://www.genecards.org/cgi-bin/carddisp.pl?gene=UBFD1
ETV4	ETS Variant Transcription Factor 4	Protein Coding	49	GC17M043527	1.163003087	https://www.genecards.org/cgi-bin/carddisp.pl?gene=ETV4

GFRA2	GDNF Family Receptor Alpha 2	Protein Coding	51	GC08M021690	1.161565185	https://www.genecards.org/cgi-bin/carddisp.pl?gene=GFRA2
CBFA2T2	CBFA2/RUNX1 Partner Transcriptional Co-Repressor 2	Protein Coding	43	GC20P033490	1.158389688	https://www.genecards.org/cgi-bin/carddisp.pl?gene=CBFA2T2
LACRT	Lacritin	Protein Coding	38	GC12M055055	1.158389688	https://www.genecards.org/cgi-bin/carddisp.pl?gene=LACRT
CRIPT	CXXC Repeat Containing Interactor Of PDZ3 Domain	Protein Coding	46	GC02P046616	1.155421019	https://www.genecards.org/cgi-bin/carddisp.pl?gene=CRIPT
HDAC11	Histone Deacetylase 11	Protein Coding	51	GC03P013749	1.151618719	https://www.genecards.org/cgi-bin/carddisp.pl?gene=HDAC11
S100A2	S100 Calcium Binding Protein A2	Protein Coding	47	GC01M153561	1.150105476	https://www.genecards.org/cgi-bin/carddisp.pl?gene=S100A2
CD96	CD96 Molecule	Protein Coding	51	GC03P111292	1.148190379	https://www.genecards.org/cgi-bin/carddisp.pl?gene=CD96
MMP20	Matrix Metallopeptidase 20	Protein Coding	51	GC11M102576	1.147861481	https://www.genecards.org/cgi-bin/carddisp.pl?gene=MMP20
STK10	Serine/Threonine Kinase 10	Protein Coding	50	GC05M172042	1.147695661	https://www.genecards.org/cgi-bin/carddisp.pl?gene=STK10
CCDC26	CCDC26 Long Non-Coding RNA	RNA Gene	31	GC08M132556	1.144457102	https://www.genecards.org/cgi-bin/carddisp.pl?gene=CCDC26
ITGA1	Integrin Subunit Alpha 1	Protein Coding	51	GC05P052788	1.142069697	https://www.genecards.org/cgi-bin/carddisp.pl?gene=ITGA1
CLEC4D	C-Type Lectin Domain Family 4 Member D	Protein Coding	44	GC12P008509	1.141966224	https://www.genecards.org/cgi-bin/carddisp.pl?gene=CLEC4D
PSPN	Persephin	Protein Coding	43	GC19M006375	1.141966224	https://www.genecards.org/cgi-bin/carddisp.pl?gene=PSPN
QPCT	Glutaminyl-Peptide Cyclotransferase	Protein Coding	50	GC02P037344	1.141539812	https://www.genecards.org/cgi-bin/carddisp.pl?gene=QPCT
KCNK3	Potassium Two Pore Domain Channel Subfamily K Member 3	Protein Coding	57	GC02P026692	1.140071511	https://www.genecards.org/cgi-bin/carddisp.pl?gene=KCNK3

KLRC1	Killer Cell Lectin Like Receptor C1	Protein Coding	48	GC12M022635	1.138934731	https://www.genecards.org/cgi-bin/carddisp.pl?gene=KLRC1
SPINT1	Serine Peptidase Inhibitor, Kunitz Type 1	Protein Coding	48	GC15P040844	1.138934731	https://www.genecards.org/cgi-bin/carddisp.pl?gene=SPINT1
SS18	SS18 Subunit Of BAF Chromatin Remodeling Complex	Protein Coding	46	GC18M026016	1.138934731	https://www.genecards.org/cgi-bin/carddisp.pl?gene=SS18
TSPAN32	Tetraspanin 32	Protein Coding	42	GC11P002302	1.138934731	https://www.genecards.org/cgi-bin/carddisp.pl?gene=TSPAN32
GRM4	Glutamate Metabotropic Receptor 4	Protein Coding	52	GC06M071850	1.136897206	https://www.genecards.org/cgi-bin/carddisp.pl?gene=GRM4
TNK2	Tyrosine Kinase Non Receptor 2	Protein Coding	54	GC03M195863	1.136035442	https://www.genecards.org/cgi-bin/carddisp.pl?gene=TNK2
NSUN5	NOP2/Sun RNA Methyltransferase 5	Protein Coding	45	GC07M073302	1.136035442	https://www.genecards.org/cgi-bin/carddisp.pl?gene=NSUN5
AFAP1	Actin Filament Associated Protein 1	Protein Coding	45	GC04M007758	1.135466218	https://www.genecards.org/cgi-bin/carddisp.pl?gene=AFAP1
ARHGEF11	Rho Guanine Nucleotide Exchange Factor 11	Protein Coding	48	GC01M156934	1.134341598	https://www.genecards.org/cgi-bin/carddisp.pl?gene=ARHGEF11
CTRL	Chymotrypsin Like	Protein Coding	47	GC16M067927	1.133171082	https://www.genecards.org/cgi-bin/carddisp.pl?gene=CTRL
MCF2	MCF.2 Cell Line Derived Transforming Sequence	Protein Coding	47	GC0XM139581	1.130852818	https://www.genecards.org/cgi-bin/carddisp.pl?gene=MCF2
GLRA4	Glycine Receptor Alpha 4 (Pseudogene)	Pseudogene	32	GC0XM103707	1.126724005	https://www.genecards.org/cgi-bin/carddisp.pl?gene=GLRA4
NCK1	NCK Adaptor Protein 1	Protein Coding	50	GC03P136862	1.126284242	https://www.genecards.org/cgi-bin/carddisp.pl?gene=NCK1
FGF5	Fibroblast Growth Factor 5	Protein Coding	50	GC04P080266	1.118101239	https://www.genecards.org/cgi-bin/carddisp.pl?gene=FGF5
HDAC10	Histone Deacetylase 10	Protein Coding	51	GC22M050245	1.117851496	https://www.genecards.org/cgi-bin/carddisp.pl?gene=HDAC10

VGLL3	Vestigial Like Family Member 3	Protein Coding	41	GC03M086876	1.116021156	https://www.genecards.org/cgi-bin/carddisp.pl?gene=VGLL3
HCLS1	Hematopoietic Cell-Specific Lyn Substrate 1	Protein Coding	50	GC03M121631	1.114315152	https://www.genecards.org/cgi-bin/carddisp.pl?gene=HCLS1
LY9	Lymphocyte Antigen 9	Protein Coding	47	GC01P160796	1.113487959	https://www.genecards.org/cgi-bin/carddisp.pl?gene=LY9
MYO6	Myosin VI	Protein Coding	51	GC06P075749	1.11263597	https://www.genecards.org/cgi-bin/carddisp.pl?gene=MYO6
MMP16	Matrix Metallopeptidase 16	Protein Coding	52	GC08M088032	1.110368371	https://www.genecards.org/cgi-bin/carddisp.pl?gene=MMP16
CARD16	Caspase Recruitment Domain Family Member 16	Protein Coding	40	GC11M105041	1.109456182	https://www.genecards.org/cgi-bin/carddisp.pl?gene=CARD16
IL12RB2	Interleukin 12 Receptor Subunit Beta 2	Protein Coding	51	GC01P067307	1.108985543	https://www.genecards.org/cgi-bin/carddisp.pl?gene=IL12RB2
EPHB1	EPH Receptor B1	Protein Coding	55	GC03P134795	1.108498573	https://www.genecards.org/cgi-bin/carddisp.pl?gene=EPHB1
EPHA8	EPH Receptor A8	Protein Coding	51	GC01P022563	1.108498573	https://www.genecards.org/cgi-bin/carddisp.pl?gene=EPHA8
PTPN7	Protein Tyrosine Phosphatase Non-Receptor Type 7	Protein Coding	51	GC01M202147	1.108498573	https://www.genecards.org/cgi-bin/carddisp.pl?gene=PTPN7
ARC	Activity Regulated Cytoskeleton Associated Protein	Protein Coding	43	GC08M142611	1.108227015	https://www.genecards.org/cgi-bin/carddisp.pl?gene=ARC
AKAP12	A-Kinase Anchoring Protein 12	Protein Coding	48	GC06P151239	1.107064486	https://www.genecards.org/cgi-bin/carddisp.pl?gene=AKAP12
TRPC1	Transient Receptor Potential Cation Channel Subfamily C Member 1	Protein Coding	48	GC03P142724	1.106306195	https://www.genecards.org/cgi-bin/carddisp.pl?gene=TRPC1
C19orf48	Chromosome 19 Putative Open Reading Frame 48	Pseudogene	36	GC19M050797	1.105926037	https://www.genecards.org/cgi-bin/carddisp.pl?gene=C19orf48
MUC2	Mucin 2, Oligomeric Mucus/Gel-Forming	Protein Coding	44	GC11P001074	1.104578614	https://www.genecards.org/cgi-bin/carddisp.pl?gene=MUC2

KRT20	Keratin 20	Protein Coding	46	GC17M040875	1.095470786	https://www.genecards.org/cgi-bin/carddisp.pl?gene=KRT20
KIR2DS4	Killer Cell Immunoglobulin Like Receptor, Two Ig Domains And Short Cytoplasmic Tail 4	Protein Coding	37	GC19P073504	1.095470786	https://www.genecards.org/cgi-bin/carddisp.pl?gene=KIR2DS4
NAA30	N-Alpha-Acetyltransferase 30, NatC Catalytic Subunit	Protein Coding	44	GC14P057390	1.092493534	https://www.genecards.org/cgi-bin/carddisp.pl?gene=NAA30
EPHB3	EPH Receptor B3	Protein Coding	53	GC03P184561	1.091133595	https://www.genecards.org/cgi-bin/carddisp.pl?gene=EPHB3
CDK20	Cyclin Dependent Kinase 20	Protein Coding	49	GC09M087966	1.091133595	https://www.genecards.org/cgi-bin/carddisp.pl?gene=CDK20
PTPRR	Protein Tyrosine Phosphatase Receptor Type R	Protein Coding	45	GC12M070638	1.091133595	https://www.genecards.org/cgi-bin/carddisp.pl?gene=PTPRR
ZFP36L1	ZFP36 Ring Finger Protein Like 1	Protein Coding	47	GC14M068787	1.089681864	https://www.genecards.org/cgi-bin/carddisp.pl?gene=ZFP36L1
RAP1B	RAP1B, Member Of RAS Oncogene Family	Protein Coding	50	GC12P068610	1.089631319	https://www.genecards.org/cgi-bin/carddisp.pl?gene=RAP1B
STMN2	Stathmin 2	Protein Coding	45	GC08P079610	1.086361289	https://www.genecards.org/cgi-bin/carddisp.pl?gene=STMN2
ADGRE5	Adhesion G Protein-Coupled Receptor E5	Protein Coding	51	GC19P014880	1.082407951	https://www.genecards.org/cgi-bin/carddisp.pl?gene=ADGRE5
GDF5	Growth Differentiation Factor 5	Protein Coding	52	GC20M035433	1.08078897	https://www.genecards.org/cgi-bin/carddisp.pl?gene=GDF5
VAV3	Vav Guanine Nucleotide Exchange Factor 3	Protein Coding	50	GC01M107571	1.078908563	https://www.genecards.org/cgi-bin/carddisp.pl?gene=VAV3
F2RL2	Coagulation Factor II Thrombin Receptor Like 2	Protein Coding	47	GC05M076615	1.078908563	https://www.genecards.org/cgi-bin/carddisp.pl?gene=F2RL2
ANGPTL1	Angiopoietin Like 1	Protein Coding	46	GC01M178849	1.078908563	https://www.genecards.org/cgi-bin/carddisp.pl?gene=ANGPTL1
RGS16	Regulator Of G Protein Signaling 16	Protein Coding	46	GC01M182598	1.078908563	https://www.genecards.org/cgi-bin/carddisp.pl?gene=RGS16

OAS3	2'-5'-Oligoadenylate Synthetase 3	Protein Coding	46	GC12P112938	1.077958465	https://www.genecards.org/cgi-bin/carddisp.pl?gene=OAS3
NMT2	N-Myristoyltransferase 2	Protein Coding	45	GC10M015206	1.072276354	https://www.genecards.org/cgi-bin/carddisp.pl?gene=NMT2
PTPRZ1	Protein Tyrosine Phosphatase Receptor Type Z1	Protein Coding	52	GC07P121873	1.068503141	https://www.genecards.org/cgi-bin/carddisp.pl?gene=PTPRZ1
PTPN4	Protein Tyrosine Phosphatase Non-Receptor Type 4	Protein Coding	47	GC02P119759	1.068503141	https://www.genecards.org/cgi-bin/carddisp.pl?gene=PTPN4
MT1E	Metallothionein 1E	Protein Coding	43	GC16P056625	1.067718744	https://www.genecards.org/cgi-bin/carddisp.pl?gene=MT1E
F2RL3	F2R Like Thrombin Or Trypsin Receptor 3	Protein Coding	53	GC19P016888	1.067248344	https://www.genecards.org/cgi-bin/carddisp.pl?gene=F2RL3
WWC1	WW And C2 Domain Containing 1	Protein Coding	47	GC05P168291	1.058328509	https://www.genecards.org/cgi-bin/carddisp.pl?gene=WWC1
BTC	Betacellulin	Protein Coding	51	GC04M074744	1.054350853	https://www.genecards.org/cgi-bin/carddisp.pl?gene=BTC
IRF9	Interferon Regulatory Factor 9	Protein Coding	51	GC14P024161	1.054350853	https://www.genecards.org/cgi-bin/carddisp.pl?gene=IRF9
KRT15	Keratin 15	Protein Coding	44	GC17M041513	1.054350853	https://www.genecards.org/cgi-bin/carddisp.pl?gene=KRT15
GTF3A	General Transcription Factor IIIA	Protein Coding	42	GC13P027429	1.052797198	https://www.genecards.org/cgi-bin/carddisp.pl?gene=GTF3A
C1S	Complement C1s	Protein Coding	54	GC12P023568	1.051964879	https://www.genecards.org/cgi-bin/carddisp.pl?gene=C1S
UNC13C	Unc-13 Homolog C	Protein Coding	40	GC15P053838	1.048702359	https://www.genecards.org/cgi-bin/carddisp.pl?gene=UNC13C
ITGB7	Integrin Subunit Beta 7	Protein Coding	52	GC12M053191	1.042477012	https://www.genecards.org/cgi-bin/carddisp.pl?gene=ITGB7
CD177	CD177 Molecule	Protein Coding	46	GC19P043353	1.040836096	https://www.genecards.org/cgi-bin/carddisp.pl?gene=CD177

GSDMC	Gasdermin C	Protein Coding	40	GC08M129705	1.03986001	https://www.genecards.org/cgi-bin/carddisp.pl?gene=GSDMC
HRG	Histidine Rich Glycoprotein	Protein Coding	52	GC03P186660	1.039795876	https://www.genecards.org/cgi-bin/carddisp.pl?gene=HRG
EFNA5	Ephrin A5	Protein Coding	51	GC05M107376	1.039711475	https://www.genecards.org/cgi-bin/carddisp.pl?gene=EFNA5
RASGRF1	Ras Protein Specific Guanine Nucleotide Releasing Factor 1	Protein Coding	50	GC15M078959	1.039711475	https://www.genecards.org/cgi-bin/carddisp.pl?gene=RASGRF1
VAV2	Vav Guanine Nucleotide Exchange Factor 2	Protein Coding	49	GC09M133761	1.039711475	https://www.genecards.org/cgi-bin/carddisp.pl?gene=VAV2
DSG3	Desmoglein 3	Protein Coding	48	GC18P031447	1.039711475	https://www.genecards.org/cgi-bin/carddisp.pl?gene=DSG3
NCK2	NCK Adaptor Protein 2	Protein Coding	48	GC02P105744	1.039711475	https://www.genecards.org/cgi-bin/carddisp.pl?gene=NCK2
SLAMF6	SLAM Family Member 6	Protein Coding	47	GC01M160454	1.039711475	https://www.genecards.org/cgi-bin/carddisp.pl?gene=SLAMF6
GEM	GTP Binding Protein Overexpressed In Skeletal Muscle	Protein Coding	46	GC08M094249	1.039711475	https://www.genecards.org/cgi-bin/carddisp.pl?gene=GEM
RHBDF1	Rhomboid 5 Homolog 1	Protein Coding	45	GC16M000058	1.039711475	https://www.genecards.org/cgi-bin/carddisp.pl?gene=RHBDF1
SIGLEC12	Sialic Acid Binding Ig Like Lectin 12	Protein Coding	45	GC19M051491	1.039711475	https://www.genecards.org/cgi-bin/carddisp.pl?gene=SIGLEC12
ERRFI1	ERBB Receptor Feedback Inhibitor 1	Protein Coding	43	GC01M008004	1.039711475	https://www.genecards.org/cgi-bin/carddisp.pl?gene=ERRFI1
GRM8	Glutamate Metabotropic Receptor 8	Protein Coding	53	GC07M126438	1.037789106	https://www.genecards.org/cgi-bin/carddisp.pl?gene=GRM8
IL17RD	Interleukin 17 Receptor D	Protein Coding	52	GC03M057089	1.029799223	https://www.genecards.org/cgi-bin/carddisp.pl?gene=IL17RD
LOC111674472	DNase I Hypersensitive Sites In Introns 16 And 17a Of CFTR	Functional Element	4	GC07P117607	1.027813554	https://www.genecards.org/cgi-bin/carddisp.pl?gene=LOC111674472

NRCAM	Neuronal Cell Adhesion Molecule	Protein Coding	51	GC07M108147	1.022346497	https://www.genecards.org/cgi-bin/carddisp.pl?gene=NRCAM
CD300A	CD300a Molecule	Protein Coding	47	GC17P074466	1.022346497	https://www.genecards.org/cgi-bin/carddisp.pl?gene=CD300A
HTN3	Histatin 3	Protein Coding	38	GC04P070028	1.022087574	https://www.genecards.org/cgi-bin/carddisp.pl?gene=HTN3
GJA4	Gap Junction Protein Alpha 4	Protein Coding	50	GC01P034792	1.019160032	https://www.genecards.org/cgi-bin/carddisp.pl?gene=GJA4
EPHA5	EPH Receptor A5	Protein Coding	53	GC04M065319	1.013868332	https://www.genecards.org/cgi-bin/carddisp.pl?gene=EPHA5
CDKL1	Cyclin Dependent Kinase Like 1	Protein Coding	46	GC14M050330	1.013868332	https://www.genecards.org/cgi-bin/carddisp.pl?gene=CDKL1
SEMA3B	Semaphorin 3B	Protein Coding	47	GC03P050267	1.013504267	https://www.genecards.org/cgi-bin/carddisp.pl?gene=SEMA3B
METTL2B	Methyltransferase 2B, Methylcytidine	Protein Coding	40	GC07P132479	1.013077974	https://www.genecards.org/cgi-bin/carddisp.pl?gene=METTL2B
PAG1	Phosphoprotein Membrane Anchor With Glycosphingolipid Microdomains 1	Protein Coding	46	GC08M080967	1.012400031	https://www.genecards.org/cgi-bin/carddisp.pl?gene=PAG1
INA	Internexin Neuronal Intermediate Filament Protein Alpha	Protein Coding	45	GC10P103277	1.011909842	https://www.genecards.org/cgi-bin/carddisp.pl?gene=INA
SLC17A6	Solute Carrier Family 17 Member 6	Protein Coding	47	GC11P022359	1.005619407	https://www.genecards.org/cgi-bin/carddisp.pl?gene=SLC17A6
NPTX1	Neuronal Pentraxin 1	Protein Coding	47	GC17M080466	1.002447128	https://www.genecards.org/cgi-bin/carddisp.pl?gene=NPTX1
IL5RA	Interleukin 5 Receptor Subunit Alpha	Protein Coding	53	GC03M003066	1.001373172	https://www.genecards.org/cgi-bin/carddisp.pl?gene=IL5RA
LGR6	Leucine Rich Repeat Containing G Protein-Coupled Receptor 6	Protein Coding	48	GC01P202193	1.000739813	https://www.genecards.org/cgi-bin/carddisp.pl?gene=LGR6
SNX1	Sorting Nexin 1	Protein Coding	46	GC15P064094	1.000739813	https://www.genecards.org/cgi-bin/carddisp.pl?gene=SNX1

KIR2DL3	Killer Cell Immunoglobulin Like Receptor, Two Ig Domains And Long Cytoplasmic Tail 3	Protein Coding	43	GC19P074381	1.000186682	https://www.genecards.org/cgi-bin/carddisp.pl?gene=KIR2DL3
KLRB1	Killer Cell Lectin Like Receptor B1	Protein Coding	47	GC12M022622	0.999716043	https://www.genecards.org/cgi-bin/carddisp.pl?gene=KLRB1
PITX3	Paired Like Homeodomain 3	Protein Coding	46	GC10M102230	0.999716043	https://www.genecards.org/cgi-bin/carddisp.pl?gene=PITX3
GPR182	G Protein-Coupled Receptor 182	Protein Coding	44	GC12P056994	0.999716043	https://www.genecards.org/cgi-bin/carddisp.pl?gene=GPR182
CHRNE	Cholinergic Receptor Nicotinic Epsilon Subunit	Protein Coding	51	GC17M004897	0.999406278	https://www.genecards.org/cgi-bin/carddisp.pl?gene=CHRNE
TUB	TUB Bipartite Transcription Factor	Protein Coding	50	GC11P008040	0.994702637	https://www.genecards.org/cgi-bin/carddisp.pl?gene=TUB
EPS8	Epidermal Growth Factor Receptor Pathway Substrate 8	Protein Coding	52	GC12M022709	0.987842262	https://www.genecards.org/cgi-bin/carddisp.pl?gene=EPS8
UBASH3B	Ubiquitin Associated And SH3 Domain Containing B	Protein Coding	48	GC11P122655	0.987842262	https://www.genecards.org/cgi-bin/carddisp.pl?gene=UBASH3B
ASAP1	ArfGAP With SH3 Domain, Ankyrin Repeat And PH Domain 1	Protein Coding	47	GC08M130052	0.987842262	https://www.genecards.org/cgi-bin/carddisp.pl?gene=ASAP1
SKAP2	Src Kinase Associated Phosphoprotein 2	Protein Coding	46	GC07M026654	0.987842262	https://www.genecards.org/cgi-bin/carddisp.pl?gene=SKAP2
KCNA2	Potassium Voltage-Gated Channel Subfamily A Member 2	Protein Coding	53	GC01M110519	0.985289454	https://www.genecards.org/cgi-bin/carddisp.pl?gene=KCNA2
IGF2BP2-AS1	IGF2BP2 Antisense RNA 1	RNA Gene	26	GC03P185620	0.982193828	https://www.genecards.org/cgi-bin/carddisp.pl?gene=IGF2BP2-AS1
SKAP1	Src Kinase Associated Phosphoprotein 1	Protein Coding	45	GC17M048133	0.980487883	https://www.genecards.org/cgi-bin/carddisp.pl?gene=SKAP1
NAA60	N-Alpha-Acetyltransferase 60, NatF Catalytic Subunit	Protein Coding	43	GC16P003443	0.97929728	https://www.genecards.org/cgi-bin/carddisp.pl?gene=NAA60
PURA	Purine Rich Element Binding Protein A	Protein Coding	51	GC05P140076	0.976546884	https://www.genecards.org/cgi-bin/carddisp.pl?gene=PURA

MEOX2	Mesenchyme Homeobox 2	Protein Coding	46	GC07M015617	0.976295114	https://www.genecards.org/cgi-bin/carddisp.pl?gene=MEOX2
OXER1	Oxoeicosanoid Receptor 1	Protein Coding	45	GC02M042762	0.973202944	https://www.genecards.org/cgi-bin/carddisp.pl?gene=OXER1
GRIN3B	Glutamate Ionotropic Receptor NMDA Type Subunit 3B	Protein Coding	49	GC19P001000	0.957497299	https://www.genecards.org/cgi-bin/carddisp.pl?gene=GRIN3B
RIN1	Ras And Rab Interactor 1	Protein Coding	44	GC11M099757	0.955837965	https://www.genecards.org/cgi-bin/carddisp.pl?gene=RIN1
TOM1L1	Target Of Myb1 Like 1 Membrane Trafficking Protein	Protein Coding	42	GC17P062999	0.955837965	https://www.genecards.org/cgi-bin/carddisp.pl?gene=TOM1L1
SH3BGRL2	SH3 Domain Binding Glutamate Rich Protein Like 2	Protein Coding	40	GC06P092694	0.955837965	https://www.genecards.org/cgi-bin/carddisp.pl?gene=SH3BGRL2
FZD9	Frizzled Class Receptor 9	Protein Coding	51	GC07P073433	0.95253402	https://www.genecards.org/cgi-bin/carddisp.pl?gene=FZD9
CADM2	Cell Adhesion Molecule 2	Protein Coding	47	GC03P084961	0.952244639	https://www.genecards.org/cgi-bin/carddisp.pl?gene=CADM2
NUDT6	Nudix Hydrolase 6	Protein Coding	43	GC04M122888	0.950953603	https://www.genecards.org/cgi-bin/carddisp.pl?gene=NUDT6
ZDHHC3	Zinc Finger DHHC-Type Palmitoyltransferase 3	Protein Coding	43	GC03M044915	0.950109363	https://www.genecards.org/cgi-bin/carddisp.pl?gene=ZDHHC3
PCDH15	Protocadherin Related 15	Protein Coding	48	GC10M053802	0.946790814	https://www.genecards.org/cgi-bin/carddisp.pl?gene=PCDH15
HGFAC	HGF Activator	Protein Coding	48	GC04P003443	0.945081234	https://www.genecards.org/cgi-bin/carddisp.pl?gene=HGFAC
PLXNC1	Plexin C1	Protein Coding	47	GC12P094150	0.945081234	https://www.genecards.org/cgi-bin/carddisp.pl?gene=PLXNC1
SLA2	Src Like Adaptor 2	Protein Coding	46	GC20M036613	0.945081234	https://www.genecards.org/cgi-bin/carddisp.pl?gene=SLA2
LILRB4	Leukocyte Immunoglobulin Like Receptor B4	Protein Coding	44	GC19P054643	0.945081234	https://www.genecards.org/cgi-bin/carddisp.pl?gene=LILRB4

RASA3	RAS P21 Protein Activator 3	Protein Coding	44	GC13M113977	0.945081234	https://www.genecards.org/cgi-bin/carddisp.pl?gene=RASA3
FCGR2C	Fc Gamma Receptor IIc (Gene/Pseudogene)	Protein Coding	40	GC01P161818	0.945081234	https://www.genecards.org/cgi-bin/carddisp.pl?gene=FCGR2C
SPAG4	Sperm Associated Antigen 4	Protein Coding	40	GC20P035615	0.945081234	https://www.genecards.org/cgi-bin/carddisp.pl?gene=SPAG4
GYPE	Glycophorin E (MNS Blood Group)	Protein Coding	35	GC04M143870	0.941309452	https://www.genecards.org/cgi-bin/carddisp.pl?gene=GYPE
GFRA3	GDNF Family Receptor Alpha 3	Protein Coding	50	GC05M138263	0.933207512	https://www.genecards.org/cgi-bin/carddisp.pl?gene=GFRA3
GRAP2	GRB2 Related Adaptor Protein 2	Protein Coding	50	GC22P045214	0.933207512	https://www.genecards.org/cgi-bin/carddisp.pl?gene=GRAP2
STON2	Stonin 2	Protein Coding	41	GC14M081260	0.933207512	https://www.genecards.org/cgi-bin/carddisp.pl?gene=STON2
GNRH2	Gonadotropin Releasing Hormone 2	Protein Coding	40	GC20P003043	0.933207512	https://www.genecards.org/cgi-bin/carddisp.pl?gene=GNRH2
MSLN	Mesothelin	Protein Coding	50	GC16P013455	0.93258357	https://www.genecards.org/cgi-bin/carddisp.pl?gene=MSLN
ECE2	Endothelin Converting Enzyme 2	Protein Coding	46	GC03P184276	0.928997397	https://www.genecards.org/cgi-bin/carddisp.pl?gene=ECE2
S100G	S100 Calcium Binding Protein G	Protein Coding	42	GC0XP016668	0.925257087	https://www.genecards.org/cgi-bin/carddisp.pl?gene=S100G
COL17A1	Collagen Type XVII Alpha 1 Chain	Protein Coding	51	GC10M104031	0.92325455	https://www.genecards.org/cgi-bin/carddisp.pl?gene=COL17A1
LNX1	Ligand Of Numb-Protein X 1	Protein Coding	48	GC04M053459	0.92325455	https://www.genecards.org/cgi-bin/carddisp.pl?gene=LNX1
GPR17	G Protein-Coupled Receptor 17	Protein Coding	48	GC02P127645	0.922787964	https://www.genecards.org/cgi-bin/carddisp.pl?gene=GPR17
UBE2NL	Ubiquitin Conjugating Enzyme E2 N Like (Gene/Pseudogene)	Protein Coding	31	GC0XP143884	0.920240343	https://www.genecards.org/cgi-bin/carddisp.pl?gene=UBE2NL

AKAP5	A-Kinase Anchoring Protein 5	Protein Coding	47	GC14P064465	0.917392969	https://www.genecards.org/cgi-bin/carddisp.pl?gene=AKAP5
DAB1	DAB Adaptor Protein 1	Protein Coding	50	GC01M056994	0.912124336	https://www.genecards.org/cgi-bin/carddisp.pl?gene=DAB1
AFDN	Afadin, Adherens Junction Formation Factor	Protein Coding	48	GC06P167827	0.912124336	https://www.genecards.org/cgi-bin/carddisp.pl?gene=AFDN
SLC9A6	Solute Carrier Family 9 Member A6	Protein Coding	53	GC0XP135985	0.905902743	https://www.genecards.org/cgi-bin/carddisp.pl?gene=SLC9A6
ZDHHC7	Zinc Finger DHHC-Type Palmitoyltransferase 7	Protein Coding	43	GC16M084975	0.905520856	https://www.genecards.org/cgi-bin/carddisp.pl?gene=ZDHHC7
ZDHHC4	Zinc Finger DHHC-Type Palmitoyltransferase 4	Protein Coding	40	GC07P006577	0.905520856	https://www.genecards.org/cgi-bin/carddisp.pl?gene=ZDHHC4
JMJD6	Jumonji Domain Containing 6, Arginine Demethylase And Lysine Hydroxylase	Protein Coding	49	GC17M076718	0.904907465	https://www.genecards.org/cgi-bin/carddisp.pl?gene=JMJD6
METAP2	Methionyl Aminopeptidase 2	Protein Coding	52	GC12P095473	0.903258324	https://www.genecards.org/cgi-bin/carddisp.pl?gene=METAP2
ABT1	Activator Of Basal Transcription 1	Protein Coding	41	GC06P091965	0.903258324	https://www.genecards.org/cgi-bin/carddisp.pl?gene=ABT1
EDN2	Endothelin 2	Protein Coding	47	GC01M041478	0.900304377	https://www.genecards.org/cgi-bin/carddisp.pl?gene=EDN2
ZC2HC1A	Zinc Finger C2HC-Type Containing 1A	Protein Coding	38	GC08P078666	0.89760989	https://www.genecards.org/cgi-bin/carddisp.pl?gene=ZC2HC1A
RAB39B	RAB39B, Member RAS Oncogene Family	Protein Coding	44	GC0XM155259	0.896075845	https://www.genecards.org/cgi-bin/carddisp.pl?gene=RAB39B
SLC24A3	Solute Carrier Family 24 Member 3	Protein Coding	46	GC20P019212	0.889909029	https://www.genecards.org/cgi-bin/carddisp.pl?gene=SLC24A3
DRAM1	DNA Damage Regulated Autophagy Modulator 1	Protein Coding	37	GC12P101877	0.887507498	https://www.genecards.org/cgi-bin/carddisp.pl?gene=DRAM1
LOC108663987	Ataxin 3 Repeat Instability Region	Functional Element	4	GC14P092071	0.887507498	https://www.genecards.org/cgi-bin/carddisp.pl?gene=LOC108663987

ERBIN	Erbb2 Interacting Protein	Protein Coding	46	GC05P065884	0.88390404	https://www.genecards.org/cgi-bin/carddisp.pl?gene=ERBIN
TRPC4	Transient Receptor Potential Cation Channel Subfamily C Member 4	Protein Coding	50	GC13M037638	0.878572762	https://www.genecards.org/cgi-bin/carddisp.pl?gene=TRPC4
ARTN	Artemin	Protein Coding	47	GC01P043933	0.878572762	https://www.genecards.org/cgi-bin/carddisp.pl?gene=ARTN
LILRA4	Leukocyte Immunoglobulin Like Receptor A4	Protein Coding	46	GC19M054333	0.878572762	https://www.genecards.org/cgi-bin/carddisp.pl?gene=LILRA4
MUC3B	Mucin 3B, Cell Surface Associated	Protein Coding	22	GC07U903146	0.878572762	https://www.genecards.org/cgi-bin/carddisp.pl?gene=MUC3B
ATP6V1D	ATPase H+ Transporting V1 Subunit D	Protein Coding	47	GC14M067294	0.878209412	https://www.genecards.org/cgi-bin/carddisp.pl?gene=ATP6V1D
MMP25	Matrix Metallopeptidase 25	Protein Coding	47	GC16P013548	0.873746693	https://www.genecards.org/cgi-bin/carddisp.pl?gene=MMP25
NTN1	Netrin 1	Protein Coding	52	GC17P011813	0.873181343	https://www.genecards.org/cgi-bin/carddisp.pl?gene=NTN1
ZMYM2	Zinc Finger MYM-Type Containing 2	Protein Coding	48	GC13P020139	0.873181343	https://www.genecards.org/cgi-bin/carddisp.pl?gene=ZMYM2
P2RX1	Purinergic Receptor P2X 1	Protein Coding	50	GC17M003896	0.867829382	https://www.genecards.org/cgi-bin/carddisp.pl?gene=P2RX1
CNTN2	Contactin 2	Protein Coding	52	GC01P205043	0.861521125	https://www.genecards.org/cgi-bin/carddisp.pl?gene=CNTN2
LILRB1	Leukocyte Immunoglobulin Like Receptor B1	Protein Coding	50	GC19P073495	0.861521125	https://www.genecards.org/cgi-bin/carddisp.pl?gene=LILRB1
PAEP	Progestagen Associated Endometrial Protein	Protein Coding	47	GC09P135561	0.861521125	https://www.genecards.org/cgi-bin/carddisp.pl?gene=PAEP
TOB1	Transducer Of ERBB2, 1	Protein Coding	47	GC17M050862	0.861521125	https://www.genecards.org/cgi-bin/carddisp.pl?gene=TOB1
SLC17A7	Solute Carrier Family 17 Member 7	Protein Coding	47	GC19M049429	0.850808859	https://www.genecards.org/cgi-bin/carddisp.pl?gene=SLC17A7

RXFP1	Relaxin Family Peptide Receptor 1	Protein Coding	51	GC04P158315	0.848623574	https://www.genecards.org/cgi-bin/carddisp.pl?gene=RXFP1
ARHGAP35	Rho GTPase Activating Protein 35	Protein Coding	46	GC19P046860	0.848623574	https://www.genecards.org/cgi-bin/carddisp.pl?gene=ARHGAP35
SHC3	SHC Adaptor Protein 3	Protein Coding	46	GC09M089005	0.848623574	https://www.genecards.org/cgi-bin/carddisp.pl?gene=SHC3
SWAP70	Switching B Cell Complex Subunit SWAP70	Protein Coding	46	GC11P009664	0.848623574	https://www.genecards.org/cgi-bin/carddisp.pl?gene=SWAP70
SH2D2A	SH2 Domain Containing 2A	Protein Coding	45	GC01M156952	0.848623574	https://www.genecards.org/cgi-bin/carddisp.pl?gene=SH2D2A
SH3BP5	SH3 Domain Binding Protein 5	Protein Coding	44	GC03M021849	0.848623574	https://www.genecards.org/cgi-bin/carddisp.pl?gene=SH3BP5
TCEAL1	Transcription Elongation Factor A Like 1	Protein Coding	42	GC0XP103628	0.848623574	https://www.genecards.org/cgi-bin/carddisp.pl?gene=TCEAL1
ETV7	ETS Variant Transcription Factor 7	Protein Coding	40	GC06M071871	0.848623574	https://www.genecards.org/cgi-bin/carddisp.pl?gene=ETV7
LOC344967	Acyl-CoA Thioesterase 7 Pseudogene	Pseudogene	15	GC04M040045	0.848313689	https://www.genecards.org/cgi-bin/carddisp.pl?gene=LOC344967
KIF5C	Kinesin Family Member 5C	Protein Coding	48	GC02P148875	0.842474699	https://www.genecards.org/cgi-bin/carddisp.pl?gene=KIF5C
ANGPT4	Angiopoietin 4	Protein Coding	49	GC20M000869	0.833984256	https://www.genecards.org/cgi-bin/carddisp.pl?gene=ANGPT4
DOK2	Docking Protein 2	Protein Coding	49	GC08M021908	0.833984256	https://www.genecards.org/cgi-bin/carddisp.pl?gene=DOK2
CD48	CD48 Molecule	Protein Coding	47	GC01M160648	0.833984256	https://www.genecards.org/cgi-bin/carddisp.pl?gene=CD48
FCAR	Fc Alpha Receptor	Protein Coding	47	GC19P073506	0.833984256	https://www.genecards.org/cgi-bin/carddisp.pl?gene=FCAR
ADRA1D	Adrenoceptor Alpha 1D	Protein Coding	51	GC20M004220	0.828397751	https://www.genecards.org/cgi-bin/carddisp.pl?gene=ADRA1D

CHRNB4	Cholinergic Receptor Nicotinic Beta 4 Subunit	Protein Coding	51	GC15M078624	0.817885756	https://www.genecards.org/cgi-bin/carddisp.pl?gene=CHRNB4
SLAMF1	Signaling Lymphocytic Activation Molecule Family Member 1	Protein Coding	49	GC01M160608	0.816619277	https://www.genecards.org/cgi-bin/carddisp.pl?gene=SLAMF1
EFNB3	Ephrin B3	Protein Coding	48	GC17P011746	0.816619277	https://www.genecards.org/cgi-bin/carddisp.pl?gene=EFNB3
GRAP	GRB2 Related Adaptor Protein	Protein Coding	48	GC17M050970	0.816619277	https://www.genecards.org/cgi-bin/carddisp.pl?gene=GRAP
ARHGAP32	Rho GTPase Activating Protein 32	Protein Coding	46	GC11M128965	0.816619277	https://www.genecards.org/cgi-bin/carddisp.pl?gene=ARHGAP32
SH2D1B	SH2 Domain Containing 1B	Protein Coding	44	GC01M162395	0.816619277	https://www.genecards.org/cgi-bin/carddisp.pl?gene=SH2D1B
ERVW-1	Endogenous Retrovirus Group W Member 1, Envelope	Protein Coding	42	GC07M092468	0.816619277	https://www.genecards.org/cgi-bin/carddisp.pl?gene=ERVW-1
INSL4	Insulin Like 4	Protein Coding	39	GC09P005231	0.816619277	https://www.genecards.org/cgi-bin/carddisp.pl?gene=INSL4
ZDHHC9	Zinc Finger DHHC-Type Palmitoyltransferase 9	Protein Coding	50	GC0XM129803	0.810890675	https://www.genecards.org/cgi-bin/carddisp.pl?gene=ZDHHC9
ZDHHC11	Zinc Finger DHHC-Type Containing 11	Protein Coding	41	GC05M000795	0.810890675	https://www.genecards.org/cgi-bin/carddisp.pl?gene=ZDHHC11
ZDHHC11B	Zinc Finger DHHC-Type Containing 11B	Protein Coding	32	GC05M000710	0.810890675	https://www.genecards.org/cgi-bin/carddisp.pl?gene=ZDHHC11B
INTS2	Integrator Complex Subunit 2	Protein Coding	40	GC17M061865	0.804532051	https://www.genecards.org/cgi-bin/carddisp.pl?gene=INTS2
DPYSL3	Dihydropyrimidinase Like 3	Protein Coding	46	GC05M147390	0.798763692	https://www.genecards.org/cgi-bin/carddisp.pl?gene=DPYSL3
GZMA	Granzyme A	Protein Coding	50	GC05P055102	0.793988824	https://www.genecards.org/cgi-bin/carddisp.pl?gene=GZMA
RPH3A	Rabphilin 3A	Protein Coding	46	GC12P112570	0.793988824	https://www.genecards.org/cgi-bin/carddisp.pl?gene=RPH3A

SEZ6L2	Seizure Related 6 Homolog Like 2	Protein Coding	46	GC16M038421	0.793988824	https://www.genecards.org/cgi-bin/carddisp.pl?gene=SEZ6L2
CNKSR1	Connector Enhancer Of Kinase Suppressor Of Ras 1	Protein Coding	44	GC01P026178	0.793988824	https://www.genecards.org/cgi-bin/carddisp.pl?gene=CNKSR1
FRS3	Fibroblast Growth Factor Receptor Substrate 3	Protein Coding	40	GC06M071935	0.793988824	https://www.genecards.org/cgi-bin/carddisp.pl?gene=FRS3
PTPRCAP	Protein Tyrosine Phosphatase Receptor Type C Associated Protein	Protein Coding	40	GC11M099809	0.793988824	https://www.genecards.org/cgi-bin/carddisp.pl?gene=PTPRCAP
OCM2	Oncomodulin 2	Protein Coding	33	GC07M097984	0.793988824	https://www.genecards.org/cgi-bin/carddisp.pl?gene=OCM2
AAVS1	Adeno-Associated Virus Integration Site 1	Genetic Locus	6	GC19U990001	0.793988824	https://www.genecards.org/cgi-bin/carddisp.pl?gene=AAVS1
NM	Neutrophil Migration	Genetic Locus	6	GC07U990060	0.793988824	https://www.genecards.org/cgi-bin/carddisp.pl?gene=NM
ZFP36L2	ZFP36 Ring Finger Protein Like 2	Protein Coding	44	GC02M043184	0.773427129	https://www.genecards.org/cgi-bin/carddisp.pl?gene=ZFP36L2
SLC1A6	Solute Carrier Family 1 Member 6	Protein Coding	51	GC19M014921	0.773018479	https://www.genecards.org/cgi-bin/carddisp.pl?gene=SLC1A6
LDC1P	Leucine Decarboxylase 1, Pseudogene	Pseudogene	13	GC01P031501	0.771194696	https://www.genecards.org/cgi-bin/carddisp.pl?gene=LDC1P
PDCD1LG2	Programmed Cell Death 1 Ligand 2	Protein Coding	50	GC09P005510	0.767283797	https://www.genecards.org/cgi-bin/carddisp.pl?gene=PDCD1LG2
IL1RL1	Interleukin 1 Receptor Like 1	Protein Coding	48	GC02P102294	0.767182589	https://www.genecards.org/cgi-bin/carddisp.pl?gene=IL1RL1
C1R	Complement C1r	Protein Coding	53	GC12M007993	0.765953422	https://www.genecards.org/cgi-bin/carddisp.pl?gene=C1R
PGGT1B	Protein Geranylgeranyltransferase Type I Subunit Beta	Protein Coding	42	GC05M115200	0.741063714	https://www.genecards.org/cgi-bin/carddisp.pl?gene=PGGT1B
TAPBP	TAP Binding Protein	Protein Coding	51	GC06M033299	0.738563597	https://www.genecards.org/cgi-bin/carddisp.pl?gene=TAPBP

HEBP1	Heme Binding Protein 1	Protein Coding	40	GC12M012974	0.737105608	https://www.genecards.org/cgi-bin/carddisp.pl?gene=HEBP1
GRIK5	Glutamate Ionotropic Receptor Kainate Type Subunit 5	Protein Coding	52	GC19M041998	0.736668408	https://www.genecards.org/cgi-bin/carddisp.pl?gene=GRIK5
POLH	DNA Polymerase Eta	Protein Coding	55	GC06P043576	0.727330506	https://www.genecards.org/cgi-bin/carddisp.pl?gene=POLH
GTF3C4	General Transcription Factor IIIC Subunit 4	Protein Coding	44	GC09P132671	0.727330506	https://www.genecards.org/cgi-bin/carddisp.pl?gene=GTF3C4
SHANK3	SH3 And Multiple Ankyrin Repeat Domains 3	Protein Coding	45	GC22P050674	0.725786924	https://www.genecards.org/cgi-bin/carddisp.pl?gene=SHANK3
H3-4	H3.4 Histone, Cluster Member	Protein Coding	44	GC01M228427	0.722606659	https://www.genecards.org/cgi-bin/carddisp.pl?gene=H3-4
ZDHHC24	Zinc Finger DHHC-Type Containing 24	Protein Coding	40	GC11M099766	0.714433014	https://www.genecards.org/cgi-bin/carddisp.pl?gene=ZDHHC24
PADI4	Peptidyl Arginine Deiminase 4	Protein Coding	51	GC01P017308	0.704137444	https://www.genecards.org/cgi-bin/carddisp.pl?gene=PADI4
NAA20	N-Alpha-Acetyltransferase 20, NatB Catalytic Subunit	Protein Coding	47	GC20P020018	0.699793696	https://www.genecards.org/cgi-bin/carddisp.pl?gene=NAA20
ZDHHC8	Zinc Finger DHHC-Type Palmitoyltransferase 8	Protein Coding	46	GC22P020129	0.699793696	https://www.genecards.org/cgi-bin/carddisp.pl?gene=ZDHHC8
MPDZ	Multiple PDZ Domain Crumbs Cell Polarity Complex Component	Protein Coding	50	GC09M013105	0.69947648	https://www.genecards.org/cgi-bin/carddisp.pl?gene=MPDZ
PTMA	Prothymosin Alpha	Protein Coding	48	GC02P231838	0.692750692	https://www.genecards.org/cgi-bin/carddisp.pl?gene=PTMA
ZDHHC6	Zinc Finger DHHC-Type Palmitoyltransferase 6	Protein Coding	43	GC10M112430	0.682428718	https://www.genecards.org/cgi-bin/carddisp.pl?gene=ZDHHC6
JUND	JunD Proto-Oncogene, AP-1 Transcription Factor Subunit	Protein Coding	48	GC19M018279	0.682285666	https://www.genecards.org/cgi-bin/carddisp.pl?gene=JUND
GRIK3	Glutamate Ionotropic Receptor Kainate Type Subunit 3	Protein Coding	51	GC01M036795	0.680615485	https://www.genecards.org/cgi-bin/carddisp.pl?gene=GRIK3

HSPE1	Heat Shock Protein Family E (Hsp10) Member 1	Protein Coding	49	GC02P197501	0.67894119	https://www.genecards.org/cgi-bin/carddisp.pl?gene=HSPE1
PDS5B	PDS5 Cohesin Associated Factor B	Protein Coding	44	GC13P032586	0.66158253	https://www.genecards.org/cgi-bin/carddisp.pl?gene=PDS5B
ZDHHC14	Zinc Finger DHHC-Type Palmitoyltransferase 14	Protein Coding	43	GC06P157381	0.659798265	https://www.genecards.org/cgi-bin/carddisp.pl?gene=ZDHHC14
ZDHHC12	Zinc Finger DHHC-Type Palmitoyltransferase 12	Protein Coding	36	GC09M128720	0.659798265	https://www.genecards.org/cgi-bin/carddisp.pl?gene=ZDHHC12
RLN2	Relaxin 2	Protein Coding	42	GC09M005438	0.655414581	https://www.genecards.org/cgi-bin/carddisp.pl?gene=RLN2
OMP	Olfactory Marker Protein	Protein Coding	42	GC11P077102	0.645645916	https://www.genecards.org/cgi-bin/carddisp.pl?gene=OMP
NPTX2	Neuronal Pentraxin 2	Protein Coding	49	GC07P098620	0.642618299	https://www.genecards.org/cgi-bin/carddisp.pl?gene=NPTX2
PPP1R9B	Protein Phosphatase 1 Regulatory Subunit 9B	Protein Coding	47	GC17M050133	0.628742099	https://www.genecards.org/cgi-bin/carddisp.pl?gene=PPP1R9B
TNXA	Tenascin XA (Pseudogene)	Pseudogene	27	GC06M071744	0.625919342	https://www.genecards.org/cgi-bin/carddisp.pl?gene=TNXA
MYO7A	Myosin VIIA	Protein Coding	50	GC11P077128	0.625438094	https://www.genecards.org/cgi-bin/carddisp.pl?gene=MYO7A
ACKR2	Atypical Chemokine Receptor 2	Protein Coding	47	GC03P042804	0.619987786	https://www.genecards.org/cgi-bin/carddisp.pl?gene=ACKR2
SLC4A10	Solute Carrier Family 4 Member 10	Protein Coding	47	GC02P161425	0.609507203	https://www.genecards.org/cgi-bin/carddisp.pl?gene=SLC4A10
ZDHHC15	Zinc Finger DHHC-Type Palmitoyltransferase 15	Protein Coding	44	GC0XM075368	0.605163455	https://www.genecards.org/cgi-bin/carddisp.pl?gene=ZDHHC15
ZDHHC19	Zinc Finger DHHC-Type Palmitoyltransferase 19	Protein Coding	43	GC03M196197	0.605163455	https://www.genecards.org/cgi-bin/carddisp.pl?gene=ZDHHC19
ZDHHC16	Zinc Finger DHHC-Type Palmitoyltransferase 16	Protein Coding	40	GC10P097446	0.605163455	https://www.genecards.org/cgi-bin/carddisp.pl?gene=ZDHHC16

ZDHHC23	Zinc Finger DHHC-Type Palmitoyltransferase 23	Protein Coding	40	GC03P113947	0.605163455	https://www.genecards.org/cgi-bin/carddisp.pl?gene=ZDHHC23
NAA11	N-Alpha-Acetyltransferase 11, NatA Catalytic Subunit	Protein Coding	38	GC04M079155	0.605163455	https://www.genecards.org/cgi-bin/carddisp.pl?gene=NAA11
ZDHHC22	Zinc Finger DHHC-Type Palmitoyltransferase 22	Protein Coding	35	GC14M077159	0.605163455	https://www.genecards.org/cgi-bin/carddisp.pl?gene=ZDHHC22
GRIK4	Glutamate Ionotropic Receptor Kainate Type Subunit 4	Protein Coding	52	GC11P120512	0.604043722	https://www.genecards.org/cgi-bin/carddisp.pl?gene=GRIK4
GJB6	Gap Junction Protein Beta 6	Protein Coding	49	GC13M020221	0.599481165	https://www.genecards.org/cgi-bin/carddisp.pl?gene=GJB6
AGAP2	ArfGAP With GTPase Domain, Ankyrin Repeat And PH Domain 2	Protein Coding	47	GC12M057723	0.596378684	https://www.genecards.org/cgi-bin/carddisp.pl?gene=AGAP2
CACNG3	Calcium Voltage-Gated Channel Auxiliary Subunit Gamma 3	Protein Coding	48	GC16P024615	0.595825553	https://www.genecards.org/cgi-bin/carddisp.pl?gene=CACNG3
DCAF4	DDB1 And CUL4 Associated Factor 4	Protein Coding	38	GC14P072926	0.595719576	https://www.genecards.org/cgi-bin/carddisp.pl?gene=DCAF4
TACR2	Tachykinin Receptor 2	Protein Coding	50	GC10M069403	0.584081471	https://www.genecards.org/cgi-bin/carddisp.pl?gene=TACR2
TRAK1	Trafficking Kinesin Protein 1	Protein Coding	48	GC03P042020	0.580011547	https://www.genecards.org/cgi-bin/carddisp.pl?gene=TRAK1
CCR10	C-C Motif Chemokine Receptor 10	Protein Coding	42	GC17M042678	0.534312725	https://www.genecards.org/cgi-bin/carddisp.pl?gene=CCR10
TMC2	Transmembrane Channel Like 2	Protein Coding	42	GC20P002536	0.529317021	https://www.genecards.org/cgi-bin/carddisp.pl?gene=TMC2
TRPM1	Transient Receptor Potential Cation Channel Subfamily M Member 1	Protein Coding	51	GC15M031001	0.490211636	https://www.genecards.org/cgi-bin/carddisp.pl?gene=TRPM1
RSL24D1	Ribosomal L24 Domain Containing 1	Protein Coding	43	GC15M055180	0.482226133	https://www.genecards.org/cgi-bin/carddisp.pl?gene=RSL24D1
TMEM37	Transmembrane Protein 37	Protein Coding	38	GC02P119429	0.465801805	https://www.genecards.org/cgi-bin/carddisp.pl?gene=TMEM37

DEFA1	Defensin Alpha 1	Protein Coding	44	GC08M006977	0.45585534	https://www.genecards.org/cgi-bin/carddisp.pl?gene=DEFA1
KEL	Kell Metallo-Endopeptidase (Kell Blood Group)	Protein Coding	48	GC07M143027	0.439531475	https://www.genecards.org/cgi-bin/carddisp.pl?gene=KEL
GRINA	Glutamate Ionotropic Receptor NMDA Type Subunit Associated Protein 1	Protein Coding	40	GC08P143990	0.43129757	https://www.genecards.org/cgi-bin/carddisp.pl?gene=GRINA
C4BPA	Complement Component 4 Binding Protein Alpha	Protein Coding	49	GC01P207105	0.429623127	https://www.genecards.org/cgi-bin/carddisp.pl?gene=C4BPA
RCOR1	REST Corepressor 1	Protein Coding	49	GC14P102592	0.429034412	https://www.genecards.org/cgi-bin/carddisp.pl?gene=RCOR1
TMC1	Transmembrane Channel Like 1	Protein Coding	46	GC09P072521	0.390098333	https://www.genecards.org/cgi-bin/carddisp.pl?gene=TMC1
CRYBA1	Crystallin Beta A1	Protein Coding	46	GC17P029246	0.389627695	https://www.genecards.org/cgi-bin/carddisp.pl?gene=CRYBA1
TAAR6	Trace Amine Associated Receptor 6	Protein Coding	40	GC06P132570	0.389627695	https://www.genecards.org/cgi-bin/carddisp.pl?gene=TAAR6
ZNF521	Zinc Finger Protein 521	Protein Coding	43	GC18M025061	0.345749646	https://www.genecards.org/cgi-bin/carddisp.pl?gene=ZNF521
CACNG1	Calcium Voltage-Gated Channel Auxiliary Subunit Gamma 1	Protein Coding	48	GC17P067044	0.342445672	https://www.genecards.org/cgi-bin/carddisp.pl?gene=CACNG1
P2RY4	Pyrimidinergic Receptor P2Y4	Protein Coding	46	GC0XM070258	0.340865254	https://www.genecards.org/cgi-bin/carddisp.pl?gene=P2RY4
USH1C	USH1 Protein Network Component Harmonin	Protein Coding	50	GC11M017665	0.334992945	https://www.genecards.org/cgi-bin/carddisp.pl?gene=USH1C
P2RY6	Pyrimidinergic Receptor P2Y6	Protein Coding	48	GC11P073264	0.310468733	https://www.genecards.org/cgi-bin/carddisp.pl?gene=P2RY6
TRAK2	Trafficking Kinesin Protein 2	Protein Coding	47	GC02M201377	0.304976404	https://www.genecards.org/cgi-bin/carddisp.pl?gene=TRAK2
MRGPRD	MAS Related GPR Family Member D	Protein Coding	43	GC11M068980	0.288439035	https://www.genecards.org/cgi-bin/carddisp.pl?gene=MRGPRD

HBB-LCR	Beta-Globin Locus Control Region	Functional Element	7	GC11P005434	0.260074615	https://www.genecards.org/cgi-bin/carddisp.pl?gene=HBB-LCR
GRIPAP1	GRIP1 Associated Protein 1	Protein Coding	41	GC0XM048973	0.23853527	https://www.genecards.org/cgi-bin/carddisp.pl?gene=GRIPAP1
CACNG8	Calcium Voltage-Gated Channel Auxiliary Subunit Gamma 8	Protein Coding	43	GC19P073470	0.237444133	https://www.genecards.org/cgi-bin/carddisp.pl?gene=CACNG8
CACNG4	Calcium Voltage-Gated Channel Auxiliary Subunit Gamma 4	Protein Coding	48	GC17P066964	0.223895937	https://www.genecards.org/cgi-bin/carddisp.pl?gene=CACNG4
OR51E1	Olfactory Receptor Family 51 Subfamily E Member 1	Protein Coding	40	GC11P004643	0.206530973	https://www.genecards.org/cgi-bin/carddisp.pl?gene=OR51E1
PCDHGC3	Protocadherin Gamma Subfamily C, 3	Protein Coding	45	GC05P141475	0.183900505	https://www.genecards.org/cgi-bin/carddisp.pl?gene=PCDHGC3
CST2	Cystatin SA	Protein Coding	39	GC20M023805	0.183900505	https://www.genecards.org/cgi-bin/carddisp.pl?gene=CST2
