# Supplementary material for: Development of a propionate metabolism-related gene-based molecular subtypes and scoring system for predicting prognosis in bladder cancer
Source: Eur J Med Res. 2024 Jul 29;29:393. doi: 10.1186/s40001-024-01982-6 (PMC11285334; doi:10.1186/s40001-024-01982-6)
Supplement: Supplementary file 5 — Supplementary Material 5: Fig. S5. Single-cell analysis and validation of the HSD17B1 from external databases.The cell type of GSE145281_aPDL1 dataset.The principal distributions of HSD17B1 on cell types in the GSE145281_aPDL1 dataset.Expression of HSD17B1 in BLCA patients in the TISCH database.Survival analysis for the subgroup classified by HSD17B1 mRNA expression.Expression of HSD17B1 in pan-cancer patients in the TISCH database.The expression of HSD17B1 between normal and tumor was analyzed by immunohistochemistryfrom the Human Protein Atlasdataset. [file 40001_2024_1982_MOESM5_ESM.pdf]

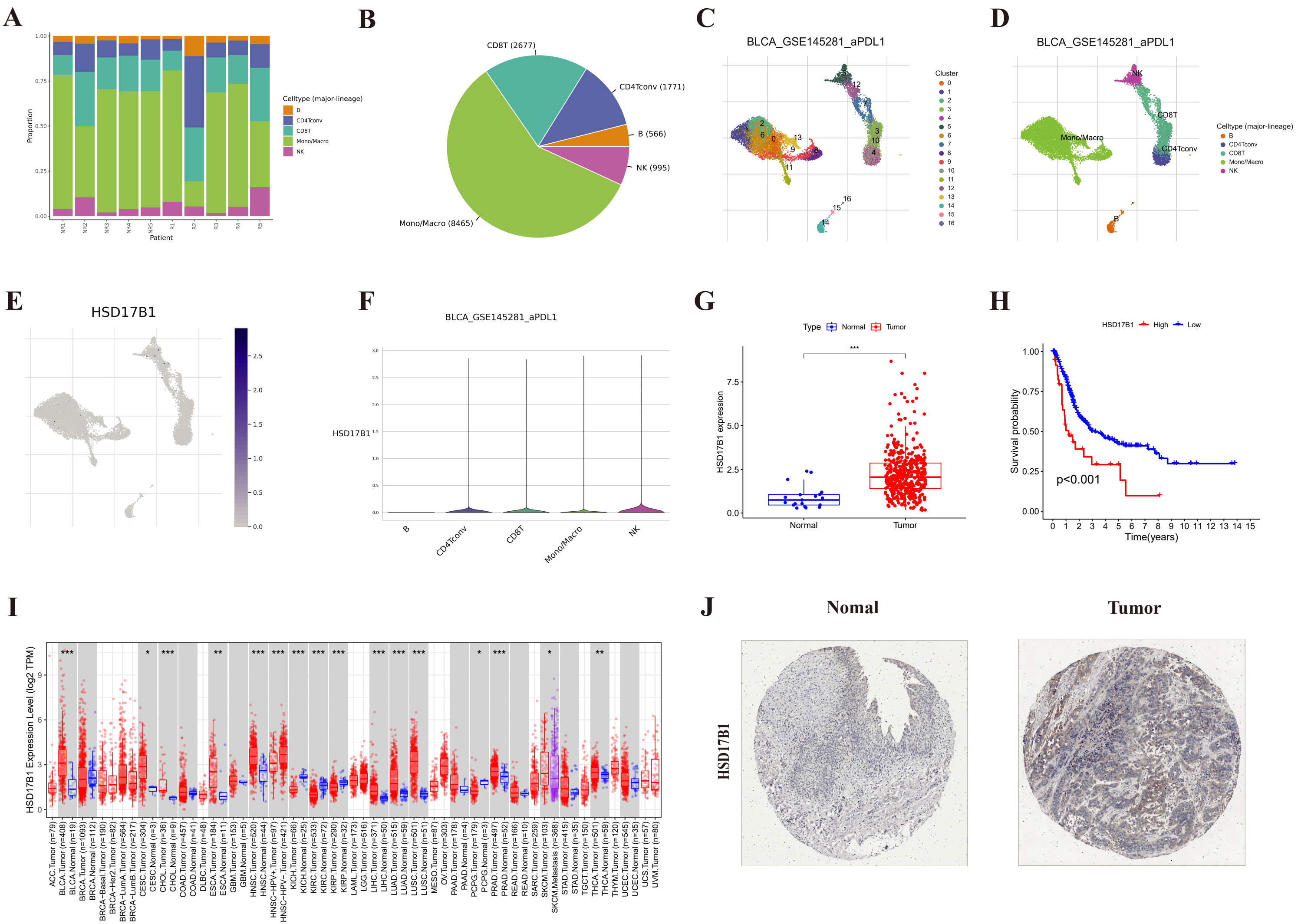
A
Proportion
Patient
Celltype (major-lineage)
B
CD4Tconv
CD8T
Mono/Macro
NK
NR1 NR2 NR3 NR4 NR5 R1 R2 R3 R4 R5
B
CD8T (2677)
CD4Tconv (1771)
B (566)
NK (995)
Mono/Macro (8465)
C
BLCA_GSE145281_aPDL1
Cluster
D
BLCA_GSE145281_aPDL1
NK
CD8T
CD4Tconv
Mono/Macro
B
E
HSD17B1
F
BLCA_GSE145281_aPDL1
HSD17B1
B
CD4Tconv
CD8T
Mono/Macro
NK
G
Type
Normal
Tumor
HSD17B1 expression
***
H
HSD17B1
High
Low
Survival probability
p<0.001
Time(years)
I
HSD17B1 Expression Level (log2 TPM)
ACC.Tumor (n=79)
BLCA.Tumor (n=408)
BLCA.Normal (n=19)
BRCA.Tumor (n=1093)
BRCA.Normal (n=112)
BRCA−Basal.Tumor (n=190)
BRCA−Her2.Tumor (n=82)
BRCA−LumA.Tumor (n=564)
BRCA−LumB.Tumor (n=217)
CESC.Tumor (n=304)
CESC.Normal (n=3)
CHOL.Tumor (n=36)
CHOL.Normal (n=9)
COAD.Tumor (n=457)
COAD.Normal (n=41)
DLBC.Tumor (n=48)
ESCA.Tumor (n=184)
ESCA.Normal (n=11)
GBM.Tumor (n=153)
GBM.Normal (n=5)
HNSC.Tumor (n=520)
HNSC.Normal (n=44)
HNSC−HPV+.Tumor (n=97)
HNSC−HPV−.Tumor (n=421)
KICH.Tumor (n=66)
KICH.Normal (n=25)
KIRC.Tumor (n=533)
KIRC.Normal (n=72)
KIRP.Tumor (n=290)
KIRP.Normal (n=32)
LAML.Tumor (n=173)
LGG.Tumor (n=516)
LIHC.Tumor (n=371)
LIHC.Normal (n=50)
LUAD.Tumor (n=515)
LUAD.Normal (n=59)
LUSC.Tumor (n=501)
LUSC.Normal (n=51)
MESO.Tumor (n=87)
OV.Tumor (n=303)
PAAD.Tumor (n=178)
PAAD.Normal (n=4)
PCPG.Tumor (n=179)
PCPG.Normal (n=3)
PRAD.Tumor (n=497)
PRAD.Normal (n=52)
READ.Tumor (n=166)
READ.Normal (n=10)
SARC.Tumor (n=259)
SKCM.Tumor (n=103)
SKCM.Metastasis (n=368)
STAD.Tumor (n=415)
STAD.Normal (n=35)
TGCT.Tumor (n=150)
THCA.Tumor (n=501)
THCA.Normal (n=59)
THYM.Tumor (n=120)
UCEC.Tumor (n=545)
UCEC.Normal (n=35)
UCS.Tumor (n=57)
UVM.Tumor (n=80)
J
Nomal
Tumor
HSD17B1
